# Giant negative photomagnetism in an alterlattice

**DOI:** 10.1038/s41467-026-76523-2

**Published:** 2026-08-07

**Authors:** Junhong Zhou, Xu Han, Haibin Huang, Taotao Huang, Yaowen Wang, Brian F. Woodfield, Liping Li, Guangshe Li

**Affiliations:** 1 State Key Laboratory of Inorganic Synthesis and Preparative Chemistry, College of Chemistry, Jilin University, Changchun, P.R. China; 2 Department of Chemistry and Biochemistry, Brigham Young University, Provo, UT USA

**Keywords:** Solid-state chemistry, Magnetic properties and materials, Structural properties, Magneto-optics, Chemical physics

## Abstract

Photomagnetism enables optical control of spin order for engineering non-equilibrium phases, yet studies remain largely confined to ferromagnetic systems. Here, we introduce the alterlattice, an emergent structural motif realized in the cation- and anion-deficient perovskite oxide La_0.4_Ca_0.4_TiO_3–δ_. The alterlattice is a three-dimensional alternately patterned superstructural network arising from structural competition, in which average lattice is described by *Ibmm* symmetry while local superstructure relaxes toward *Cmmm* symmetry. Within this motif, photo-induced antiferromagnetic correlations develop below 19 K. Under 473 nm illumination, the susceptibility of the alterlattice host decreases by 51.7% at 2 K and 1 T and rapidly recovers in the dark. The observed giant negative photomagnetism is associated with anisotropic strain fields that guide defect-mediated photocarrier transport, thereby driving the system from a spin-dilute paramagnetic state toward a short-range antiferromagnetic state with quasi-one-dimensional characteristics. The alterlattice opens a route to three-dimensional defect-topology engineering for non-equilibrium phases and antiferromagnetic spintronics.

## Abstract (web summary)

The authors identify an alterlattice in lanthanum–calcium titanate and report a reversible photoinduced decrease in magnetic susceptibility, with evidence for enhanced short-range antiferromagnetic correlations.

## Introduction

Photomagnetic coupling allows ultrafast low-energy spin control^1,2^, and many advances have been made in elucidating photo-enhanced ferromagnetism (FM) or photo-induced metallization^3–7^. In molecular magnets, light can reversibly switch magnetic order through driving redox processes and spin-state transitions at liquid-helium temperatures^8–10^. For transition metal oxides^3–5^, reported examples include sub-bandgap photo-induced magnetization in SrTiO_3–δ_ below 18 K, 9 THz optically enhanced FM in YTiO_3_ below 80 K, and 3 THz optically driven magnetization in SrTiO_3_ at room temperature. Thus, intense light can induce non-equilibrium charge-lattice reconstructions, enabling effective optically driven control of ferromagnetic order. Negative photomagnetic effects, namely photo-induced or enhanced antiferromagnetism (AFM), remain largely unexplored (Supplementary Table 1). AFM is attractive for spintronics owing to its zero net magnetization, sub-picosecond spin dynamics, and robustness to perturbations^11,12^. However, AFM order is highly temperature sensitive, and the alternating spin pattern is difficult to address directly with light because the optical field is nearly uniform over the magnetic unit cell^13,14^. Thus, AFM control primarily operates through indirect modulation of local magnetic interactions^14–16^, such as exchange modulation or zone-edge magnon excitation. This emphasizes the importance of designing materials that reconstruct local lattice structures and exchange pathways to strengthen optical coupling to AFM correlations.

Motivated by this challenge, we focus on a titanate system, the prototypical A-site vacancy (V_A_) perovskite La_2(1−*x*)/3_Ca_*x*_TiO_3−δ_^17^, whose average structure evolves with increasing *x* from layered long-range V_A_ ordering in La_2/3_TiO_3_ to tetragonal distortions and ultimately to disordered orthorhombic structure^18,19^. Yet in compositionally driven oxides, phase evolution may proceed through locally competing structural motifs rather than through a simple abrupt change in average symmetry^20^. Therefore, in such oxides, the relevant structural identity is not always determined only by average translational symmetry, atomic composition, and long-range crystallographic order. It may also depend on how local chemical order, lattice distortion, and domain topology are organized across multiple length scales^21,22^. Advances in atomic-scale characterization have revealed that cation displacements and vacancy ordering can form complex superstructural modulations in both real and reciprocal space^19,23^, significantly shaping macroscopic magnetoelectric and transport properties^24,25^. Conventional crystallographic mean-field theories often fail to capture such local distortions and short-range chemical order^26^. These considerations motivate the need for a framework that defines locally ordered, spatially interwoven superstructural networks and links their formation to emergent properties.

Here, we show a giant negative photomagnetic effect in the helium temperature range, which can be interpreted as arising from antiferromagnetic interactions between photoexcited magnetic species and intrinsic magnetic ions, compensating magnetic moments, and sharply reducing the macroscopic susceptibility. We trace this effect to a distinct 3D structural framework, termed the alterlattice, in which an averaged space group with statistically mixed occupancy is preserved macroscopically, while the lattice locally relaxes into nanoscale V_A_-ordered superstructure variants modulated by two orthogonal modulation vectors and alternating coherently in real space. The prefix “alter” in alterlattice therefore denotes both local alteration of the average framework by chemical ordering and alternation between nanoscale structural variants. The structural definition of the alterlattice distinguishes it from a conventional long-range ordered superlattice^27,28^, a twinned crystal^29,30^, or a phase mixture^31,32^. Its intertwined topology provides pathways for defect-mediated anisotropic carrier transport and frustrated spin interactions^6,33^, enabling illumination to rearrange local spins, suppress magnetization, and enhance short-range AFM correlations.

A perovskite oxide with a nominal composition of La_0.4_Ca_0.4_TiO_3−δ_ is used as a representative alterlattice to investigate photo-induced AFM correlations. The alterlattice framework consists of an orthorhombic *Ibmm* average host containing *Cmmm* V_A_-ordered superstructure domains with mutually orthogonal modulation vectors. We propose that coupling between local structure and short-range magnetic interactions provides a plausible explanation for the giant negative photomagnetic effect and quantify its dependence on sample thickness, laser power, temperature, and magnetic field. To test the proposed structure magnetism interpretation, we combine multimodal electron microscopy, diffraction, and low-temperature magnetometry to correlate 3D structural modulation, strain fields, and short-range chemical order with the photomagnetic response. Overall, the alterlattice provides a route for engineering 3D networks of vacancy-ordered domains that template defect states and spin-exchange pathways for optically driven AFM correlations in correlated oxides.

## Results

### The alterlattice design principles

**Fig. 1:**
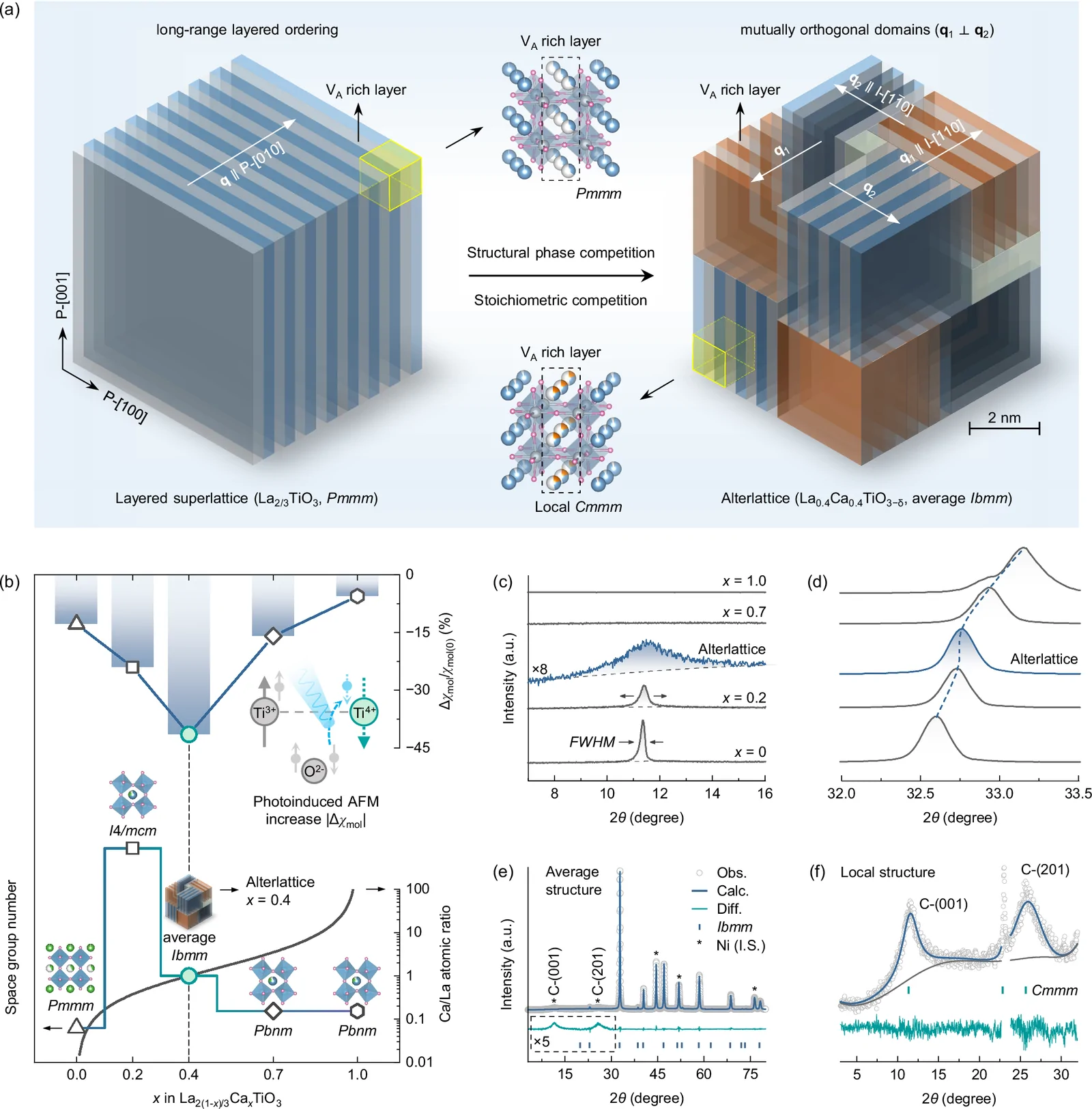
Structural paradigm and design strategy of the alterlattice driven by competitive coupling. **a** Schematic of competition between the *Ibmm* and *Pmmm* phases, fragmenting long-range A-site vacancy (V_A_) ordering into an interwoven 3D network of *Cmmm* domains termed the alterlattice. The alterlattice model represents an approximately 6 nm cubic structural region, with grayish green areas indicating A-site disorder. **b** Compositional window and negative photomagnetic response Δ*χ*_mol_/*χ*_mol(0)_ of La_2(1−*x*)/3_Ca_*x*_TiO_3_. The alterlattice appears near the orthorhombic-tetragonal phase boundary and is most pronounced near equimolar La and Ca (x = 0.4), exhibiting the largest negative Δ*χ*_mol_/*χ*_mol(0)_ at 2 K and 1 T under 473 nm illumination at 10 mW mm^−2^. The upper-right inset illustrates the proposed mechanism for photo-induced AFM correlations. Photoexcitation transfers an electron from O 2*p* into Ti 3 *d* states. The resulting Ti^3+^-like state enhances local spin compensation with nearby Ti^3+^, leading to a decrease Δ*χ*_mol_ in *χ*_mol(0)_ relative to the dark state. **c** XRD patterns of La_2(1−*x*)/3_Ca_*x*_TiO_3_ in the 2*θ* range of 7.5–16°, showing broadening of the superstructure reflection near 11.5°, indicating a progressive decrease in the correlation length of V_A_ ordering with increasing Ca substitution. **d** High-resolution XRD patterns in the 2*θ* range of 32–33.5°, showing the shift of the main reflection in La_2(1−*x*)/3_Ca_*x*_TiO_3_ toward higher angles with increasing Ca substitution. **e** Rietveld refinement of La_0.4_Ca_0.4_TiO_3–δ_ based on the average *Ibmm* structure (ICSD-245543). Discrepancies between the observed and calculated diffraction patterns near 2*θ* = 11.5° and 25.6° arise from additional local structural features associated with *Cmmm* symmetry, which are embedded within the *Ibmm* phase. Obs. in the figure denotes the observed pattern, Calc. the calculated pattern, Diff. the difference pattern, and Ni (I.S.) the reflection positions of the Ni internal standard. **f** Rietveld refinement of La_0.4_Ca_0.4_TiO_3–δ_ based on the *Cmmm* structure (ICSD-245545). Broadened superstructure reflections assigned to C-(001) and C-(201) evidence short-range order of the alterlattice. Note: X-(*hkl*) and X-[*uvw*] denote the crystallographic plane (*hkl*) and the crystallographic direction [*uvw*] referenced to the *Ibmm* (X = I) or *Cmmm* (X = C) structural setting, respectively.

Stabilizing metastable and short-range photo-induced AFM correlations requires a local structural framework that balances structural coherence with dynamic tunability, using spatial confinement to template local spin rearrangement along defined exchange pathways. In the La_2(1–*x*)/3_Ca_*x*_TiO_3_ perovskite oxide system, we identified such a framework as the alterlattice (*x* = 0.4), a V_A_ short-range ordered lattice characterized by intergrown orthogonal superstructure domains. Figure 1a schematically depicts a compositionally driven route to produce the alterlattice in nominal La_0.4_Ca_0.4_TiO_3−δ_ from a V_A_ long-range ordered La_2/3_TiO_3_ matrix. Ca substitution on the La site in the La_2/3_TiO_3_ matrix induces competing structural instabilities, frustrating the propagation of a single V_A_ long-range order. The long-range ordered structure fragments into orthorhombic domains that remain constrained by the parent symmetry and interweave into a continuous 3D network. Two 90°-rotated domains are characterized by two orthogonal superstructure modulation vectors (**q**_1_ ⊥ **q**_2_, **q**_1_ = I-½[110] and **q**_2_ = I−½[1$\bar{1}$0]), forming a double-**q** superstructure (Supplementary Fig. 1), unlike layered superlattices that can be described by a single-**q** modulation^18^. In the alterlattice, the average structure remains orthorhombic, whereas the local structure exhibits *Cmmm* distortions. In the I-[001] projection, the twin domains are orthogonal, whereas along the I-[100] projection, they are nearly parallel. Within this structural paradigm, long-range superstructure coherence is frustrated, while short-range ordered sublattices remain embedded within the host lattice.

Figure 1b summarizes the compositional evolution from La_2(1–*x*)/3_Ca_*x*_TiO_3_ to the alterlattice, with the corresponding full-range XRD patterns provided in Supplementary Fig. 2. The refined structures of the La_2/3_TiO_3_ (*x* = 0), La_0.2_Ca_0.7_TiO_3_ (*x* = 0.7), and CaTiO_3_ (*x* = 1.0) are consistent with the reported phase symmetries (Supplementary Fig. 3 and Supplementary Table 2). Near the alterlattice composition (*x* = 0.4), La_0.53_Ca_0.2_TiO_3_ (*x* = 0.2) exhibits similar but sharper superstructure reflections, consistent with long-range V_A_ ordering. With increasing Ca content (*x*), the C-(001) superstructure reflection progressively broadens (Fig. 1c), indicating that V_A_ ordering evolves from long-range to short-range order. The apparent ordering length decreases from 41.5 nm in La_2/3_TiO_3_ to 3.4 nm in the alterlattice. The strongest fundamental reflection progressively shifts toward higher 2*θ* values (Fig. 1d), consistent with gradual lattice contraction. The La_0.4_Ca_0.4_TiO_3–δ_ (*x* = 0.4) composition lies in the orthorhombic *Ibmm* region, where the C-(001) reflection reaches its maximum broadening, and the shortest structural correlation length of the superstructure domains. This near-degeneracy maximizes structural competition, and the equimolar La/Ca occupancy at the A-site enhances local instability. The alterlattice arises from coupling between structural competition and A-site chemical competition. In the equimolar La^3+^/Ca^2+^ regime (Fig. 1b), La_0.4_Ca_0.4_TiO_3–δ_ preserves macroscopic orthorhombic symmetry while developing a coherent network of interwoven *Cmmm* domains in orthogonal orientations at the microscopic scale. At 2 K and 1 T under 473 nm illumination at 10 mW mm^−2^, the negative photomagnetic response Δ*χ*_mol_/*χ*_mol(0)_ is maximized near La_0.4_Ca_0.4_TiO_3–δ_ (Fig. 1b and Supplementary Fig. 4). This response may be attributed to photoexcited electron transfer from O 2*p* to Ti^4+^ 3 *d*. The resulting Ti^3+^-like states induce local AFM interactions with neighboring Ti^3+^ centers, leading to a decrease Δ*χ*_mol_ in molar magnetic susceptibility *χ*_mol(0)_ relative to the dark state. Accordingly, this response tracks the evolution from long-range V_A_ ordering to short-range structural heterogeneity.

XRD Rietveld refinement for La_0.4_Ca_0.4_TiO_3−δ_ over the 2*θ* range of 3–80° (Fig. 1e and Supplementary Table 3) shows that the fundamental lattice reflections are well described by the average *Ibmm* orthorhombic structure, while additional broadened C-(001) and C-(201) superstructure reflections are assigned to *Cmmm* domains (Fig. 1f and Supplementary Table 4). Superstructure modeling and XRD simulations support this assignment (Supplementary Fig. 5). The *Ibmm* model accounts for the sharp fundamental reflections, whereas the *Cmmm* domains account for the broadened superstructure reflections. The superstructure reflections mainly originate from local V_A_ ordering, whereas the average crystallographic framework remains governed by the *Ibmm* host structure. The broadening of the superstructure reflections is attributed to finite and distributed correlation lengths of orthogonally oriented *Cmmm* domains with double-**q** modulations (Supplementary Fig. 6). These results indicate that such domains are coherently accommodated within the host lattice.

From the perspective of crystal chemistry, the alterlattice expresses the frustrated balance between La^3+^-driven framework stabilization and Ca^2+^-induced octahedral tilting. La–O bonds are comparatively strong (~756 kJ mol^−1^)^34^, and La^3+^ has a high ionic potential (2.21 Å^–1^), both of which favor the stability of the perovskite framework with high symmetry. In contrast, Ca–O bonds are weaker (~393 kJ mol^−1^)^35^, and Ca^2+^ has a lower ionic potential (1.49 Å^–1^). These properties favor rotations and tilts of the TiO_6_ octahedra and produce local anisotropic strain that lowers the local symmetry. At an equimolar 1:1 A-site composition, these two tendencies compete strongly, preventing the formation of a long-range ordered phase and instead producing a 3D short-range texture. Notably, although *Cmmm* superstructures are metastable and typically require reducing synthetic conditions, the present alterlattice locally stabilizes this motif under air-synthesis conditions. Moreover, differences in the diffusion kinetics of La and Ca during high-temperature sintering (1400 °C) and subsequent annealing may further amplify local inhomogeneity and favor the spontaneous formation of structural domains. Within this compositional window, the interwoven nanoscale domains provide a plausible structural environment for coupling among photogenerated carriers, defect fields, and Ti^3+^-related local spin centers, consistent with the maximized negative photomagnetic response near La_0.4_Ca_0.4_TiO_3–δ_. Under these competing thermodynamic and kinetic conditions, the alterlattice likely represents a distinct, short-range ordered pattern rather than an accidental artifact.

### Atomic-scale evidence of the alterlattice

**Fig. 2:**
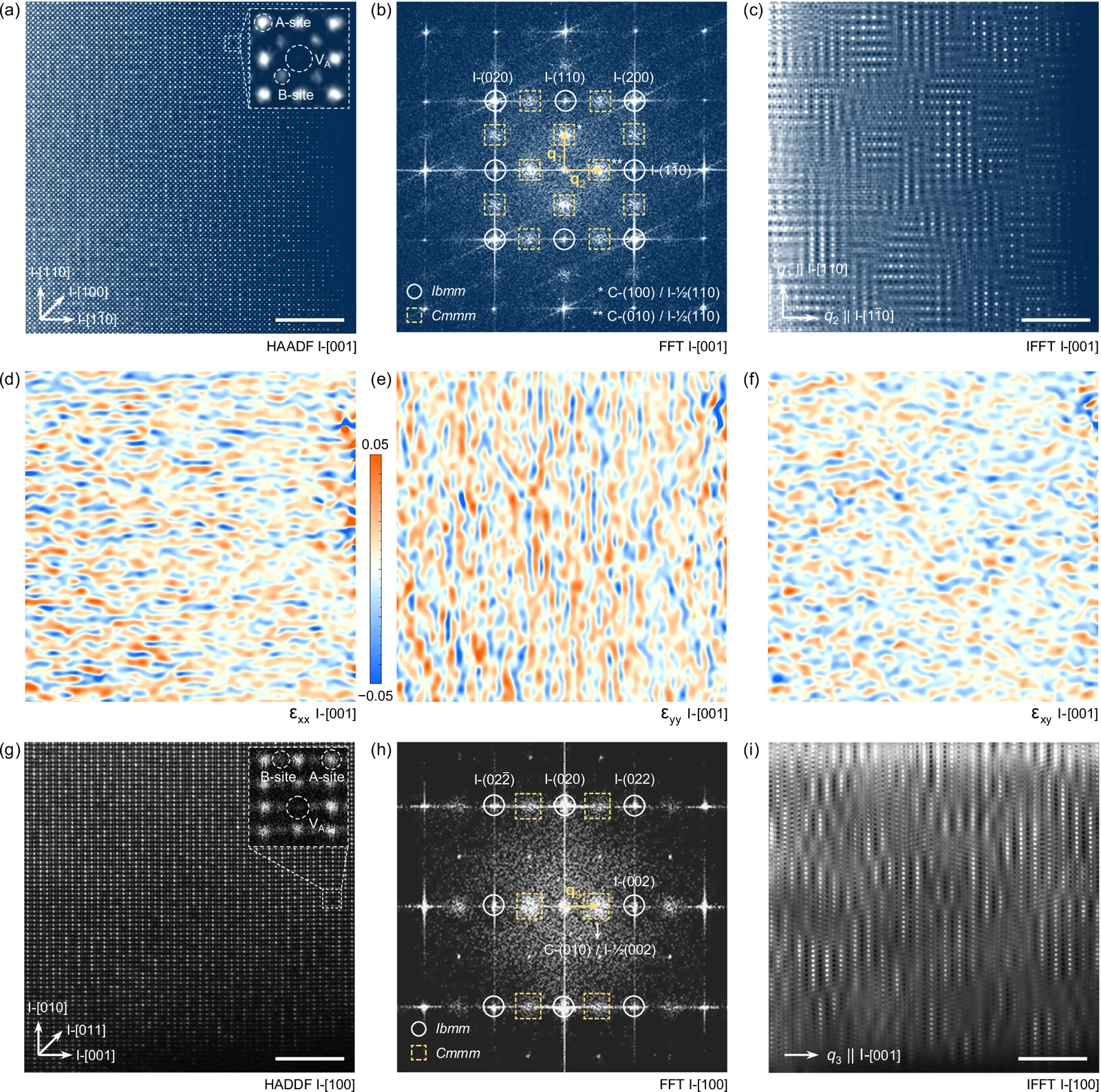
HAADF-STEM imaging and GPA mapping of the alterlattice. **a**, **g** HAADF-STEM images along the I-[001] and I-[100] zone axes (scale bar, 5 nm). The insets schematically show typical positions of A-site cations and B-site cations, and A-site vacancies within a unit cell. **b**, **h** FFT patterns along the I-[001] and I-[100] zone axes. The superstructure diffraction spots from short-range ordered domains with *Cmmm* symmetry are marked with yellow boxes, and primary reflections from long-range *Ibmm* symmetry are marked with white circles. **c**, **i** IFFT reconstructions from the yellow-boxed spots in (**b**, **h**), representing real-space lattice modulations. **d**–**f** GPA maps along the I-[001] orientation, showing in-plane normal strain components *ε*_xx_ (**d**), *ε*_yy_ (**e**), and shear strain *ε*_xy_ (**f**). The color scale ranges from –0.05 (blue, compression) to 0.05 (red, tension), indicating significant anisotropic local lattice distortions. Note: X-[*uvw*] (X = I or C) denotes the lattice direction [*uvw*] referenced to the *Ibmm* or *Cmmm* structural setting, respectively.

Atomic-scale alterlattice structures were revealed by HAADF-STEM imaging and geometric phase analysis (GPA). In the following description, I-[001] denotes the [001] zone axis of the *Ibmm* lattice, and all X-[*uvw*] (X = C or I) symbols follow the same rule, referenced to the *Cmmm* or *Ibmm* structural setting. In the I-[001] projection (Fig. 2a), HAADF-STEM images directly show locally enriched V_A_. EELS mapping revealed relatively homogeneous Ca distributions but spatial fluctuations in La concentration, suggesting preferential vacancy formation at La sites (Supplementary Fig. 7). The FFT (Fig. 2b) displays *Cmmm* superstructure spots defined by two orthogonal modulation vectors, **q**_1_ along I-½[110] and **q**_2_ along I-½[1$\bar{1}$0], as well as primary reflections from the long-range ordered framework. An IFFT image generated from these *Cmmm* reflection spots produces a real-space pattern of V_A_-ordered domains along **q**_1_ and **q**_2_ (Fig. 2c), within which the V_A_ arrangement exhibits quasi-layered ordering. Atomic column intensities oscillate by 14.6 ± 3% within these domains (Supplementary Fig. 8), consistent with vacancy modulation. GPA maps show that the in-plane normal strain components *ε*_xx_ (Fig. 2d) and *ε*_yy_ (Fig. 2e) form stripe patterns (magnitude of ±0.05), while the shear strain *ε*_xy_ (Fig. 2f) presents a 2D grid pattern, consistent with an anisotropic strain field induced by the double-**q** modulation. Annular dark-field (ADF) analysis further reveals local lattice distortions associated with TiO_6_ octahedral tilting (Supplementary Fig. 9). Independent HAADF verification of a different grain along the I-[001] zone axis (Supplementary Fig. 10), reveals identical atomic-scale phase features of the alterlattice.

The I-[100] zone axis was analyzed to assess structural anisotropy. In the HAADF-STEM image (Fig. 2g), V_A_-enriched columns were arranged in quasi-one-dimensional (1D) along I-[100]. The FFT (Fig. 2h) shows superstructure diffraction spots only along **q**_3_ (I-½[001]), indicating a predominantly quasi-1D modulation in this orientation, in sharp contrast to the double-**q** modulation observed in the I-[001] zone axis. IFFT reconstruction shows that the superstructure domains distribute as stripes running parallel to the I-[100] zone axis (Fig. 2i). Line scan analysis (Supplementary Fig. 11) reveals 1D periodic oscillations in atomic column intensity with an amplitude of 11 ± 2%, consistent with vacancy modulation. A selective IFFT further indicates that the domains extend parallel to the c axis and have a typical width of ~5 nm (Fig. 2i). GPA analysis reveals that the out-of-plane strain *ε*_yy_ is much larger than the in-plane strain *ε*_xx_, while the shear strain *ε*_xy_ appears as discontinuous stripes (Supplementary Fig. 12) instead of a 2D grid, as seen in the I-[001] case. An ADF image reveals pronounced oxygen-related lattice distortions associated with the *ε*_yy_ field (Supplementary Fig. 13). Independent HAADF verification along the I−[100] zone axis (Supplementary Fig. 14) confirms identical atomic-scale features. Therefore, the modulation dimensionality clearly differs in I-[001] and I-[100] projections.

Indeed, STEM observations along I-[001] and I-[100] zone axes show that the alterlattice is a 3D texture. Short-range superstructure domains with *Cmmm* symmetry interpenetrate along I-[110], I-[1$\bar{1}$0], and I-[100] via corner-sharing TiO_6_ octahedra and coexist coherently with a long-range orthorhombic framework. The I-[001] projection exhibits orthogonal domains (double-**q** modulation), while the I-[100] projection displays parallel domains (single-**q** modulation). Together, these observations yield a structural picture of the alterlattice as a coherent network of multi-dimensional, mutually orthogonal, short-range ordered domains embedded in an orthorhombic average lattice, with local anisotropy that depends on orientation. The observed structural modulation is thus intimately related to chemical frustration between La^3+^ and Ca^2+^ co-occupying the A-site. This twin-related, coherently intergrown domain network provides a structural constraint for the excitation of low-dimensional magnetic correlations.

### Real- and reciprocal-space verification of the alterlattice

**Fig. 3:**
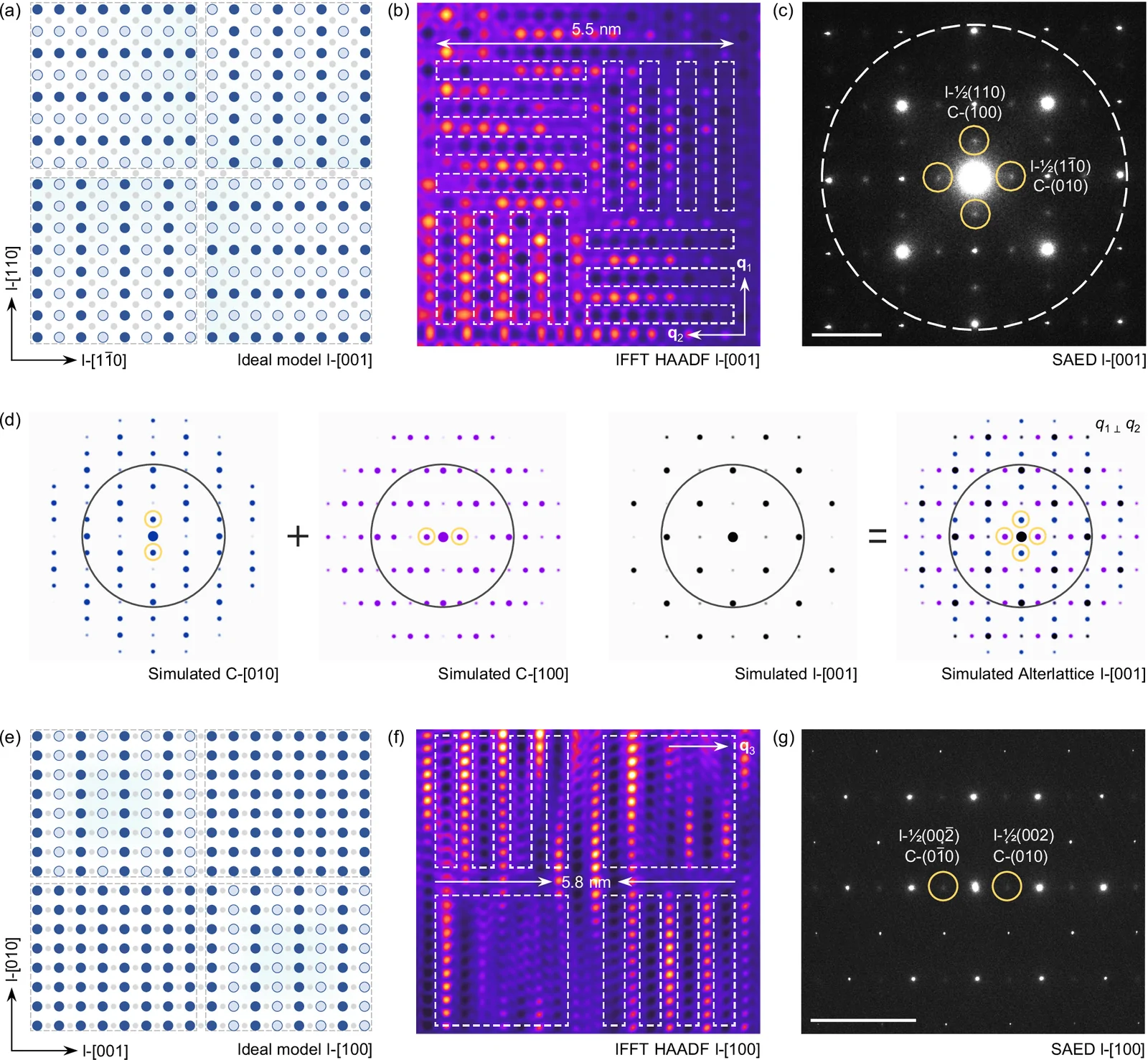
Real-space idealized model of the alterlattice validated by reciprocal-space diffraction. **a** Idealized atomic model along I−[001], showing a V_A_-enriched superstructure domain (*Cmmm*) embedded in the host lattice (*Ibmm*). Colors denote V_A_-depleted sites (dark blue), V_A_-enriched sites (light blue), and B-site cations (gray). **b** The magnified HAADF image of alterlattice along the I-[001] zone axis, showing an alterlattice motif of four intertwined superstructure domains (about 5.5 nm). **c** SAED along I-[001], in which yellow circles highlight superstructure reflections consistent with *Cmmm* symmetry. **d** Simulated I-[001] electron diffraction pattern of the alterlattice, obtained by overlaying the C-[010] and C-[100] diffraction patterns of La_0.6_Ca_0.1_TiO_3_ (space group *Cmmm*, V_A_-ordered structure with a modulation wavevector) and La_0.4_Ca_0.4_TiO_3_ (space group *Ibmm*). This mixed simulation reproduces the key features observed in the experimental I-[001] diffraction pattern of the alterlattice. **e** The alterlattice idealized atomic model along the I-[100] projection, showing a local *Cmmm* symmetry embedded within the *Ibmm* host lattice. This symmetry mismatch drives the formation of a quasi-1D modulation stripe. **f** HAADF image of alterlattice along I-[100], showing parallel superstructure domains (about 5.8 nm) of the alterlattice. **g** SAED along I-[100], in which yellow circles highlight superstructure spots consistent with quasi-1D modulation. Note: the white circle in (**c**) and the black circles in (**d**) mark the same radius in reciprocal space, corresponding to an identical 1/d scale.

Using atomic-resolution evidence along the I-[001] and I-[100] zone axes, we constructed an ideal atomic-scale model of the alterlattice and verified its multi-dimensional modulations in reciprocal space. The real-space model viewed along I-[001] projection (Fig. 3a) consists of an interlaced network of four equivalent superstructure domains related by rotational or mirror symmetry. Each domain contains layers of A-sites with periodically enriched vacancies. A representative HAADF image of a motif consisting of four ordered domains (~5.5 nm) is shown in Fig. 3b, where *Cmmm* domains with two mutually perpendicular modulation orientations alternate regularly and form the fundamental intermediate-range structural unit of the alterlattice. The simulated FFT from the I-[001] projection of the idealized alterlattice model reproduced the fundamental reflections of the orthorhombic host and additional ½-indexed superstructure reflections arising from the equivalent domains (Supplementary Fig. 15a). Two near-orthogonal modulation vectors, **q**_1_ along I-½[110] and **q**_2_ along I-½[1$\bar{1}$0], define a double-**q** modulation whose reciprocal-space fingerprint (satellite spots plus main reflections) matches the experimental SAED pattern (Fig. 3c).

At the local scale, however, anisotropic modulations and strain gradients are still measurable. SAED patterns show two sets of superstructure reflections, corresponding to the orthogonal modulation vectors (Fig. 3c). This complex diffraction pattern is incompatible with a single homogeneous structure, instead arising from the superimposition of host reflections with two modulation sets offset by 90° (Fig. 3d). Some discrepancies between the simulation and experiment are expected because FFT images are 2D projections and cannot capture full 3D dynamical scattering in SAED. Crucially, this double-**q** pattern should not be interpreted as a single superlattice: a classical single-**q** superstructure typically produces only one set of satellites. Instead, we interpret the data as arising from two twinned short-range-ordered domains (Fig. 3d), related by a 90° rotation and each exhibiting *Cmmm* symmetry, whose reciprocal space contributions overlap with the *Ibmm* host reflections. This interpretation supports the view that the alterlattice comprises interwoven short-range *Cmmm* domains embedded in a long-range *Ibmm* matrix. The alterlattice therefore exemplifies strong coupling between local ordered distortions and the macroscopic symmetry.

To develop a projection-consistent 3D structural picture of the alterlattice, we further examined the idealized model along the I-[100] projection (Fig. 3e). In this view, superstructure domains run parallel to I-[100] and are interwoven by translational symmetry. Each domain contains A-site layers with periodically ordered vacancy enrichment. A representative HAADF image of these parallel domains (≈ 5.8 nm) is presented in Fig. 3f. The simulated I-[100] FFT pattern of the idealized alterlattice model (Supplementary Fig. 15b) displays superstructure reflections distributed quasi-1D along I-[100], consistent with the modulation of the parallel domains, and this pattern matches the experimental diffraction (Fig. 3g). To further constrain this 3D picture, ROI-integrated HAADF column-intensity analysis was performed on thin regions viewed along I-[001] (Supplementary Fig. 16) and I-[100] (Supplementary Fig. 17), using Ti-column intensities as a semi-quantitative proxy for the projected B-site occupancy. The *Cmmm* domains were estimated to adopt nearly cubic geometries with characteristic lengths of ~3 nm and ~3.5 nm, respectively, close to the average apparent correlation length of ~3.4 nm estimated from XRD analysis, supporting the alterlattice model of intergrown, nearly equiaxed *Cmmm* domains within the *Ibmm* host. Because the domains are finite in size and occur as paired orientations, the local distortions in one domain are on average compensated by its orthogonal partner, preventing these distortions from coherently propagating into a macroscopic distortion. Thus, low-symmetry distortion is possible in each type of domain, but the orthogonal partner partially averages out these distortions. The average lattice constants adapt, and only chemical order persists, preserving overall orthorhombic symmetry. The experimental and simulated results (atomic models and SAED patterns) for the I-[001] and I-[100] zone axes indicate that the alterlattice originates from a 3D modulation network of mutually orthogonal short-range superstructure domains densely interwoven into a long-range orthorhombic host.

### Giant negative photomagnetism in the alterlattice

**Fig. 4:**
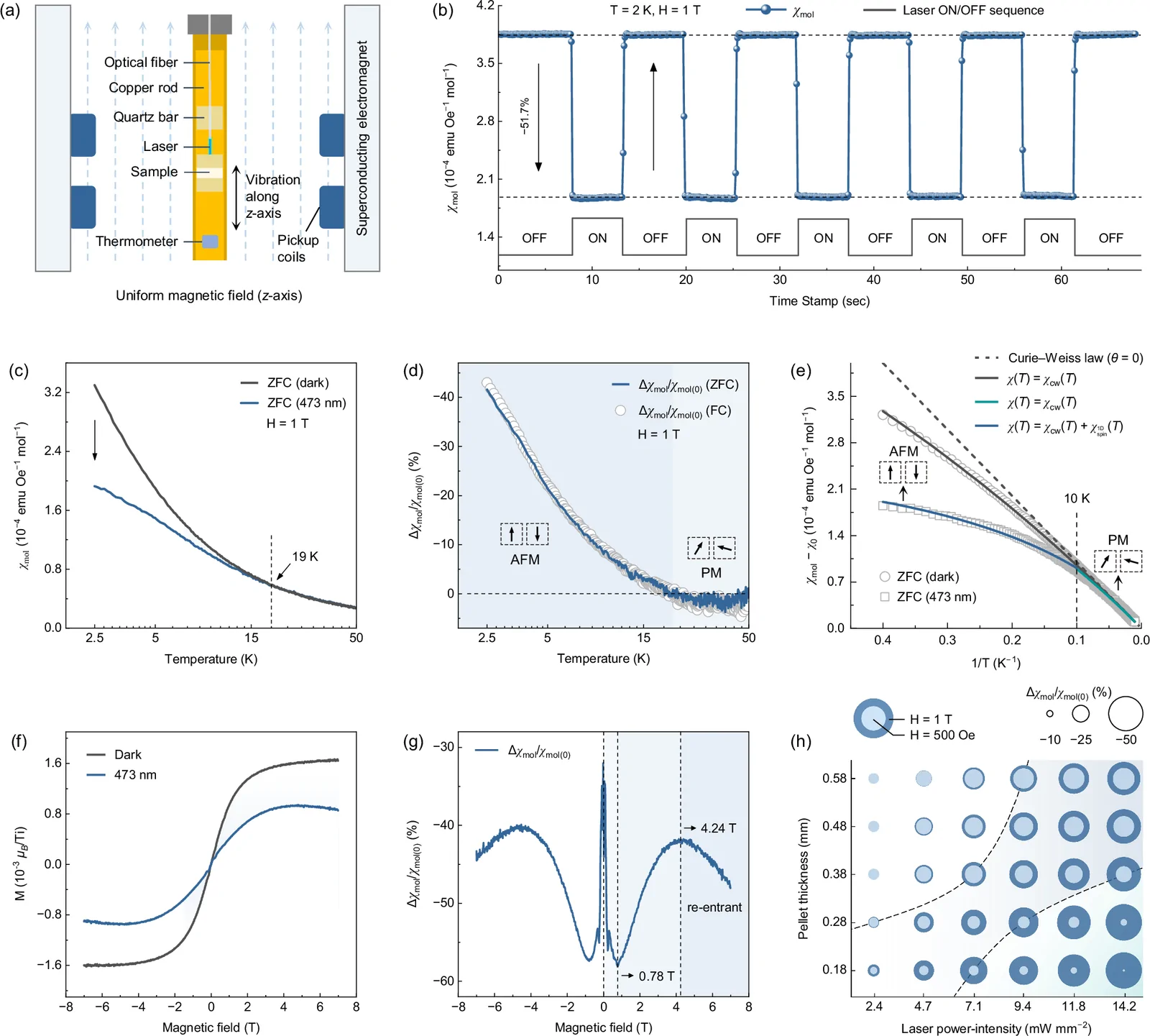
Temperature-, magnetic field-, and laser power density-dependent photomagnetism in the alterlattice. **a** Schematic of the measurement setup. A continuous 473 nm laser is incident normal to the sample surface, and a constant magnetic field is applied parallel to the sample normal, resulting in collinear light propagation and magnetic field directions. **b** Time-domain response of the molar magnetic susceptibility (*χ*_mol_) of alterlattice at 2 K and 1 T under illumination. The sample shows a reversible reduction in *χ*_mol_, and *χ*_mol_ rapidly returns to the dark baseline when the light is turned off. **c**, **d** Temperature dependence of *χ*_mol_ and its relative change Δ*χ*_mol_/*χ*_mol(0)_ at 1 T under 14.2 mW mm^−2^ illumination versus darkness, where Δ*χ*_mol_ = *χ*_mol_(light) − *χ*_mol_(dark) and *χ*_mol(0)_ is *χ*_mol_(dark). **e** ZFC *χ*_mol_ − *χ*_0_ versus 1/T measured at 1 T under illumination (14.2 mW mm^−2^) and in darkness, where *χ*_0_ is the temperature-independent susceptibility. Below 10 K, the illuminated response requires an additional quasi-1D AFM correlation term beyond the Curie–Weiss law. **f** M–H curves measured under 473 nm illumination and in darkness at 2 K. **g** Field-dependent Δ*χ*_mol_/*χ*_mol(0)_ curve, which reveals two characteristic fields at ~0.78 T and ~4.24 T, corresponding to an initial enhancement, subsequent weakening, and high-field re-entrant evolution of the photo-induced AFM correlations. **h** Size-coded scatter plot of the laser power-density (*I*) and thickness (*d*) dependence of Δ*χ*_mol_/*χ*_mol(0)_ for alterlattice pellets measured at 1 T and 500 Oe. Outer and inner circles denote measurements at 1 T and 500 Oe, respectively, while circle size scales with |Δ*χ*_mol_/*χ*_mol(0)_|. The difference in Δ*χ*_mol_/*χ*_mol(0)_ between 500 Oe and 1 T is small at *d* ≥ 0.28 mm and *I* ≤ 2.4 mW mm^−2^, but becomes pronounced at *d* ≤ 0.28 mm and *I* ≥ 7.1 mW mm^−2^, reflecting field-modulated photocarrier dynamics that alter the population of photo-induced Ti^3+^-like species.

To investigate how illumination induces magnetic interactions of the alterlattice, we performed photomagnetic measurements on a polycrystalline pellet of alterlattice (3 mm in diameter, with thickness *d* = 0.18–0.58 mm). Measurements were carried out on a PPMS equipped with a vibrating-sample magnetometer (VSM). The experimental geometry is shown in Fig. 4a, a continuous 473 nm laser illuminated the pellet at normal incidence (Fig. 4b–g were obtained from a pellet with *d* = 0.48 mm), while a static magnetic field was applied along the sample normal (collinear with the illumination). For clarity, zero-field cooling (ZFC) and field cooling (FC) denote the magnetic-field history during cooling, whereas dark/light labels the illumination state during measurement, thus temperature-sweep curves are referenced as ZFC-dark, ZFC-light, FC-dark, and FC-light. In the dark state, the sample exhibited paramagnetic behavior from 2 K to 300 K with a measurable magnetic signal, attributable to local Ti^3+^. The ZFC-dark and FC-dark curves remained nearly identical over the measured temperature range (Supplementary Fig. 18). Within the alterlattice, spatially isolated Ti^3+^ (3*d*^1^, *S* = 1/2) ions are incorporated into a Ti^4+^ (3*d*^0^) host matrix with local V_A_ ordering. Quantitative EELS analysis from two independent regions reveals sub-nanometer V_A_ accumulation that perturbs local oxygen coordination (Supplementary Figs. 19 and 20), showing spatially correlated V_A_ enrichment, enhanced Ti^3+^ character, and oxygen deficiency. Complementary Ti 2*p* XPS further supports the presence of a minor surface Ti^3+^ component (Supplementary Fig. 21). We attribute this local valence reconstruction to the electron-donor effect of nearby oxygen vacancies. The vacancy electrons partially reduce adjacent Ti^4+^ to magnetically active Ti^3+^ (3*d*^1^). Although magnetic super-exchange requires connected Ti^3+^-O-Ti^3+^ pathways, the Ti^3+^ ions are too sparse to form a percolating network in alterlattice under dark conditions.

The time-dependent magnetic *χ*_mol_ was measured during periodic light on/off switching, allowing direct visualization of the photomagnetic response amplitude, reversibility, and response timescale. Under 473 nm illumination (14.2 mW mm^−2^) at 2 K and 1 T, the molar susceptibility *χ*_mol_ drops to ~48% of its initial (dark) value within 1 s (Δ*χ*_mol_/*χ*_mol(0)_ ≈ –51.7%), indicating a large reversible negative photomagnetic effect, where Δ*χ*_mol_ = *χ*_mol(light)_ − *χ*_mol(dark)_ and *χ*_mol(0)_ = *χ*_mol(dark)_ (Fig. 4b). We term this large, reversible decrease in *χ*_mol_ as a negative photomagnetic effect. The photomagnetic effect of the alterlattice is highly repeatable (>98% recovery over five on/off cycles) with recovery occurring on the order of seconds. This timescale is much faster than thermally driven magnetic phase changes. Although La_0.4_Ca_0.4_TiO_3−δ_ has an optical bandgap of ~3.2 eV (Supplementary Fig. 22), sub-bandgap 473 nm (~2.62 eV) illumination still induces a pronounced photomagnetic response, indicating that direct band-to-band excitation is not the dominant mechanism. Wavelength-dependent measurements further support this interpretation (Supplementary Figs. 23 and 24), with the strongest response observed under 473 nm rather than under 405 nm (~3.06 eV), despite the latter being closer to the band edge. These results suggest that Ti^3+^/V_O_ related intragap states participate in sub-bandgap absorption and photocarrier redistribution, thereby inducing AFM correlations and reducing *χ*_mol_ under illumination.

Temperature-dependent *χ*_mol_ revealed thermally activated features of the photomagnetism in the alterlattice (Fig. 4c). The ZFC-light and ZFC-dark curves diverge near 19 K and show different increases upon further cooling. In the temperature range 2.5–19 K, *χ*_mol_ in ZFC-light is consistently lower than that in ZFC-dark. The FC-light curve exhibits a slight divergence from the corresponding ZFC-light curve near 15 K (Supplementary Fig. 25a), suggesting the emergence of weak and short-range magnetic correlations. The relative photo-induced change Δ*χ*_mol_/*χ*_mol(0)_ increases below ~19 K (Fig. 4d), indicating strengthened magnetic interactions at low temperatures. As shown in Fig. 4e, an analysis of *χ*_mol_ − χ_0_ versus 1/T was performed for the ZFC-dark and ZFC-light curves, where *χ*_0_ = 7.7 ×  10^−6^ emu mol^−1^ Oe^−1^ is the temperature-independent *χ*. In the dark state, the relationship between *χ*_mol_ and 1/T was consistent with Curie–Weiss behavior, with a fitted Curie constant of C = 0.00101 emu K mol^−1^ Oe^−1^ and a Weiss temperature of *θ* = −0.87 K. Under illumination, the high-temperature region remained roughly linear, but below 10 K this linearity broke down and the slope decreases, showing the presence of additional antiferromagnetic correlations at low temperature. Analysis using a thermally activated low-dimensional AFM correlation model (Supplementary Fig. 26)^36,37^. This analysis suggests that the photo-induced magnetic response is more consistent with quasi-1D AFM correlations (Supplementary Table 5), with a small spin-excitation gap of *Δ* = 1.33 K, consistent with short-range correlated magnetic excitations. At higher temperatures (50–100 K), thermal fluctuations suppress significant photo-induced AFM correlations (Supplementary Fig. 27). Collectively, the photo-induced *χ*_mol_ reduction, ZFC/FC-light splitting, deviation of ZFC-light from Curie–Weiss behavior, and consistency with a low-dimensional AFM correlation model indicate a crossover from a paramagnetic regime to one with enhanced AFM correlations or a precursor of short-range AFM orders, consistent with the structural modulation length scale observed in the alterlattice.

Field-dependent magnetization-versus-field (M–H) measurements were performed at 2 K under illumination and in the dark to clarify how the magnetic field modulates photomagnetism and governs the competition between field spin polarization and photo-induced AFM correlations. The M–H curves show that illumination reduces the magnetization over the entire 0–7 T field range relative to the dark state (Fig. 4f), together with a non-saturating suppression of magnetization above ~4 T. As H increases to 0.78 T (Fig. 4g), |Δ*χ*_mol_/*χ*_mol(0)_| increases due to field-suppressed spin fluctuations. Above 0.78 T, additional field-induced spin polarization weakens the light-enhanced photo-induced AFM correlations, leading to a decrease in |Δ*χ*_mol_/*χ*_mol(0)_|. When H exceeds 4.24 T, the spin background approaches saturation, and further increases in H cause |Δ*χ*_mol_/*χ*_mol(0)_| to increase again. This behavior suggests a re-entrance of AFM correlations, possibly associated with anomalous magnetoelectronic transport under high magnetic fields. To reduce the suppression and bias of spontaneous correlations by the large applied field, additional measurements were carried out at 500 Oe (Supplementary Fig. 28a). At this low field, the ZFC-light vs ZFC-dark separation is much reduced, and the ZFC–FC splitting under illumination becomes weak (Supplementary Fig. 25b), indicating that photo-induced low-temperature magnetic correlations persist but are weak in magnitude. This behavior is consistent with the usual interpretation of ZFC/FC splitting as an indicator of low-temperature glassy or short-range order. Consistent with the behavior at 1 T (Fig. 4d), the relative Δχ_mol_/χ_mol(0)_ at 500 Oe indicates a crossover from high-temperature paramagnetism to enhanced antiferromagnetic correlations at low temperature (Supplementary Fig. 28b), passing through an intermediate regime characterized by emerging spin correlations. However, the onset temperature for enhanced antiferromagnetic correlations shifts downward to ~11 K. For the ZFC-light at 500 Oe, the *χ*_mol_ versus 1/T remains nearly linear at 50–100 K, but the deviation from Curie–Weiss behavior shifts to lower temperatures and becomes weaker relative to 1 T (Supplementary Fig. 29). A thickness-dependent behavior was also observed (Supplementary Fig. 30a). When *d* > 0.48 mm, the extrapolated ZFC Δ*χ*_mol_/*χ*_mol(0)_ at 1 T begins to decrease, and a similar thickness-dependent trend appears for the measurements at 500 Oe (Supplementary Fig. 30b).

The dependence of the photomagnetic response on field strength, laser power-density (*I*), and sample thickness (*d*) was also evaluated to probe its carrier-mediated mechanism. At 2 K and 1 T, increasing *I* caused a rapid drop in *χ*_mol_ (Supplementary Fig. 31a). At 500 Oe, a clear power-density threshold was observed, with *χ*_mol_ dropping rapidly at *I* = 2.36 mW mm^−2^ and saturating at *I* = 14.2 mW mm^−2^ (Supplementary Fig. 31b). Laser power-density and sample thickness-dependent photomagnetic response mapping showed that |Δ*χ*_mol_/*χ*_mol(0)_| generally increased with power-density, and above 6 mW mm^−2^, the difference between 1 T and 500 Oe field responses grew markedly. This field-dependent enhancement was more pronounced for the thin pellet *d* = 0.28 mm (Fig. 4h). Notably, at 500 Oe, for *d* < 0.38 mm and *I* > 9 mW mm^−2^, the photomagnetic response inverted from enhancement to suppression. We attribute the suppressed photomagnetic response in thin pellets under high excitation at 500 Oe to reduced light penetration depth and enhanced photocarrier recombination. The dependence on pellet thickness, laser power density, and applied magnetic field supports a carrier-mediated photomagnetic mechanism. These observations argue against a purely thermal mechanism or direct photon-spin coupling, and instead support a defect-mediated, photocarrier-driven pathway.

## Discussion

Overall, the results support a delicate scale matching between the short-range photomagnetism and the short-range structure in the alterlattice, specifically between the photo-induced low-dimensional AFM correlations and the V_A_-ordered superstructure domains. This correspondence suggests that the local structure constrains the development of the short-range magnetic state. Such coupling between local symmetry, strain fields, and short-range correlated states highlights the important role of mesoscale structural organization in stabilizing emergent non-equilibrium phases.

At the atomic scale in the alterlattice, the intrinsic isolated Ti^3+^ spins may act as local magnetic “seeds” that nucleate emergent magnetic states under external perturbations, making the system highly sensitive to external fields. Ti^3+^ has a 3*d*^1^ configuration and carries an *S* = 1/2 local moment, whereas Ti^4+^ has a nonmagnetic 3*d*^0^ configuration. Therefore, super-exchange pathways are not intrinsically established within Ti^4+^–O–Ti^3+^ linkages under equilibrium conditions. Under 473 nm illumination in an applied magnetic field, defect-state-related sub-gap excitation near Ti^3+^ sites can generate localized carriers and induce Ti^3+^-like species at least on neighboring Ti^4+^ sites (Supplementary Fig. 32a), increase the local Ti^3+^ population, and reconstruct local Ti^3+^–O–Ti^3+^ exchange pathways (Supplementary Fig. 32b). Simultaneously, *χ*_mol_ decreases sharply as the magnetic configuration rearranges, consistent with the giant negative photomagnetic effect observed in alterlattice. This decrease can be understood as partial compensation of local moments when photo-induced Ti^3+^-like small polarons increase the probability of local Ti^3+^–O–Ti^3+^ exchange units. Consistently, fitting of the illuminated susceptibility requires an additional short-range quasi-1D AFM term beyond a simple Curie–Weiss paramagnetic state. For the AFM term, the short and distributed correlation lengths make a well-defined magnetic phase transition difficult to resolve, but they also allow the magnetic state to be switched rapidly under external perturbations.

At the sub-mesoscopic scale of the orthogonal domains in the alterlattice, the structure becomes anisotropic, and the photo-induced carrier and magnetic reorganizations are therefore orientation-dependent. Along I-[001], the B-site sublattice within the *Cmmm* domains exhibits a more symmetric local arrangement than the average *Ibmm* lattice and contains Ti–O–Ti linkages closer to 180°, which may strengthen AFM super-exchange. Under the orientational constraint imposed by the intergrown domains, the associated crystal-field anisotropy and coherent domain boundaries may stabilize an orthogonal AFM texture between adjacent domains (Supplementary Fig. 33). Moreover, mutual perturbations between differently oriented AFM domains may promote relaxation toward a lower-energy non-coplanar spin texture, with non-coplanar AFM configurations representing a plausible sub-mesoscopic stabilized state. In the idealized alterlattice, with uniformly sized domains arranged into a strictly continuous orthogonal network, illumination may drive nearly complete compensation of the macroscopic magnetic moment, producing a local AFM state that follows the domain orientational and arrangement texture.

The present measurements probe the bulk-averaged magnetic response of polycrystalline pellets. The pellets exhibit a terrace-like microstructure associated with oxygen deficiency (Supplementary Figs. 34 and 35)^38,39^, consisting of grains with a log-normal size distribution (*D*_50_ ≈ 11.6 μm). Variations in the grain orientation of the pellet may give rise to different photomagnetic behaviors. The dependence on pellet thickness is non-monotonic, the effect is reduced for very thin (carrier recombination) or very thick (insufficient penetration) pellets, and is strongest when thickness matches the carrier diffusion length (Supplementary Fig. 36). These trends suggest that, given the limited optical penetration depth, carrier recombination and delocalization more readily suppress the volume fraction of the photo-induced correlated phase at low field. Similarly, power-density dependence shows an optimal window (Supplementary Fig. 37). At low power-density, the sparse photoexcited Ti^3+^ population prevents the formation of percolating Ti^3+^-O-Ti^3+^ pathways, resulting in isolated spins. At high power density, recombination becomes dominant, which limits further response. An applied magnetic field enhances the photomagnetic response by extending carrier lifetimes and increasing effective diffusion lengths through field modulation of carrier transport (Supplementary Fig. 38). The consistency of the effect with sub-bandgap photon energies, the dependence on thickness matching carrier diffusion, sensitivity to applied field, and the fast reversible dynamics all indicate a nonthermal, non-equilibrium carrier-mediated process.

From a materials design perspective, the alterlattice offers a generalizable strategy for coupling chemistry, lattice strain, and spin on a 3D network through defect-mediated local structural modulation. Equimolar substitution of A-site cations with different valences and ionic radii can drive structural-phase competition, frustrate long-range order, and produce short-range textured domains. More broadly, the alterlattice concept can be extended to A-site-vacancy stabilized perovskites [V_A(x)_R_((1–x)/2)_A_((1–x)/2)_]MO_3–δ_, where M is a transition-metal cation, R is a rare-earth cation, A is an alkaline-earth cation, V_A_ denotes an A-site vacancy, and x is the vacancy fraction. Established here for titanates, vacancy ordering and double-**q** competition likely stem from generic defect-structure coupling in this material class, suggesting broader relevance to related families with representative compositions such as La_0.4_Sr_0.4_TiO_3_, Nd_0.4_Sr_0.4_MnO_3_, and La_0.4_Sr_0.4_NiO_3_. In this context, the alterlattice acts as a structured amplifier of photo-induced magnetism by combining short-range vacancy ordering, defect-assisted sub-gap excitation, and local exchange reconstruction. For potential device applications, each alterlattice grain may function as a spin-logic unit constructed from interwoven structural domains. This architecture enables data encoding in optically reversible net magnetic moments, which are programmed via controlling the domain arrangement.

In conclusion, we establish the alterlattice as a structural route for generating optically tunable short-range magnetism in an A-site deficient perovskite oxide. The term alterlattice describes a structurally coherent lattice state in which the average framework is locally altered by superstructure domains, making this lattice motif distinct from a conventional long-range superlattice, a twin crystal, a simple phase mixture, or phase separation. The alterlattice is defined by an intergrown nanoscale domain network arising from a pair of orthogonal superstructure modulation vectors, which produces a grid-like local strain field. Under sub-bandgap illumination, this constrained strain environment promotes low-dimensional antiferromagnetic excitations with an extremely small gap energy, indicating that the photo-induced state is governed primarily by short-range spin correlations confined within the structural domains. These findings point to local structure and strain-network engineering in A-site-deficient oxides as an effective strategy for realizing light-controlled magnetic responses. Future studies combining techniques such as three-dimensional electron tomography, NV-center quantum magnetometry, magnetic pair distribution function analysis, and first-principles spin-exchange simulations may help further resolve the 3D structural topology of the alterlattice, as well as the dynamical evolution and microscopic exchange pathways of the photo-induced magnetic phase.

## Methods

### Sample preparation

The nominal composition La_0.4_Ca_0.4_TiO_3–δ_ (alterlattice) was synthesized using the solid-state reaction route. High-purity La_2_O_3_ (99.99%, Aladdin), CaCO_3_ (99.99%, Aladdin), and TiO_2_ (99.8%, Aladdin) were used as raw materials. Before weighing, La_2_O_3_ and TiO_2_ were pre-calcined at 950 °C for 3 h in a muffle furnace to remove adsorbed water, while CaCO_3_ was dehydrated at 250 °C for 6 h in a vacuum oven. Then, La_2_O_3_, CaCO_3_, and TiO_2_ powders were weighed according to the nominal La_0.4_Ca_0.4_TiO_3–δ_ stoichiometry, suspended in anhydrous ethanol, and ultrasonically dispersed for 12 h to promote precursor fragmentation and homogeneous mixing. The resulting suspension was vacuum-dried at 80 °C to obtain fine and compositionally uniform precursor powders for subsequent sintering.

A stepwise sintering process was employed to optimize crystal growth. Powders were first pre-calcined in air at 1000 °C for 12 h (heating/cooling rate 5 °C min^−1^). The product was ground into a fine powder in an agate mortar and pressed into green pellets (*φ* = 10 mm) under 10 MPa uniaxial pressure, followed by main sintering in air at 1400 °C for 12 h (heating/cooling rate 5 °C min^−1^). The sintering cycle was repeated twice to reduce lattice defects. Cold isostatic pressing (*φ* = 3 mm, 1 MPa) was applied to samples for magnetic and optical tests, and then sintering at 1400 °C for 10 h (heating/cooling rate: 5 °C min^−1^) was performed to obtain dense polycrystalline pellets. For a La_0.4_Ca_0.4_TiO_3–δ_ ceramic pellet with a diameter of 3 mm and a thickness of 0.48 mm, Curie–Weiss fitting of the susceptibility from 2 to 100 K gives an estimated Ti^3+^ fraction of 2.7 × 10^−3^ (Supplementary Fig. 39), corresponding by charge neutrality to *δ* ≈ 1.4 × 10^−3^ and an oxygen stoichiometry of La_0.4_Ca_0.4_TiO_2.9986_.

A series of La_2(1−*x*)/3_Ca_*x*_TiO_3_ samples with *x* = 0.2, 0.7, and 1.0 were prepared using the same synthesis protocol, whereas La_2/3_TiO_3_ (*x* = 0) was synthesized by a solid-state reaction at 1300 °C for 10 h in flowing 5% H_2_/Ar. Similar to the synthesis procedure described above, the grinding and sintering process was repeated three times to improve phase purity and homogeneity.

### Structural characterization

The crystallographic structure was characterized by XRD (Rigaku Miniflex) using Cu Kα radiation (*λ* = 1.5418 Å). Rietveld refinement was done through GSAS II for diffraction data processing. The field emission scanning electron microscopy (MERLIN VP Compact, ZEISS) was used to examine the surface morphology of the sample. The UV–vis spectra were recorded on the UV-1900i spectrometer.

STEM specimens were prepared by gentle grinding of the ceramic sample, dispersion in anhydrous ethanol, ultrasonic treatment, and drop casting onto carbon-supported copper grids. An aberration-corrected JEOL Cs-corrected electron microscope operated at 200 kV was used for HAADF/ADF-STEM imaging, SAED measurements, and EELS mapping. SAED patterns were acquired in TEM diffraction mode with a nominal camera length of 600 mm and a calibrated camera constant of ~150.48 Å mm. For HAADF/ADF-STEM imaging, the camera length was 60 mm, the probe convergence semi-angle was 20.3 mrad with a probe current of 40 pA and a probe size of 0.08 nm. The detector collection angles were 40–160 mrad for ADF and 90–370 mrad for HAADF imaging. The HAADF-STEM images were further treated and analyzed by digital filtering (FFT and IFFT) through Gatan Digital Micrograph.

### Photomagnetic measurements

Magnetic measurements were performed on a 7 T Physical Property Measurement System (DynaCool, Quantum Design) with an option for a Vibrating Sample Magnetometer (VSM) and a multi-field coupling measurement module for in situ optical excitation. The DC magnetic field was applied along the *Z* axis of the sample, normal to the pellet surface. The temperature was controlled by the PPMS cryostat in closed-cycle mode. Continuous wave 405, 473, and 532 nm diode lasers were fiber coupled to the sample space using a fiber optic sample holder, delivering nominal power densities of 0 to 14.2 mW mm^−2^ to a 3 mm diameter spot roughly centered on the sample surface. All quoted power-density values correspond to the output power at the laser port.

Thermal, magnetic field, and optical effects are separated by a number of magnetization protocols performed in the dark. In the present ZFC (light) measurements, the sample is first cooled from 300 K to 2 K in zero field and in darkness; after thermal equilibrium is reached at 2 K, the field is set, the laser is switched on, and magnetization data are taken upon warming at constant field and illumination. The ZFC (dark) curve is obtained with exactly the same temperature and field sequence; however, the laser is switched off throughout the sequence. In the FC (light) measurement, the sample is cooled from 300 K under a field and continuous 473 nm illumination to 2 K; magnetization is then measured upon warming in the same field and under the same illumination. The FC (dark) reference is obtained when the same field-cooling sequence is done in the dark. All temperature-dependent measurements were performed point by point at a warming rate of 0.5 K min^−1^ to ensure good sample thermal equilibration.

Isothermal M–H loops under illumination were measured after dark cooling in zero field from 300 K to the target temperature. After temperature stabilization, the 473 nm laser was switched on and kept on while measuring the magnetization variation versus applied magnetic field, mainly between −7 T and +7 T. Dark M–H loops were measured by identical temperature and magnetic field sequences without turning on the laser. Light pulses were also applied at constant temperature and applied magnetic field to determine the dynamic photomagnetic response. After cooling to the desired temperature in the dark, the field is applied and stabilized. Afterward, the magnetization is monitored while the laser at 473 nm is periodically switched on and off like a step. The measurement was continued under illumination or in the dark until quasi-steady magnetization values were reached.

For consistency in data processing and comparison, the temperature points in the M–T data were linearly interpolated to fixed values with one decimal place, and the magnetic field points in the M–H data were linearly interpolated to fixed field intervals. Unless otherwise stated, all magnetization data were background-subtracted using sample holder measurements performed at the same temperature, magnetic field, and illumination conditions.

### Geometric phase analysis

Geometric phase analysis (GPA) was performed on HAADF-STEM images using a commercial Digital Micrograph plugin (DM 1.8.3 suite, HREM Research, Inc.). Displacement fields were retrieved by Fourier filtering. Two non-collinear Bragg vectors were selected and filtered from the image power spectrum, enabling visualization of the strain distribution as color-coded contour maps. For comparison, all strain maps shown in this paper were plotted using the same scale from −5% to +5%.

### Electron-diffraction simulations

The electron-diffraction simulations were carried out using SingleCrystal (CrystalMaker Software Ltd., Oxford, UK). The crystallographic structures obtained from ICSD were imported into SingleCrystal in CIF format. The simulations were performed in TEM diffraction mode using an accelerating voltage of 200 kV, an electron wavelength of 0.0251 Å, a nominal camera length of 600 mm, a camera constant of 150.48 Å mm, a convergence angle of 0.05°, and a sample thickness of 100 Å. All simulated patterns used for comparison or overlay were displayed on the same reciprocal space scale.

## Supplementary information

Supplementary Information

Transparent Peer Review file

## Source data

Source data

## Data Availability

The data generated in this study are provided in the Source Data file. All data supporting the findings of this study are available within the paper and its Supplementary Information or from the corresponding author upon request. Source data are provided with this paper.
