# Supplementary material for: Giant negative photomagnetism in an alterlattice
Source: Nat Commun. 2026 Aug 7;17:9522. doi: 10.1038/s41467-026-76523-2 (PMC13547212; doi:10.1038/s41467-026-76523-2)
Supplement: Supplementary file 1 [file 41467_2026_76523_MOESM1_ESM.pdf]

Supplementary Information

Giant Negative Photomagnetism in an Alterlattice

Junhong Zhou[1], Xu Han[1], Haibin Huang[1], Taotao Huang[1], Yaowen Wang[1], Brian F. Woodfield[2], Liping Li[1,*], Guangshe Li[1,*]

[1] State Key Laboratory of Inorganic Synthesis and Preparative Chemistry, College of Chemistry, Jilin University, Changchun 130012, P.R. China

[2] Department of Chemistry and Biochemistry, Brigham Young University, Provo, UT 84602, United States

[*] Correspondence to: lipingli@jlu.edu.cn; guangshe@jlu.edu.cn

Supplementary Figures and Tables

Supplementary Table 1 4
Supplementary Figure 1 5
Supplementary Figure 2 7
Supplementary Figure 3 8
Supplementary Table 2 9
Supplementary Figure 4 10
Supplementary Table 3 11
Supplementary Table 4 11
Supplementary Figure 5 12
Supplementary Figure 6 14
Supplementary Figure 7 16
Supplementary Figure 8 17
Supplementary Figure 9 18
Supplementary Figure 10 19
Supplementary Figure 11 20
Supplementary Figure 12 21
Supplementary Figure 13 22
Supplementary Figure 14 23
Supplementary Figure 15 24
Supplementary Figure 16 25
Supplementary Figure 17 28
Supplementary Figure 18 30
Supplementary Figure 19 31
Supplementary Figure 20 32
Supplementary Figure 21 33
Supplementary Figure 22 34

Supplementary Figure 23 35
Supplementary Figure 24 36
Supplementary Figure 25 37
Supplementary Figure 26 38
Supplementary Table 5 39
Supplementary Figure 27 40
Supplementary Figure 28 41
Supplementary Figure 29 42
Supplementary Figure 30 43
Supplementary Figure 31 44
Supplementary Figure 32 45
Supplementary Figure 33 46
Supplementary Figure 34 47
Supplementary Figure 35 48
Supplementary Figure 36 49
Supplementary Figure 37 50
Supplementary Figure 38 51
Supplementary Figure 39 52
References 53

Supplementary Table 1 | Photomagnetic response of the alterlattice compared with representative materials. Comparison of photo-induced magnetic responses in representative perovskites, titanates, and related photomagnetic materials, including material type, photomagnetic-response characteristics, and corresponding temperature ranges. Compared with previously reported systems, the alterlattice exhibits a rare and pronounced negative photomagnetic response in a bulk transition-metal oxide, characterized by a rapid light-induced decrease in macroscopic magnetic susceptibility.

**Materials**	**Material Type**	**Photomagnetic Response**	**Temperature Range**	**Refs.**
$La_{0.4}Ca_{0.4}TiO_{3-\delta}$	Alterlattice (cation- and anion-deficient titanate)	473 nm induced AFM interactions	< 19 K	This work
$SrTiO_{3-\delta}$	O-deficient titanate	400−500 nm induced persistent magnetization	< 18 K	[1]
$YTiO_3$	Mott titanate	9 THz enhanced FM	< 80 K	[2]
$Ni/BaTiO_3$	Heterostructures	532 nm enhanced magnetization	< 100 K	[3]
$La_{1.2}Sr_{1.8}Mn_2O_7$	Bilayer manganite	532 nm enhanced magnetization	30–200 K	[4]
$Gd_{0.55}Sr_{0.45}MnO_3$	Charge-ordered manganite	800 nm induced FM switch	< 44 K	[5]
$La_{1/2}Sr_{1/2}MnO_{3-\delta}$	Manganite thin film	500 nm enhanced magnetization	< 50 K	[6]
$Zn_{0.5}Co_{0.5}Fe_2O_4$	Spinel ferrite	532 nm induced magnetoresistance effects	< 350 K	[7]

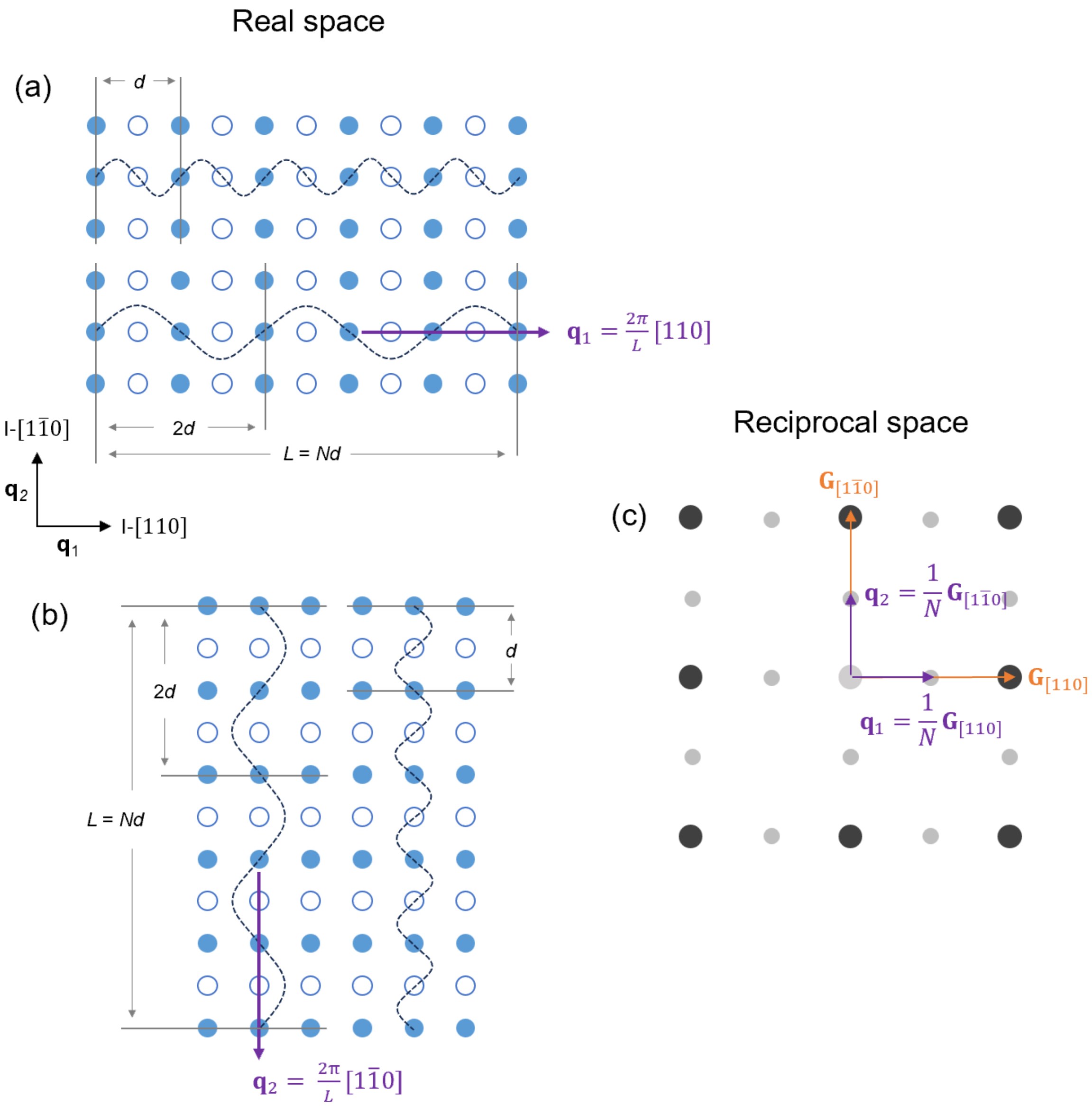

Supplementary Figure 1 | Real-space and reciprocal-space schematics of the double-q superstructure modulation vectors in the alterlattice. (a,b) Local superstructure modulations along [110] and $[1\bar{1}0]$ orthogonal directions. In real space, A-site cation/vacancy-related ordering and local lattice distortions introduce an additional twofold periodic modulation on the perovskite lattice. The modulation periods extend along [110] and $[1\bar{1}0]$. The characteristic length is $L = Nd$, where d is the parent-lattice spacing and N is the period multiplication factor. Here, $N = 2$. The corresponding modulation vectors are $\mathbf{q}_1 = \mathbf{G}_{[110]}/N$ and $\mathbf{q}_2 = \mathbf{G}_{[1\bar{1}0]}/N$, where $\mathbf{G}_{[110]}$ and $\mathbf{G}_{[1\text{-}10]}$ are the parent-lattice reciprocal vectors along the corresponding directions. Equivalently, $|\mathbf{q}| = 2\pi/L$. Thus, q describes the additional periodicity of the superstructure relative to the parent lattice. The filled and open blue circles indicate periodic variations in local structural environment or site occupancy. The dashed lines

indicate modulation waves caused by local structural distortions. **(c)** Reciprocal-space schematic corresponding to the real-space modulation. The black spots represent the fundamental reflections $\mathbf{G}_{[110]}$ and $\mathbf{G}_{[1\bar{1}0]}$. The purple arrows represent $\mathbf{q}_1$ and $\mathbf{q}_2$ generated by the superstructure. For a commensurate superstructure, $\mathbf{q} = \mathbf{G}/N$. Therefore, the superstructure reflections appear at half-integer positions between main reflections, with displacement directions consistent with **q**. The two orthogonal modulation vectors indicate that this is not a simple single-**q** modulation, but a double-**q** superstructure with a two-dimensionally coupled modulation network. The double-**q** modulation is a key reciprocal-space signature of the local ordered state in the alterlattice.

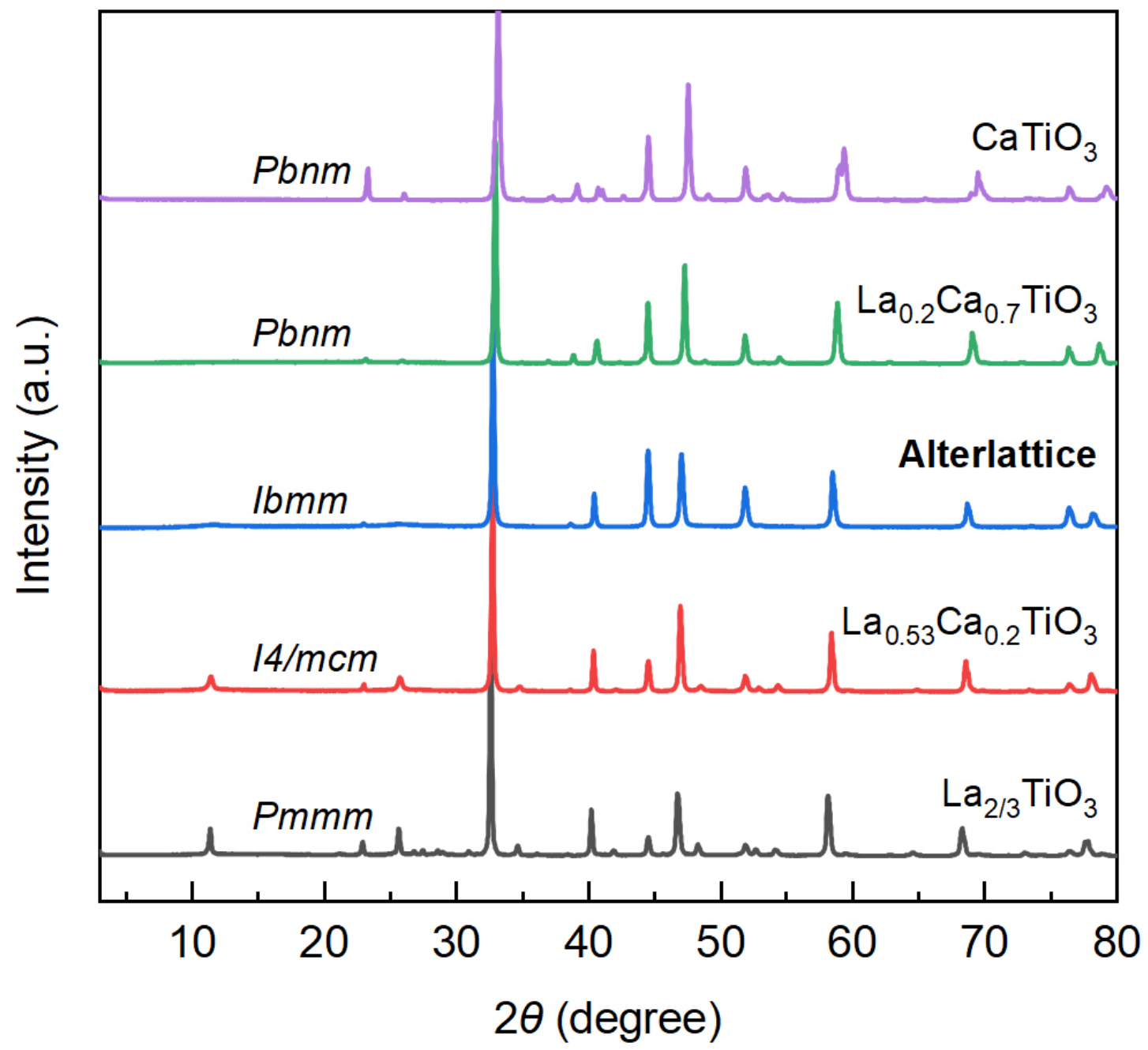

Supplementary Figure 2 | XRD patterns of $La_{2(1-x)/3}Ca_xTiO_3$ (x = 0, 0.2, 0.4, 0.7, 1.0) in the 2θ range of 3–80°. The data reveal the emergence of the alterlattice upon Ca incorporation into the parent phase, driven by A-site chemical disorder and crystallographic phase competition.

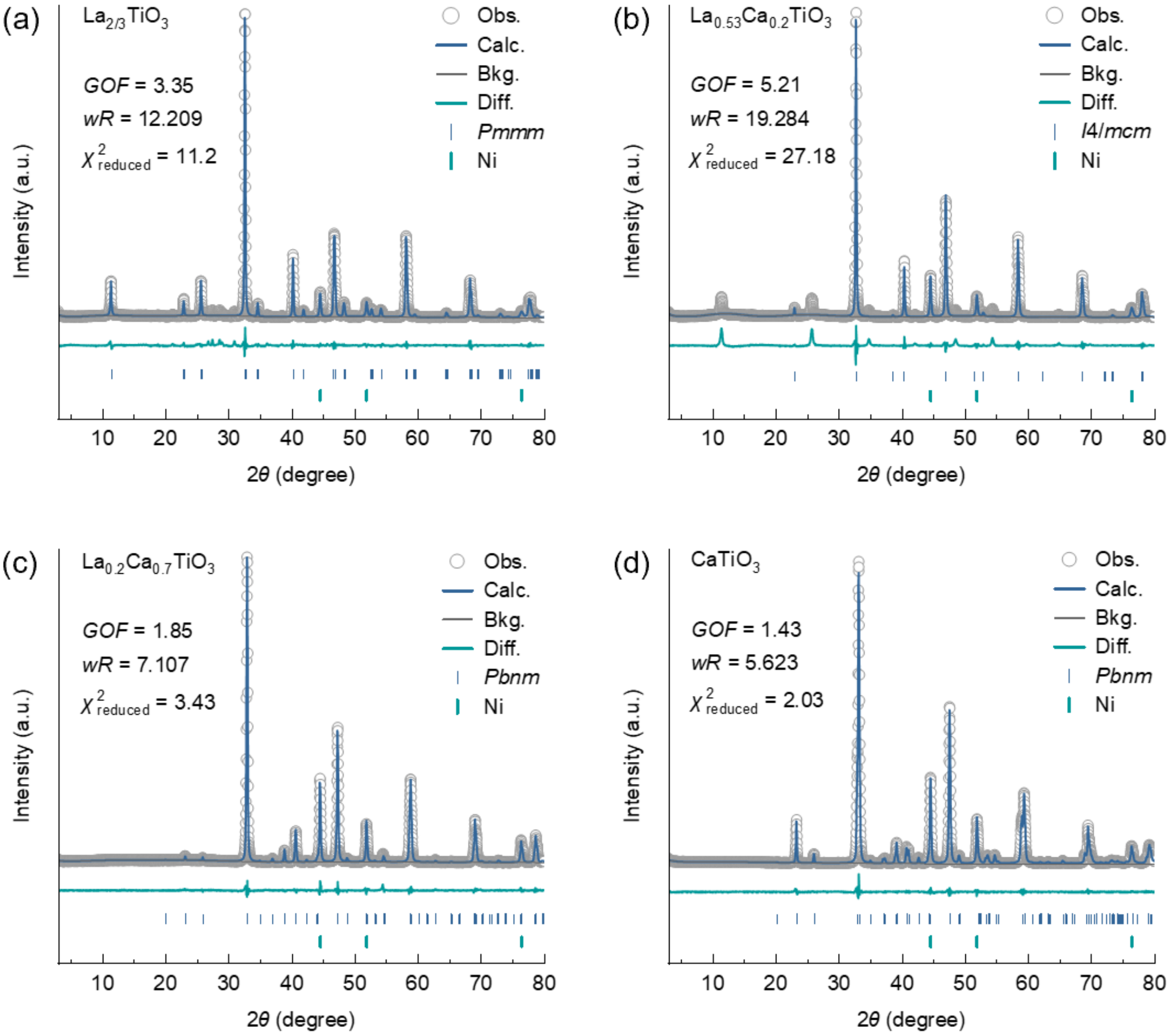

Supplementary Figure 3 | Rietveld refinement profiles for the $La_{2(1-x)/3}Ca_xTiO_3$ series (*x* = 0, 0.2, 0.7, and 1.0). **(a)** *x* = 0 ($La_{2/3}TiO_3$) with *Pmmm* symmetry (ICSD-77671). **(b)** *x*= 0.2 ($La_{0.53}Ca_{0.2}TiO_3$) with *I*4/*mcm* symmetry (ICSD-245544). **(c)** *x* = 0.7 ($La_{0.2}Ca_{0.7}TiO_3$) with *Pbnm* symmetry (ICSD-245542). **(d)** *x* = 1.0 ($CaTiO_3$) with *Pbnm* symmetry (ICSD-150420). Reliability factors (*GOF*, *wR*, reduced χ^2) are given in the inset to each panel. **Note**: Reduced χ^2 represents the variance-weighted residual normalized by the number of degrees of freedom and is used to evaluate the statistical quality of the refinement. *wR* is the weighted residual factor, which describes the weighted difference between the observed and calculated diffraction profiles. *GOF* denotes the goodness of fit and provides an overall measure of the agreement between the experimental and calculated patterns.

Supplementary Table 2 | XRD refinement results of $La_{2(1-x)/3}Ca_xTiO_3$. Chemical formula, space group, and lattice parameters (*a*, *b*, *c*, and *V*) obtained from Rietveld refinement of representative $La_{2(1-x)/3}Ca_xTiO_3$ (x = 0, 0.2, 0.7, 1.0) powder diffraction patterns.

**Chemical formula**	**Space group**	**Lattice parameters**			
		a (Å)	*b* (Å)	*c* (Å)	*V* (Å^3)
$La_{2/3}TiO_3$	*Pmmm*	3.8767(3)	3.8903(3)	7.7755(6)	117.2664(157)
$La_{0.53}Ca_{0.2}TiO_3$	*I*4/*mcm*	5.4711(5)	5.4711(5)	7.7369(8)	231.5881(383)
$La_{0.2}Ca_{0.7}TiO_3$	*Pbnm*	5.4281(3)	5.4517(2)	7.6867(3)	227.4677(175)
$CaTiO_3$	*Pbnm*	5.3822(2)	5.4448(1)	7.6432(2)	223.9840(110)

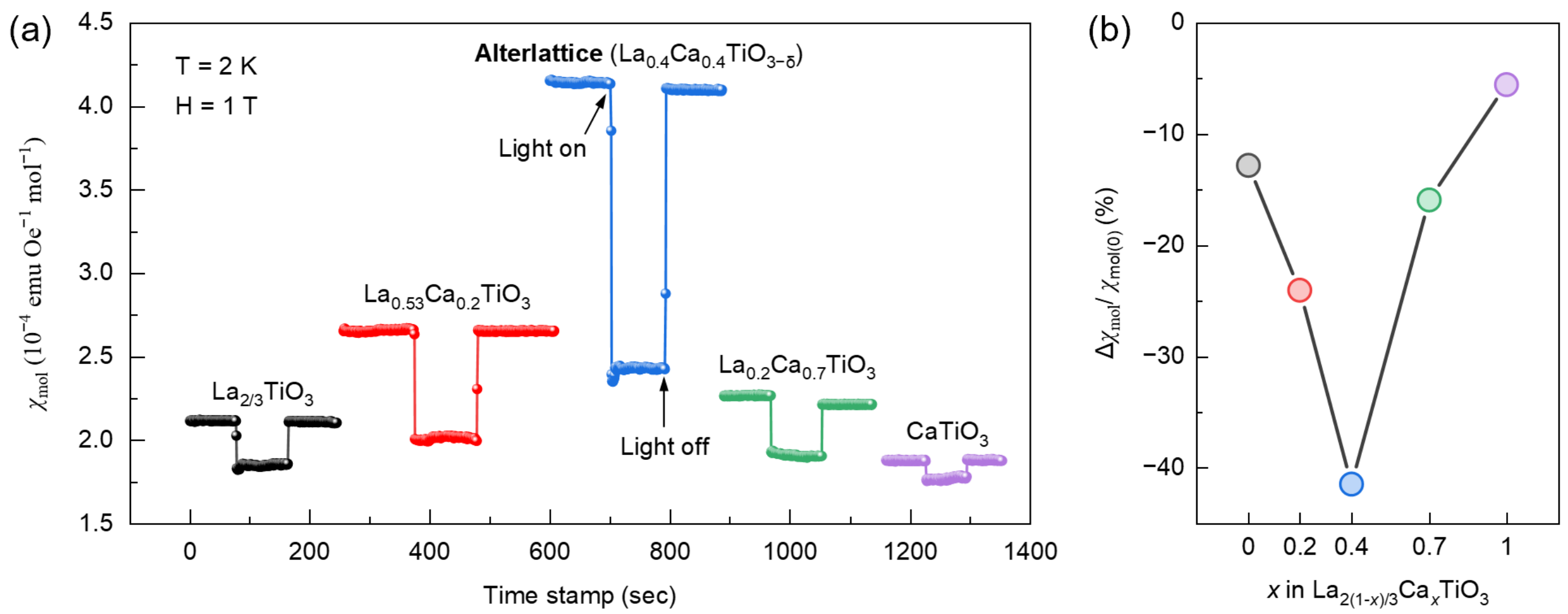

Supplementary Figure 4 | Light on/off photomagnetic measurements of the $La_{2(1-x)/3}Ca_xTiO_3$ series. Time evolution of the molar magnetic susceptibility (χ_{mol}) at 2 K under 1 T using 473 nm illumination with a light intensity of 10 mW mm^{-2}. The measurements show a negative photomagnetic response, characterized by a rapid decrease in χ_{mol} under illumination and recovery after the light is turned off. **(a)** Negative photomagnetic responses of $La_{2(1-x)/3}Ca_xTiO_3$ with $x = 0$, 0.2, 0.4, 0.7, and 1.0. **(b)** Comparison of $\Delta\chi_{mol}/\chi_{mol(0)}$ for different compositions under identical conditions, where $\Delta\chi_{mol}$ is the difference between χ_{mol} under illumination and χ_{mol} in the dark state, and $\chi_{mol(0)}$ is the dark-state value before illumination.

Supplementary Table 3 | XRD refinement results of alterlattice average structure. Lattice parameters (*a*, *b*, *c* and *V*) and refinement factors (Reduced χ^2, *wR*, and *GOF*) obtained from Rietveld refinement of the $La_{0.4}Ca_{0.4}TiO_{3-\delta}$ (alterlattice) powder diffraction pattern in the 2θ = 3–80° range using the *Ibmm* structure model from ICSD-245543.

**Lattice parameters**				**Refinement factors**		
a (Å)	*b* (Å)	*c* (Å)	*V* (Å^3)	Reduced χ^2	*wR*	*GOF*
5.4599(8)	5.4597(7)	7.7345(4)	230.5661(383)	7.04	11.879	2.65

Note: Reduced χ^2 represents the variance-weighted residual normalized by the number of degrees of freedom and is used to evaluate the statistical quality of the refinement. *wR* is the weighted residual factor, which describes the weighted difference between the observed and calculated diffraction profiles. *GOF* denotes the goodness of fit and provides an overall measure of the agreement between the experimental and calculated patterns.

Supplementary Table 4 | XRD refinement results of alterlattice local structure. Lattice parameters (*a*, *b*, *c* and *V*) and refinement factors (Reduced χ^2, *wR*, and *GOF*) obtained from Rietveld refinement of the $La_{0.4}Ca_{0.4}TiO_{3-\delta}$ (alterlattice) powder diffraction pattern in the 2θ = 3–33° range using the *Cmmm* structure model from ICSD-245545.

**Lattice parameters**				**Refinement factors**		
a (Å)	*b* (Å)	*c* (Å)	*V* (Å^3)	Reduced χ^2	*wR*	*GOF*
7.7150(2)	7.7186(3)	7.7415(4)	460.9986(321)	0.91	5.505	0.95

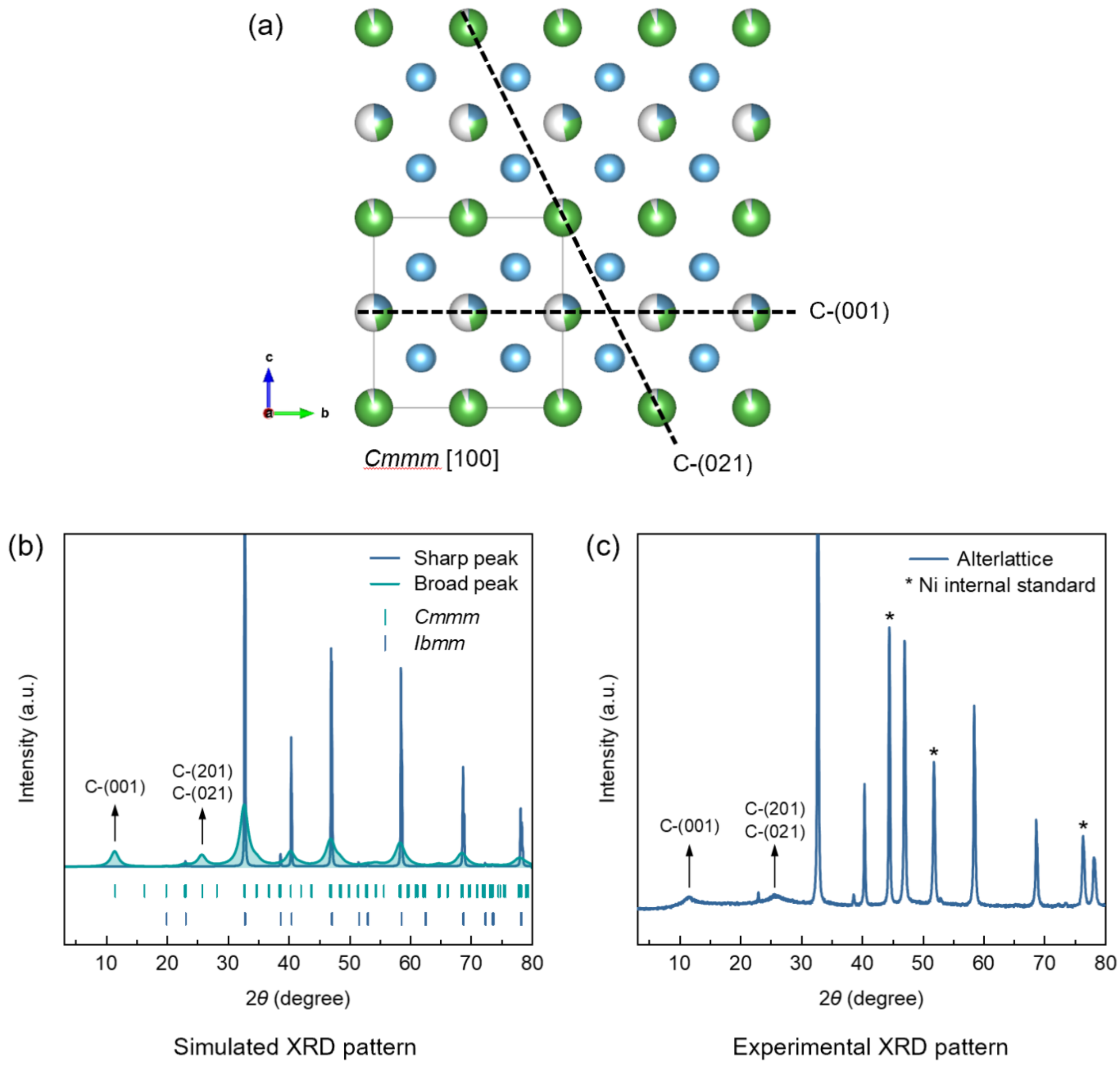

Supplementary Figure 5 | Simulated and experimental XRD patterns comparing the *Ibmm* average structure with the *Cmmm* short-range superstructure contributions in the alterlattice. **(a)** Projected *Cmmm* structural model viewed along the [100] direction, showing the real-space origin of the *Cmmm*-related C-(001) and C-(201)/(021) superstructure reflections in the alterlattice. **(b)** The simulated patterns, obtained using the GSAS program, clarify the structural origins of the diffraction features. Sharp Bragg peaks are dominated by the orthorhombic *Ibmm* average structure, defining the long-range lattice. In contrast, the *Cmmm* A-site vacancy ordered model accounts for the broadened reflections, particularly those below $2\theta = 20°$. **(c)** The experimental pattern exhibits strong, sharp *Ibmm* main reflections, whereas only two broadened *Cmmm*-related superstructure reflections, C-(001) and C-(201)/(021), are observed. **Note**: Asterisks denote peaks from the Ni internal standard. This difference between the simulated and experimental patterns indicates that the *Cmmm* component mainly reflects microscopic A-site vacancy chemical ordering. The associated local distortions are

therefore either short-ranged or averaged into the long-range *Ibmm* structure. The broadening of the low-angle superstructure reflections arises from orthogonally oriented short-range domains and their 90° rotational intergrowth, which produce overlapping diffraction signals in the powder pattern. The absence of sharp, high-angle reflections characteristic of a long-range *Cmmm* phase confirms that the alterlattice does not constitute a conventional long-range chemically ordered phase.

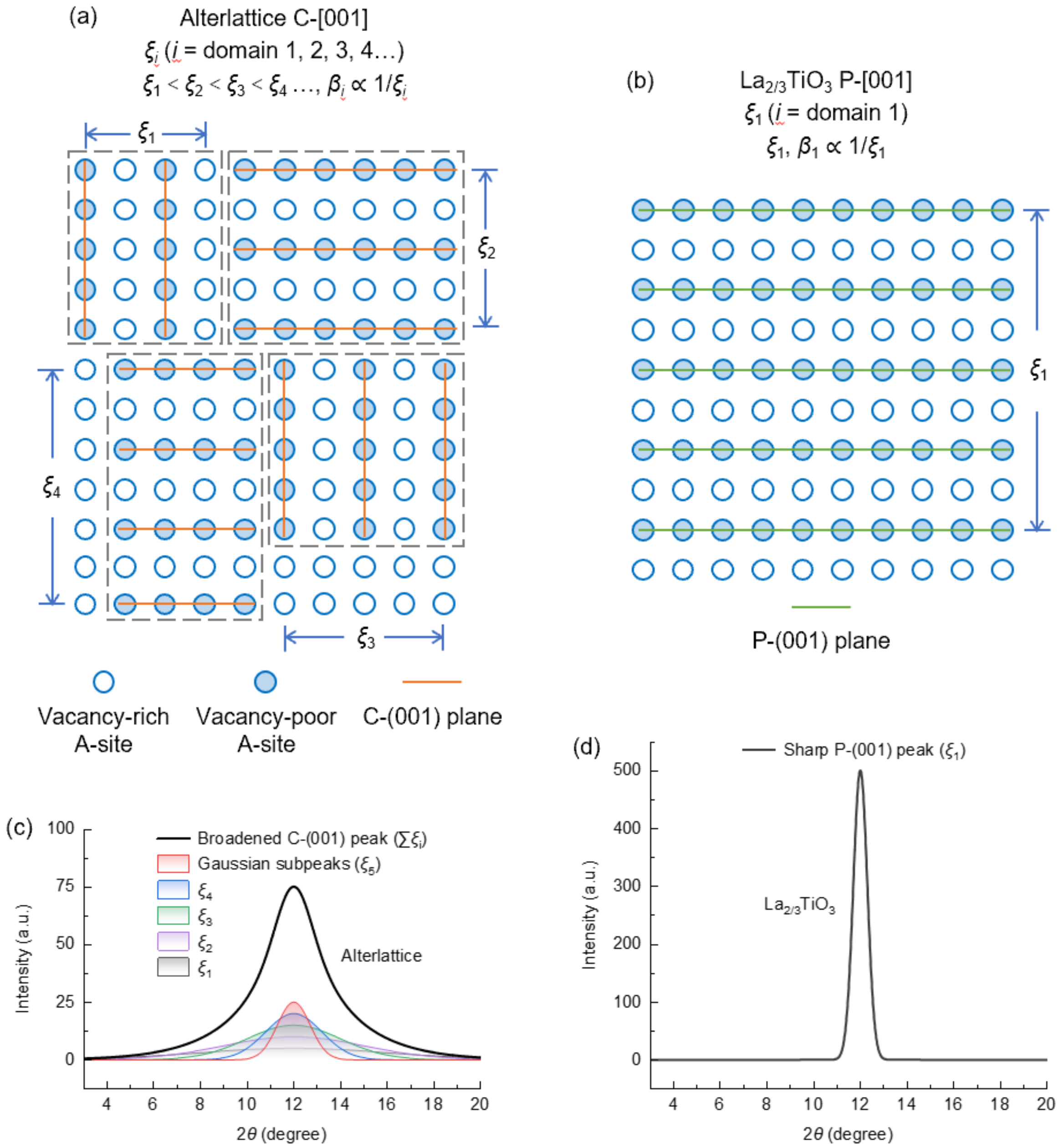

Supplementary Figure 6 | Schematic origin of broadened superstructure reflections in the alterlattice. (a) Schematic illustration of short-range *Cmmm* A-site vacancy ordered domains in the alterlattice viewed along C-[001]. The local domains have finite and distributed superstructure correlation lengths, denoted as ξ_i (i = 1,2,3,4…). Their approximately orthogonal orientations and rotational intergrowth generate multiple partially overlapping superstructure diffraction contributions. Because each local domain has a limited correlation length, these contributions do not form a single sharp long-range Bragg reflection, but instead give weak and broadened superstructure intensity. The peak width follows approximately $\beta_i \propto 1/\xi_i$. **(b)** Schematic illustration of long-range A-site vacancy

ordering in $La_{2/3}TiO_3$ viewed along P-[001]. In contrast to the alterlattice, the vacancy ordering can be approximated by one dominant long correlation length, ξ_1, which produces a much sharper superstructure reflection. **(c)** Schematic representation of the broadened low-angle superstructure peak in the alterlattice. The broad peak can be regarded as the superposition of multiple nearby Gaussian-like diffraction contributions with different intensities and widths. These components originate from *Cmmm* short-range ordered domains with distributed local correlation lengths and different orientations. The broadening mainly reflects finite correlation lengths and orientational intergrowth of A-site vacancy chemical ordering, rather than overall lattice strain. **(d)** Schematic sharp superstructure peak of long-range ordered $La_{2/3}TiO_3$. The longer-range A-site vacancy ordering gives a narrower Bragg-like reflection. The comparison indicates that the low-angle broadened superstructure reflections in the alterlattice most likely originate from short-range A-site vacancy chemical ordering and orthogonal orientational intergrowth, rather than from a long-range single-phase *Cmmm* structure or overall lattice distortion.

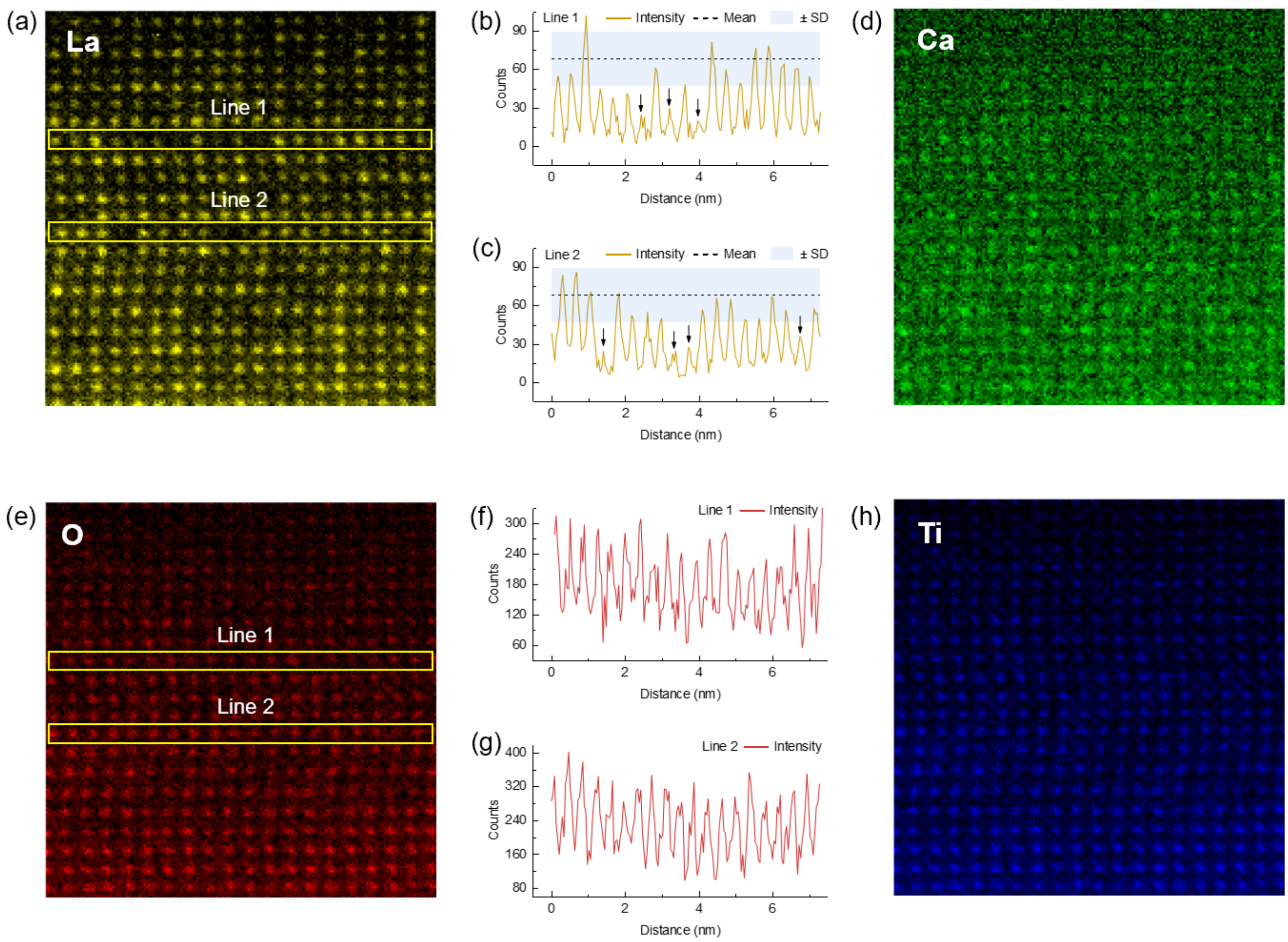

Supplementary Figure 7 | Atomic-scale EELS analysis of local chemical inhomogeneity. (a) EELS map of La (La $M_{4,5}$ edge) along the I-[001] zone axis. **(b)** Line-intensity profile from boxed region 1 in (a), showing La-deficient or La-vacancy-rich regions. Compared with the statistical intensity distribution, the arrow-marked sites show intensities below the mean value, indicating positions where La deficiency is more pronounced. **(c)** Line-intensity profile from region 2 in (a), further confirming La-deficient sites. The yellow curve is the measured intensity profile, the black dashed line is the average intensity from 80 atomic columns, and the blue region represents the mean ± one standard deviation. **(d)** EELS map of Ca (Ca $L_{2,3}$ edge) along the I-[001] zone axis. **(e)** EELS map of O (O K edge) along the I-[001] zone axis. **(f)** Line-intensity profile from boxed region 1 in (e), showing slight variation in the O-related signal. **(g)** Line-intensity profile from boxed region 2 in (e), showing a similar trend. (h) EELS map of Ti (Ti $L_{2,3}$ edge) along I-[001].

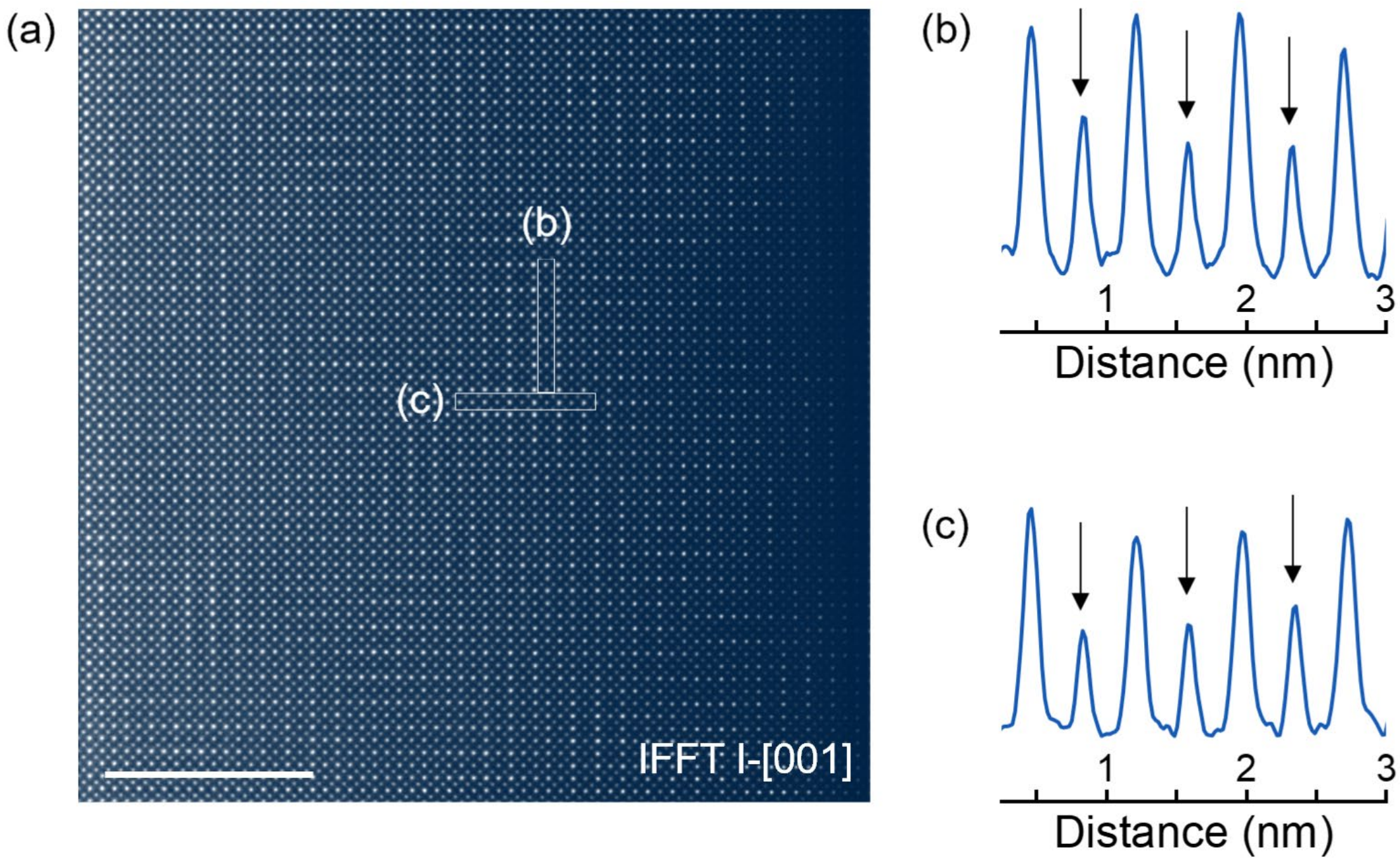

Supplementary Figure 8 | IFFT analysis of A-site vacancy-rich domain in the alterlattice along I-[001]. (a) IFFT image of the alterlattice in $La_{0.4}Ca_{0.4}TiO_{3-\delta}$ along the I-[001] zone axis (scale bar, 5 nm). **(b,c)** Line-scan profiles from the corresponding boxed regions, showing periodic modulations in atomic-column intensity. The arrowed segments mark A-site vacancy rich regions.

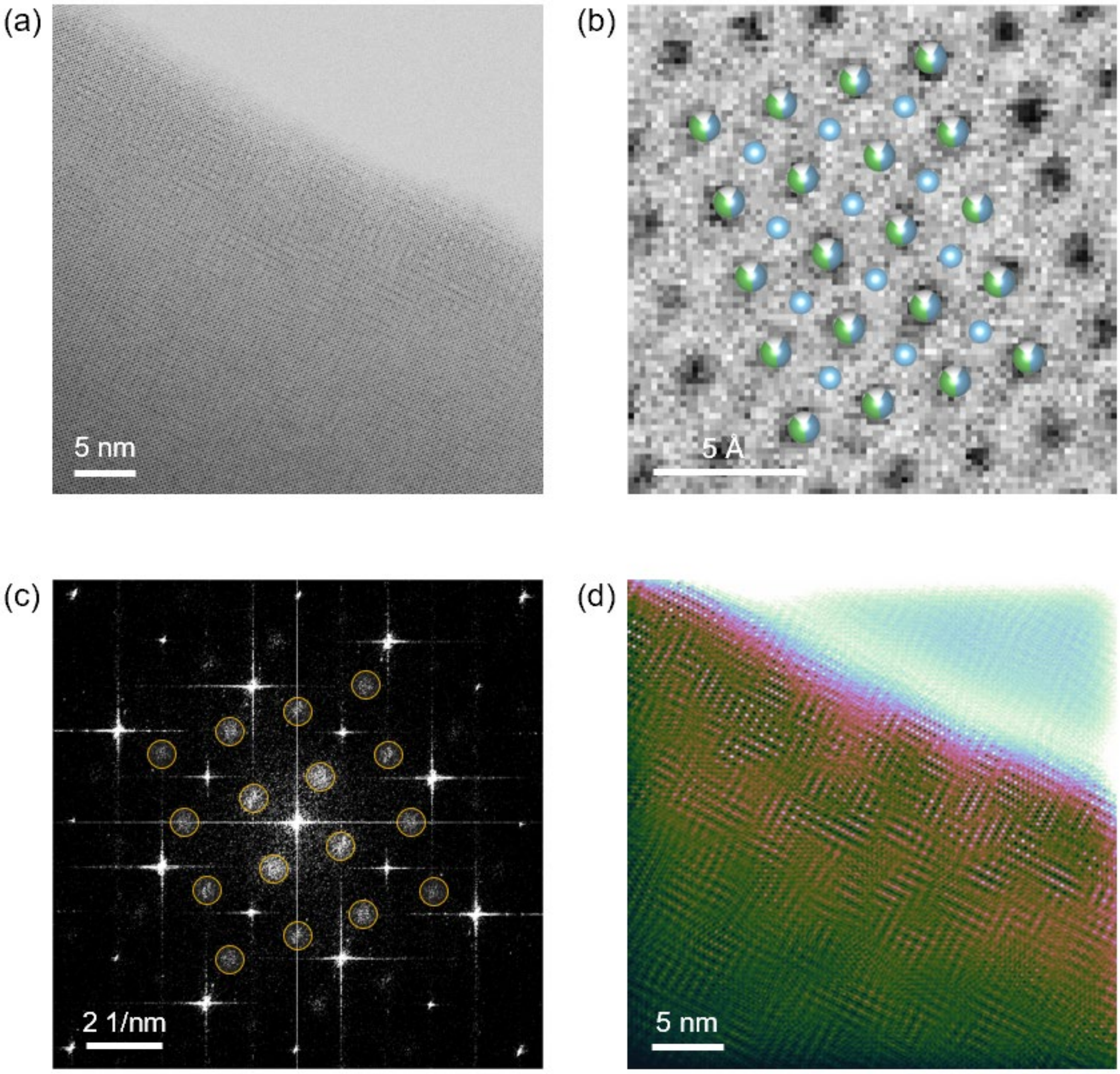

Supplementary Figure 9 | ADF-STEM imaging of the $La_{0.4}Ca_{0.4}TiO_{3-\delta}$ (alterlattice) along the I-[001] zone axis. (a) ADF images acquired along the I-[001] zone axis. **(b)** Magnified ADF view of (a) with the cation sublattice model overlaid. **(c)** FFT patterns from the I-[001] direction, with superlattice reflections marked by yellow circles. **(d)** IFFT reconstructions obtained by selecting the yellow-circled reflections in (c), showing a double-**q** real-space lattice modulation with two orthogonal wave vectors ($\mathbf{q}_1$, $\mathbf{q}_2$).

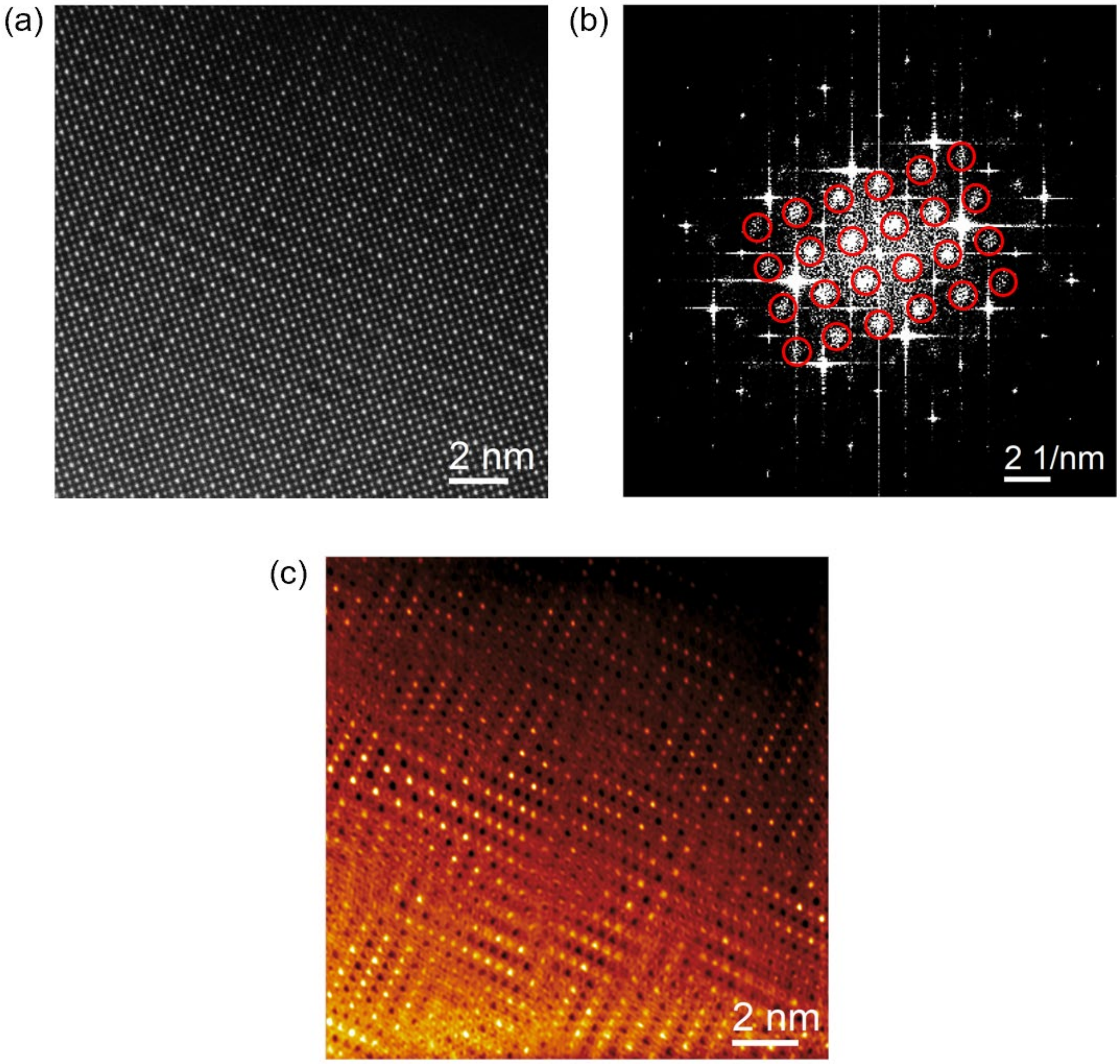

Supplementary Figure 10 | HAADF-STEM characterization of the alterlattice in $La_{0.4}Ca_{0.4}TiO_{3-\delta}$. (a) HAADF-STEM images along the I-[001] zone axes (scale bar, 2 nm). **(b)** FFT pattern calculated from the images in (a); superstructure diffraction spots arising from short-range ordered domains with *Cmmm* symmetry are marked by red circles. **(c)** IFFT reconstruction using only the red-circled spots in (b), showing the real-space lattice modulation associated with the alterlattice.

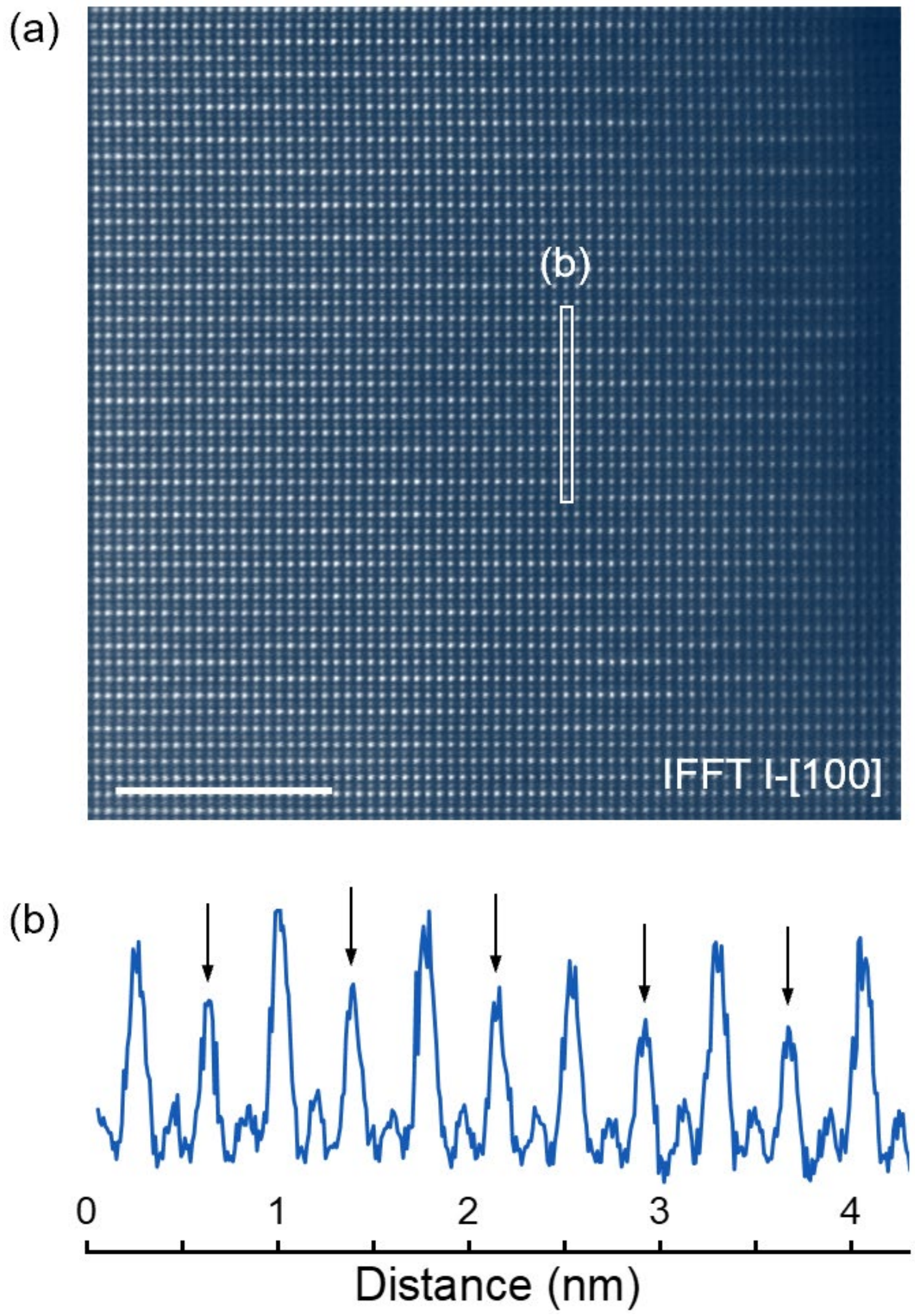

Supplementary Figure 11 | IFFT analysis of A-site vacancy-rich domain in the alterlattice along I-[100]. (a) IFFT image of the alterlattice in $La_{0.4}Ca_{0.4}TiO_{3-\delta}$ along the [100] zone axis (scale bar, 5 nm). **(b)** Line-scan profiles from the corresponding boxed region b in (a), showing periodic modulations in atomic-column intensity. The arrowed segments mark A-site vacancy rich regions.

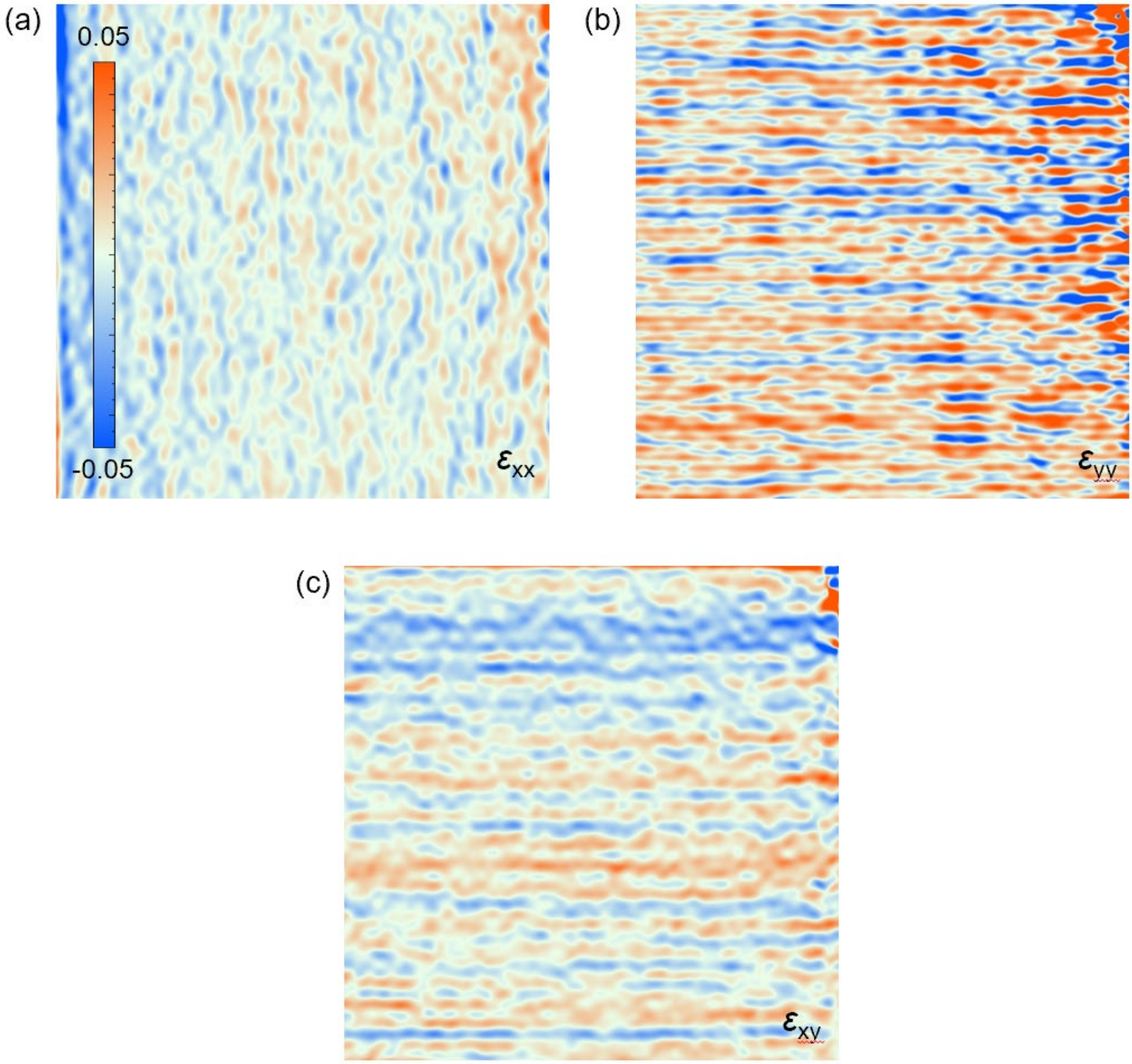

Supplementary Figure 12 | GPA maps of the alterlattice in $La_{0.4}Ca_{0.4}TiO_{3-\delta}$. The results showing the in-plane normal strain ε_{xx} **(a)** and out-of-plane normal strain ε_{yy} **(b)**, and the in-plane shear strain ε_{xy} **(c)** along the I-[100] zone axis. GPA was performed on the HAADF image in Figure 2g. The color scale spans −0.05 (blue, compression) to +0.05 (red, tension), revealing pronounced anisotropic local lattice distortions.

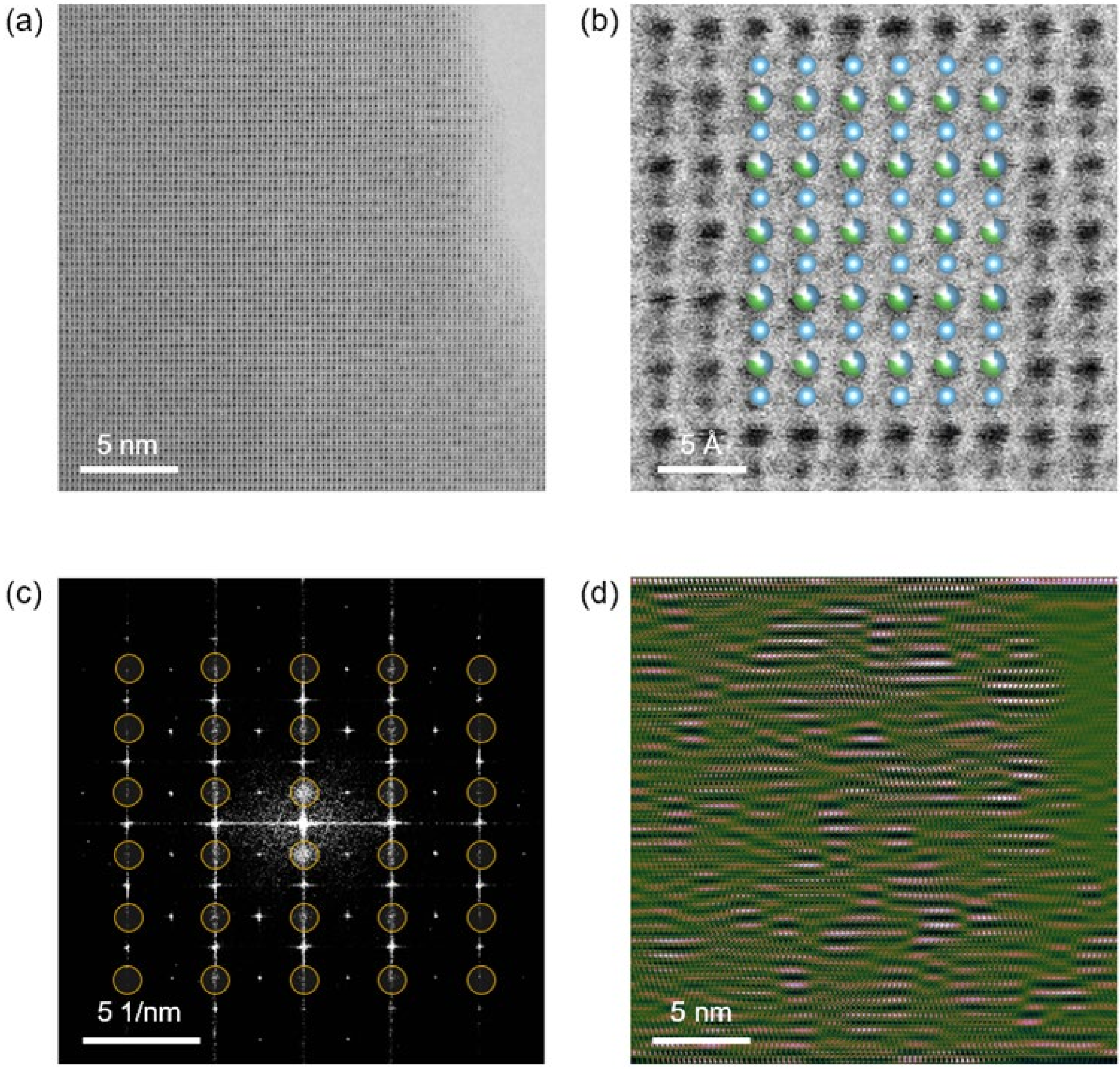

Supplementary Figure 13 | ADF-STEM characterization of the alterlattice in $La_{0.4}Ca_{0.4}TiO_{3-\delta}$ along the I-[100] zone axis. (a) ADF images acquired along the I-[100] zone axis. **(b)** Magnified view of the region in a with the cation sublattice model overlaid. **(c)** FFT of the image in a; superlattice reflections are indicated by yellow circles. **(d)** IFFT reconstruction using only the yellow-circled reflections in c, revealing a single-**q** real-space lattice modulation with one wave vector ($\mathbf{q}_3$).

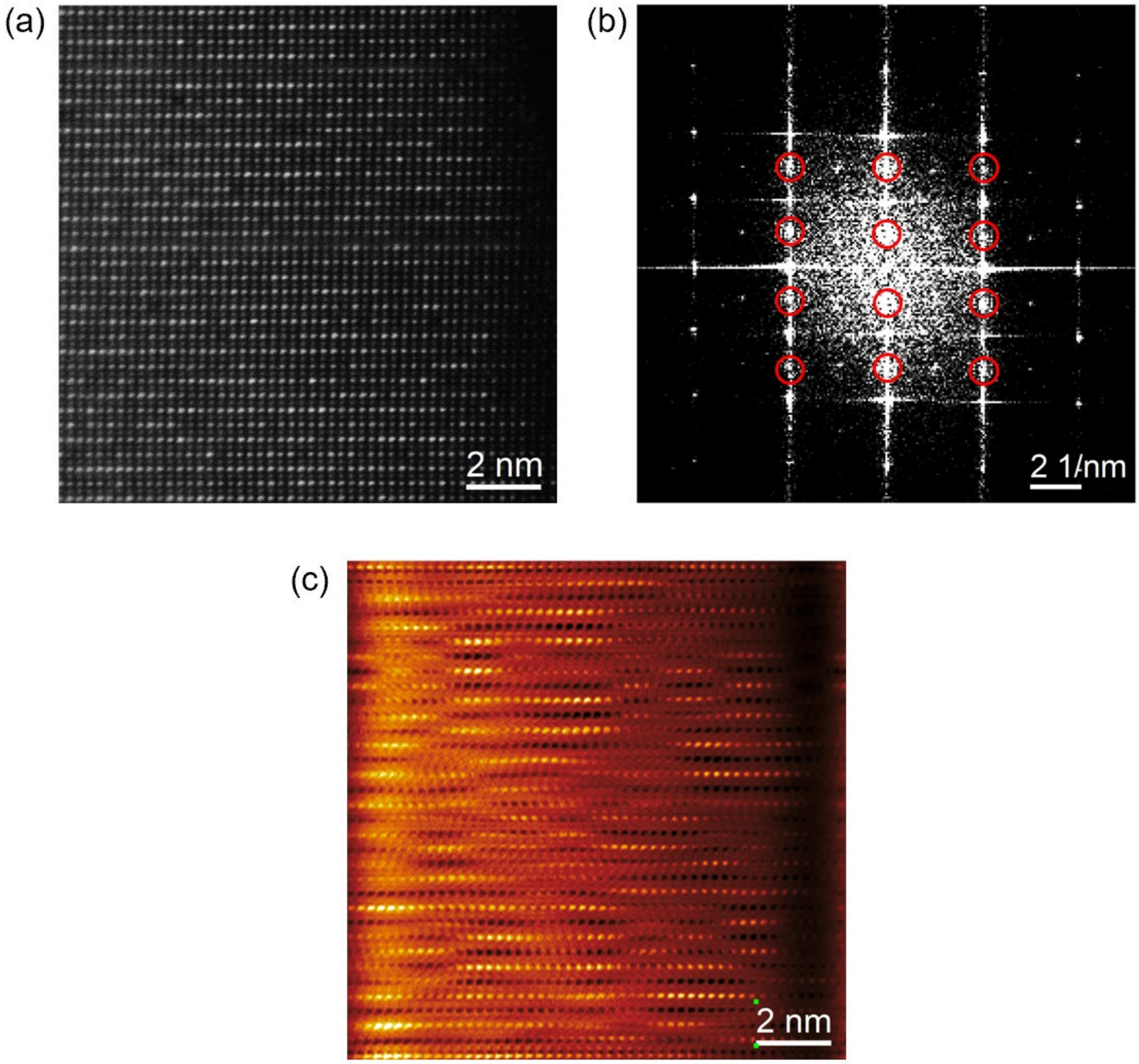

Supplementary Figure 14 | HAADF-STEM characterization of the alterlattice in $La_{0.4}Ca_{0.4}TiO_{3-\delta}$. (a) HAADF-STEM images along the I-[100] zone axes (scale bar, 2 nm). **(b)** FFT pattern calculated from the images in (a); superstructure diffraction spots arising from short-range ordered domains with *Cmmm* symmetry are marked by red circles. **(c)** IFFT reconstruction using only the red-circled spots in (b), showing the real-space lattice modulation associated with the alterlattice.

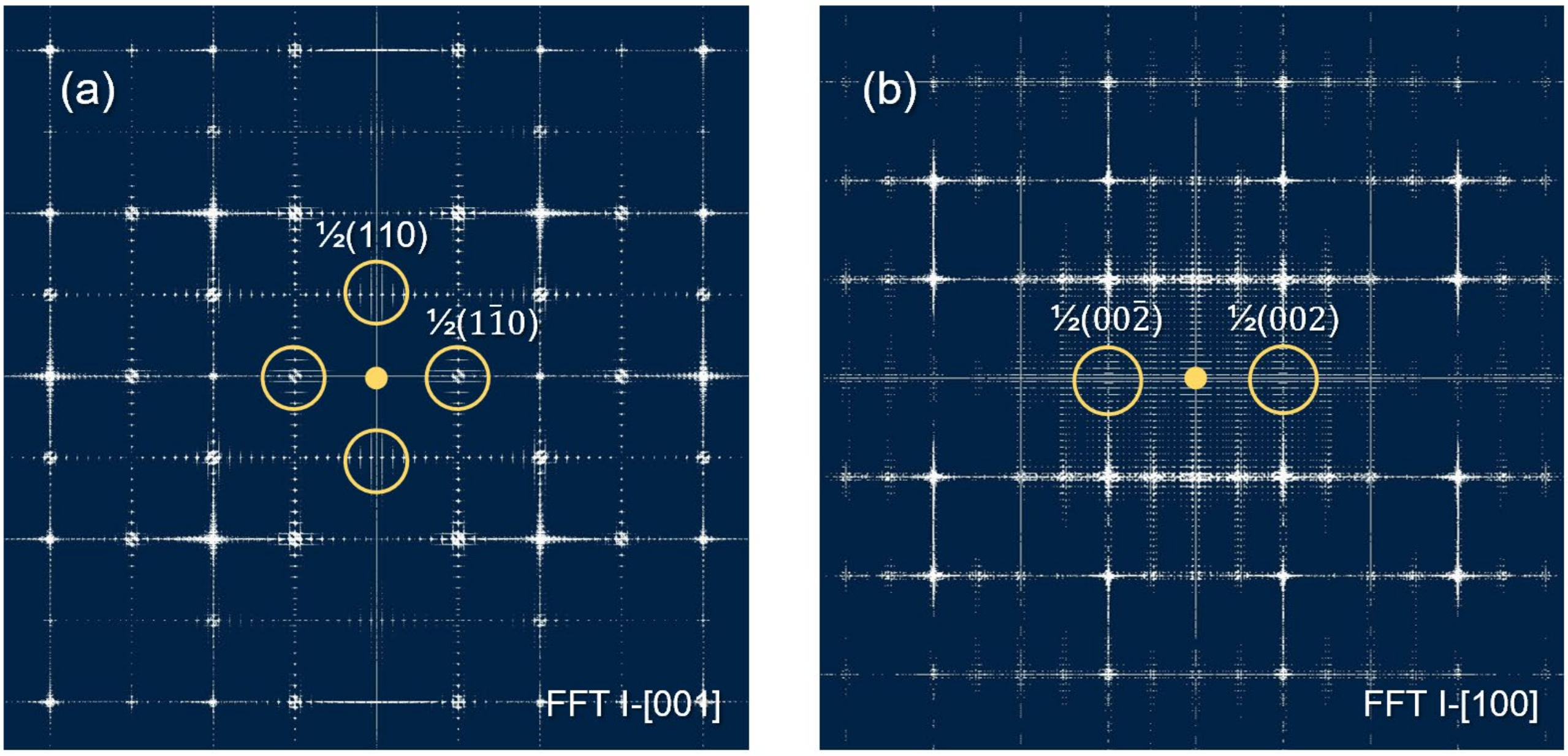

Supplementary Figure 15 | FFT pattern calculated from the idealized alterlattice atomic model shown in Figs. 3a and 3e. The pattern reproduces V_A short-range order superstructure features and long-range *Ibmm* host reflections observed experimentally along **(a)** I-[001] and **(b)** I-[100].

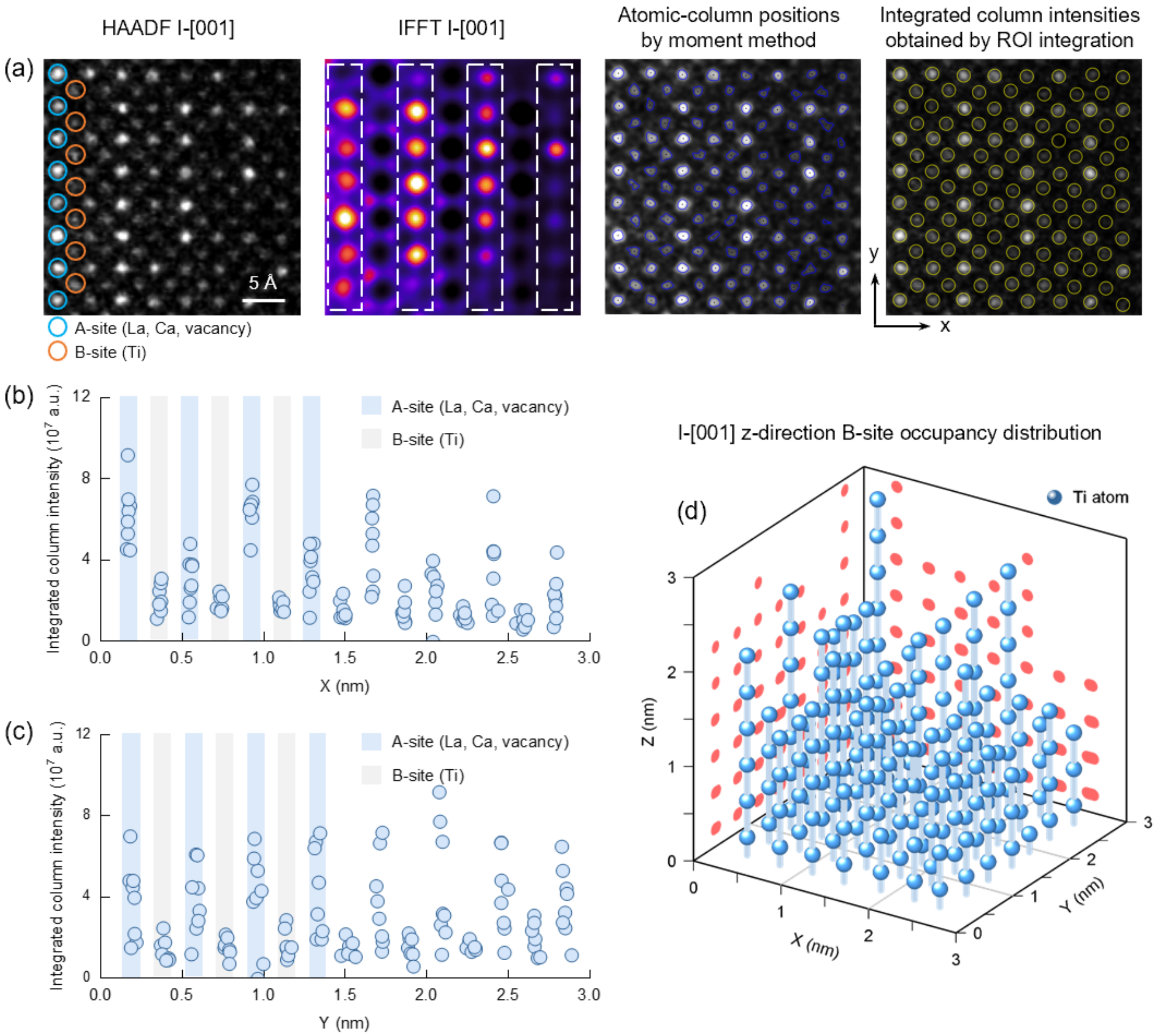

Supplementary Figure 16 | Quantitative HAADF-STEM intensity analysis of the I-[001] *Cmmm* superstructure domain. (a) From left to right, atomic-resolution HAADF-STEM image of a thin region in $La_{0.4}Ca_{0.4}TiO_{3-\delta}$ viewed along I-[001], IFFT image reconstructed using only the superstructure spots, atomic-column positions determined by the moment method, and ROI-based integrated column intensity map. The selected thin region falls within the approximate linear atom number-contrast regime. The IFFT image highlights a single-**q** *Cmmm* superstructure domain embedded in the average perovskite framework. The moment-fitted atomic-column coordinates were used as the centers for ROI integration of the HAADF intensity. **(b)** Integrated column intensity distribution plotted as a function of x. The alternating A-site and B-site columns show distinct intensity levels, mainly arising from the different average atomic numbers of the A-site columns,

La/Ca/vacancy, and the B-site Ti columns. **(c)** Integrated column intensity distribution plotted as a function of y. The projected intensity modulation further confirms that the contrast variation is spatially correlated with the *Cmmm* superstructure domain rather than random imaging noise. **(d)** I-[001] intensity-derived z-direction B-site occupancy distribution. The z-direction Ti occupancy was estimated semi-quantitatively from the integrated B-site column intensities using the thin-specimen approximation, ($I_{col} \propto N_{Ti}Z_{Ti}^{\alpha}$). The obtained B-site occupancy distribution indicates a nanoscale *Cmmm* domain with an approximate spatial extent of about 3×3×3 nm^3.

To obtain a more quantitative description of the local *Cmmm* intergrowth structure, we performed integrated HAADF-STEM column-intensity analysis on thin regions viewed along I-[001] (Supplementary Figure 16) and I-[100] (Supplementary Figure 17). This analysis supplements the projected HAADF contrast by providing semi-quantitative information on the z-direction B-site atomic occupancy and offers a more constrained description of the nanoscale *Cmmm* domain geometry. The workflow includes q-selective inverse fast Fourier transform reconstruction of the superstructure contrast, moment-based atomic-column localization using the CalAtom software[8], region-of-interest-based column intensity integration (Supplementary Figures 16a and 17a), and semi-quantitative conversion of B-site integrated intensities into z-direction Ti occupancy distributions (Supplementary Figures 16d and 17d).

For the I-[001] analysis (Supplementary Figure 16a), the HAADF-STEM image was taken from a thin region of Fig. 2a, where the contrast is closer to the linear atom number-contrast regime. The superstructure spots were isolated in reciprocal space using an IFFT mask around the selected q vector. Atomic-column positions were determined by the moment method using an average atomic radius of 10 pixels and a contour threshold of 0.5. Integrated column intensities were then obtained by circular region-of-interest integration with a signal radius of 7 pixels. The x- and y-dependent intensity profiles show clear intensity separation between the A-site columns, La/Ca/vacancy, and the B-site Ti columns. The same procedure was applied to a thin region of Fig. 2g for the I-[100] analysis. The q-selective IFFT reconstruction again reveals *Cmmm* superstructure contrast, while the moment-fitted atomic positions and ROI-integrated intensities provide a column-by-column description of the projected local domain. The intensity distributions along both x (Supplementary Figure 17b) and y

(Supplementary Figure 17c) directions further show the contrast difference associated with the atomic-number difference between the A-site and B-site columns.

The z-direction B-site occupancy distribution was estimated from the integrated Ti-column intensities using the approximate thin-specimen relation[9-11] $I_{col} \propto N_{Ti} Z_{Ti}^{\alpha}$, where N_{Ti} is the number of Ti atoms along the projected column, Z_{Ti} is the atomic number of Ti, and α was set to 1.7. The B site was selected for this estimation because it contains only Ti, avoiding the ambiguity introduced by mixed A-site occupation by La, Ca, and vacancies. The background-corrected integrated intensity of each B-site column was normalized against a reference column with an estimated maximum occupancy of 8 Ti atoms. Because absolute atom counting from HAADF intensity can be affected by thickness variation, channeling, and residual sample tilt, this analysis is treated as a semi-quantitative occupancy visualization rather than a definitive three-dimensional atomic reconstruction. The I-[001] (Supplementary Figure 16d) and I-[100] (Supplementary Figure 17d) analyses indicate that the *Cmmm* superstructure domains are confined to nanoscale volumes of approximately $3\times3\times3$ nm^3 and $3.5\times3.5\times3.5$ nm^3, respectively. The pseudo-cubic grain constraint is justified by the reported Cmmm lattice parameters, a = 7.7150(2) Å, b = 7.7186(3) Å, and c =7.7415(4) Å, which are close in magnitude. Therefore, the *Cmmm* domains can be approximated as pseudo-cubic grains for geometric visualization. These domain sizes are close to the average apparent ordering correlation length of about 3.4 nm estimated from Scherrer analysis of the C-(001) superstructure reflection in the XRD pattern. The agreement between the local HAADF-STEM intensity analysis and the reciprocal-space XRD estimate supports the model that the alterlattice consists of three-dimensionally intergrown *Cmmm* domains with nearly cubic geometric sizes, forming a quasi-mesoscopically ordered structure.

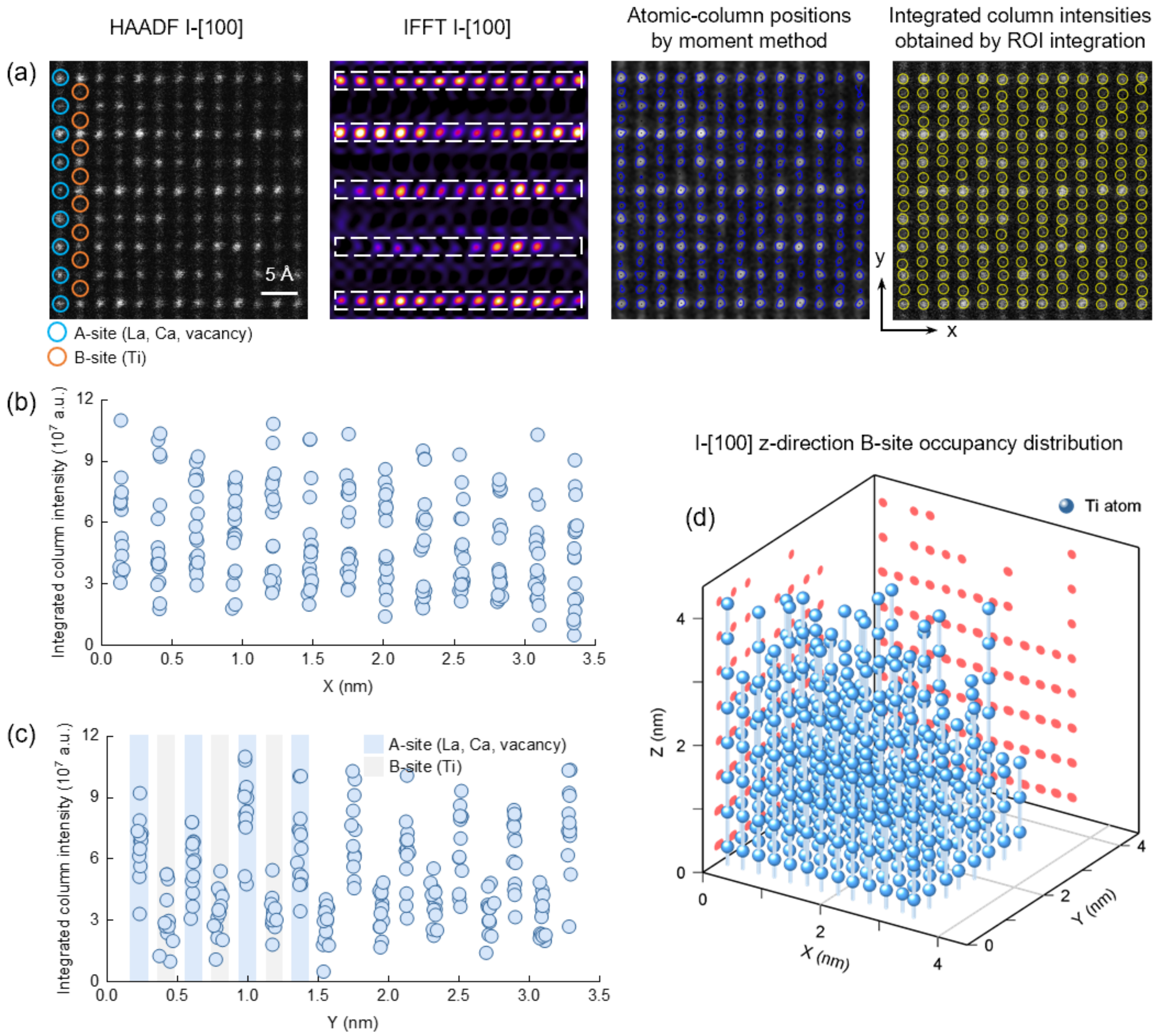

Supplementary Figure 17 | Quantitative HAADF-STEM intensity analysis of the I-[100] *Cmmm* superstructure domain. (a) From left to right, atomic-resolution HAADF-STEM image of a thin region in $La_{0.4}Ca_{0.4}TiO_{3-\delta}$ viewed along I-[100], IFFT image reconstructed using only the superstructure spots, atomic-column positions determined by the moment method, and ROI-based integrated column intensity map. The selected thin region falls within the approximate linear atom number-contrast regime. The IFFT image highlights a single-**q** *Cmmm* superstructure domain embedded in the average perovskite framework. The moment-fitted atomic-column coordinates were used as the centers for ROI integration of the HAADF intensity. **(b)** Integrated column intensity distribution plotted as a function of x. The alternating A-site and B-site columns show distinct intensity levels, mainly arising from the different average atomic numbers of the A-site columns,

La/Ca/vacancy, and the B-site Ti columns. **(c)** Integrated column intensity distribution plotted as a function of y. The projected intensity modulation further confirms that the contrast variation is spatially correlated with the *Cmmm* superstructure domain rather than random imaging noise. **(d)** I-[100] intensity-derived z-direction B-site occupancy distribution. The z-direction Ti occupancy was estimated semi-quantitatively from the integrated B-site column intensities using the thin-specimen approximation, ($I_{col} \propto N_{Ti}Z_{Ti}^{\alpha}$). The obtained B-site occupancy distribution indicates a nanoscale *Cmmm* domain with an approximate spatial extent of about 3.5×3.5×3.5 nm^3.

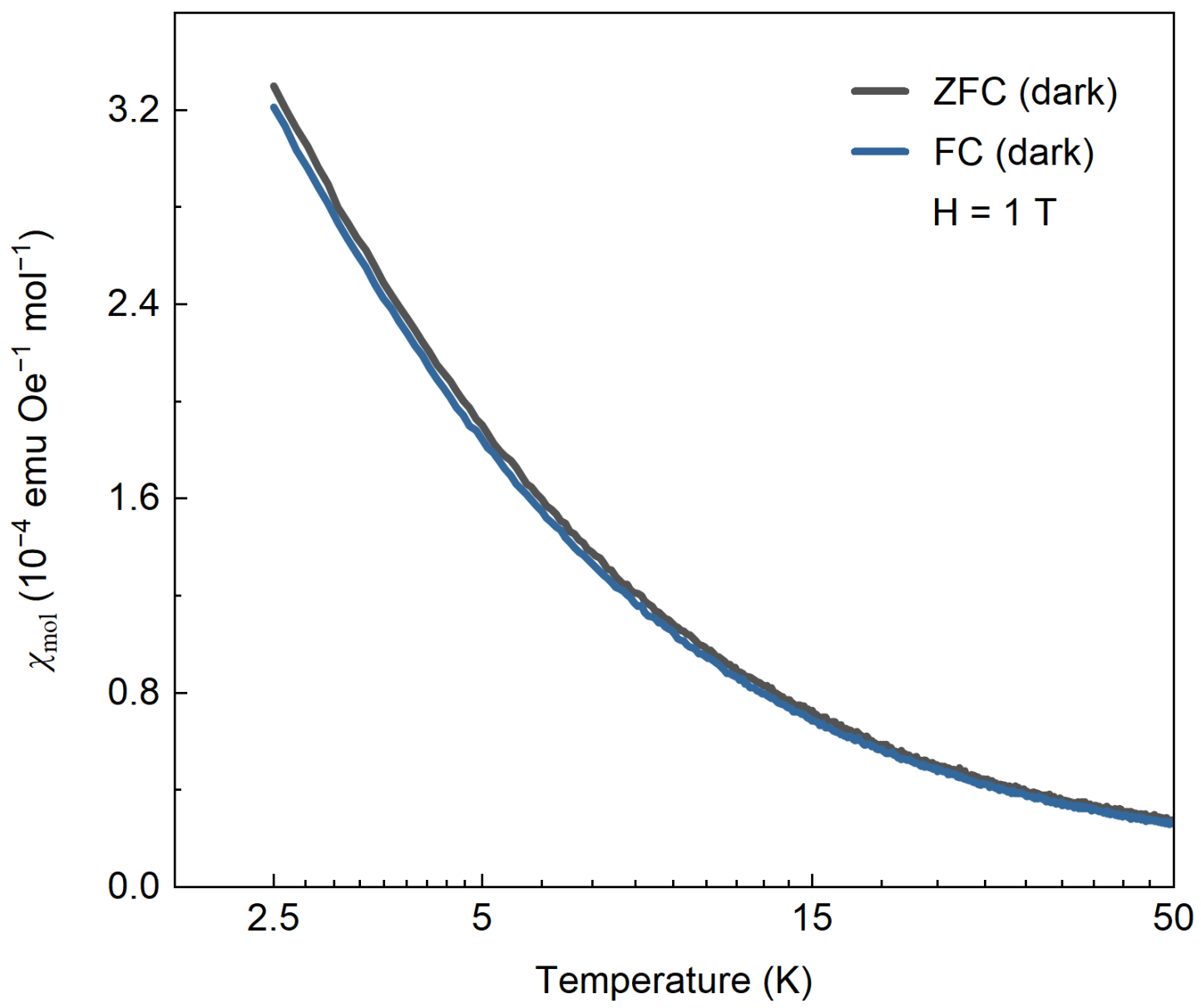

Supplementary Figure 18 | Nearly overlapping ZFC and FC curves characteristic of paramagnetic behaviour. ZFC and FC curves of the 0.48 mm-thick alterlattice sample measured at H = 1 T under dark conditions. The two curves are nearly identical, indicating that the magnetic behavior of the sample under dark conditions is close to ideal paramagnetism.

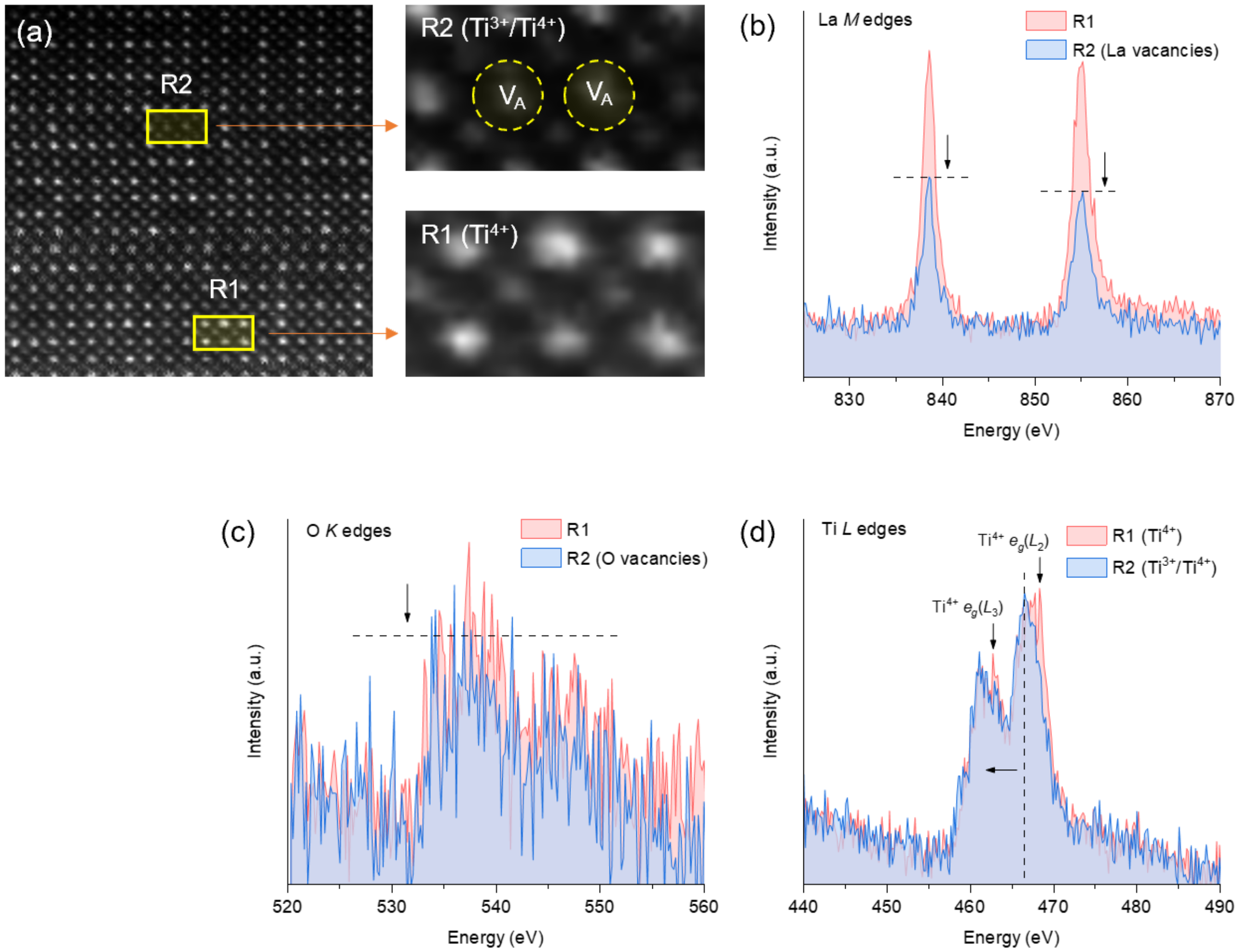

Supplementary Figure 19 | EELS evidence for the spatial correlation of A-site vacancies, oxygen deficiency, and Ti^{3+} character. (a) HAADF-STEM image of the alterlattice in $La_{0.4}Ca_{0.4}TiO_{3-\delta}$ along the I-[001] zone axis. The A-site-vacancy-depleted region (R1) and A-site-vacancy-enriched region (R2) are marked. **(b)** La $M_{4,5}$ edge intensity spectra extracted from the R1 and R2 regions. The La intensity in R2 is significantly lower than that in R1, indicating La deficiency in R2. **(c)** O *K* edge intensity spectra extracted from the R1 and R2 regions. The O intensity in R2 is slightly lower than that in R1, suggesting possible oxygen deficiency in R2. **(d)** Ti $L_{2,3}$ edge spectra extracted from the R1 and R2 regions. Compared with R1, the e_g feature associated with Ti^{4+} is markedly reduced in R2, indicating the presence of partial Ti^{3+} character in R2.

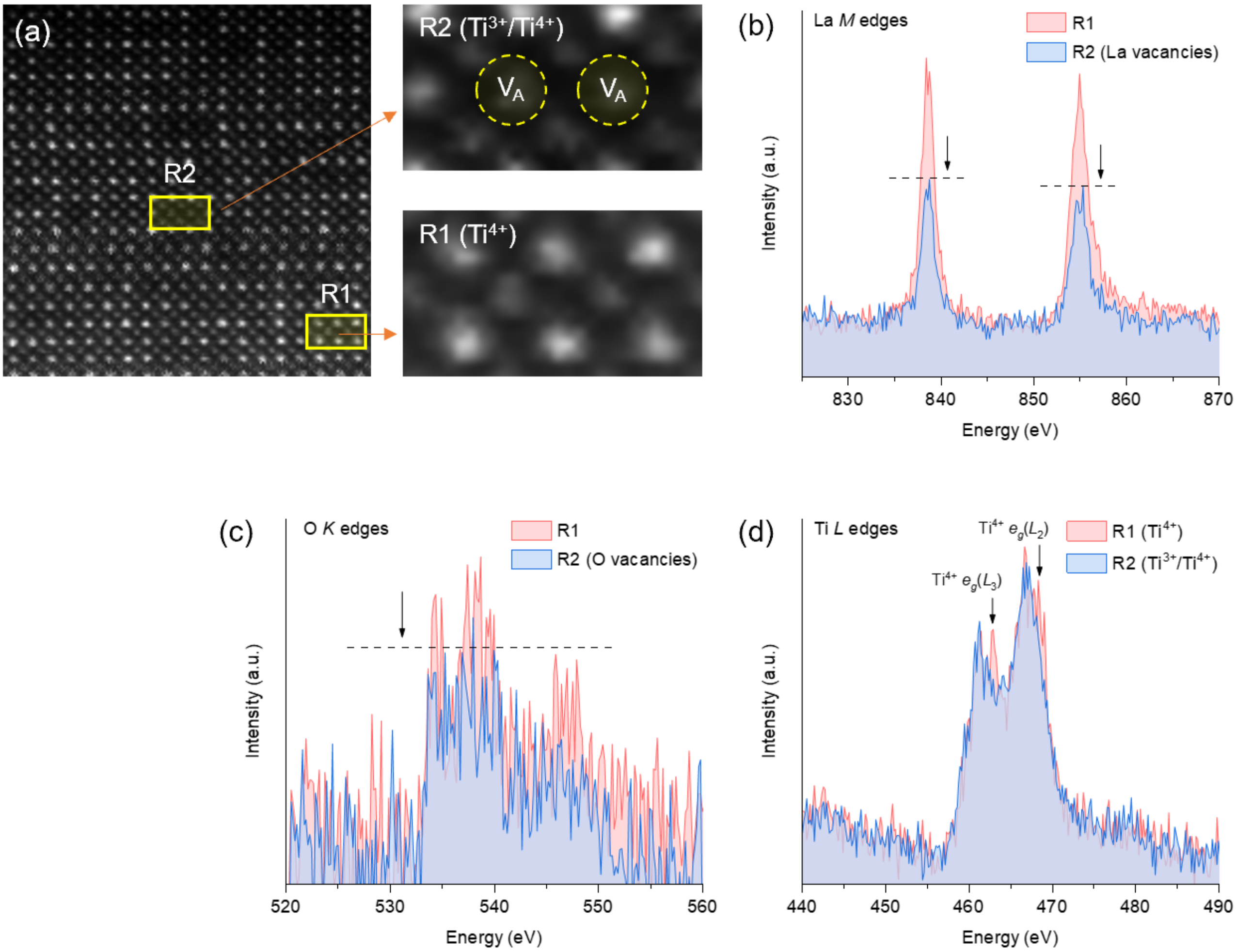

Supplementary Figure 20 | Independent EELS evidence for the spatial correlation of A-site vacancies, oxygen deficiency, and Ti^{3+} character. (a) Another HAADF-STEM image of the alterlattice in $La_{0.4}Ca_{0.4}TiO_{3-\delta}$ along the I-[001] zone axis used for repeat verification. The A-site vacancy-depleted region (R1) and A-site vacancy-enriched region (R2) are marked. **(b)** La $M_{4,5}$ edge intensity spectra extracted from the R1 and R2 regions. The La intensity in R2 is significantly lower than that in R1, indicating La deficiency in R2. **(c)** O *K* edge intensity spectra extracted from the R1 and R2 regions. The O intensity in R2 is slightly lower than that in R1, suggesting possible oxygen deficiency in R2. **(d)** Ti $L_{2,3}$ edge spectra extracted from the R1 and R2 regions. Compared with R1, the e_g feature associated with Ti^{4+} is markedly reduced in R2, indicating the presence of partial Ti^{3+} character in R2.

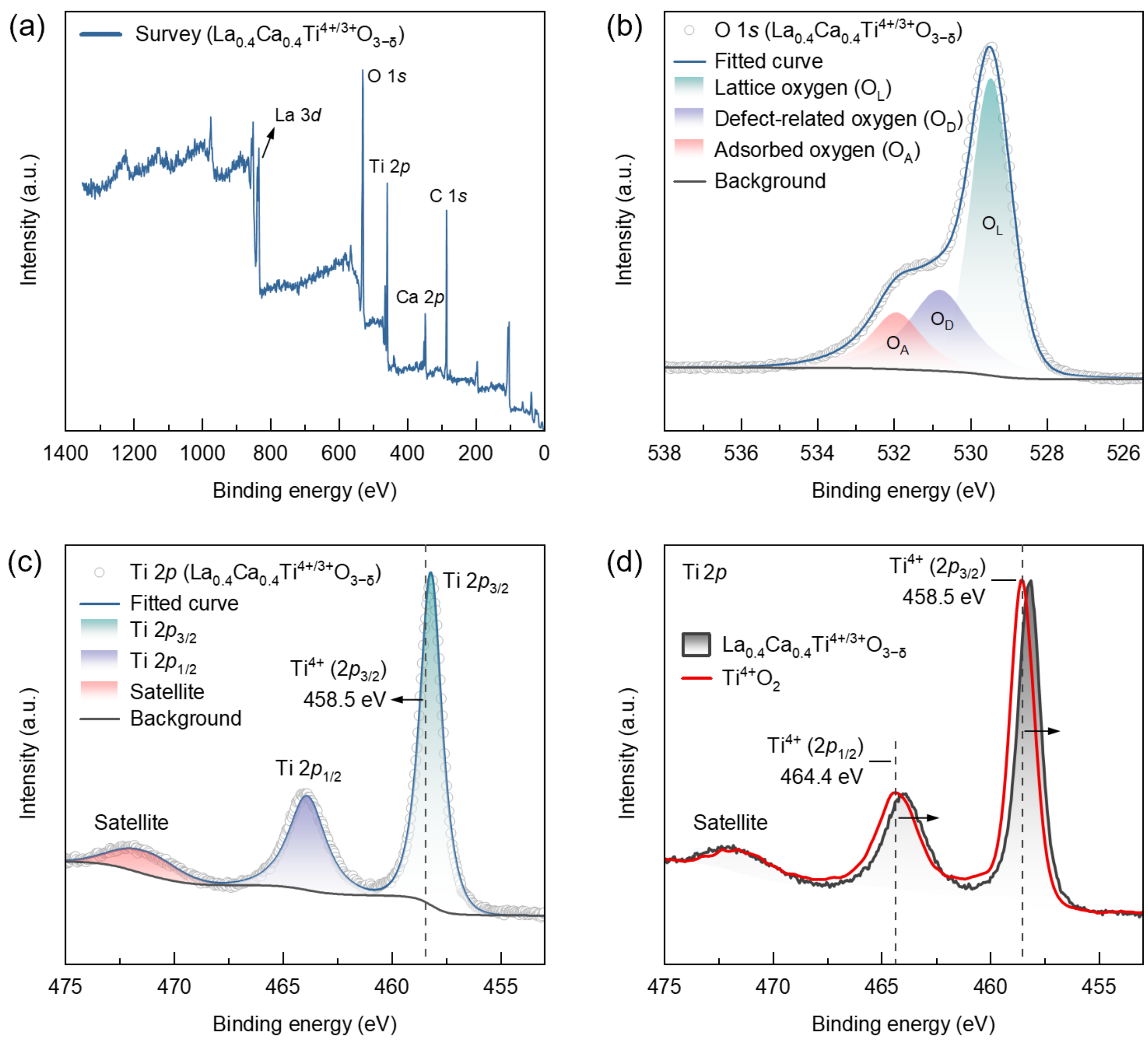

Supplementary Figure 21 | XPS analysis of $La_{0.4}Ca_{0.4}TiO_{3-δ}$. (a) Survey XPS spectrum showing the characteristic La 3*d*, Ca 2*p*, Ti 2*p*, O 1*s*, and C 1*s* signals. **(b)** High-resolution O 1*s* spectrum fitted with three components assigned to lattice oxygen (O_L), defect-related oxygen (O_D), and adsorbed oxygen (O_A). The defect-related oxygen component is consistent with oxygen nonstoichiometry and local defect structures in $La_{0.4}Ca_{0.4}TiO_{3-δ}$. **(c)** High-resolution Ti 2*p* spectrum and peak-fitting result. The Ti 2*p* spectrum is dominated by Ti $2p_{3/2}$ and Ti $2p_{1/2}$ components, together with a weak satellite contribution. Compared with the typical Ti^{4+} $2p_{3/2}$ binding energy at approximately 458.5 eV, the Ti $2p_{3/2}$ peak in $La_{0.4}Ca_{0.4}TiO_{3-δ}$ shifts toward lower binding energy, indicating partial reduction of Ti^{4+} and the presence of a small amount of Ti^{3+}. **(d)** Comparison of Ti 2*p* XPS spectra between $La_{0.4}Ca_{0.4}TiO_{3-δ}$ and TiO_2, emphasizing the distinct lower-binding-energy shift of the Ti $2p_{3/2}$ and Ti $2p_{1/2}$ peaks in $La_{0.4}Ca_{0.4}TiO_{3-δ}$.

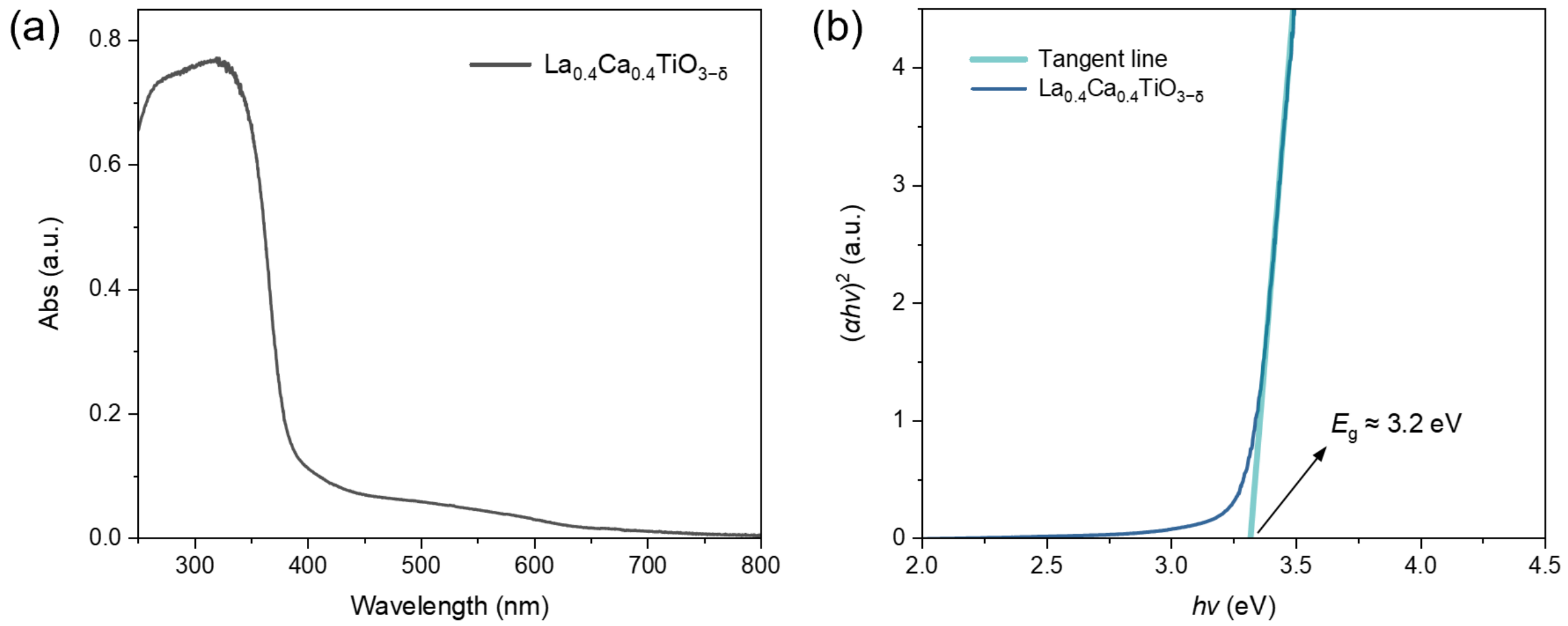

Supplementary Figure 22 | Optical band-gap determination of $La_{0.4}Ca_{0.4}TiO_{3-\delta}$. (a) UV-vis spectrum of the $La_{0.4}Ca_{0.4}TiO_{3-\delta}$ (alterlattice) powders. **(b)** Optical band-gap determination from UV–Vis diffuse reflectance data. Tauc plots used to extract the band-gap energy for the assumed direct transitions. Linear extrapolation yields the estimated optical band-gap values (E_g = 3.2 eV).

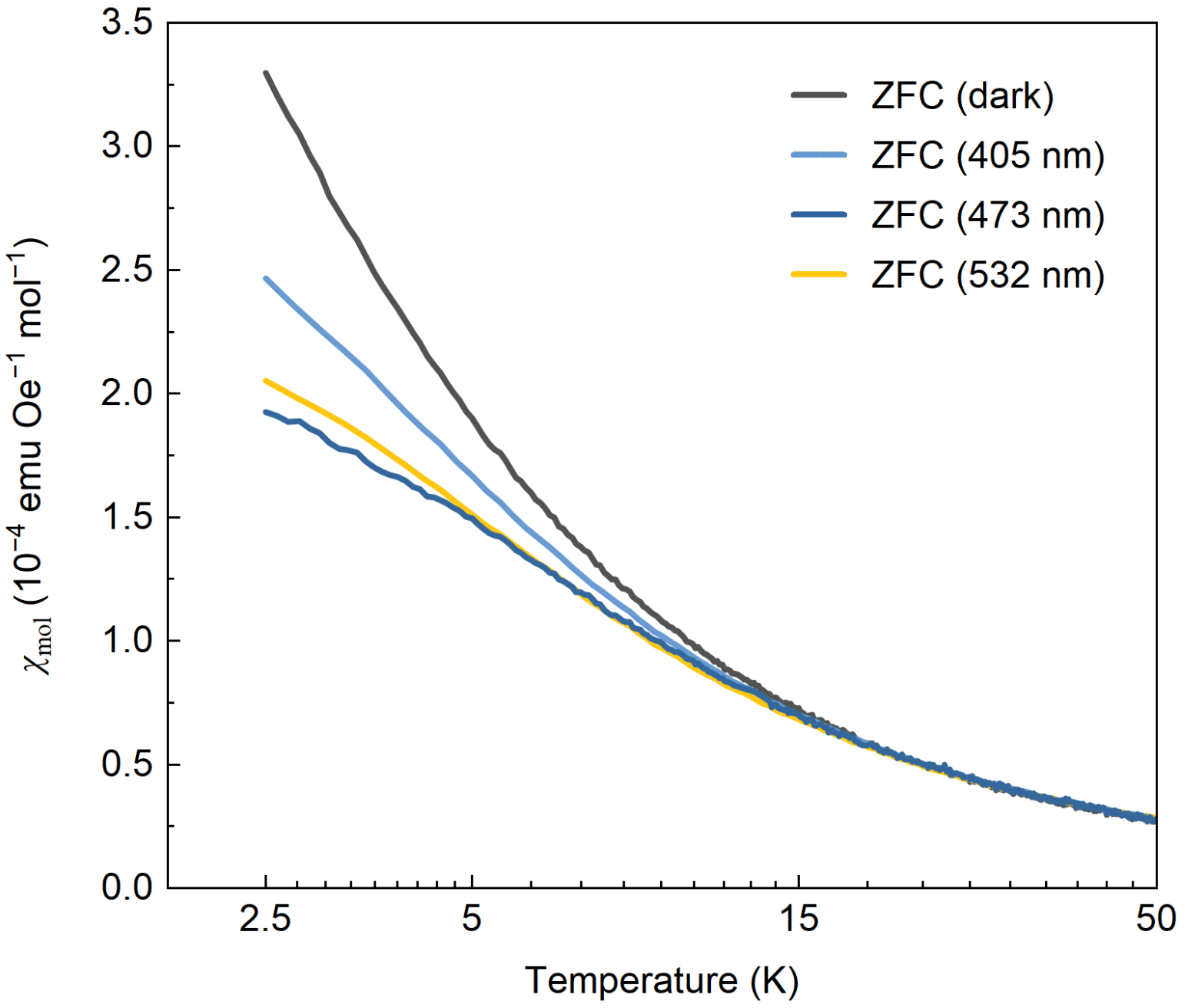

Supplementary Figure 23 | Wavelength-dependent ZFC curves of the alterlattice. Comparison of the ZFC curves of the 0.48 mm-thick sample measured in dark conditions and under 405, 473, and 532 nm illumination. The ZFC curve measured under 473 nm illumination shows the most pronounced decrease compared with the dark-state curve, indicating that 473 nm light induces the strongest antiferromagnetic-like exchange interaction in the alterlattice. This wavelength selectivity supports a photocarrier-mediated mechanism for the formation of the light-induced magnetic response.

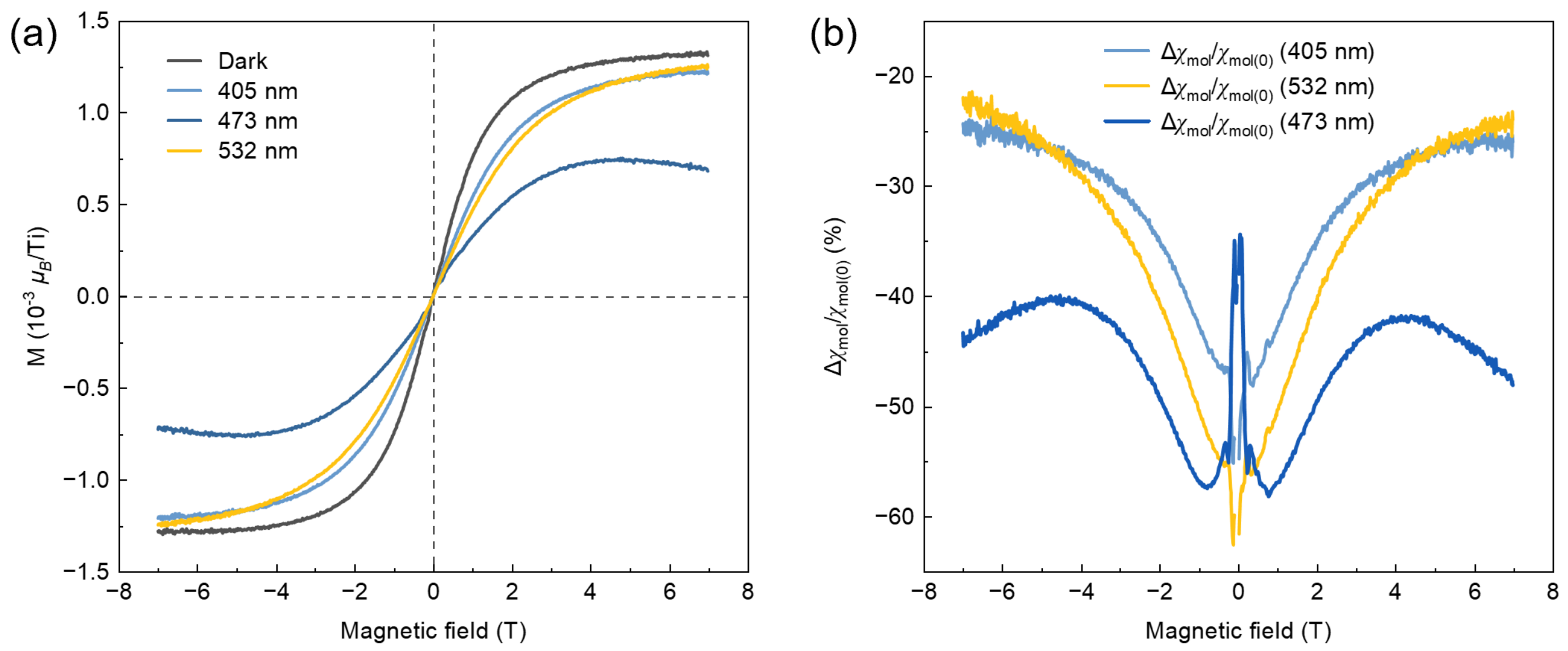

Supplementary Figure 24 | Wavelength-dependent M–H curves of the alterlattice. The results show that 473 nm illumination produces the strongest effect. **(a)** Magnetic-field dependence of magnetization (μ_B/Ti) at 2 K for the 0.48 mm-thick sample under 405, 473, and 532 nm illumination (14.2 mW mm^{-2}), compared with the dark state. **(b)** Field-dependent $\Delta\chi_{mol}/\chi_{mol(0)}$ curves under different illumination wavelengths, including 405, 473, and 532 nm.

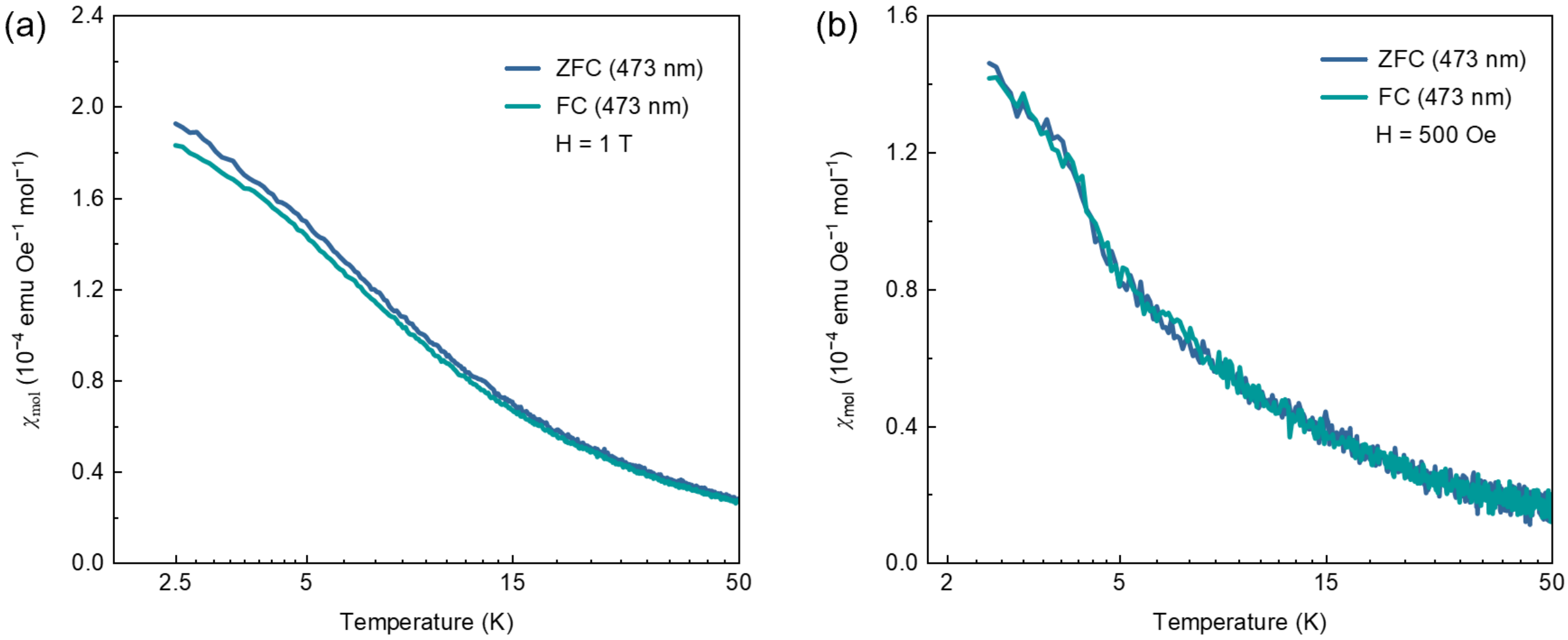

Supplementary Figure 25 | Field dependence of the ZFC–FC bifurcation in the alterlattice ($La_{0.4}Ca_{0.4}TiO_{3-\delta}$) under 473 nm illumination. (a) ZFC and FC magnetic susceptibility of a 0.48 mm $La_{0.4}Ca_{0.4}TiO_{3-\delta}$ sample measured from 2 to 50 K under an applied field of 1 T during continuous illumination at 14.2 mW mm^{-2}. **(b)** ZFC and FC susceptibility of the same sample measured from 2 to 50 K under an applied field of 500 Oe, with the same illumination.

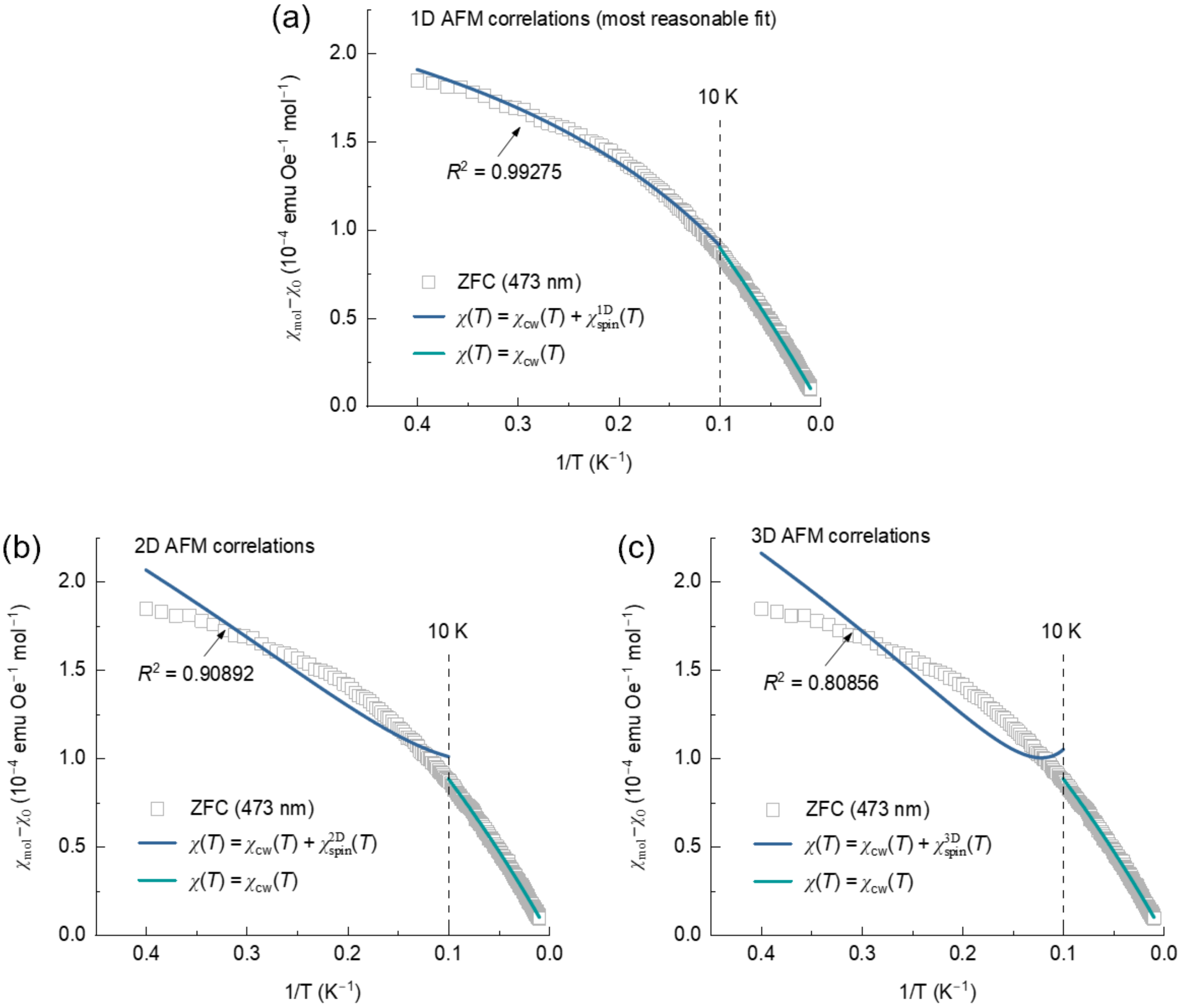

Supplementary Figure 26 | Fitting and analysis of the 473 nm light-induced magnetic response from 2.5 to 10 K using a combined paramagnetic (PM) and antiferromagnetic (AFM) correlation model $\chi(T) = \chi_{cw}(T) + \chi_{spin}(T)$[12,13]. The PM contribution ($\chi_{cw}(T)$) was compared using Curie–Weiss model described by $\chi_{mol}(x) - \chi_0 = Cx/(1-\theta x)$, where x = 1/T, χ_0 is the temperature-independent background susceptibility, C is the Curie constant, θ is the Weiss temperature.The AFM contribution ($\chi_{spin}(T)$) was compared using 1D, 2D, and 3D models described by $\chi_{mol}(x)$ = ax^(1−d/2)e^(−Δx), where x = 1/T, a is the amplitude coefficient of the AFM term, d is the effective excitation dimensionality, and Δ is the characteristic AFM energy gap. Panels **(a)**, **(b)**, and **(c)** correspond to 1D-, 2D-, and 3D-like thermally activated AFM excitations, respectively. The illuminated χ versus 1/T curve was fitted with the model containing the AFM term from 2.5 to 10 K, while the 10–100 K range was fitted using a single Curie–Weiss model. The physically reasonable

fitting parameters indicate that the light-induced magnetic response below 10 K is more consistent with a 1D AFM-like correlation. The small characteristic energy gap suggests a short-range ordered response, likely related to structural confinement from the short-range ordered superstructure domains in the alterlattice.

Supplementary Table 5 | Fitting parameters obtained from Curie–Weiss and *d*-dimensional (*d* = 1, 2, 3) AFM spin-excitation models in Supplementary Figure 26. In the Curie–Weiss term, χ_0 (emu Oe^{-1} mol^{-1}) is the temperature-independent background susceptibility, C (emu K Oe^{-1} mol^{-1}) is the Curie constant, θ (K) is the Weiss temperature. In the gapped low-dimensional AFM excitation term, a (emu Oe^{-1} mol^{-1} $K^{1-d/2}$) is the amplitude coefficient of the AFM term, d is the effective excitation dimensionality, and Δ (K) is the characteristic AFM energy gap.

Model	Fitting range	Fitting parameters			
		χ_0	C	θ	R^2
PM	10–100 K	8×10^{-6}	0.001013	−1.187151	0.998583
		a		Δ	R^2
1D AFM	2.5–10 K	0.000123		1.335813	0.992759
2D AFM	2.5–10 K	0.000123		12.063121	0.908927
3D AFM	2.5–10 K	0.000123		22.806433	0.808562

χ_0 represents a temperature-independent contribution, which may be associated with defect states related to oxygen vacancies or the Pauli paramagnetism of partially delocalized Ti 3*d* electrons.

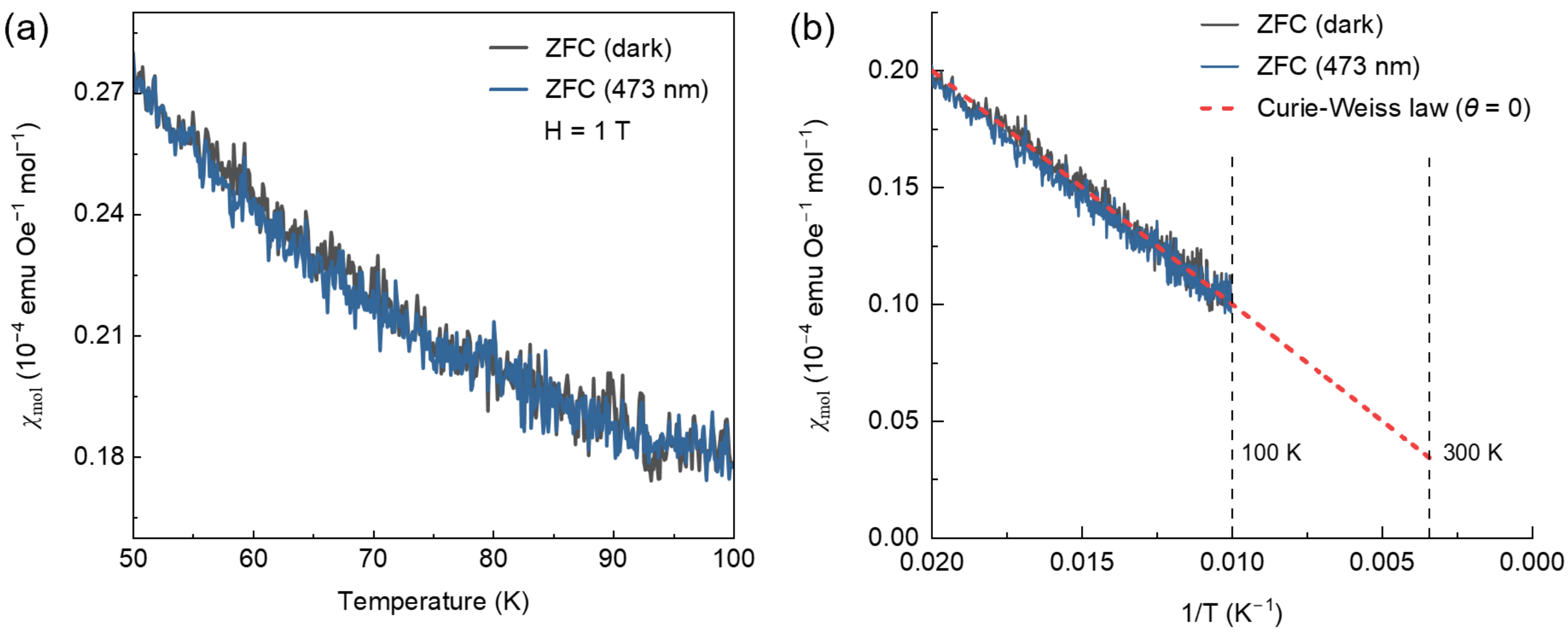

Supplementary Figure 27 | Paramagnetic behavior under illumination in the 50–100 K range. **(a)** Temperature dependence of χ_{mol} for the 0.48 mm thick sample from 50 to 100 K, measured at H = 1 T under dark conditions and 473 nm illumination with a light intensity of 14.2 mW mm^{-2}. The ZFC curves under dark and illuminated conditions nearly overlap, indicating that illumination does not induce obvious magnetic ordering at higher temperatures. **(b)** χ_{mol} versus 1/T analysis showing that both dark and illuminated curves are close to ideal Curie–Weiss behavior with very small Weiss temperatures. This indicates that no pronounced magnetic exchange interaction is present from 50 to 100 K, and that both curves are mainly governed by paramagnetic behavior from dilute magnetic centers.

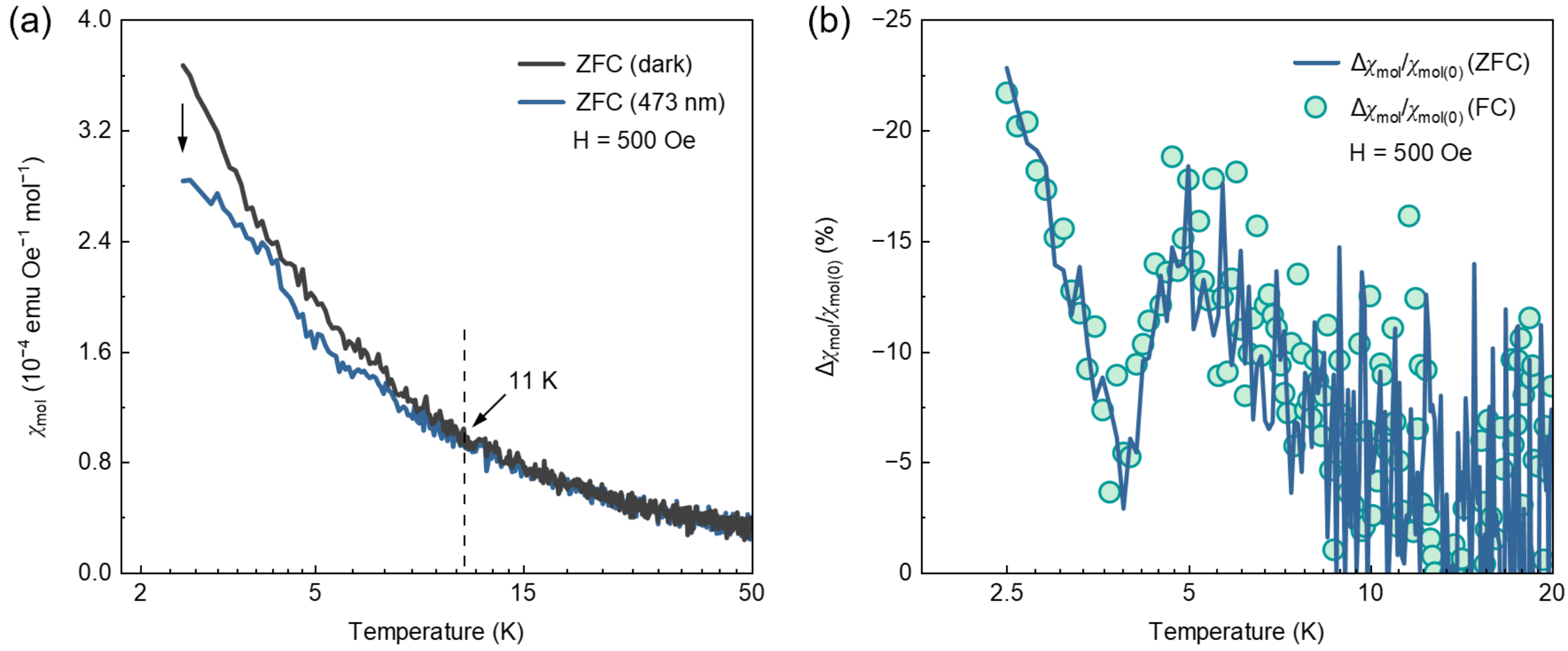

Supplementary Figure 28 | Low-field photomagnetic response of the alterlattice. (a) Temperature dependence of χ_{mol} at 500 Oe under 14.2 mW mm^{-2} illumination versus darkness (d = 0.48 mm sample). **(b)** Temperature dependence of $\Delta\chi_{\mathrm{mol}}/\chi_{\mathrm{mol(0)}}$ at 500 Oe under 14.2 mW mm^{-2} illumination (d = 0.48 mm).

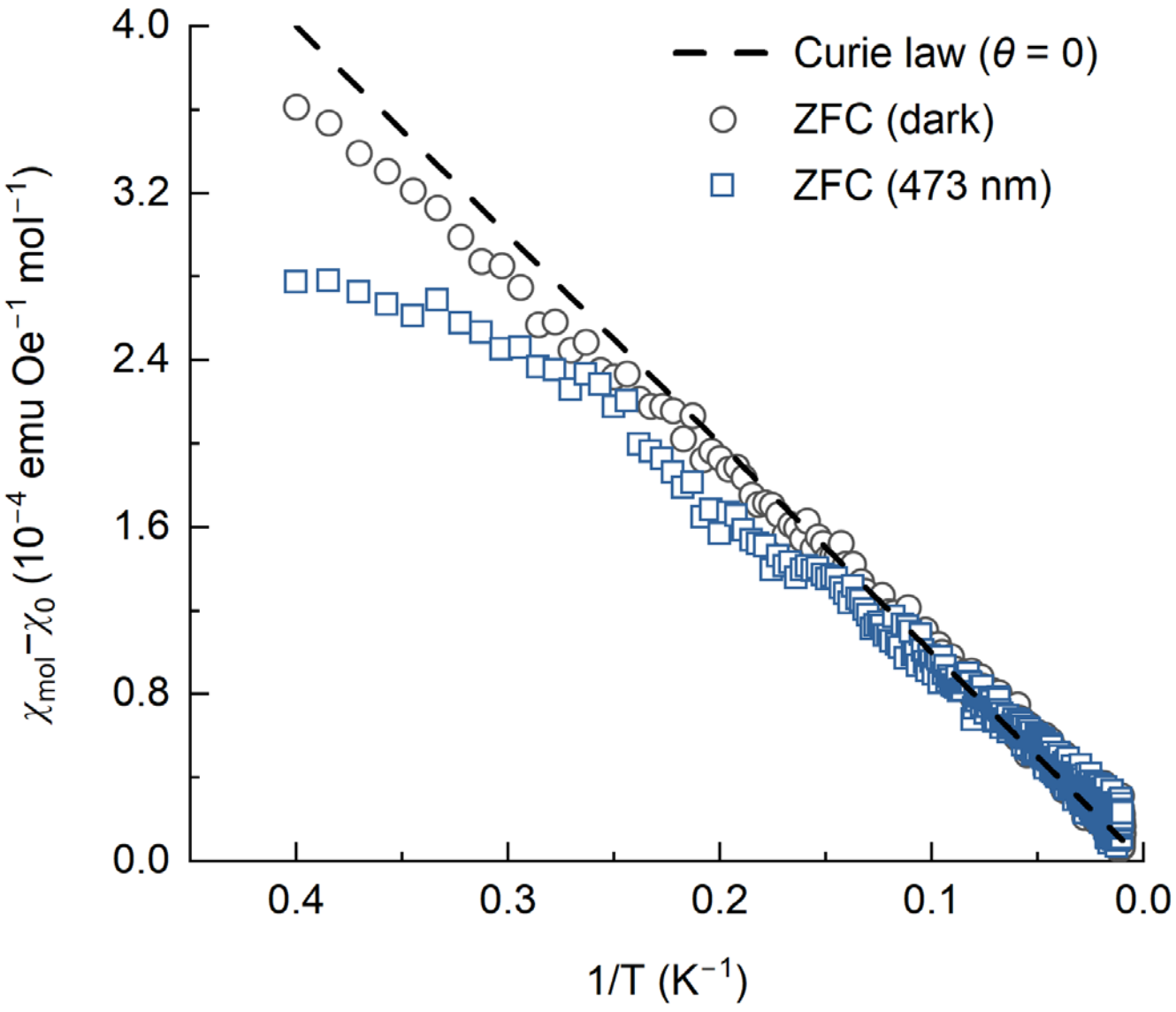

Supplementary Figure 29 | Dependence of χ_{mol}–χ_0 on 1/T in the alterlattice (*d* = 0.48 mm sample). χ_{mol} was measured in ZFC mode at 500 Oe under both illumination (14.2 mW mm^{-2}) and dark conditions. In the Curie law limit ($\theta = 0$), χ_{mol} varies linearly with 1/T, indicating that the magnetism in the dark state is dominated by nearly ideal paramagnetic moments with weak magnetic interactions. The small deviation from linearity observed under illumination indicates a photomagnetic phase modulation. **Note**: $\chi_0 = 5.6\times10^{-6}$ emu Oe^{-1} mol^{-1} represents a temperature-independent contribution, which may be associated with defect states related to oxygen vacancies or the Pauli paramagnetism of partially delocalized Ti 3*d* electrons.

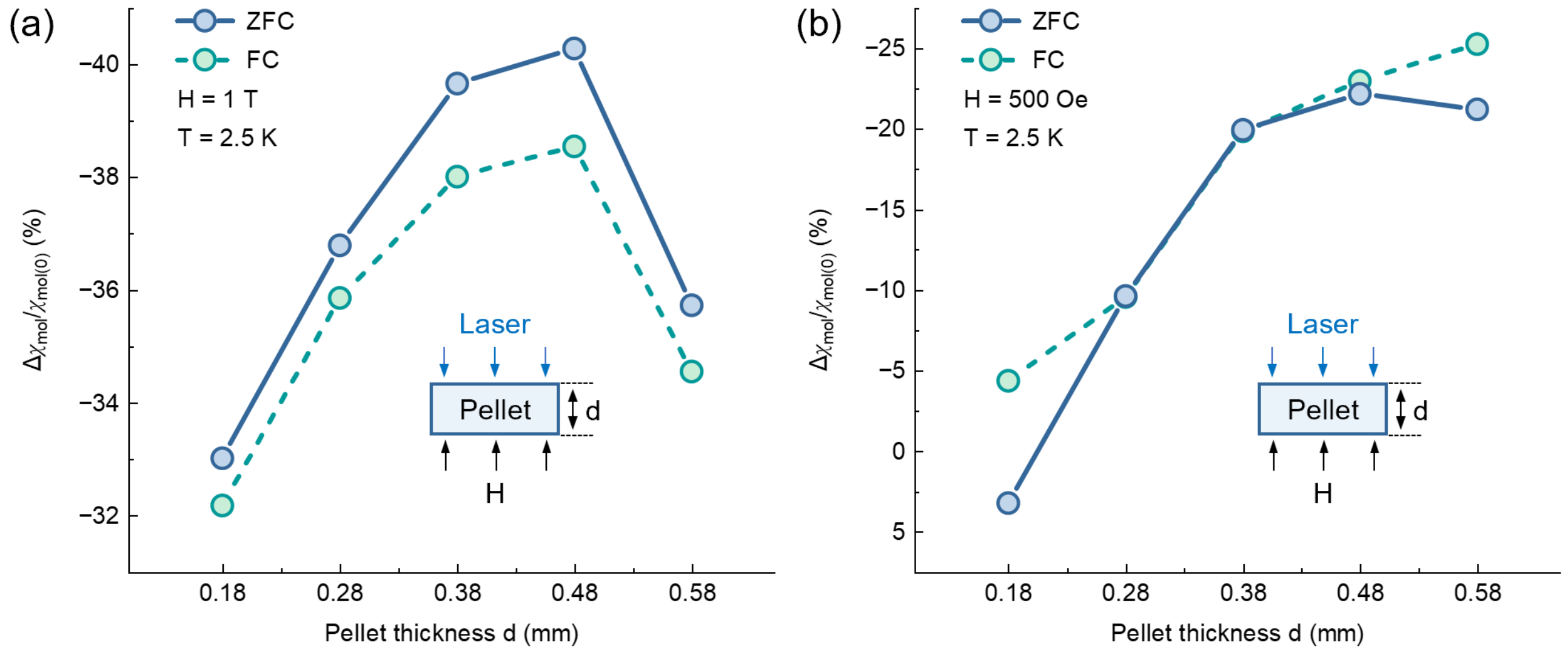

Supplementary Figure 30 | Effect of alterlattice pellet thickness on $\Delta\chi_{mol}/\chi_{mol(0)}$ under an applied field of (a) 1 T and (b) 500 Oe from ZFC and FC data.

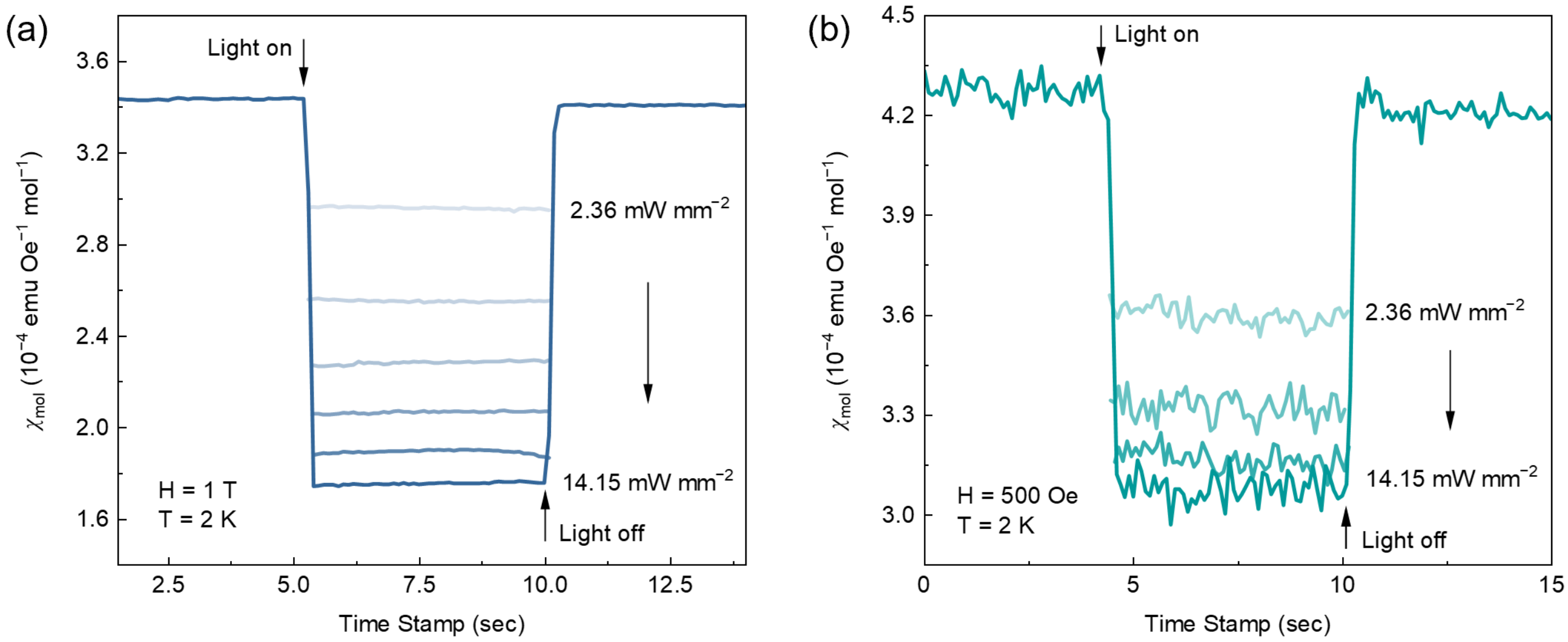

Supplementary Figure 31 | Laser power-density dependence of the optically induced reduction in magnetic susceptibility. Measurements were performed on an $La_{0.4}Ca_{0.4}TiO_{3-\delta}$ (alterlattice) pellet (*d* = 0.48 mm) at T = 2 K under an applied field of **(a)** 1 T and **(b)** 500 Oe. χ_{mol} was recorded while switching the laser on and off at different power densities (listed in the plot).

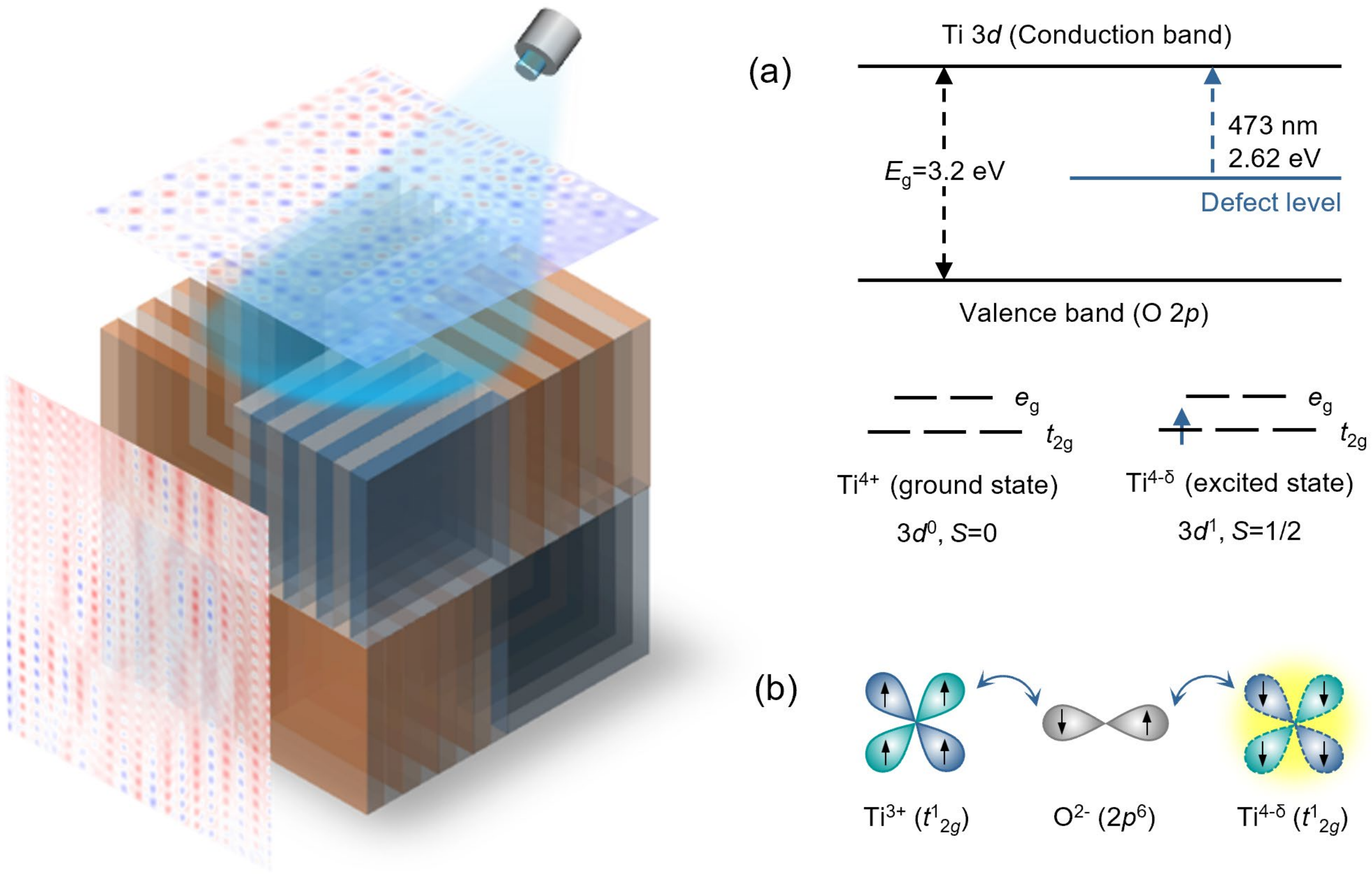

Supplementary Figure 32 | Proposed mechanism for the photocarrier-mediated magnetic response. (a) Defect level excitation, in which electrons are photoexcited from oxygen vacancy related defect levels within the band gap into Ti 3*d* conduction states, followed by local trapping or redistribution that converts nearby Ti^{4+} to Ti^{3+} with unpaired spins. **(b)** Photocarrier transport facilitated by Ti^{3+} "seeds" that promote collective ordering, with an external field assisting to overcome transport barriers.

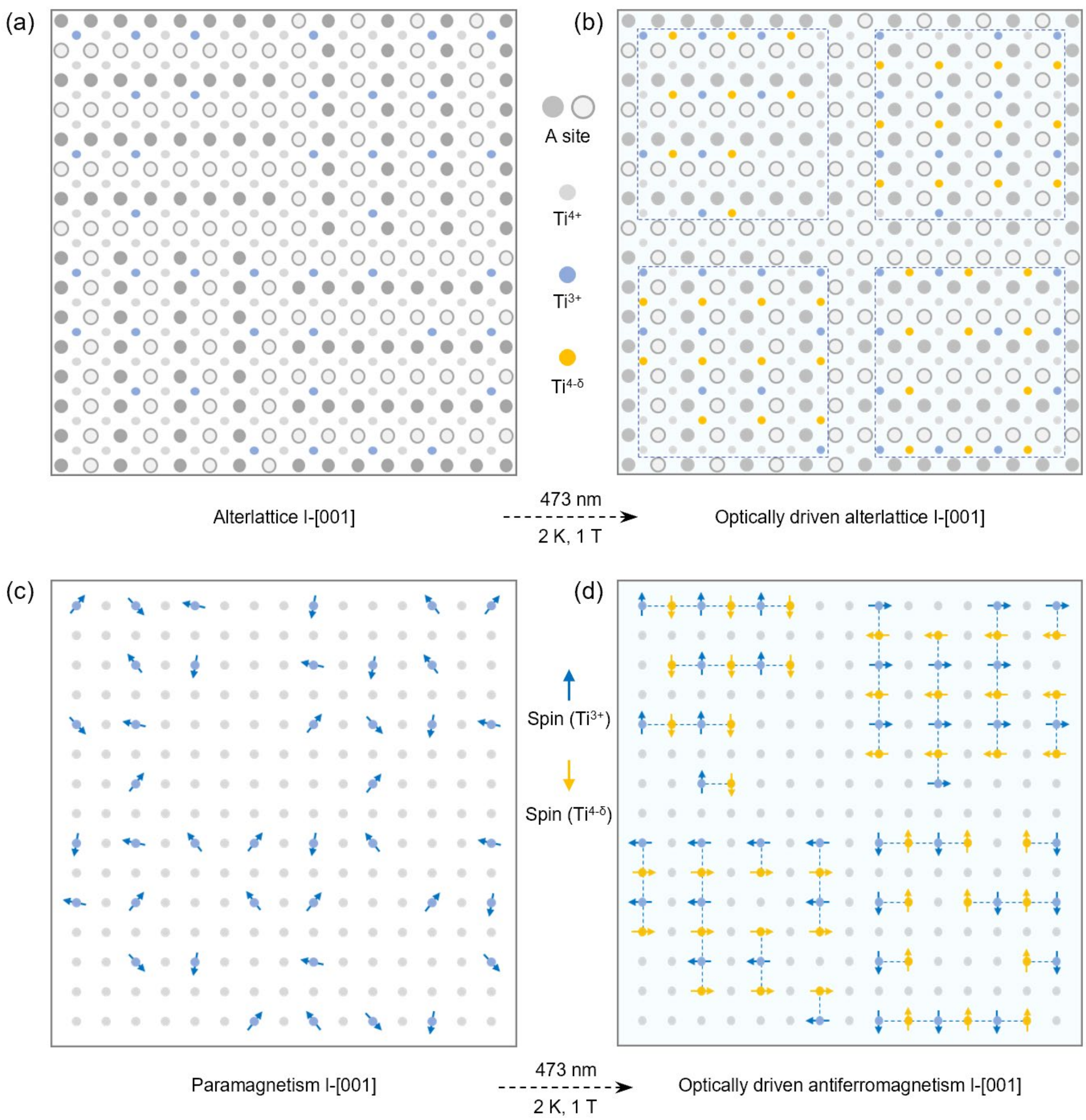

Supplementary Figure 33 | Schematic of short-range AFM order in the alterlattice along the I-[001] zone axis driven by defect-mediated anisotropic photocarrier transport. (a) I-[001] projection in dark-state, showing sparse, disordered Ti^{3+}. **(b)** Enhancement of short-range AFM order under illumination, driven by coupling between photogenerated Ti^{3+} and pre-existing Ti^{3+} in the presence of an applied magnetic field. **(c)** Spin configuration of the paramagnetic ground state along [001], showing that the paramagnetic background is dominated by distributed S = 1/2 spins. **(d)** Anisotropic ordering within orthogonal superstructure domains of the alterlattice. Under geometric constraints, photogenerated carriers and an external magnetic field cooperatively reorganize short-range AFM domains along in-plane orthogonal orientations, which coexist with the host framework. At the macroscopic scale this reorganization produces a reversible, large negative photomagnetic response.

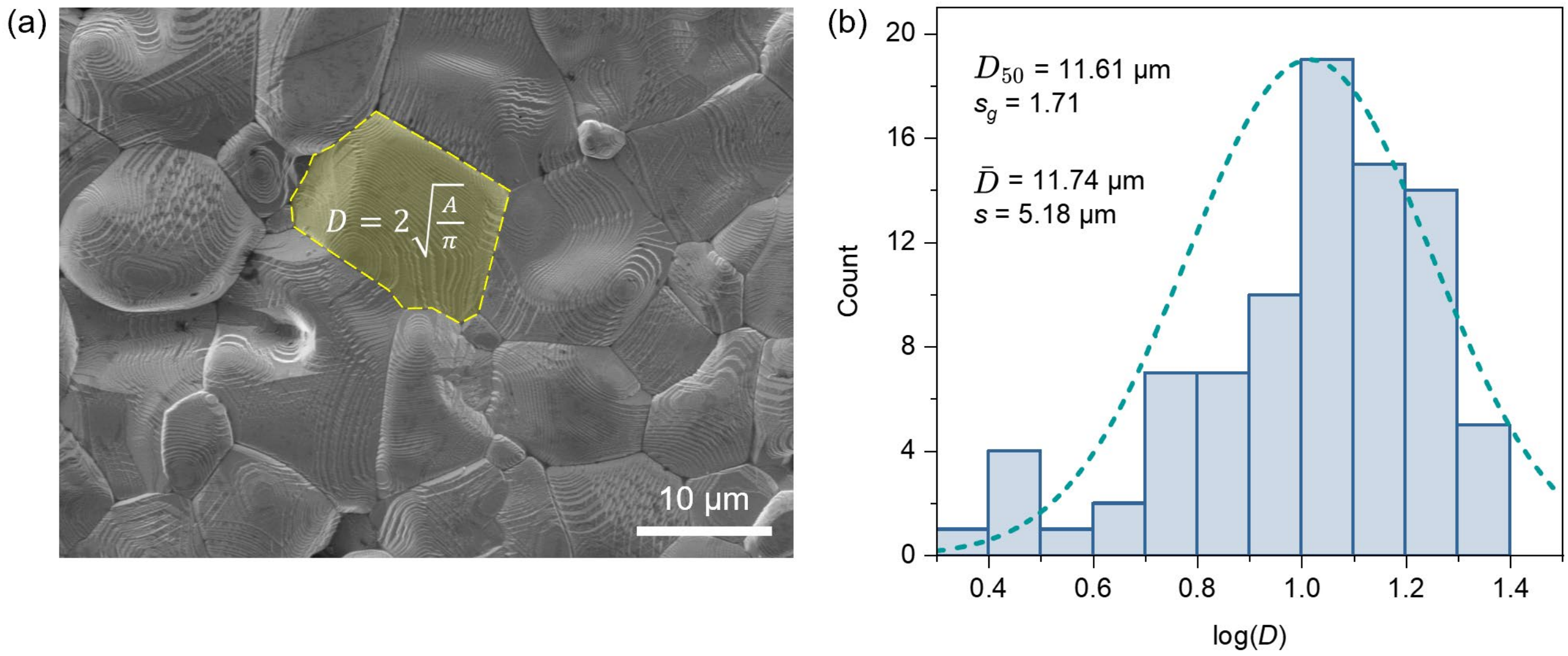

Supplementary Figure 34 | SEM characterization and grain-size distribution analysis of the sintered alterlattice ceramic pellet used for photomagnetic measurements. (a) Low-magnification SEM image of the sintered ceramic pellet showing its dense polycrystalline microstructure. A representative grain is outlined to illustrate the calculation of the equivalent circular diameter using $D = 2(A/\pi)$^1/2, where A is the projected grain area. **(b)** Histogram of the logarithmic grain-size distribution fitted using a log-normal function. The extracted parameters include the median grain size D_{50}, geometric standard deviation σ_g, arithmetic mean grain size D, and standard deviation σ.

We performed statistical grain-size analysis based on low-magnification SEM images. The area of each grain was obtained by image analysis, and the apparent grain size was represented by the equivalent circular diameter $D = 2(A/\pi)$^1/2, where A is the projected grain area[14,15]. This approach is commonly used to estimate two-dimensional grain sizes in ceramic microstructure analysis. Related studies also frequently determine equivalent circular diameters from grain areas in SEM images and describe ceramic grain-size distributions using a log-normal function. The statistical results show that the ceramic pellet is composed of micrometer-sized polycrystalline grains, mainly distributed in the range of approximately 5 to 20 μm. The grain-size distribution can be well described by a log-normal function. The extracted median grain size is $D_{50} = 11.61$ μm, the geometric standard deviation is $\sigma_g = 1.71$, and the arithmetic mean grain size is $D = 11.74 \pm 5.18$ μm. These results indicate a typical sintered polycrystalline ceramic microstructure without obvious abnormal grain growth.

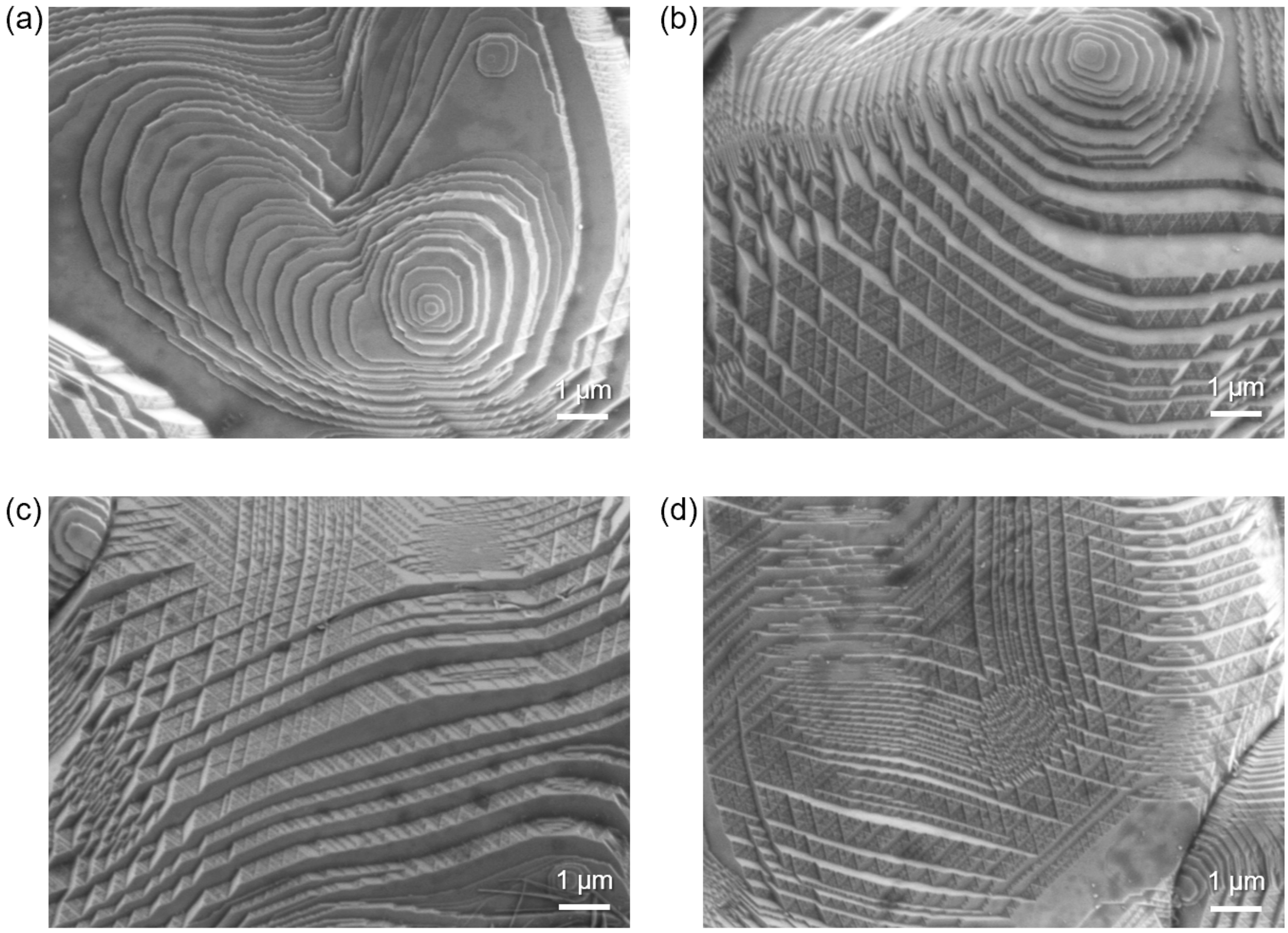

Supplementary Figure 35 | SEM characterization of the step-like surface microstructure on grains of the sintered alterlattice ceramic pellet. (a-d) High-magnification SEM images collected from different grain regions show clear step-like, terrace-like, and ledge-terrace-type surface microstructures on the surfaces of the sintered ceramic grains.

High magnification SEM images further show clear step like, terrace like, and ledge terrace type surface morphologies on the ceramic grains. Similar terraced and faceted native surfaces have been reported in A-site deficient titanate perovskites, especially La and Sr based titanates[16-18], where surface morphology is closely related to oxygen non-stoichiometry and A site defect chemistry. In these systems, oxygen deficient compositions can develop terrace like motifs, indicating structurally and chemically inhomogeneous surface regions. Therefore, the step terrace morphology observed here is likely associated with defect mediated surface reconstruction, facet dependent surface energy minimization, and grain boundary or surface mass transport during high temperature sintering and subsequent cooling.

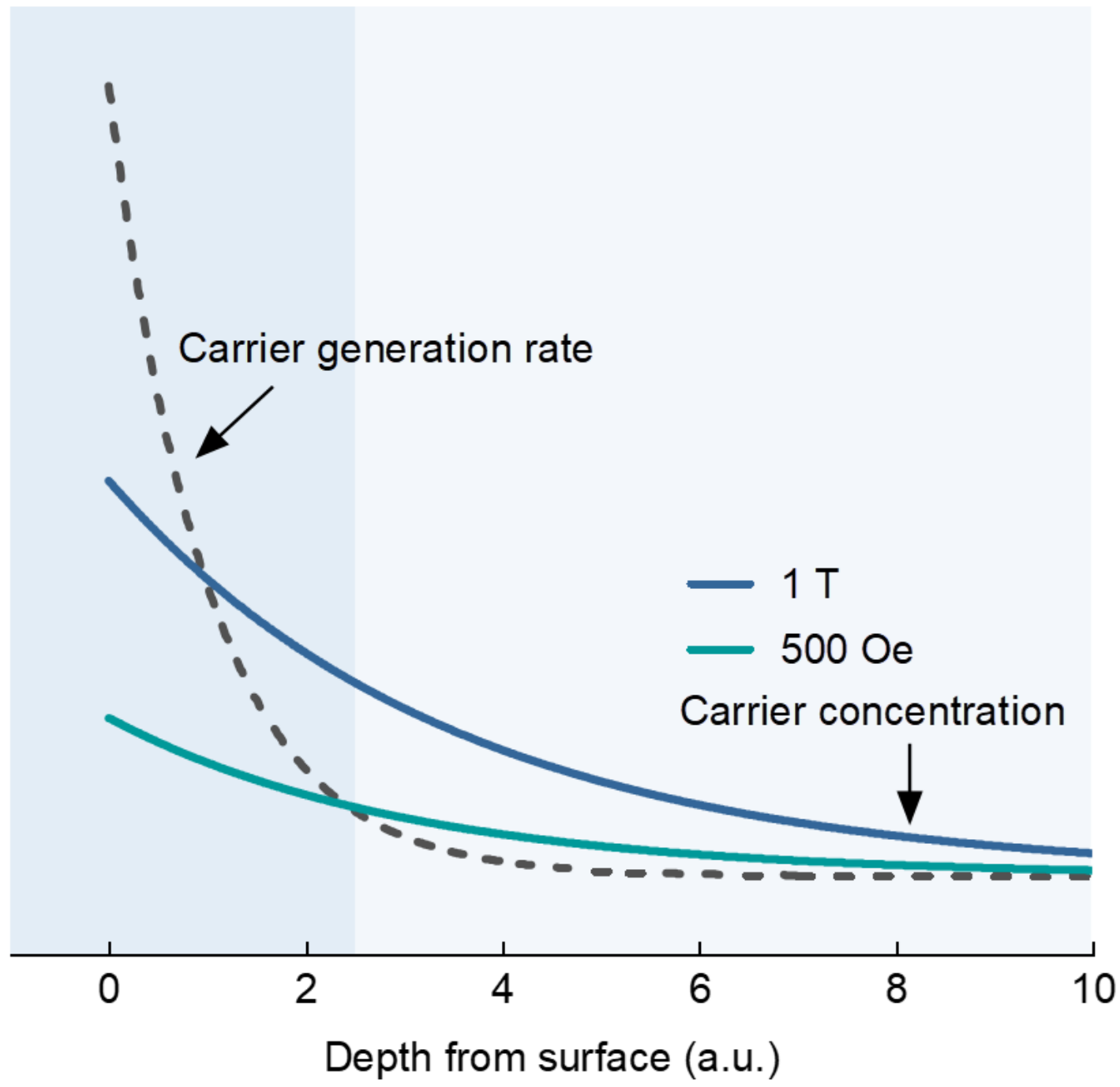

Supplementary Figure 36 | Schematic optical penetration depth and resulting photocarrier profile from the Lambert-Beer law.

The schematic shows that at low temperature long carrier lifetimes and an applied magnetic field extend carrier migration beyond the optical absorption depth. More specifically, when light is incident on a semiconductor surface, the photogeneration rate decays exponentially with depth according to the Lambert-Beer law. The photocarriers diffuse from their point of generation and can be described by the one-dimensional diffusion–recombination equation $\mathrm{D}(\mathrm{d}^2 n/\mathrm{d}x^2) - n/\tau + G(x) = 0$, where $n(x)$ is the photocarrier density, D is the diffusion coefficient, and τ is the carrier lifetime. The characteristic diffusion length $L = \sqrt{(D\tau)}$ quantifies the average distance carriers travel before recombination and sets the length scale over which light-generated carriers mediate local photomagnetic responses. In a strong magnetic field, particularly in the liquid-helium temperature regime and below, magnetotransport effects can substantially modify both D and τ. The field also affects recombination dynamics—for example by altering carrier trajectories, enhancing the spatial separation of electrons and holes, or modifying spin-dependent recombination pathways—thereby extending the effective lifetime. Consequently, in our system a strong external field increases the diffusion length L, enabling photocarriers to sample a larger volume and strengthening the long-range photomagnetic response.

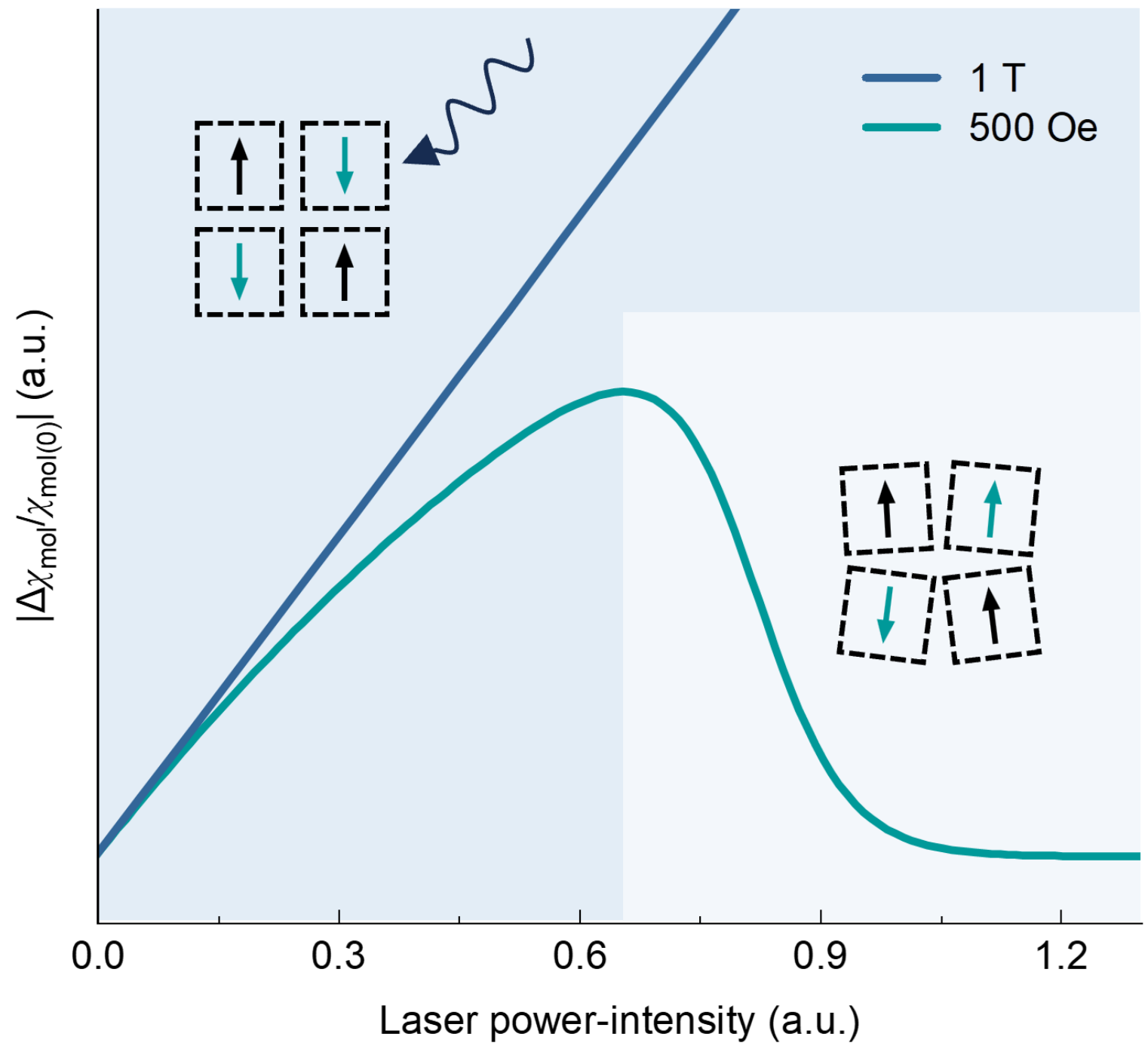

Supplementary Figure 37 | Schematic of the laser power-density dependence of $\Delta\chi_{mol}/\chi_{mol(0)}$. At 1 T, $|\Delta\chi_{mol}/\chi_{mol(0)}|$ increases nearly linearly with increasing laser power density, indicating that the field-stabilized photo-induced magnetic response is progressively enhanced by carrier generation. In contrast, at 500 Oe, $|\Delta\chi_{mol}/\chi_{mol(0)}|$ first increases approximately linearly at low excitation power, approaches saturation at intermediate power, and is then suppressed at high power, consistent with carrier delocalization and disruption of short-range spin order.

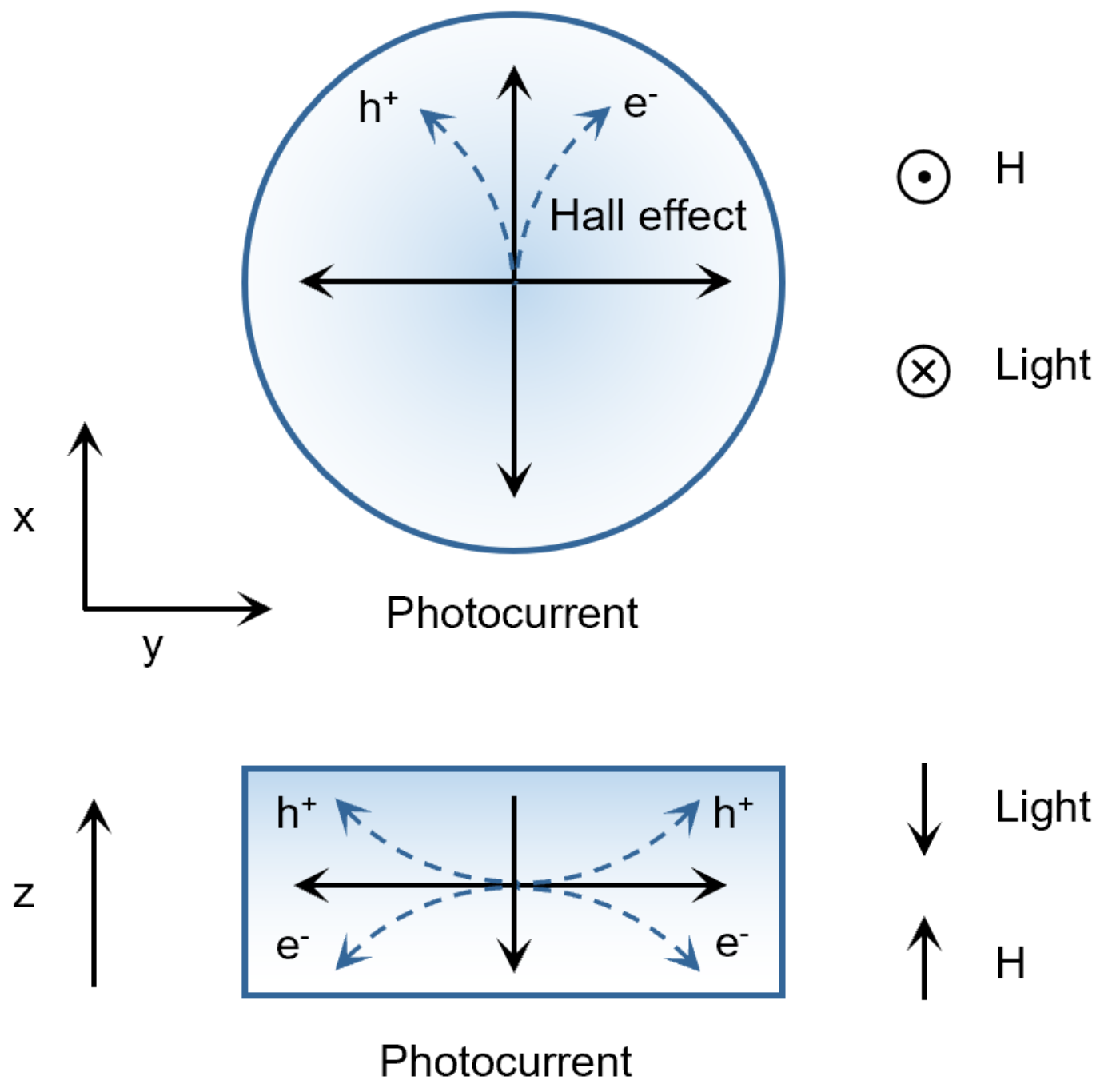

Supplementary Figure 38 | Prolonged carrier lifetimes at low temperature due to reduced scattering and magnetic confinement. A strong external field can further extend the effective carrier lifetime and diffusion length by modifying carrier transport properties. Possible contributions include field-induced reductions in resistivity (negative magnetoresistance), as well as enhanced spatial separation of photoexcited electrons and holes, which carry opposite charge and therefore drift in different directions under the field, suppressing their recombination and thereby strengthening the photomagnetic response.

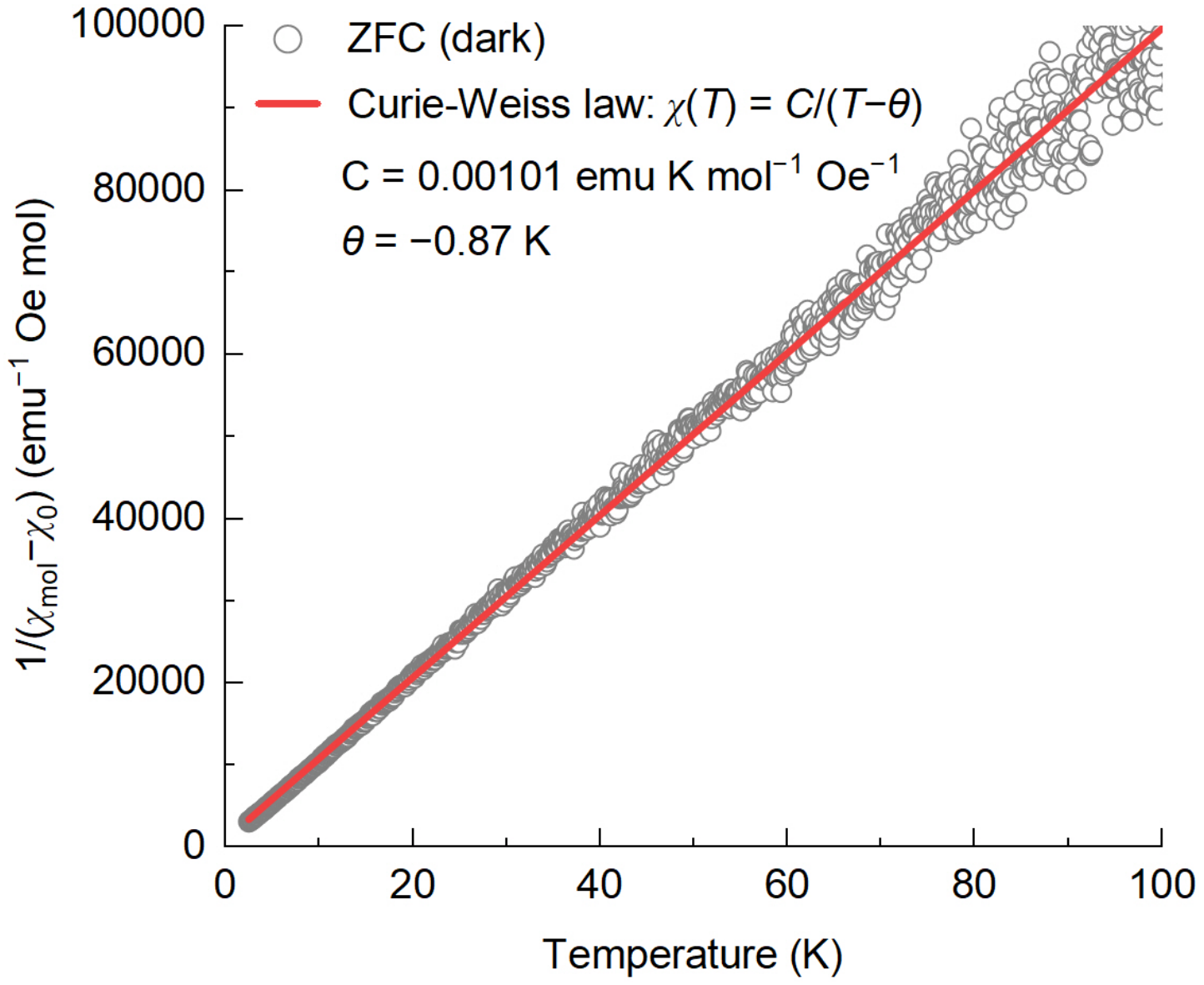

Supplementary Figure 39 | Curie–Weiss fitting of the ZFC inverse susceptibility measured at 1 T in the dark for a $La_{0.4}Ca_{0.4}TiO_{3-\delta}$ ceramic pellet with a diameter of 3 mm and a thickness of 0.48 mm.

The paramagnetically active Ti^{3+} content was estimated by Curie–Weiss fitting in the paramagnetic region, and the corresponding oxygen nonstoichiometry δ was derived from charge neutrality. The susceptibility was fitted using the Curie–Weiss law, $\chi mol(T)-\chi_0 = C/(T-\theta)$, giving $\chi_0 = 7.7\times 10^{-6}$ emu mol^{-1} Oe^{-1}, C = 0.00101 emu K mol^{-1} Oe^{-1} and $\theta = -0.87$ K. The corresponding effective magnetic moment is $\mu_{eff} = 2.828C^{1/2} = 0.090\ \mu_B$ per formula unit. In this system, Ca^{2+}, La^{3+}, Ti^{4+}, and O^{2-} are diamagnetic or closed-shell ions. The main paramagnetic contribution is therefore expected to arise from localized $3d^1$ electrons of Ti^{3+}. Assuming that the local magnetic moments mainly originate from Ti^{3+} and that Ti^{3+} can be approximated as $S = 1/2$ with $g \approx 2$, the effective moment of one isolated Ti^{3+} ion is $\mu_{eff}(Ti^{3+}) = g[S(S+1)]^{1/2} = 1.73\ \mu_B$. The estimated Ti^{3+} fraction is therefore $f(Ti^{3+}) = (0.090/1.73)^2 \approx 2.7 \times 10^{-3}$, corresponding to ~0.27% of Ti sites. Based on charge neutrality for $La_{0.4}Ca_{0.4}TiO_{3-\delta}$, $f(Ti^{3+}) = 2\delta$, giving $\delta \approx 1.4 \times 10^{-3}$.

References

1. Rice WD, *et al.* Persistent optically induced magnetism in oxygen-deficient strontium titanate. *Nature materials* **13**, 481-487 (2014).

2. Disa AS, *et al.* Photo-induced high-temperature ferromagnetism in $YTiO_3$. *Nature* **617**, 73-78 (2023).

3. Bagri A, *et al.* Light-Controlled Magnetoelastic Effects in Ni/$BaTiO_3$ Heterostructures. *ACS Applied Materials & Interfaces* **15**, 18391-18401 (2023).

4. Zhao BC, *et al.* Photomagnetic effect in a single crystal of the bilayer manganite $La_{1.2}Sr_{1.8}Mn_2O_7$. *Physical Review B* **72**, 012414 (2005).

5. Matsubara M, *et al.* Ultrafast photoinduced insulator-ferromagnet transition in the perovskite manganite $Gd_{0.55}Sr_{0.45}MnO_3$. *Phys Rev Lett* **99**, 207401 (2007).

6. Li F, *et al.* Optical control of magnetism in manganite films. *Physical Review B* **93**, 024406 (2016).

7. Muraoka Y, Tabata H, Kawai T. Effects of light-irradiation on spin-glass state and magnetoresistive properties of $Zn_{0.5}Co_{0.5}Fe_2O_4$ spinel ferrite films. *Solid State Communications* **120**, 255-258 (2001).

8. Zhang Q, *et al.* CalAtom: A software for quantitatively analysing atomic columns in a transmission electron microscope image. *Ultramicroscopy* **202**, 114-120 (2019).

9. Hillyard S, Silcox J. Detector geometry, thermal diffuse scattering and strain effects in ADF STEM imaging. *Ultramicroscopy* **58**, 6-17 (1995).

10. Combs AH, *et al.* Fast approximate STEM image simulations from a machine learning model. *Advanced Structural and Chemical Imaging* **5**, 2 (2019).

11. Walther T. A new experimental procedure to quantify annular dark field images in scanning transmission electron microscopy. *Journal of Microscopy* **221**, 137-144 (2006).

12. Troyer M, Tsunetsugu H, Würtz D. Thermodynamics and spin gap of the Heisenberg ladder calculated by the look-ahead Lanczos algorithm. *Physical Review B* **50**, 13515-13527 (1994).

13. He Z, Kyômen T, Itoh M. $BaCu_2V_2O_8$: Quasi-one-dimensional alternating chain compound with a large spin gap. *Physical Review B* **69**, 220407 (2004).

14. Syha M, *et al.* Three-dimensional grain structure of sintered bulk strontium titanate from X-ray diffraction contrast tomography. *Scripta Materialia* **66**, 1-4 (2012).

15. Coutinho Y, Rooney S, Payton E. Analysis of EBSD grain size measurements using microstructure simulations and a customizable pattern matching library for grain perimeter estimation. *Metallurgical and Materials Transactions A* **48**, 2375-2395 (2017).

16. Neagu D, *et al.* In situ growth of nanoparticles through control of non-stoichiometry. *Nature*

Chemistry **5**, 916-923 (2013).

17. Neagu D*, et al.* Nano-socketed nickel particles with enhanced coking resistance grown in situ by redox exsolution. *Nature Communications* **6**, 8120 (2015).

18. O'Leary W*, et al.* Influence of Sr-Site Deficiency, Ca/Ba/La Doping on the Exsolution of Ni from $SrTiO_3$. *Journal of the American Chemical Society* **145**, 13768-13779 (2023).
