# Supplementary material for: Giant negative photomagnetism in an alterlattice
Source: Nat Commun. 2026 Aug 7;17:9522. doi: 10.1038/s41467-026-76523-2 (PMC13547212; doi:10.1038/s41467-026-76523-2)
Supplement: Supplementary file 2 [file 41467_2026_76523_MOESM2_ESM.pdf]

nature portfolio

Peer Review File

Giant Negative Photomagnetism in an Alterlattice

Corresponding Author: Professor Guangshe Li

This file contains all reviewer reports in order by version, followed by all author rebuttals in order by version.

Version 0:

Reviewer comments:

Reviewer #1

(Remarks to the Author)
This manuscript reports in-depth investigation about an A-site deficient perovskite material with nanoscale staggered structural domains. The authors attribute a photo-induced magnetization decrease of this material to a mechanism of photo-carrier induced antiferromagnetic correlation. Detailed characterization of structural and macroscopic magnetic properties is included. I will first describe some general comments, then mention some smaller advices and minor shortages, errors or typos.

General comments:
1. The overall logic of the manuscript seems not satisfactory. The authors conducted very detailed structural analysis, which occupies a large portion of the manuscript. But my feeling is that the structural characterization and the magnetic properties are weekly connected. The light-induced magnetization change is attributed to photocarriers, but this is not necessarily related to such “alterlattice” structure. The word “design strategy” is mentioned (line 99), but I cannot be convinced that the photo-induced demagnetization can be intentionally “designed” by growing such a sample. I also do not like the “potential application” in “spin-based quantum information technologies”. Although Fig.S31 is fancy enough, it does not provide any useful information, for example how to realize such application by the present material.
2. The structure of the manuscript need to be improved. After the introduction part, the manuscript keeps on describing experimental results with very little analysis. And then in the discussion part, the manuscript starts to recap the previous descriptions before in-depth discussions, which is not efficient at all. The authors should consider to reorganize the whole manuscript and try to merge more discussion into the experimental part, and describe more about the motivation to do this or that measurement.
3. In the part discussing about the photo-induced magnetic properties, I feel it difficult to follow since comparison between ZFC/FC and light-on/off are not described with enough order. χm, moment and $1/\chi mol$, etc. are plotted without explanation of why to plot like that. Why not using $1/\chi m$ instead of $1/\chi mol$? And in Fig.S19 a similar figure is shown, but with a log scale, which is not consistent with Fig.4.
4. The mechanism of photo-induced AFM seems to be a hypothesis, but not a solid confirmation. The authors showed deviation from Curie-Weiss scaling in Fig.4e, but the high temperature part of the $1/\chi$-H curve of an AFM material should also follow Curie-Weiss scaling but with a negative intersection with the $1/\chi$ axis, which seems not the case in Fig.4e.

Smaller advices:
1. The authors mainly discussed about symmetries of Ibmm, Cmmm, or Pmmm, which all belong to orthorhombic crystal system. However, at some place tetragonal phase is also mentioned. It should be explained that why tetragonal phase could not be observed at an orthorhombic-tetragonal phase boundary.
2. In line 119, “multi-q superstructure” is mentioned, with reference of XRD refinement results. I can only see that the refinement results are good, but it is not clear at all what is this superstructure, for example what are the “multi” q values.
3. In Fig. S2b, I cannot recommend such plot with atom dots on the TEM images. The TEM image seems just a square lattice and there is no sign of alternating two-atom and one-atom arrays, as shown by the red dots. Such figure is misleading and does not provide any useful information, since you can also overlap a simple square lattice or some other similar lattice to the TEM image.
4. “Local fluctuations in La and O concentrations” is mentioned, but in Fig.S3 it seems to me not so obvious. One can never observe identical atom intensity in TEM images and there are always some intensity fluctuations due to statistics or sample preparation, etc.
5. I suggest Fig.4h be replaced by some fluence dependence of the demagnetization effect, such as Fig. S23. The present figure shows similar information as Fig.4b and can be put into the supplementary material.

6. In Fig.S15a, why a part of the blue curve is missing?
7. It is shown in Fig. S16 “ZFC/FC (dark)” magnetization curve, but the measurement results of ZFC and FC could not be exactly identical. Please show both curves. And it is mentioned that “these $\Delta\chi m/\chi m(0)$ were obtained by nonlinear fitting to reduce noise”(line 256). I do not understand how to reduce noise with a mathematic fitting and see no physical meaning of the fitting parameters in Fig. S16.
8. It is mentioned in line 338 that “the Ti L2,3 edges shift to lower energy and the L2 t2g-split shoulder is enhanced”. But the shift seems just fluctuation of noise in Fig.S24d. Please provide data with better statistics.
9. The mechanism proposed in Fig.S25 seems not convincing enough. The excitation from valence band to defect levels and then from defect levels to the conduction band may have less possibility than direct excitation from valence band to conduction band. In this sense, wavelength dependence of the photo-induced demagnetization should be conducted to verify the proposed mechanism.

Minor shortages, errors or typos:
1. There are some abbreviations which are not clearly explained, which may be not obvious to ones who are not familiar with the techniques or terminologies. For example, “Obs.” and “Ni I. S.” in Fig.1d, Reduced $\chi 2$, wR, and GOF in Table S1,2, etc.
2. In Fig.1b, I do not understand why the Ca/La atomic ratio should be plotted by log scale.
3. “Dual-q” and “double-q” are both used, which I recommend to unify. some q’s are not Italic.
4. Sentence error in line 206.
5. Line 282, duplicate words.
6. Line 291~292, grammar error.
7. Fig.4f, the unit of moment seems to be wrong. Can the moment be converted to μB/Ti to give a impression of the local moment size?

Thus, I cannot recommend publication of this manuscript before major revision and proper response to the abovementioned points.

Reviewer #2

(Remarks to the Author)
In this work, the authors present an emergent structural motif referred to as the alterlattice, which manifests photoinduced short-range antiferromagnetic correlations below 19 K and is associated with two critical magnetic fields. The authors prepared $La_{0.4}Ca_{0.4}TiO_{3-\delta}$ with the well known solid state reaction. The alterlattice here shown to act as a structured amplifier of photoinduced magnetism and provides a distinct materials pathway toward next-generation spin-based quantum information technology. The article needs some modifications to be published in Nat. communications.
1- Some language, typos and grammar issues need to be revised
2-Comparison with reported literature concerning miscellaneous perovskites and/ or titanates must be cited.
3- Is there any estimation of the crystallite size, morphology/ microstructure, please add if available
4- Is there any evidence based data concerning the existence of Ti^{3+} ions, qualitative or quantitative analysis. We suggest to perform XPS for the obtained sample to find out binding energies of both Ti^{4+} and Ti^{3+}
5- It is also suggested to carry out a quantitative analysis to find out the exact oxygen stoichiometry as it plays a critical role in controlling the magnetic exchange interactions in AFM.
6- The field-dependent photo-response exhibits two characteristic fields at approximately 0.78 T and 4.24 T. This part needs extensive explanation.
7-Is there any correlation between Ti^{4+}-O-Ti^{3+} chemical bond, photoinduced negative magnetism and magnetooptical properties of such perovskite??? if there is please discuss.
8-rewrite the conclusion

Reviewer #3

(Remarks to the Author)
The authors report a so-called “alterlattice” structure with giant negative photomagnetic effect. The authors present many high-quality HAADF-STEM images as well as detailed structural analyses, and the properties are also noteworthy. However, there are several issues need to be carefully addressed before the paper can be accepted for publication.

1. The description of synthesis control is insufficient. The authors should elaborate on the factors that give rise to this structure and on how the photomagnetic response varies among the different phases. Such details would greatly clarify the structure–property relationships.

2. In Fig. 1a, the authors show an idealized schematic of a 3D network composed of orthorhombic short-range domains. This schematic drawing is overly idealized, the actual intergrowth domains are more disordered. I suggest revising the schematic to better reflect the actual arrangement.

3. The authors indexed the structure as the Ibmm phase, and regard the additional diffraction spots from superstructures. The existence of the Ibmm structure was mainly confirmed by XRD analysis (Fig. 1d). However, the diffraction peak positions corresponding to the Cmmm structure are annotated in Fig. 1c but not in Fig. 1d. Would the 90° rotational intergrowth of Cmmm also give rise to diffraction peaks similar to those in Fig. 1d? Further superstructure construction and simulation are needed to verify this point.

4. In Figs. 2a and 2g the entire structure appears to display contrast features corresponding to Cmmm, especially in the thin regions at the right edge of Fig. 2a and the bottom edge of Fig. 2g. By extracting intensity information from these thin regions, one could confirm the atomic count within the linear atom number-contrast regime and provide a more quantitative description of the intergrowth structure. Combining different projection directions would also help. Perhaps ptychography could be employed to reveal the 3D structure, even though the z-resolution is much lower.

5. The insets in Fig. 2a and 2g do not correspond with the selected areas in the original images.

6. In Fig. 3d, did the authors simply overlay three simulated SAED patterns? Do the circles indicate the same d-values? If so, the camera length for the Ibmm [001] pattern appears inconsistent with the two left pattern.

7. Lattice/basis fluctuations can be observed at the bottom of Fig. 3b, the top-left corner and top-middle of Fig. 3f, and the middle of Fig. S3. Do these distortions affect the photomagnetic properties of the material?

8. Some figures in the main text and captions only present their conclusions. The authors are suggested to providing detailed explanations for each figure to improve readability.

9. The TEM sample preparation method and imaging conditions (e.g., convergence angle, camera length, etc.) are not provided. These conditions are essential for interpreting image contrast.

Version 1:

Reviewer comments:

Reviewer #1

(Remarks to the Author)
The authors have seriously replied to all of my questions. I also found the manuscript itself greatly improved. I think that the present manuscript has elucidated the alterlattice strucuture, the photo-induced demagnetization, as well as the direct but phoenomenalogical connection between them. These results are worth publishing. However, I still think that the connection between the alterlattice structure and the photo-induced demagnetization somewhat lacks theoretical base and deep physics. The mechanism that the authors proposed could be one explanation, but not necessarily be it. So I just express my opinion here and leave the final decision to the editor. It is ok to make any decision on this manuscript without contacting me again.
A few small comments:
Figure S5 is not in the table of contents of the supplementary materials.
Some AFM models, such as 1D,2d models, were mentioned without refering to any literatures. The readers may not be familiar with these models.

Reviewer #2

(Remarks to the Author)
The article is now ready to be accepted for publication in Nat.Communications

Reviewer #3

(Remarks to the Author)
All my concerns have been addressed by the authors. I have no further comments and recommend acceptance of this paper in the current form.

Open Access This Peer Review File is licensed under a Creative Commons Attribution 4.0 International License, which permits use, sharing, adaptation, distribution and reproduction in any medium or format, as long as you give appropriate credit to the original author(s) and the source, provide a link to the Creative Commons license, and indicate if changes were made.
In cases where reviewers are anonymous, credit should be given to 'Anonymous Referee' and the source.
The images or other third party material in this Peer Review File are included in the article's Creative Commons license, unless indicated otherwise in a credit line to the material. If material is not included in the article's Creative Commons license and your intended use is not permitted by statutory regulation or exceeds the permitted use, you will need to obtain permission directly from the copyright holder.
To view a copy of this license, visit https://creativecommons.org/licenses/by/4.0/

Point-by-point responses to the Reviewers

Dear reviewers,

We sincerely thank the reviewers for their valuable time, careful evaluation, and insightful comments on our manuscript entitled "*Giant Negative Photomagnetism in an Alterlattice*" (Full Paper, NCOMMS-25-70275-T). We have completed the invited revision and carefully addressed each comment in a point-by-point manner.

In the response letter, ***the reviewers' comments are shown in blue italics***, and **our responses are presented in black text**. All revisions in the manuscript and the Supporting Information have been highlighted in blue for clarity.

We hope that the revised manuscript adequately addresses the reviewers' concerns and is now more accessible and convincing for readers. Our detailed point-by-point responses are provided below, followed by a summary of the major changes made in the revised manuscript and Supporting Information.

Sincerely,

Guangshe Li and Liping Li
On behalf of all authors

Contents

I. Reviewer #1 (Remarks to the Author) ... 3

Comments 1.1 ... 3

Comments 1.2 ... 3

Comments 1.3 ... 5

Comments 1.4 ... 6

Comments 1.5 ... 7

Comments 1.6 ... 9

Comments 1.7 ... 10

Comments 1.8 ... 12

Comments 1.9 ... 12

Comments 1.10 ... 13

Comments 1.11 ... 13

Comments 1.12 ... 14

Comments 1.13: ... 16

Comments 1.14: ... 18

Comments 1.15-1.22 ... 20

II. Reviewer #2 (Remarks to the Author) ... 23

Comments 2.1 ... 23

Comments 2.2 ... 23

Comments 2.3 ... 23

Comments 2.4: ... 24

Comments 2.5 ... 25

Comments 2.6 ... 27

Comments 2.7 ... 27

Comments 2.8 ... 28

Comments 2.9 ... 28

III Reviewer #3 (Remarks to the Author) ... 30

Comments 3.1 ... 30

Comments 3.2 ... 30

Comments 3.3 ... 32

Comments 3.4 ... 32

Comments 3.5 ... 36

Comments 3.6 ... 38

Comments 3.7 ... 39

Comments 3.8 ... 40

Comments 3.9 ... 40

Comments 3.10 ... 41

I. Reviewer #1 (Remarks to the Author)

Comments 1.1: "This manuscript reports in-depth investigation about an A-site deficient perovskite material with nanoscale staggered structural domains. The authors attribute a photo-induced magnetization decrease of this material to a mechanism of photo-carrier induced antiferromagnetic correlation. Detailed characterization of structural and macroscopic magnetic properties is included. I will first describe some general comments, then mention some smaller advices and minor shortages, errors or typos."

Response 1.1: We thank the reviewer for the positive evaluation of this work. Following the reviewer's suggestions, we have improved the overall organization and presentation of the manuscript and provide point-by-point responses below. The reviewer's comments have helped us improve the clarity and scientific rigor of the manuscript.

General comments:

Comments 1.2: "The overall logic of the manuscript seems not satisfactory. The authors conducted very detailed structural analysis, which occupies a large portion of the manuscript. But my feeling is that the structural characterization and the magnetic properties are weekly connected. The light-induced magnetization change is attributed to photocarriers, but this is not necessarily related to such "alterlattice" structure. The word "design strategy" is mentioned (line 99), but I cannot be convinced that the photo-induced demagnetization can be intentionally "designed" by growing such a sample. I also do not like the "potential application" in "spin-based quantum information technologies". Although Fig.S31 is fancy enough, it does not provide any useful information, for example how to realize such application by the present material."

Response 1.2: We thank the reviewer for this critical comment. In the original manuscript, the structural characterization was presented in detail, but the connection between the alterlattice structure and the observed photomagnetic response was not stated clearly enough. We have revised the manuscript by reorganizing the "The alterlattice design principles" section, clarifying the relationship between structure and magnetic response, and moderating the application-oriented claims.

(i) We have refocused the "The alterlattice design principles" section on structural features directly relevant to the photomagnetic response (the revised Fig. 1b–d). We compared the $La_{2(1-x)/3}Ca_xTiO_3$ series with $x = 0$, 0.2, 0.4, 0.7, and 1.0 (the added Fig. R1 and the revised manuscript, lines 130–139), supporting how the alterlattice ($x = 0.4$) was designed through compositional tuning. The added comparison shows that the superstructure reflection relevant to the photomagnetic response evolves from a relatively sharp feature in $La_{2/3}TiO_3$ ($x = 0$) to a broadened and diffuse feature near the alterlattice composition $La_{0.4}Ca_{0.4}TiO_{3-\delta}$. The apparent ordering correlation length decreases from 41.5 nm to 3.4 nm, indicating the formation of abundant local structural domains that may favor photomagnetic coupling. We also compared the photomagnetic responses across different compositions. The negative photomagnetic response is weak in $La_{2/3}TiO_3$, becomes stronger after Ca introduction, reaches its maximum near $La_{0.4}Ca_{0.4}TiO_{3-\delta}$ (the added Fig. S4 and the revised manuscript, lines 146–151), and then weakens as the composition moves away from this range. These results establish a more direct correlation between the distinctive A-site-vacancy-ordered domains in the alterlattice and the enhanced photomagnetic response, moving beyond the single-composition analysis in the original manuscript.

(ii) We also revised the mechanistic language (the revised manuscript, lines 175–178). We state that compositionally tuned short-range structural order provides an experimentally accessible route to enhance the low-temperature photomagnetic response in this A-site-deficient titanate system.

(iii) We removed the speculative statement on potential applications in spin-based quantum information technologies. We also removed the original Figure S31 and deleted the application schematic. The revised text now uses more restrained wording (the revised manuscript, lines 28–29, 84–86, 478–480, 485–486), emphasizing the alterlattice as a platform for studying structure-mediated photomagnetic coupling and as a structural tuning route for low-temperature light-controllable magnetic oxides.

These revisions directly address the reviewer's concerns about the manuscript logic, the structure–magnetism connection, the wording of "design strategy," and the overstated application discussion.

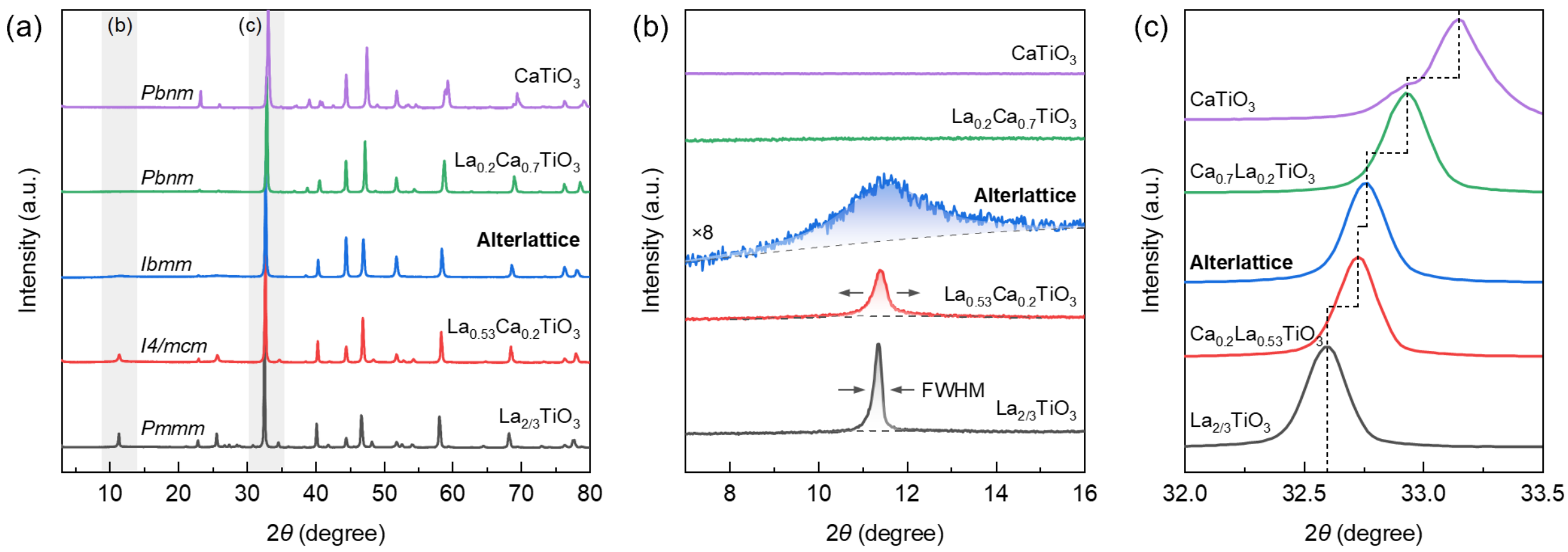

Figure R1. Structural evolution towards the alterlattice (x = 0.4) in the $La_{2(1-x)/3}Ca_xTiO_3$ series. (a) Full-range XRD patterns (2θ = 3–80°) reveal the emergence of the alterlattice upon Ca incorporation into the parent phase, driven by A-site chemical disorder and crystallographic phase competition. **(b)** Low-angle XRD patterns (2θ = 7–16°), featuring Scherrer analysis of the superstructure reflection near $2\theta \approx 12°$ to determine the full width at half maximum. **(c)** High-resolution XRD patterns (2θ = 32–33.5°), tracking the progressive shift of the strongest main-lattice reflection to higher angles with increasing Ca content.

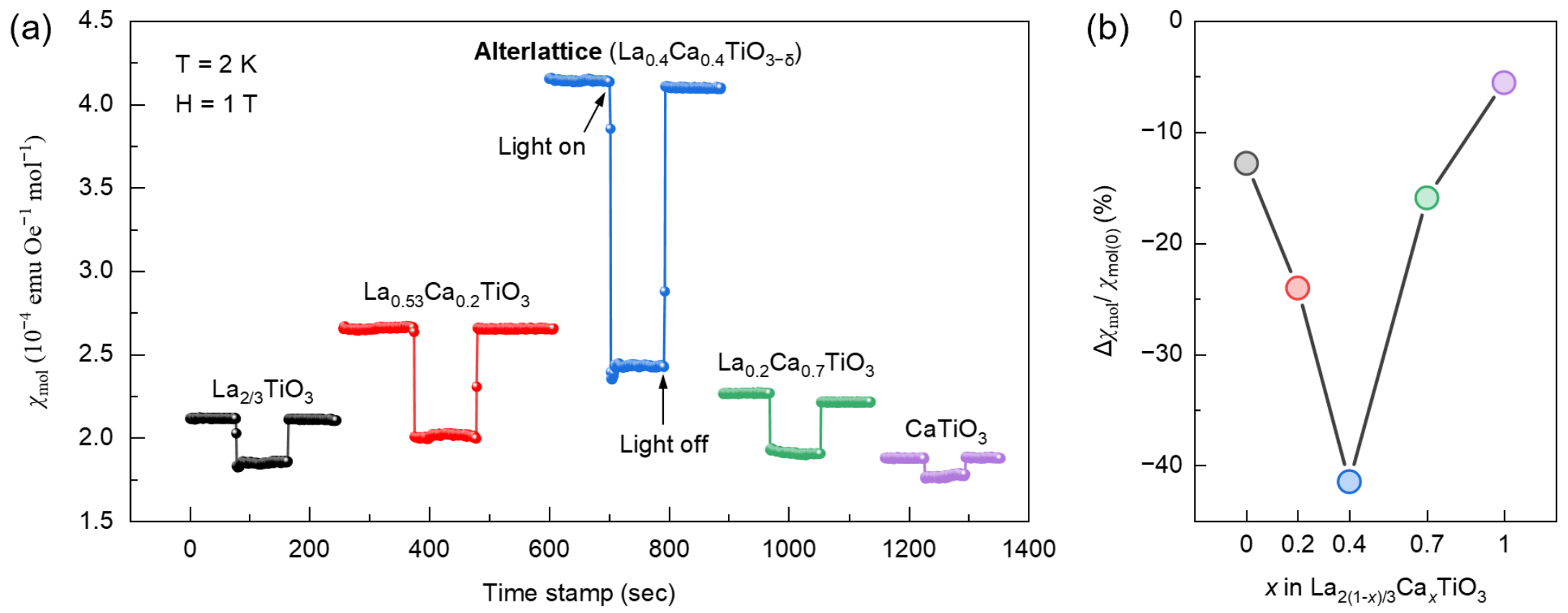

Figure S4 | Light on/off photomagnetic measurements of the $La_{2(1-x)/3}Ca_xTiO_3$ series. Time evolution of the molar magnetic susceptibility (χ_{mol}) at 2 K under 1 T using 473 nm illumination with a light intensity of 10 mW mm^{-2}. The measurements show a negative photomagnetic response, characterized by a rapid decrease in χ_{mol} under illumination and recovery after the light is turned off. **(a)** Negative photomagnetic responses of $La_{2(1-x)/3}Ca_xTiO_3$ with x = 0, 0.2, 0.4, 0.7, and 1.0. **(b)** Comparison of $\Delta\chi_{mol}/\chi_{mol(0)}$ for different compositions under identical conditions, where $\Delta\chi_{mol}$ is the difference between χ_{mol} under illumination and χ_{mol} in the dark state, and $\chi_{mol(0)}$ is the dark-state value before illumination.

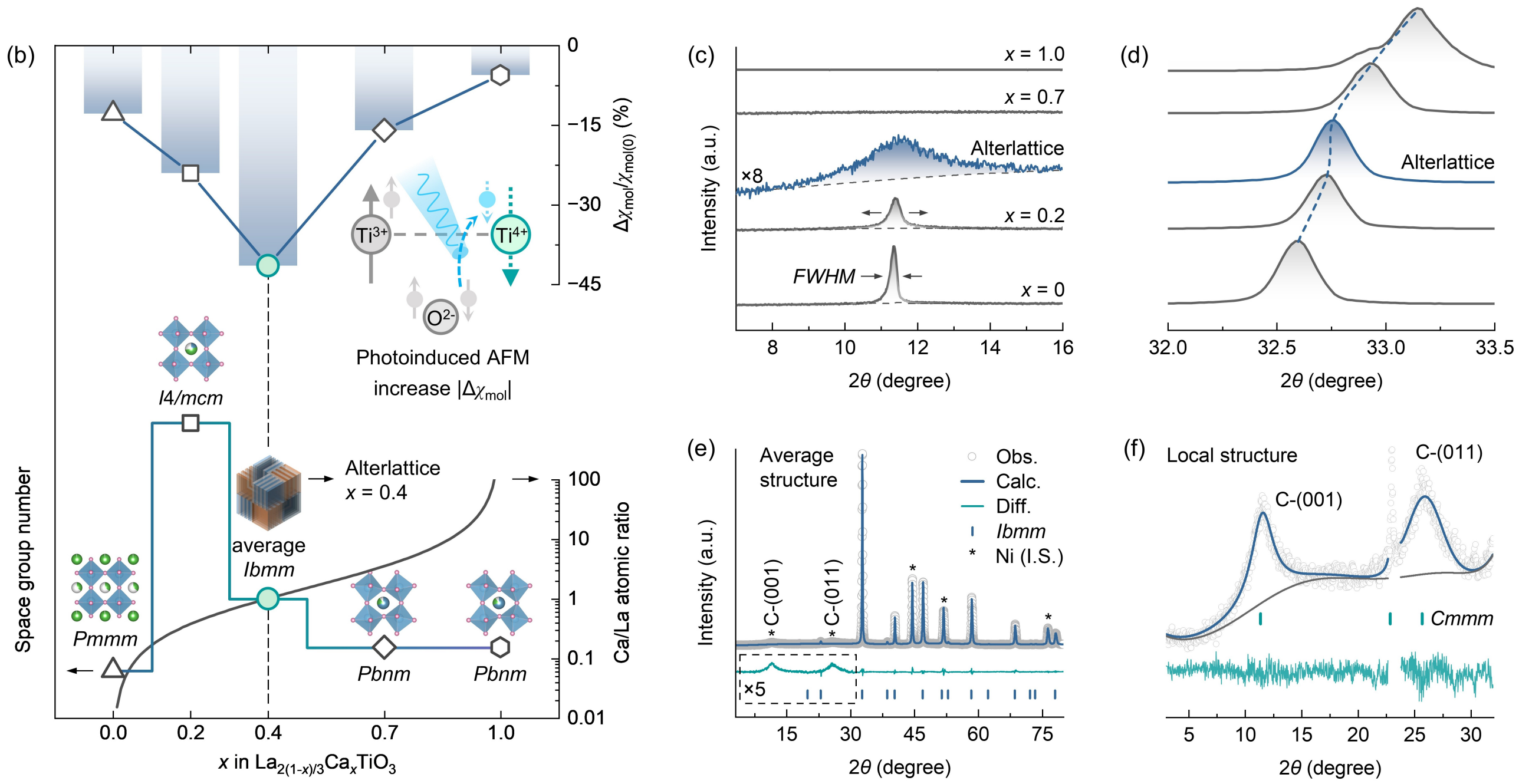

Fig. 1b-f | Structural paradigm and design strategy of the alterlattice driven by competitive coupling. (b) Compositional window and negative photomagnetic response $\Delta\chi_{mol}/\chi_{mol(0)}$ of $La_{2(1-x)/3}Ca_xTiO_3$. The alterlattice appears near the orthorhombic-tetragonal phase boundary and is most pronounced near equimolar La and Ca (x = 0.4), where it exhibits the largest negative $\Delta\chi_{mol}/\chi_{mol(0)}$ at 2 K and 1 T under 473 nm illumination at 10 mW mm^{-2}. The upper-right inset illustrates the proposed mechanism for photo-induced AFM correlations. Photoexcitation transfers an electron from O 2*p* states into Ti 3*d* states. The resulting Ti^{3+}-like state enhances local spin compensation with nearby Ti^{3+}, leading to a decrease $\Delta\chi_{mol}$ in $\chi_{mol(0)}$ relative to the dark state. **(c)** XRD patterns of $La_{2(1-x)/3}Ca_xTiO_3$ in the 2θ range of 3–16°, showing broadening of the superstructure reflection near 11.5°, indicating a progressive decrease in the correlation length of V_A ordering with increasing Ca substitution. **(d)** High-resolution XRD patterns in the 2θ range of 32–33.5°, showing the shift of the main reflection in $La_{2(1-x)/3}Ca_xTiO_3$ toward higher angles with increasing Ca substitution. **(e)** Rietveld refinement of $La_{0.4}Ca_{0.4}TiO_{3-\delta}$ based on the average *Ibmm* structure (ICSD-245543). Discrepancies between the observed and calculated diffraction patterns near 2θ = 11.5° and 25.6° arise from microstructural features consistent with *Cmmm* symmetry that coexist with *Ibmm* phase. Obs. in the figure denotes the observed pattern, Calc. the calculated pattern, Diff. the difference pattern, and Ni (I.S.) the reflection positions of the Ni internal standard. **(f)** Rietveld refinement of $La_{0.4}Ca_{0.4}TiO_{3-\delta}$ based on the *Cmmm* structure (ICSD-245545). Broadened superstructure reflections assigned to C-(001) and C-(011) evidence short-range order of the alterlattice.

Comments 1.3: "The structure of the manuscript need to be improved. After the introduction part, the manuscript keeps on describing experimental results with very little analysis. And then in the discussion part, the manuscript starts to recap the previous descriptions before in-depth discussions, which is not efficient at all. The authors should consider to reorganize the whole manuscript and try to merge more discussion into the experimental part, and describe more about the motivation to do this or that measurement."

Response 1.3: We thank the reviewer for the constructive comments on the organization and presentation of the manuscript. In the original version, the structural characterization and the discussion of the physical properties could be streamlined further to improve readability and logical flow. The motivation for key measurements is now stated more explicitly, and the Discussion has been reorganized to avoid redundancy and to strengthen the link

between experimental results and mechanistic interpretation. We have reorganized the manuscript accordingly. The related text has been updated in the revised manuscript.

(i) We moved part of the discussion closely related to the experimental results into the corresponding Results sections (the revised manuscript, lines 163–175, 291–296, 331–334…). The structural, magnetic, and photoresponse results are now followed more directly by their physical interpretation, rather than being summarized again only in the Discussion.

(ii) We clarified the motivation for the key measurements. STEM and SAED are now presented as complementary probes of the average structure, short-range ordering, and superstructure modulation (the revised manuscript, lines 193–194, 252–254). Light on/off magnetic measurements, temperature- and field-dependent magnetization, and wavelength- and thickness-dependent measurements are now described in relation to the photoinduced magnetic response and its possible origin (the revised manuscript, lines 319, 340–341, 356–357, 378–379, 402–403).

(iii) We removed repetitive recapitulation from the Discussion. The revised Discussion now focuses on connecting the main observations, including the alterlattice structure, Ti^{3+}-related local moments, photogenerated carriers, and possible short-range antiferromagnetic correlations (the revised manuscript, lines 417–483).

These revisions improve the logical flow of the manuscript and make the presentation more question-driven, rather than a simple sequence of experimental results.

Comments 1.4: "In the part discussing about the photo-induced magnetic properties, I feel it difficult to follow since comparison between ZFC/FC and light-on/off are not described with enough order. χ_m, moment and $1/\chi_{mol}$, etc. are plotted without explanation of why to plot like that. Why not using $1/\chi_m$ instead of $1/\chi_{mol}$? And in Fig.S19 a similar figure is shown, but with a log scale, which is not consistent with Fig.4."

Response 1.4: We thank the reviewer for this important comment. In the original manuscript, the discussion of the photoinduced magnetic properties did not distinguish clearly enough among the ZFC/FC results, the light on/off measurements, and the dark-versus-illuminated comparisons. The definitions and usage of moment, mass susceptibility, molar susceptibility, and inverse susceptibility also required a more consistent presentation. We have therefore reorganized this section and unified the notation throughout the text and figures.

(i) We reorganized the "Giant negative photomagnetism in the alterlattice" section (the revised manuscript, lines 319–415). The revised manuscript now first presents the dark-state ZFC/FC curves to establish the baseline magnetic behavior, then shows the light on/off measurements to demonstrate the reversibility and time response, and finally compares the dark and illuminated states under the same measurement conditions.

(ii) We replaced $1/\chi_m$ with $1/\chi_{mol}$ in the revised figure and manuscript (such as the revised Fig. 4b, revised Fig. 4c, and the corresponding text in the revised manuscript). Because the composition is fixed, χ_m and χ_{mol} differ only by a constant molar-mass factor, so this change does not alter the physical trend. It improves consistency with the rest of the photomagnetic analysis.

(iii) We corrected the inconsistency between Figure 4e and the previous Fig. S19. The corresponding supplementary figure has been replotted with the same axis format (the revised Fig. S29).

These revisions make the relationships among ZFC/FC, dark/light comparison, and light on/off measurements clearer, and unify the notation, units, and graphical presentation across the main text and Supporting Information.

Comments 1.5: "The mechanism of photo-induced AFM seems to be a hypothesis, but not a solid confirmation. The authors showed deviation from Curie-Weiss scaling in Fig.4e, but the high temperature part of the 1/χ-H curve of an AFM material should also follow Curie-Weiss scaling but with a negative intersection with the 1/χ axis, which seems not the case in Fig.4e."

Response 1.5: We thank the reviewer for this careful comment. In the original manuscript, the term "photo-induced AFM" could have been interpreted as implying long-range antiferromagnetic order, and we have therefore revised the wording throughout the manuscript to "photo-induced AFM correlations" (such as the revised manuscript, lines 86, 112, 354…).

(i) We revised the interpretation of the low-temperature deviation from Curie–Weiss behavior (the revised Fig. 4e, added Fig. S26, added Table S5 and the revised manuscript, lines 365–372). The low-temperature deviation from Curie–Weiss behavior under illumination suggests the possible emergence of AFM correlations. In the revised manuscript, the illuminated χ_{mol} versus 1/T data are well fitted by a low-temperature AFM model with an excitation gap, suggesting that the photoinduced magnetic response is more likely associated with short-range, one-dimensional local AFM correlations.

(ii) We added high-temperature Curie–Weiss analysis under illumination (the added Fig. S27 and the revised manuscript, lines 371–372). The illuminated χ_{mol} versus 1/T data follow Curie–Weiss behavior at sufficiently high temperature, with a weakly negative θ. This result indicates that illumination does not induce an AFM state in the 50–100 K paramagnetic regime.

(iii) We added wavelength-dependent measurements (the added Fig. S23 and the revised manuscript, lines 350–354), together with the thickness- and field-dependent results, to further constrain the mechanism. These results support a carrier-mediated process involving defect-related electronic states and Ti^{3+}/Ti^{4+} local environments.

These revisions address the reviewer's concern by separating the experimental observation from the mechanistic interpretation. The revised manuscript describes a photo-induced local AFM correlation that is consistent with the susceptibility analysis and photo-response measurements.

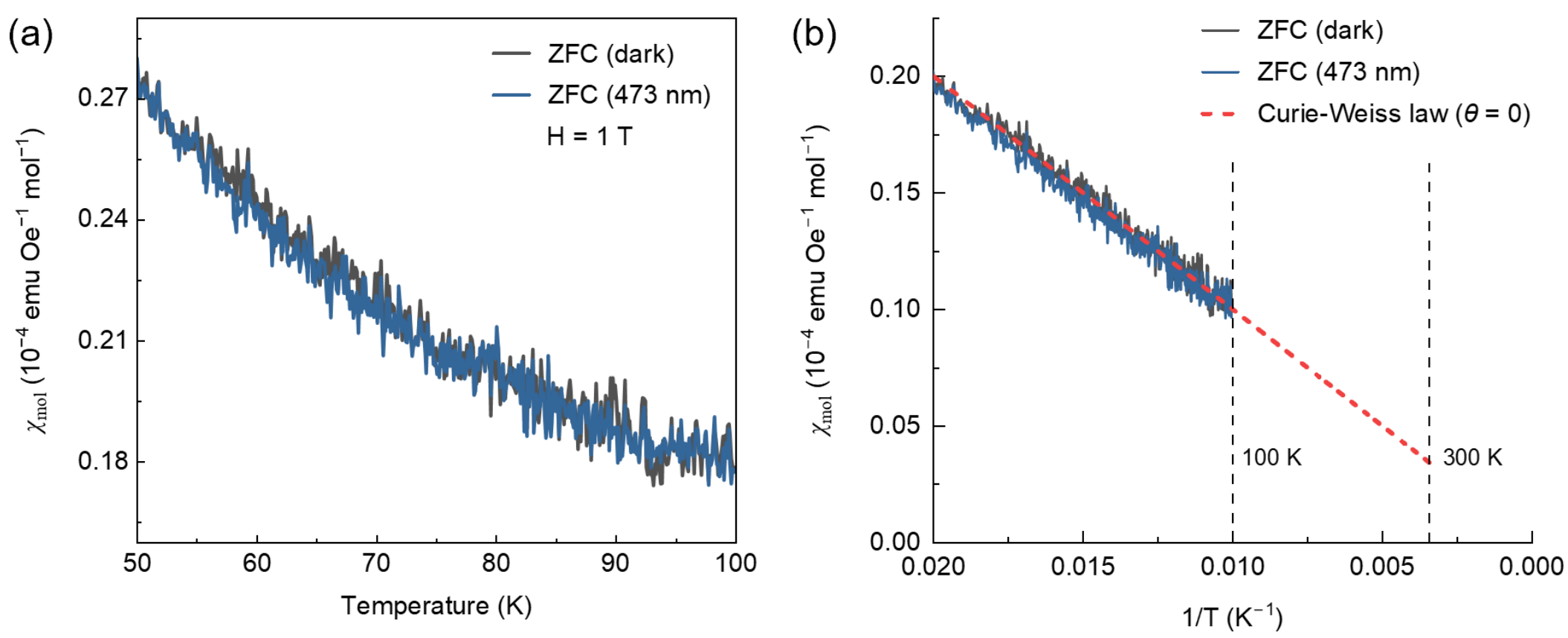

Figure S27 | Paramagnetic behavior under illumination in the 50–100 K range. (a) Temperature dependence of χ_{mol} for the 0.48 mm thick sample from 50 to 100 K, measured at H = 1 T under dark conditions and 473 nm illumination with a light intensity of 14.2 mW mm^{-2}. The ZFC curves under dark and illuminated conditions nearly overlap, indicating that illumination does not induce obvious magnetic ordering at higher temperatures. **(b)** χ_{mol}–1/T analysis showing that both dark and illuminated curves are close to ideal Curie–Weiss behavior with very small Weiss temperatures. This indicates that no pronounced magnetic exchange interaction is present from 50 to 100 K, and that both curves are mainly governed by paramagnetic behavior from dilute magnetic centers.

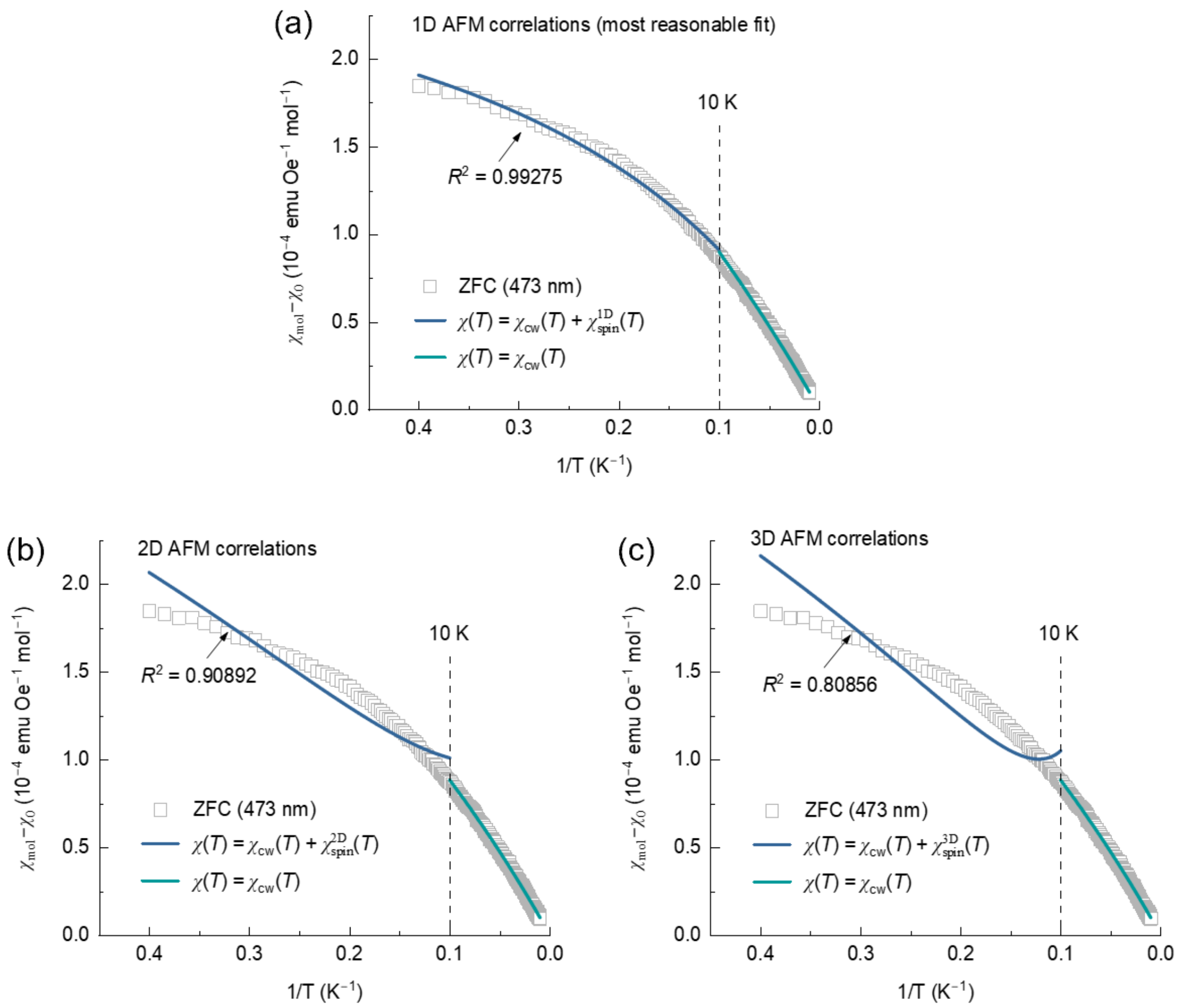

Figure S26 | Fitting and analysis of the 473 nm light-induced magnetic response from 2.5 to 10 K using a combined paramagnetic (PM) and antiferromagnetic (AFM) correlation model $\chi(T) = \chi_{cw}(T) + \chi_{spin}(T)$. The PM contribution ($\chi_{cw}(T)$) was compared using a Curie–Weiss model described by $\chi_{mol}(x) - \chi_0 = Cx/(1-\theta x)$, where x = 1/T, χ_0 is the temperature-independent background susceptibility, C is the Curie constant, θ is the Weiss temperature. The AFM contribution ($\chi_{spin}(T)$) was compared using 1D, 2D, and 3D models described by $\chi_{mol}(x) - \chi_0 = ax\^{}(1-d/2)e\^{}(-\Delta x)$, where x = 1/T, χ_0 is the temperature-independent background susceptibility, a is the amplitude coefficient of the AFM term, d is the effective excitation dimensionality, and Δ is the characteristic AFM energy gap. Panels **(a)**, **(b)**, and **(c)** correspond to 1D-, 2D-, and 3D-like thermally activated AFM excitations, respectively. The illuminated χ versus 1/T curve was fitted with the model containing the AFM term from 2.5 to 10 K, while the 10–100 K range was fitted using a single Curie–Weiss model. The physically reasonable fitting parameters indicate that the light-induced magnetic response below 10 K is more consistent with a 1D AFM-like correlation. The small characteristic energy gap suggests a short-range ordered response, likely related to structural confinement from the short-range ordered superstructure domains in the alterlattice.

Table S5 | Fitting parameters obtained from Curie–Weiss and *d*-dimensional ($d = 1, 2, 3$) AFM spin-excitation models in Figure S26. In the Curie–Weiss term, χ_0 (emu Oe^{-1} mol^{-1}) is the temperature-independent background susceptibility, C (emu K Oe^{-1} mol^{-1}) is the Curie constant, θ (K) is the Weiss temperature. In the gapped low-dimensional AFM excitation term, a (emu Oe^{-1} mol^{-1} $K^{1-d/2}$) is the amplitude coefficient of the AFM term, d is the effective excitation dimensionality, and Δ (K) is the characteristic AFM energy gap.

Model	Fitting range	Fitting parameters			
		χ_0	C	θ	R^2
PM	10–100 K	8×10^{-6}	0.001013	−1.187151	0.998583
		a		Δ	R^2
1D AFM	2.5–10 K	0.000123		1.335813	0.992759
2D AFM	2.5–10 K	0.000123		12.063121	0.908927
3D AFM	2.5–10 K	0.000123		22.806433	0.808562

χ_0 represents a temperature-independent contribution, which may be associated with defect states related to oxygen vacancies or the Pauli paramagnetism of partially delocalized Ti 3*d* electrons.

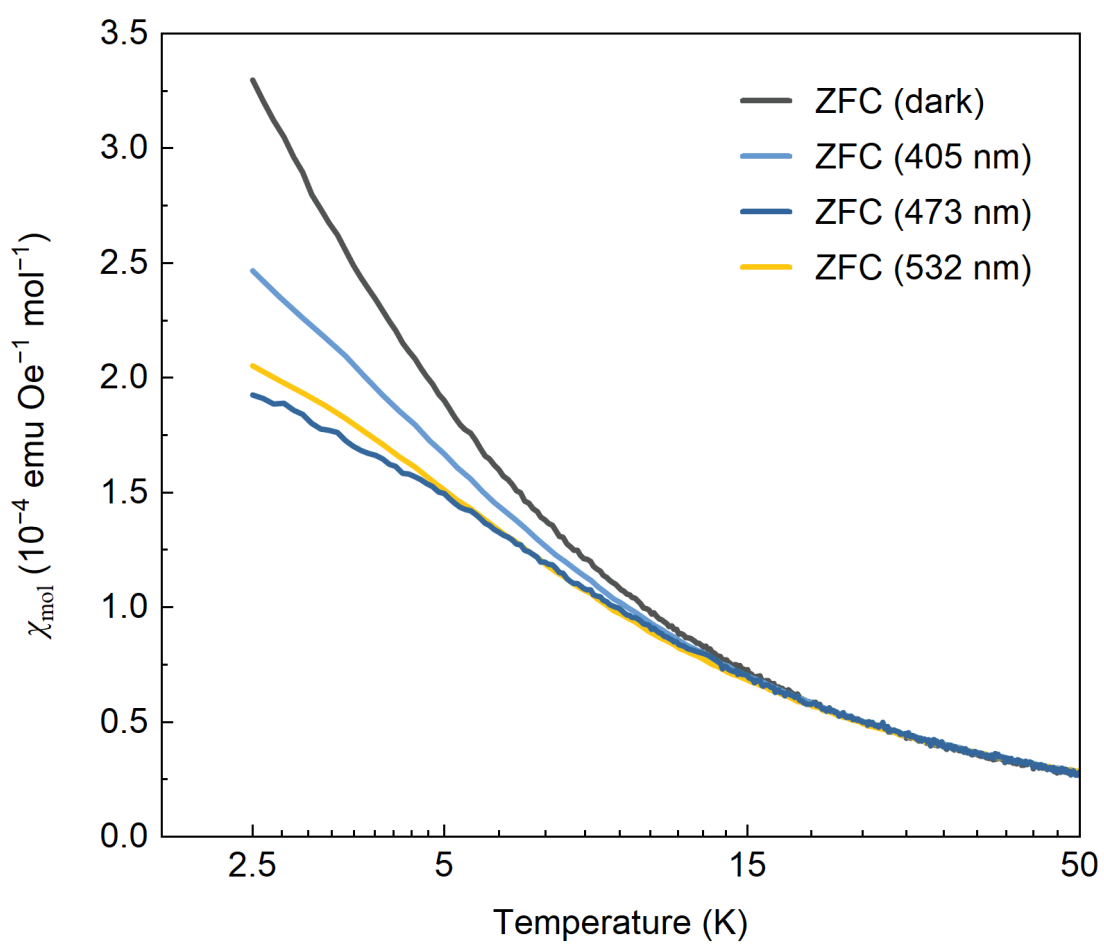

Figure S23 | Wavelength-dependent ZFC curves of the alterlattice. Comparison of the ZFC curves of the 0.48 mm-thick sample measured in dark conditions and under 405, 473, and 532 nm illumination. The ZFC curve measured under 473 nm illumination shows the most pronounced decrease compared with the dark-state curve, indicating that 473 nm light induces the strongest antiferromagnetic-like exchange interaction in the alterlattice. This wavelength selectivity supports a photocarrier-mediated mechanism for the formation of the light-induced magnetic response.

Smaller advices:

Comments 1.6: "The authors mainly discussed about symmetries of Ibmm, Cmmm, or Pmmm, which all belong to orthorhombic crystal system. However, at some place tetragonal phase is also mentioned. It should be explained that why tetragonal phase could not be observed at an orthorhombic-tetragonal phase boundary."

Response 1.6: We thank the reviewer for this important comment. In the original manuscript, the discussion of the tetragonal phase was not sufficiently explicit. We clarified that the tetragonal phase mentioned in the manuscript refers to a neighboring competing phase in the reported composition–temperature phase diagram of $Ca_{1-x}La_{2x/3}TiO_3$, not to a long-range tetragonal phase observed in the present $La_{0.4}Ca_{0.4}TiO_{3-\delta}$ sample.

(i) We added Rietveld refinement results for comparison samples in the $La_{2(1-x)/3}Ca_xTiO_3$ series to show the composition-dependent structural evolution (the added Fig. S3, added Table S2 and the revised manuscript, lines 130–133). These results are consistent with the reported phase sequence.

(ii) For the present $La_{0.4}Ca_{0.4}TiO_{3-\delta}$ sample, no independent XRD, SAED, or STEM evidence supports a long-range tetragonal *I*4/*mcm* phase. The average structure is therefore assigned to orthorhombic *Ibmm*. We further clarified that the very similar a and b lattice parameters indicate a pseudo-tetragonal metric feature near the orthorhombic–tetragonal boundary, rather than true tetragonal symmetry. Local tetragonal-like distortions or competing octahedral-tilting tendencies may exist, but they remain short-ranged and are averaged into the orthorhombic structure in diffraction.

These revisions distinguish the experimentally observed orthorhombic average structure from the neighboring tetragonal phase in the phase diagram and explain why no resolvable long-range tetragonal phase is observed in the present sample.

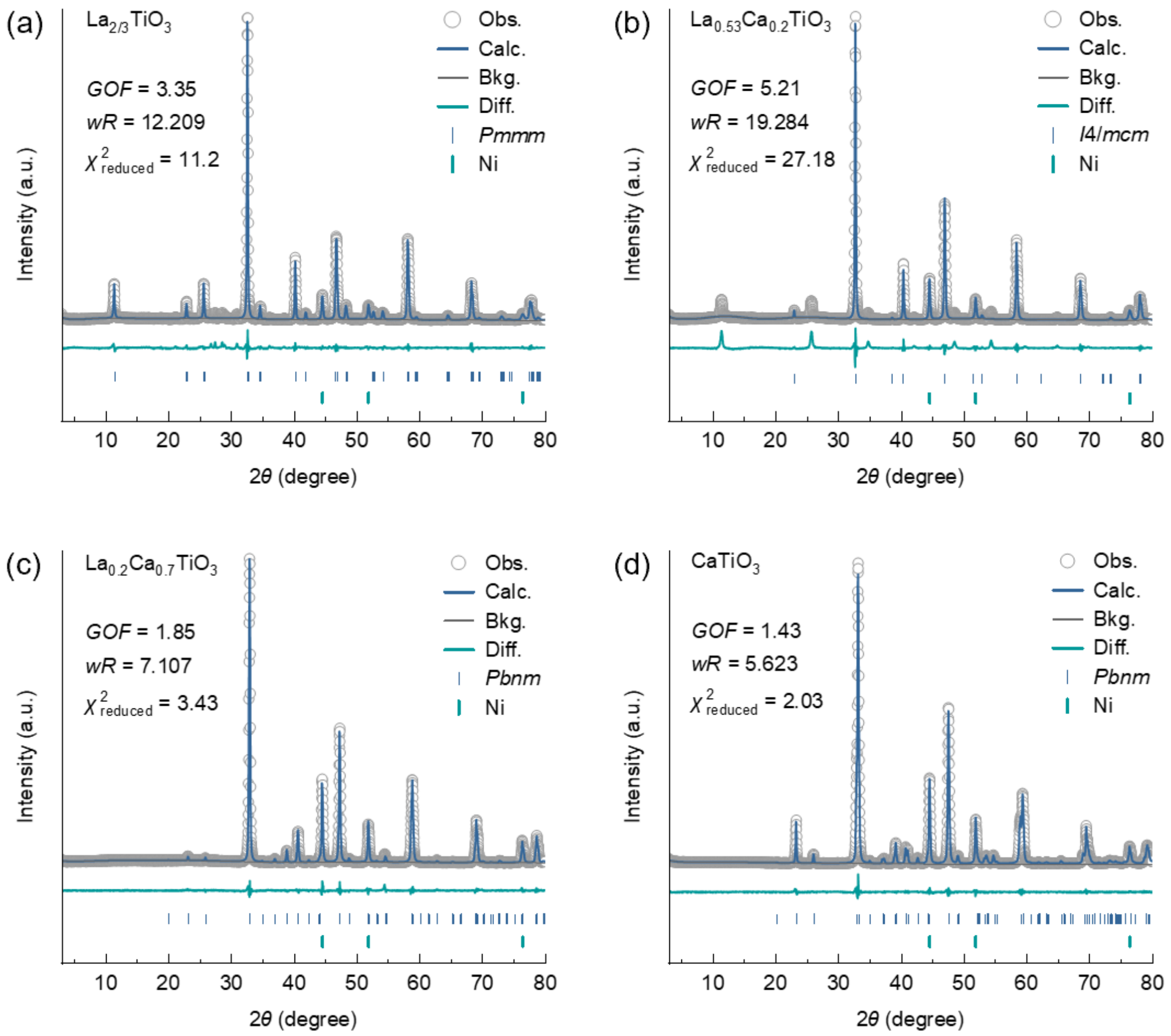

Figure S3 | Rietveld refinement profiles for the $La_{2(1-x)/3}Ca_xTiO_3$ series (x = 0, 0.2, 0.7, and 1.0). (a) $x = 0$ ($La_{2/3}TiO_3$) with *Pmmm* symmetry (ICSD-77671). **(b)** x= 0.2 ($La_{0.53}Ca_{0.2}TiO_3$) with *I*4/*mcm* symmetry (ICSD-245544). **(c)** x = 0.7 ($La_{0.2}Ca_{0.7}TiO_3$) with *Pbnm* symmetry (ICSD-245542). **(d)** x = 1.0 ($CaTiO_3$) with *Pbnm* symmetry (ICSD-150420). Reliability factors (*GOF*, *wR*, reduced χ^2) are given in the inset to each panel. **Note**: Reduced χ^2 represents the variance-weighted residual normalized by the number of degrees of freedom and is used to evaluate the statistical quality of the refinement. *wR* is the weighted residual factor, which describes the weighted difference between the observed and calculated diffraction profiles. *GOF* denotes the goodness of fit and provides an overall measure of the agreement between the experimental and calculated patterns.

Comments 1.7: "In line 119, "multi-q superstructure" is mentioned, with reference of XRD refinement results. I can only see that the refinement results are good, but it is not clear at all what is this superstructure, for example what are the "multi" q values."

Response 1.7: We thank the reviewer for pointing this out. We have clarified the meaning of the term "double-**q** superstructure" in the revised manuscript and revised Supporting Information.

We added an explicit definition of the A-site-vacancy related superstructure modulation (the added Fig. S1 and the revised manuscript, lines 122–124). In A-site-deficient perovskites, partial ordering of A-site cations and vacancies introduces additional periodicity beyond the parent lattice, giving rise to half-integer superstructure reflections. The modulation vector **q** describes both the displacement of the superstructure reflections from the parent reciprocal vector **G** ($\mathbf{Q} = \mathbf{G} + \mathbf{q}$) and the ordering direction of the vacancy-related superstructure. In the *Ibmm* reciprocal-lattice setting, the two modulation vectors are $\mathbf{q}_1 = ½[110]$ and $\mathbf{q}_2 = ½[1\bar{1}0]$. These two mutually orthogonal vectors account for the half-integer superstructure reflections observed in the diffraction data. Fig. S1 illustrates the real-space modulation, reciprocal-space indexing, and the relationship between the main reflection **G** and the modulation vectors $\mathbf{q}_1$ and $\mathbf{q}_2$.

These revisions distinguish the *Ibmm* average structure from the short-range double-**q** superstructure and clarify the meaning, values, and experimental origin of the two modulation vectors.

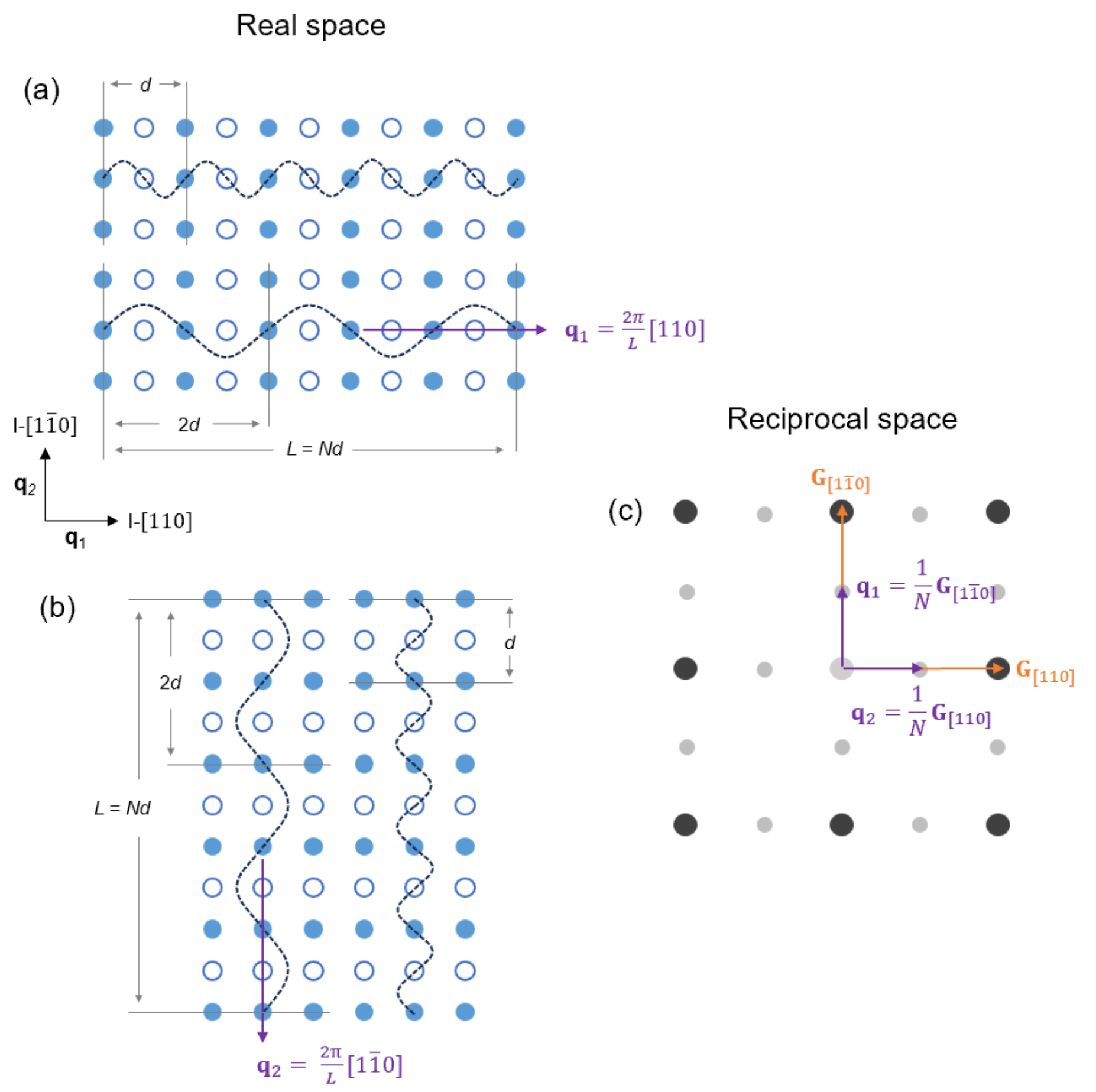

Figure S1 | Real-space and reciprocal-space schematics of the double-q superstructure modulation vectors in the alterlattice. (a,b) Local superstructure modulations along [110] and [1$\bar{1}$0] orthogonal directions. In real space, A-site cation/vacancy-related ordering and local lattice distortions introduce an additional twofold periodic modulation on the perovskite lattice. The modulation periods extend along [110] and [1$\bar{1}$0]. The characteristic length is $L = Nd$, where d is the parent-lattice spacing and N is the period multiplication factor. Here, $N = 2$. The

corresponding modulation vectors are $\mathbf{q}_1 = \mathbf{G}_{[110]}/N$ and $\mathbf{q}_2 = \mathbf{G}_{[1\bar{1}0]}/N$, where $\mathbf{G}_{[110]}$ and $\mathbf{G}_{[1\text{-}10]}$ are the parent-lattice reciprocal vectors along the corresponding directions. Equivalently, $|\mathbf{q}| = 2\pi/L$. Thus, q describes the additional periodicity of the superstructure relative to the parent lattice. The filled and open blue circles indicate periodic variations in local structural environment or site occupancy. The dashed lines indicate modulation waves caused by local structural distortions. **(c)** Reciprocal-space schematic corresponding to the real-space modulation. The black spots represent the fundamental reflections $\mathbf{G}_{[110]}$ and $\mathbf{G}_{[1\bar{1}0]}$. The purple arrows represent $\mathbf{q}_1$ and $\mathbf{q}_2$ generated by the superstructure. For a commensurate superstructure, $\mathbf{q} = \mathbf{G}/N$. Therefore, the superstructure reflections appear at half-integer positions between main reflections, with displacement directions consistent with **q**. The two orthogonal modulation vectors indicate that this is not a simple single-**q** modulation, but a double-**q** superstructure with a two-dimensionally coupled modulation network. The double-**q** modulation is a key reciprocal-space signature of the local ordered state in the alterlattice.

Comments 1.8: "In Fig. S2b, I cannot recommend such plot with atom dots on the TEM images. The TEM image seems just a square lattice and there is no sign of alternating two-atom and one-atom arrays, as shown by the red dots. Such figure is misleading and does not provide any useful information, since you can also overlap a simple square lattice or some other similar lattice to the TEM image."

Response 1.8: We thank the reviewer for the careful evaluation of this figure. The corresponding STEM figures have been revised accordingly (the revised Figs. S9b and S13b), retaining only the cation-lattice framework directly supported by the experimentally visible contrast and periodicity.

Comments 1.9: "Local fluctuations in La and O concentrations is mentioned, but in Fig.S3 it seems to me not so obvious. One can never observe identical atom intensity in TEM images and there are always some intensity fluctuations due to statistics or sample preparation, etc."

Response 1.9: We thank the reviewer for raising this important issue. In the original manuscript, the local La and O variations were described mainly from EELS contrast maps, whereas such contrast can also be affected by counting statistics and noise. We have therefore revised the wording and added quantitative analysis to avoid overinterpretation.

We added atomic-site-resolved La intensity statistics for the analyzed region (the revised Fig. S7, and the revised manuscript, lines 197–198). The revised Fig. S7 now includes the La-column intensity distribution, the mean value, the standard-deviation range, and the columns showing relatively low La intensity. The La-signal variation is now discussed as evidence for nonuniform A-site occupancy and local chemical inhomogeneity associated with A-site vacancy distribution, supported by quantitative intensity statistics and repeated line-profile analysis. The O-related signal is now described more cautiously. We no longer assign it as a definite O concentration fluctuation. Instead, it is treated as a possible weak variation in the O-related EELS signal and used only as auxiliary evidence for changes in the local chemical environment.

These revisions distinguish statistically supported La-signal variations from the weaker O-related contrast variation and reduce the possibility of interpreting random image-intensity fluctuations as definite chemical fluctuations.

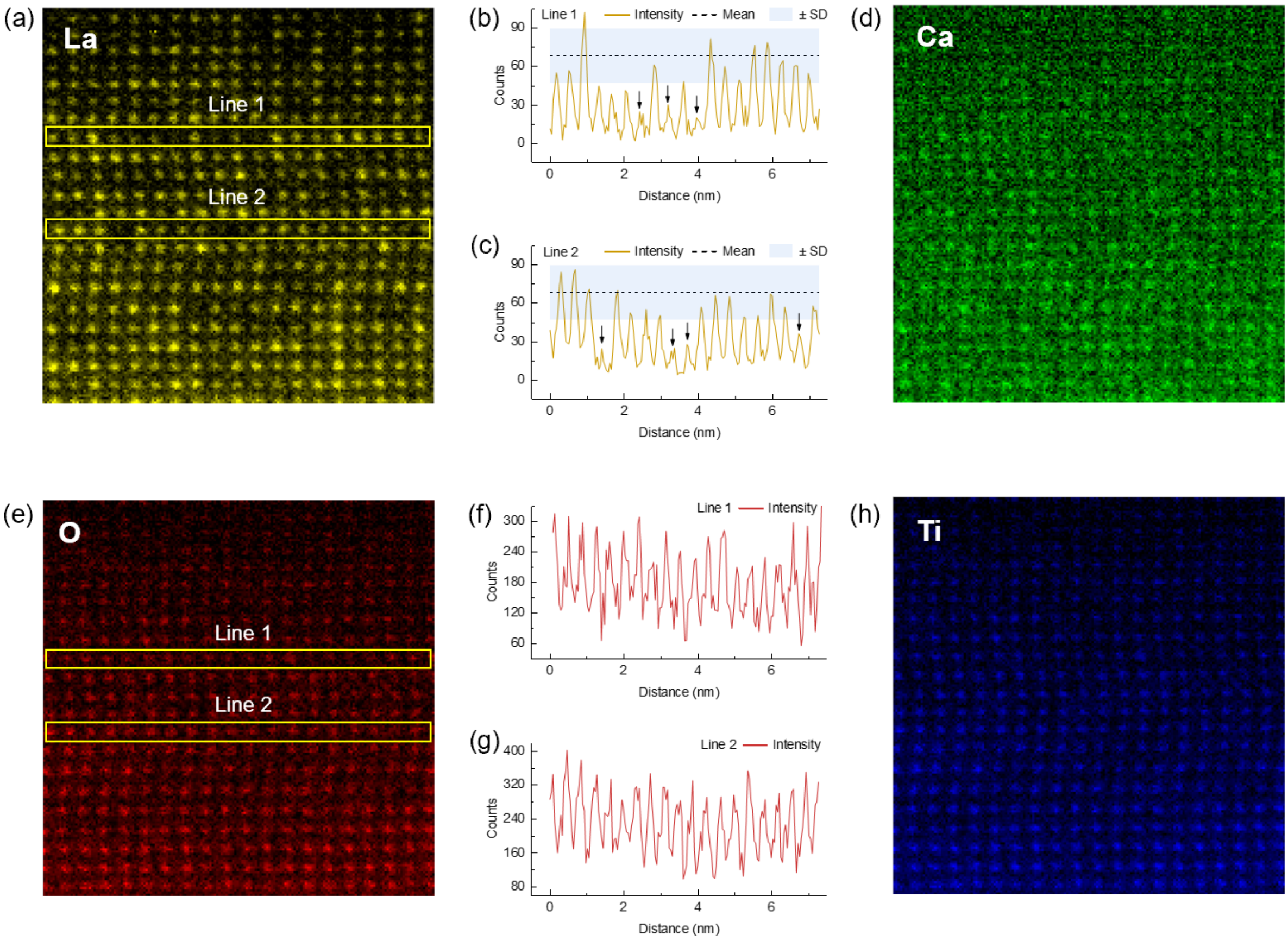

Figure S7 | Atomic-scale EELS analysis of local chemical inhomogeneity. (a) EELS map of La (La $M_{4,5}$ edge) along the I-[001] zone axis. **(b)** Line-intensity profile from boxed region 1 in (a), showing La-deficient or La-vacancy-rich regions. Compared with the statistical intensity distribution, the arrow-marked sites show intensities below the mean value, indicating positions where La deficiency is more pronounced. **(c)** Line-intensity profile from region 2 in (a), further confirming La-deficient sites. The yellow curve is the measured intensity profile, the black dashed line is the average intensity from 80 atomic columns, and the blue region represents the mean ± one standard deviation. **(d)** EELS map of Ca (Ca $L_{2,3}$ edge) along the I-[001] zone axis. **(e)** EELS map of O (O K edge) along the I-[001] zone axis. **(f)** Line-intensity profile from boxed region 1 in (e), showing slight variation in the O-related signal. **(g)** Line-intensity profile from boxed region 2 in (e), showing a similar trend. (h) EELS map of Ti (Ti $L_{2,3}$ edge) along I-[001].

Comments 1.10: "I suggest Fig.4h be replaced by some fluence dependence of the demagnetization effect, such as Fig. S23. The present figure shows similar information as Fig.4b and can be put into the supplementary material."

Response 1.10: We thank the reviewer for this constructive suggestion. The original Fig. 4h has been moved to the revised Supporting Information (the added Fig. S31a), and replaced by a laser power-density- and sample-thickness-dependent analysis of the negative photoinduced magnetic response based on the original Fig. S23 (the revised Fig. 4h and the revised manuscript, lines 406–412).

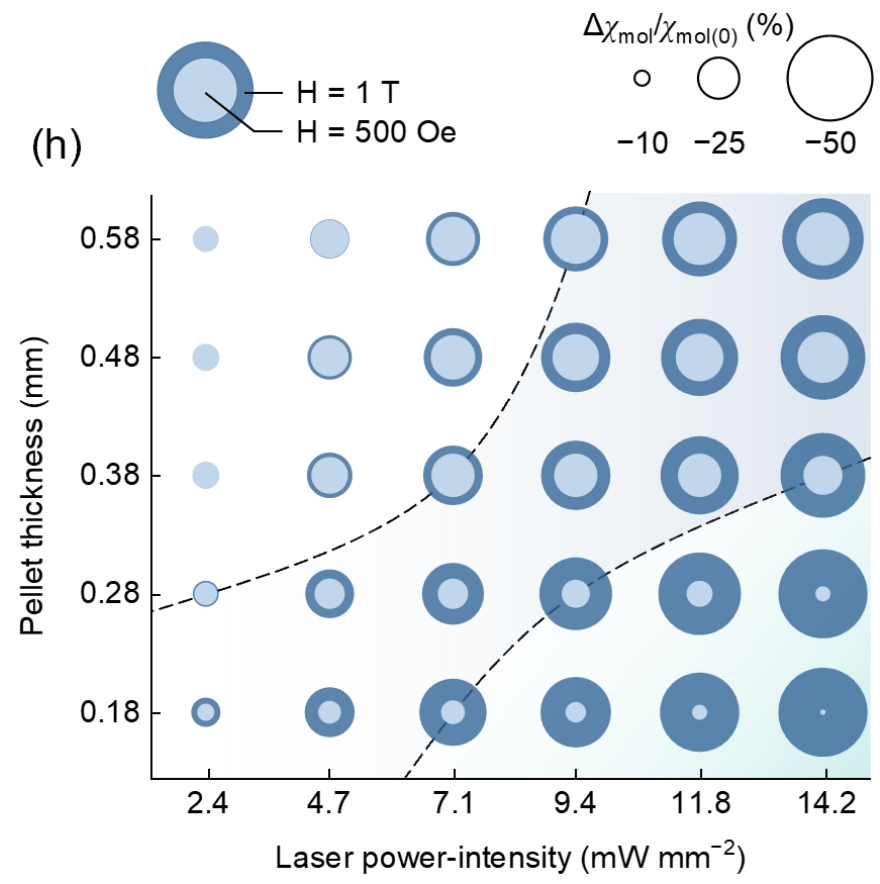

Fig. 4h. Size-coded scatter plot of the laser power-density (I) and thickness (d) dependence of $\Delta\chi_{mol}/\chi_{mol(0)}$ for alterlattice pellets measured at 1 T and 500 Oe. Outer and inner circles denote measurements at 1 T and 500 Oe, respectively, while circle size scales with $\Delta\chi_{mol}/\chi_{mol(0)}$. The difference in $\Delta\chi_{mol}/\chi_{mol(0)}$ between 500 Oe and 1 T is small at $d \geq 0.28$ mm and $I \leq 2.4$ mW mm^{-2}, but becomes pronounced at $d \leq 0.28$ mm and $I \geq 7.1$ mW mm^{-2}, reflecting field-modulated photocarrier dynamics that alter the population of photo-induced Ti^{3+}-like species.

Comments 1.11: "In Fig.S15a, why a part of the blue curve is missing?"

Response 1.11: We thank the reviewer for noting this detail. The missing segment in the original blue curve was caused by incomplete data acquisition during the PPMS-VSM temperature sweep. The corresponding measurement has been repeated, and the original Fig.S15a (the revised Fig. S25a) now shows the complete dataset without the missing temperature interval.

Comments 1.12: "It is shown in Fig. S16 "ZFC/FC (dark)" magnetization curve, but the measurement results of ZFC and FC could not be exactly identical. Please show both curves. And it is mentioned that "these $\Delta\chi_{mol}/\chi_{mol(0)}$ were obtained by nonlinear fitting to reduce noise"(line 256). I do not understand how to reduce noise with a mathematic fitting and see no physical meaning of the fitting parameters in Fig. S16."

Response 1.12: We thank the reviewer for pointing out this issue. In the original manuscript, the label "ZFC/FC (dark)" was not sufficiently clear, and the statement that $\Delta\chi_{mol}/\chi_{mol(0)}$ was obtained by "nonlinear fitting to reduce noise" could lead to misunderstanding.

(i) The independently measured ZFC and FC curves under dark conditions are now shown (the added Fig. S18 and the revised manuscript, lines 329–330). The two curves show the same overall paramagnetic trend but retain small differences.

(ii) The statement on "nonlinear fitting to reduce noise" has been removed. The previous empirical fitting was not used to extract physical parameters. We now describe the data treatment as interpolation-based temperature-coordinate alignment (the added Fig. R2). This procedure only maps independently measured χ_{mol}(T) datasets onto a common temperature grid before calculating $\Delta\chi_{mol}/\chi_{mol(0)}$ (the revised Fig. 4d and Fig. S28b), because the actual temperature points recorded by PPMS do not coincide exactly between different measurements.

These revisions clarify the ZFC/FC presentation, remove the ambiguous fitting description, and make the calculation of $\Delta\chi_{mol}/\chi_{mol(0)}$ more transparent.

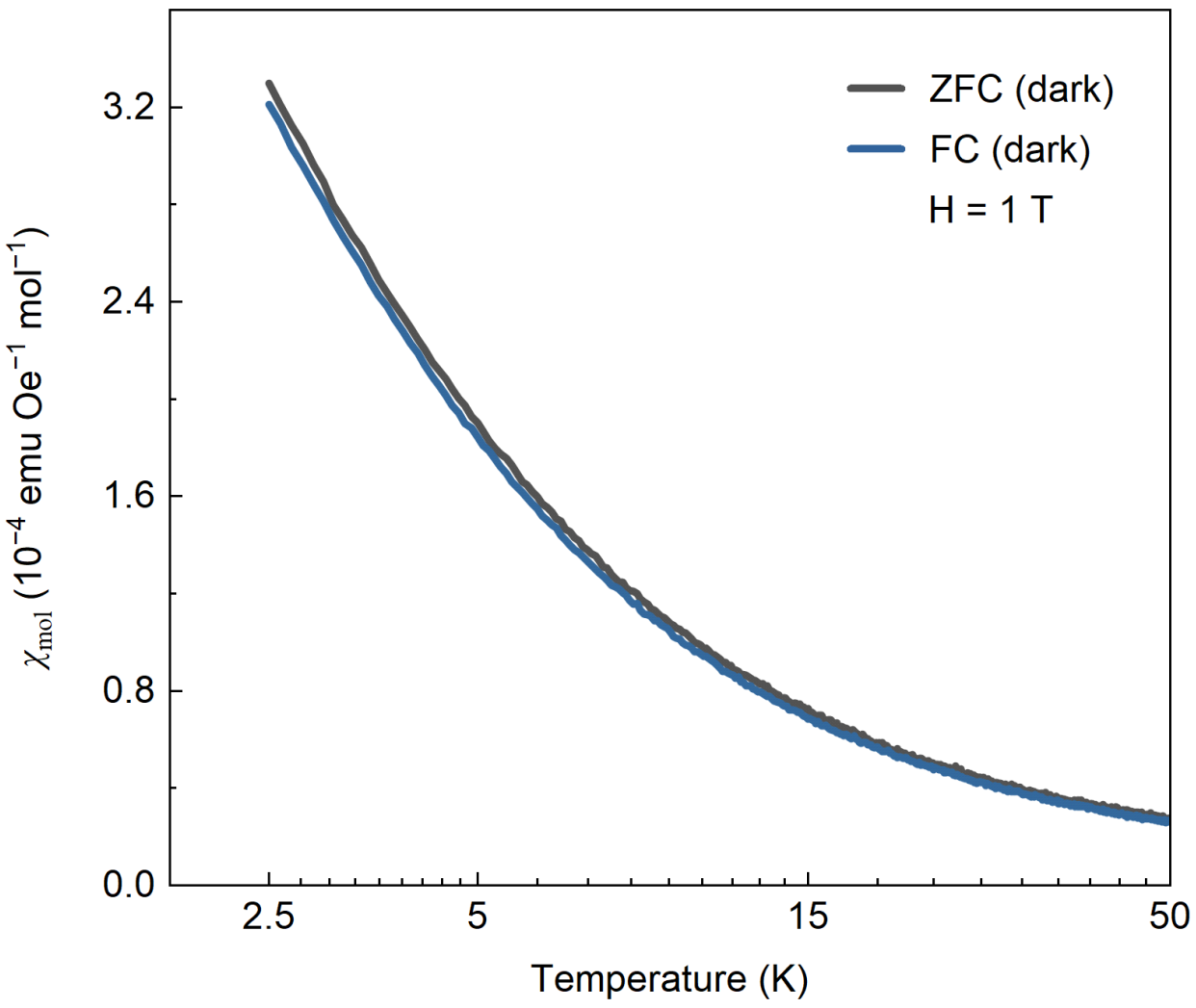

Figure S18 | Nearly overlapping ZFC and FC curves characteristic of paramagnetic behaviour. ZFC and FC curves of the 0.48 mm-thick alterlattice sample measured at H = 1 T under dark conditions. The two curves are nearly identical, indicating that the magnetic behavior of the sample under dark conditions is close to ideal paramagnetism.

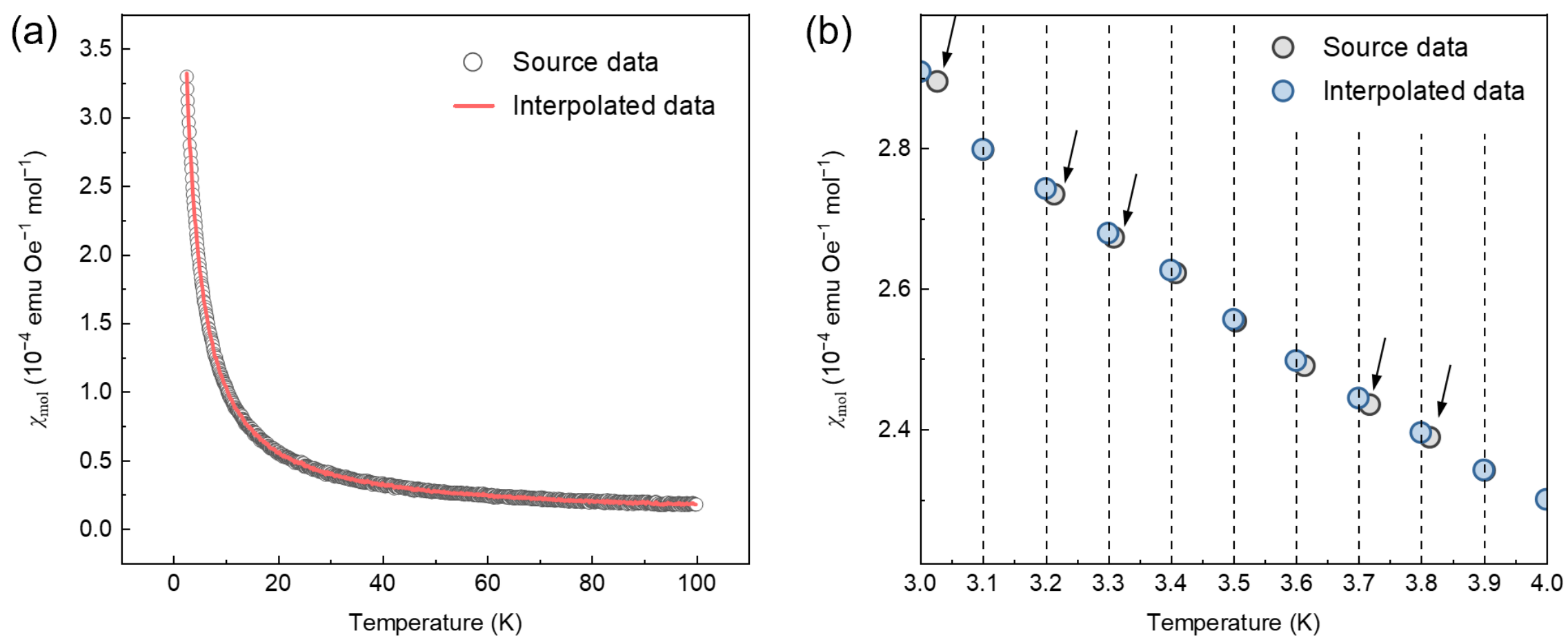

Figure R2. Linear interpolation of the ZFC curves, used to align temperature points in the raw data. The recorded temperatures are not exact integer values and do not strictly coincide between independent measurements. For example, a set temperature of 3 K may be recorded as 3.0012 K. This alignment allows a more accurate calculation of $\Delta\chi_{mol}/\chi_{mol(0)}$ by subtracting illuminated data from dark-state data at the same temperature grid. **(a)** The interpolated curve reproduced the experimental ZFC data with high fidelity, yielding a root-mean-square error (RMSE) of 1.1097×10^{-12} and a coefficient of determination (R^2) of 0.99945. Here, the RMSE quantitatively reflects the average deviation between the interpolated and experimental data, while the high R^2 value indicates excellent agreement in the overall variation trend, confirming that the interpolation introduced negligible distortion to the original dataset. **(b)** Enlarged comparison before and after interpolation, showing that the original temperature points do not exactly coincide at nominal values such as 3.1 or 3.2 K.

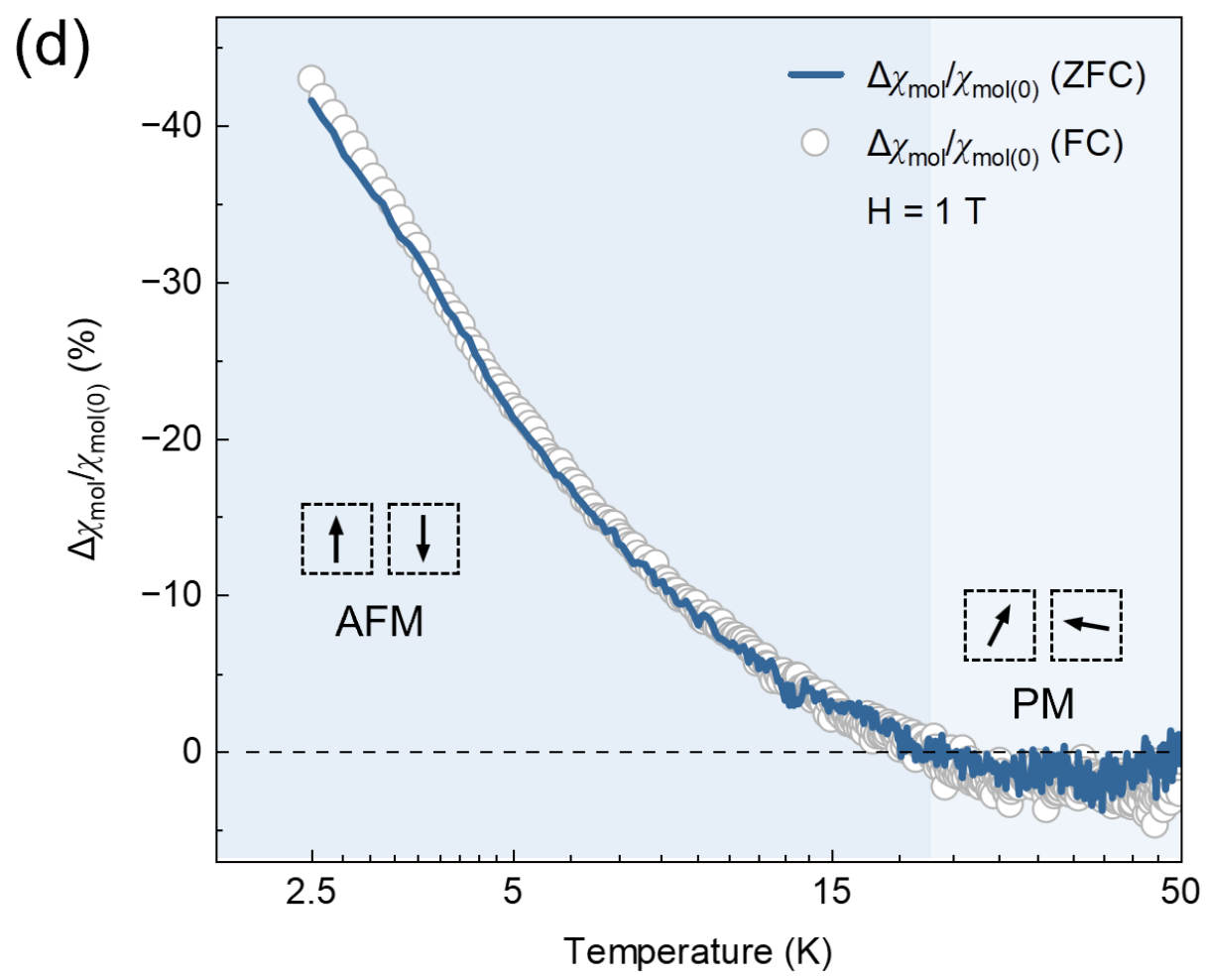

Fig. 4d. Temperature dependence of χ_{mol} relative change $\Delta\chi_{mol}/\chi_{mol(0)}$ at 1 T under 14 mW mm^{-2} illumination versus darkness, where $\Delta\chi_{mol} = \chi_{mol}(\text{light}) - \chi_{mol}(\text{dark})$ and $\chi_{mol(0)} = \chi_{mol}(\text{dark})$.

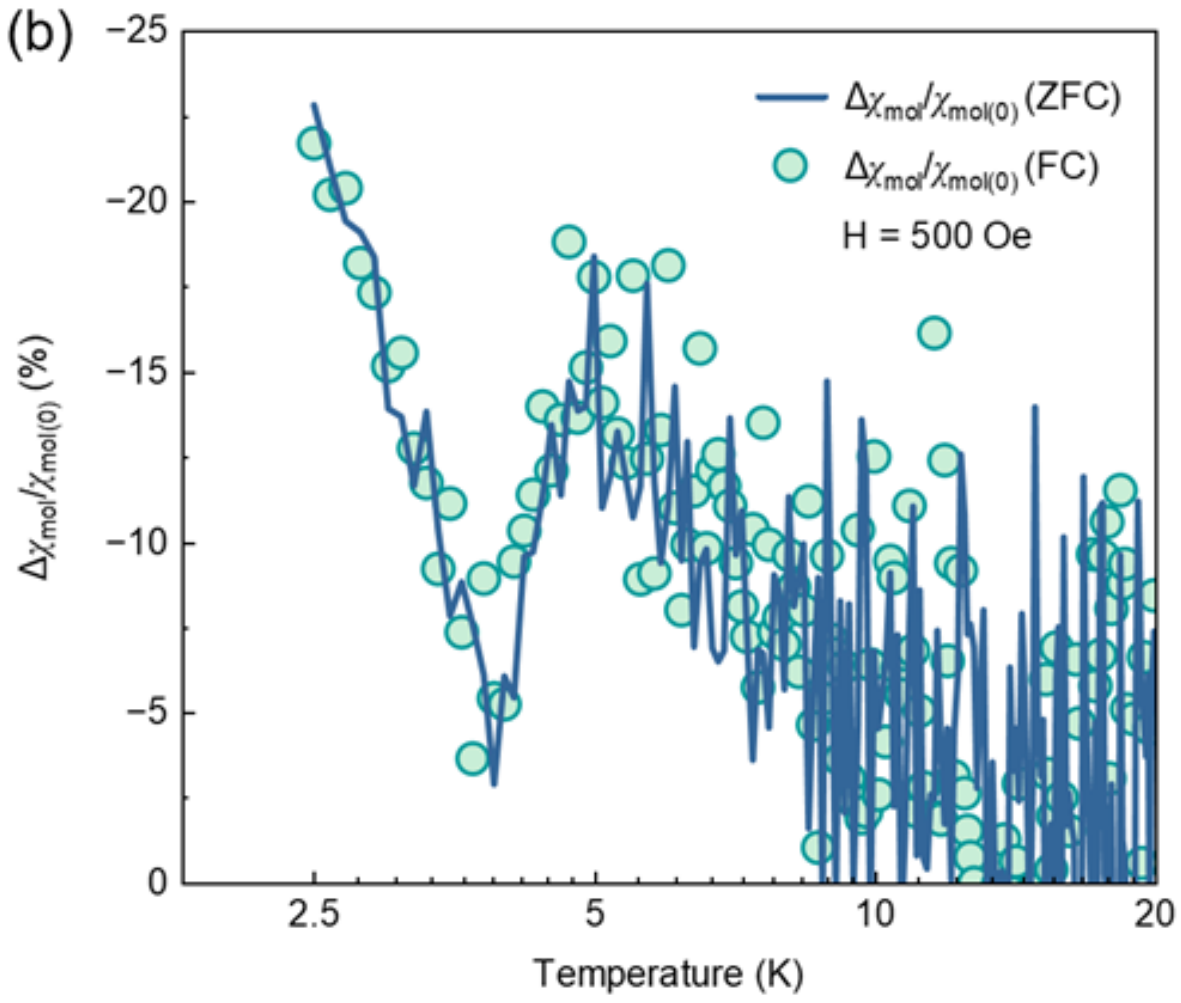

Figure S28b. Temperature dependence of $\Delta\chi_{mol}/\chi_{mol(0)}$ at 500 Oe under 14 mW mm^{-2} illumination (d = 0.48 mm sample).

Comments 1.13: "It is mentioned in line 338 that "the Ti $L_{2,3}$ edges shift to lower energy and the L2 t2g-split shoulder is enhanced". But the shift seems just fluctuation of noise in Fig.S24d. Please provide data with better statistics."

Response 1.13: We thank the reviewer for raising this concern. In the original manuscript, the Ti $L_{2,3}$ analysis was based on spectra extracted from a relatively localized region, which limited the statistical reliability of the observed spectral variations. To improve the robustness of the analysis, we reacquired the EELS data, selected spectra with improved signal-to-noise ratios, and performed independent repeat analyses from multiple regions.

The EELS panel previously shown in original Figure S24d has now been replaced by a revised dataset (the revised Fig. S19 and the revised manuscript, lines 331–334), and an additional independently repeated dataset has been added (the added Fig. S20). In both datasets, two representative regions were compared between an A-site-vacancy depleted region and an A-site-vacancy enriched region. The vacancy-enriched region exhibits a reduced La signal together with a slightly reduced O signal, indicating local A-site deficiency and possible oxygen deficiency. The Ti $L_{2,3}$ edge in the vacancy-enriched region shifts toward lower energy and shows a reduced Ti^{4+}-related eg feature,

indicating increased Ti^{3+} character.

These revisions demonstrate that the Ti $L_{2,3}$ spectral evolution is consistently correlated with local A-site-vacancy enrichment rather than random noise fluctuation.

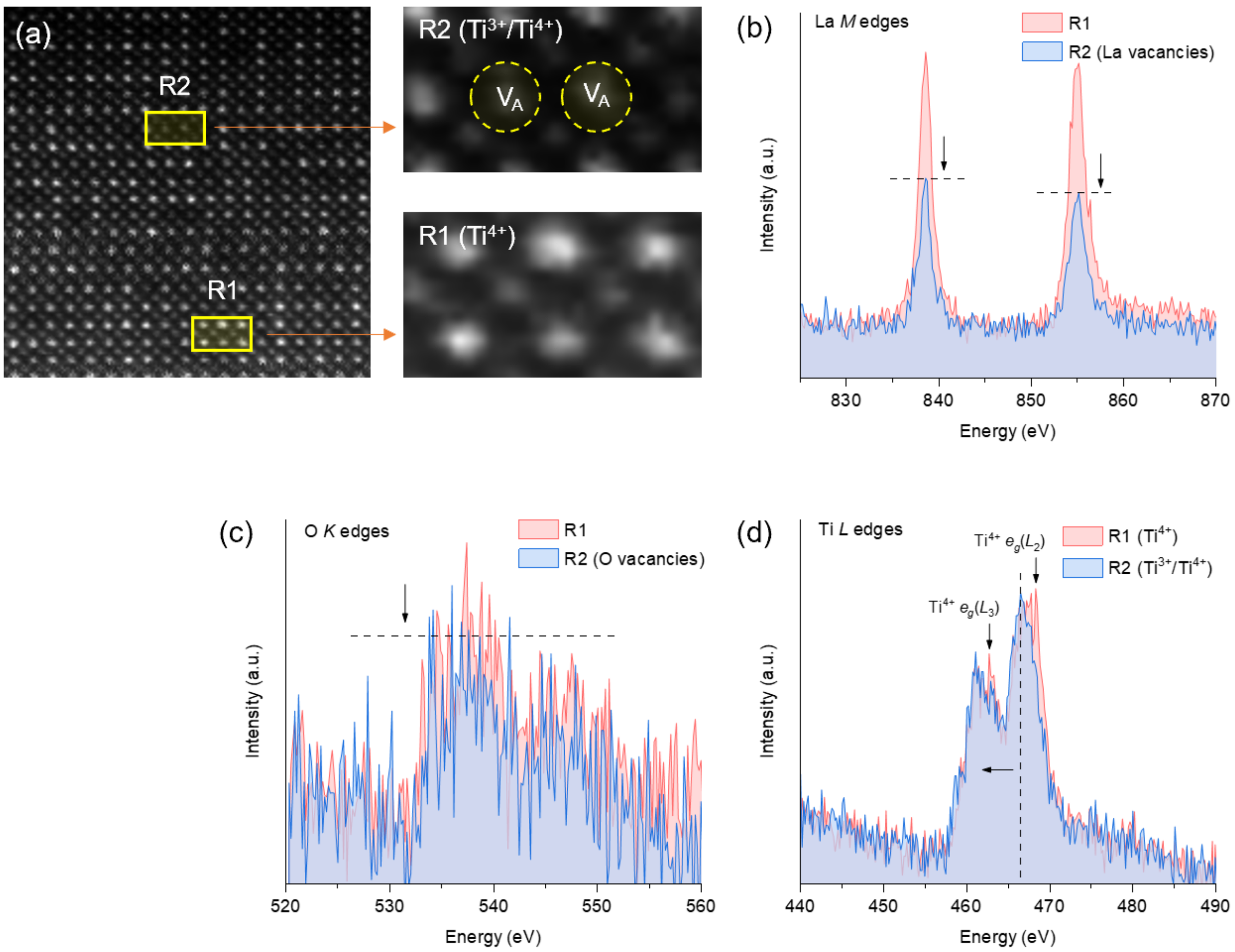

Figure S19 | EELS evidence for the spatial correlation of A-site vacancies, oxygen deficiency, and Ti^{3+} character. (a) HAADF-STEM image of the alterlattice in $La_{0.4}Ca_{0.4}TiO_{3-\delta}$ along the I-[001] zone axis. The A-site-vacancy-depleted region (R1) and A-site-vacancy-enriched region (R2) are marked. **(b)** La $M_{4,5}$ edge intensity spectra extracted from the R1 and R2 regions. The La intensity in R2 is significantly lower than that in R1, indicating La deficiency in R2. **(c)** O K edge intensity spectra extracted from the R1 and R2 regions. The O intensity in R2 is slightly lower than that in R1, suggesting possible oxygen deficiency in R2. **(d)** Ti $L_{2,3}$ edge spectra extracted from the R1 and R2 regions. Compared with R1, the e_g feature associated with Ti^{4+} is markedly reduced in R2, indicating the presence of partial Ti^{3+} character in R2.

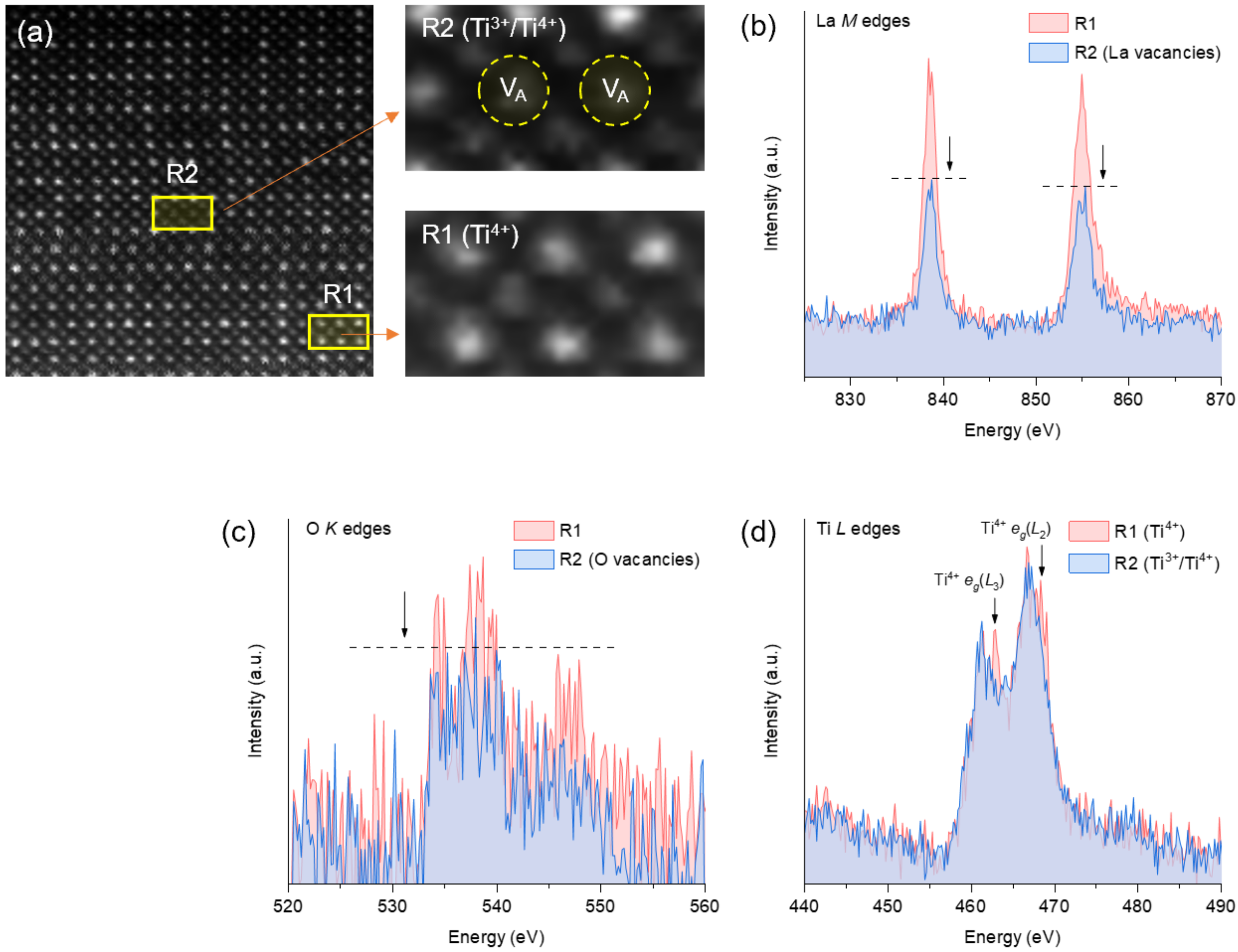

Figure S20 | Independent EELS evidence for the spatial correlation of A-site vacancies, oxygen deficiency, and Ti^{3+} character. (a) Another HAADF-STEM image of the alterlattice in $La_{0.4}Ca_{0.4}TiO_{3-\delta}$ along the I-[001] zone axis used for repeat verification. The A-site vacancy-depleted region (R1) and A-site vacancy-enriched region (R2) are marked. **(b)** La $M_{4,5}$ edge intensity spectra extracted from the R1 and R2 regions. The La intensity in R2 is significantly lower than that in R1, indicating La deficiency in R2. **(c)** O *K* edge intensity spectra extracted from the R1 and R2 regions. The O intensity in R2 is slightly lower than that in R1, suggesting possible oxygen deficiency in R2. **(d)** Ti $L_{2,3}$ edge spectra extracted from the R1 and R2 regions. Compared with R1, the eg feature associated with Ti^{4+} is markedly reduced in R2, indicating the presence of partial Ti^{3+} character in R2.

Comments 1.14: "The mechanism proposed in Fig.S25 seems not convincing enough. The excitation from valence band to defect levels and then from defect levels to the conduction band may have less possibility than direct excitation from valence band to conduction band. In this sense, wavelength dependence of the photo-induced demagnetization should be conducted to verify the proposed mechanism."

Response 1.14: We thank the reviewer for raising this point. In the original manuscript, the defect-assisted photoexcitation pathway was presented without sufficient wavelength-dependent evidence. To address this issue, we added wavelength-dependent photomagnetic measurements under 405, 473, and 532 nm illumination and revised the mechanism as a plausible defect-assisted scenario rather than a uniquely established excitation pathway.

(i) The added wavelength-dependent ZFC measurements (the added Fig. S23 and the revised manuscript, lines 350–354) show that 473 nm illumination produces the strongest negative photomagnetic response under identical conditions. At 2.5 K and 1 T, $\Delta\chi_{mol}/\chi_{mol(0)}$ reaches −41.6% under 473 nm illumination, compared with −25.2% for 405 nm and −37.7% for 532 nm. Moreover, within the temperature range of 2.5–50 K, 473 nm illumination

consistently exhibits the strongest influence on the magnetic behavior, indicating the most pronounced light-enhanced AFM-like correlations. Since the optical band gap is about 3.2 eV, 405 nm excitation is close to the band edge, whereas 473 and 532 nm correspond to sub-bandgap excitations. Therefore, the strongest response under 473 nm illumination cannot be explained solely by direct valence-band-to-conduction-band excitation.

(ii) We further added wavelength-dependent M–H measurements (the added Fig. S24 and the revised manuscript, lines 350–354). Compared with 405 and 532 nm illumination, the M–H curves under 473 nm illumination exhibit a more strongly compensated magnetic response over the 0–7 T range, consistent with enhanced AFM interactions partially compensating the local magnetic moments. In addition, within the approximately 0–0.78 T range, the photomagnetic response induced by 473 nm illumination continues to increase, whereas the responses under 405 and 532 nm illumination decrease monotonically with increasing magnetic field. This behavior indicates that the AFM interactions induced by 473 nm illumination are significantly stronger, which is consistent with the intrinsic robustness of AFM correlations against external magnetic-field perturbations.

(iii) Based on these results, we revised the mechanistic discussion (the revised manuscript, lines 352–354, 427–430) and original Fig. S25 (the revised Fig. S32a). The revised text states that oxygen-vacancy-related intragap states may contribute to sub-bandgap absorption, photocarrier generation, and trapping processes. The photoexcited carriers may further induce neighboring Ti^{4+} ions adjacent to intrinsic Ti^{3+} centers to form Ti^{3+}-like species with opposite spin alignment, thereby modifying local magnetic exchange interactions and generating metastable AFM correlations under illumination, which leads to the observed decrease in magnetic susceptibility.

These added measurements support the involvement of defect-related intragap states in the photomagnetic response and provide additional evidence for the proposed defect-assisted mechanism.

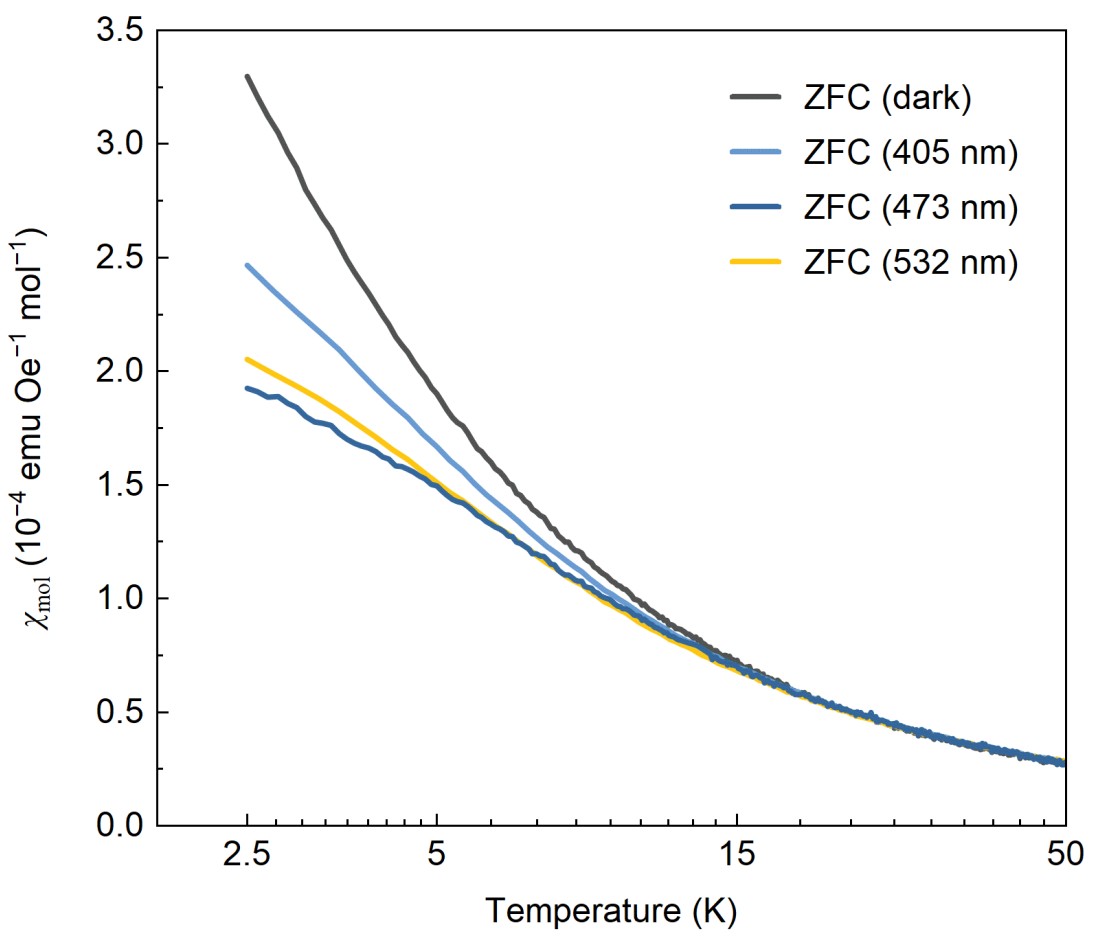

Figure S23 | Wavelength-dependent ZFC curves of the alterlattice. Comparison of the ZFC curves of the 0.48 mm-thick sample measured in dark conditions and under 405, 473, and 532 nm illumination. The ZFC curve measured under 473 nm illumination shows the most pronounced decrease compared with the dark-state curve, indicating that 473 nm light induces the strongest antiferromagnetic-like exchange interaction in the alterlattice. This wavelength selectivity supports a photocarrier-mediated mechanism for the formation of the light-induced magnetic response.

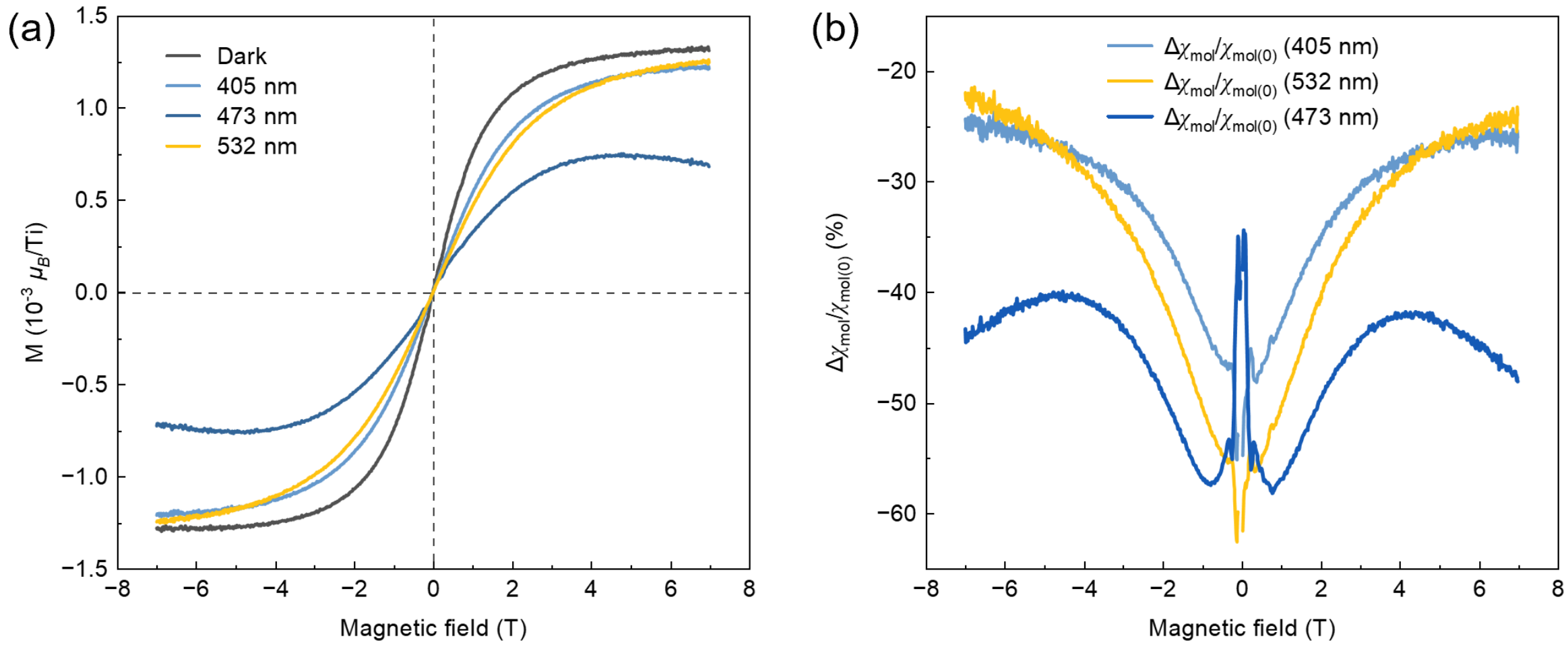

Figure S24 | Wavelength-dependent M–H curves of the alterlattice. The results show that 473 nm illumination produces the strongest effect. **(a)** Magnetic-field dependence of magnetization (μ_B/Ti) at 2 K for the 0.48 mm-thick sample under 405, 473, and 532 nm illumination (14 mW mm^{-2}), compared with the dark state. (b) M–H curves measured under 405, 473, and 532 nm illumination and under dark conditions at 2 K. **(c)** Field-dependent $\Delta\chi_{mol}/\chi_{mol(0)}$ curves under different illumination wavelengths, including 405, 473, and 532 nm.

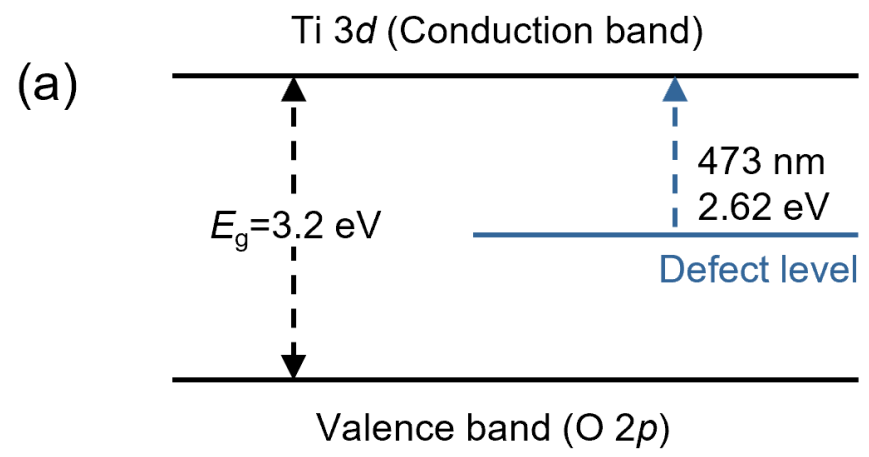

Figure S32a. Defect level excitation, in which electrons are photoexcited from oxygen vacancy related defect levels within the band gap into Ti 3*d* conduction states, followed by local trapping or redistribution that converts nearby Ti^{4+} to Ti^{3+} with unpaired spins.

Minor shortages, errors or typos:

Comments 1.15: "There are some abbreviations which are not clearly explained, which may be not obvious to ones who are not familiar with the techniques or terminologies. For example, "Obs." and "Ni I. S." in Fig.1d, Reduced χ2, wR, and GOF in Table S1,2, etc."

Response 1.15: We appreciate this constructive suggestion. In the original manuscript, several abbreviations were not explicitly defined, which could reduce readability for readers unfamiliar with the related techniques or refinement parameters. We have checked the manuscript and Supporting Information and added the missing definitions.

(i) The abbreviations in the revised Fig. 1e have been clarified in the figure caption (the revised manuscript, lines 106–107). "Obs." is now defined as the observed data, and "Ni I.S." is defined as the Ni internal standard used in the XRD measurements.

(ii) The refinement parameters in the relevant refinement tables defined in the corresponding table captions. "Reduced χ^2," "w_R," and "*GOF*" are now described as the reduced chi-square, weighted residual factor, and goodness of fit, respectively (the revised Supporting Information, lines 97–101, 122–126).

(iii) Other abbreviations appearing in the manuscript and Supporting Information have also been checked. Full terms

are now provided at their first occurrence, followed by consistent use of the abbreviated forms.

These revisions improve the clarity and accessibility of the figures, tables, and refinement results. The corresponding changes have been marked in the revised manuscript and Supporting Information.

Comments 1.16: In Fig.1b, I do not understand why the Ca/La atomic ratio should be plotted by log scale.

Response 1.16: We thank the reviewer for raising this question. In the original manuscript, the rationale for using a logarithmic scale in Figure 1b was not stated clearly.

Fig. 1b shows the A-site Ca/La stoichiometric ratio in $La_{2(1-x)/3}Ca_xTiO_3$, where Ca/La = $3x/[2(1-x)]$. At $x = 0.4$, Ca/La = 1, corresponding to equal Ca and La proportions at the A-site. The logarithmic scale was used to highlight the compositional region around Ca/La = 1, where the strongest A-site chemical competition occurs. We have revised Figure 1b. The positions of Ca/La = 1 and $x = 0.4$ are now explicitly marked.

Comments 1.17: "Dual-q" and "double-q" are both used, which I recommend to unify. some q's are not Italic.

Response 1.17: We thank the reviewer for pointing this out. In the revised manuscript, we have checked all occurrences of "dual-**q**" and "double-**q**" and unified the terminology as "double-**q**" throughout the revised manuscript, figures and Supporting Information.

We have also revised the formatting of the wave vector notation. Following the widely adopted convention in crystallography and condensed matter physics, reciprocal-space vectors are now consistently formatted in boldface (e.g., $\mathbf{q}$, $\mathbf{q}_1$, and $\mathbf{q}_2$) throughout the manuscript and Supporting Information. We note that this notation is also commonly used in publications in Nature Communications and related journals.

Comments 1.18: Sentence error in line 206.

Response 1.18: We thank the reviewer for pointing this out. We have carefully corrected the wording in the revised manuscript (line 278).

Comments 1.19: Line 282, duplicate words.

Response 1.19: We thank the reviewer for the careful correction. We have removed the duplicated wording in the revised manuscript (line 402).

Comments 1.20: Line 291~292, grammar error.

Response 1.20: We thank the reviewer for the careful correction. We have revised the grammatical errors in the revised manuscript (lines 410–412).

Comments 1.21: Fig.4f, the unit of moment seems to be wrong. Can the moment be converted to μB/Ti to give a impression of the local moment size?

Response 1.21: We thank the reviewer for pointing this out. In the original Figure 4f, the moment unit was not

properly presented. We have corrected the unit label and converted the magnetic moment to Bohr magnetons per Ti ion (μ_B/Ti) (the revised Fig. 4f).

The conversion was performed using the measured magnetization, sample mass, molar mass, and Ti content of $La_{0.4}Ca_{0.4}TiO_{3-\delta}$. The corresponding description has also been revised in the manuscript, so that the local moment size associated with Ti sites can be evaluated more directly.

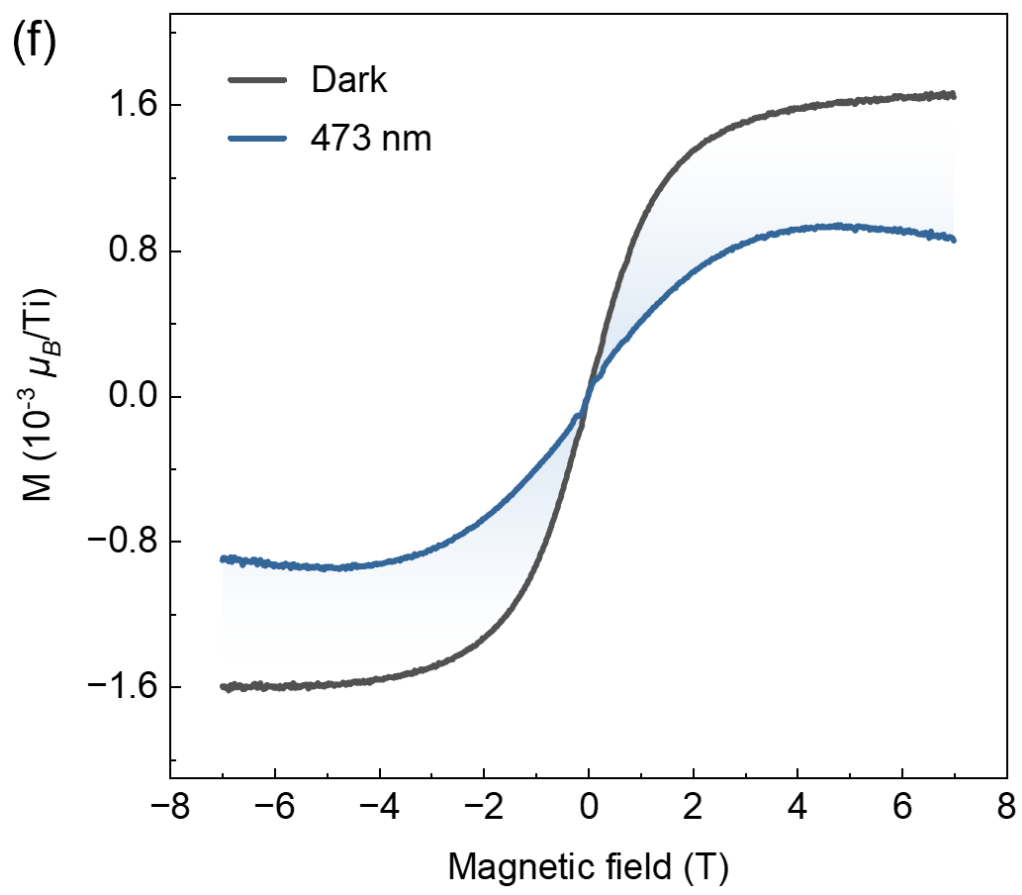

Fig. 4f. M–H curves measured under 473 nm illumination and in darkness at 2 K.

Comments 1.22: Thus, I cannot recommend publication of this manuscript before major revision and proper response to the abovementioned points.

Response 1.22: We thank the reviewer for the comprehensive and constructive comments on this work. In response to all the issues raised above, we have carried out substantial revisions and additions throughout the manuscript, including a systematic reorganization of the manuscript structure, further completion of key experimental results, and additional clarification and strengthening of the relevant physical mechanisms. At the same time, we have optimized the presentation of figures and the data-analysis procedures in order to improve the reliability and readability of the results. All revisions have been implemented point by point in the revised manuscript and are described in detail in the present response letter. We hope that these improvements adequately address the reviewer's concerns and significantly enhance the overall quality of the manuscript.

II. Reviewer #2 (Remarks to the Author)

Comments 2.1: In this work, the authors present an emergent structural motif referred to as the alterlattice, which manifests photoinduced short-range antiferromagnetic correlations below 19 K and is associated with two critical magnetic fields. The authors prepared La0.4Ca0.4TiO3-δ with the well known solid state reaction. The alterlattice here shown to act as a structured amplifier of photoinduced magnetism and provides a distinct materials pathway toward next-generation spin-based quantum information technology. The article needs some modifications to be published in Nat. communications.

Response 2.1: We thank the reviewer for the overall evaluation and recognition of this work. The reviewer has accurately summarized the central findings of the manuscript. We agree that there was still room for improvement in both the presentation and the supporting discussion, and we have therefore carried out systematic revisions and additions throughout the manuscript in accordance with the reviewer's comments.

Comments 2.2: Some language, typos and grammar issues need to be revised

Response 2.2: We thank the reviewer for pointing out the language, spelling, and grammatical issues in the manuscript. We have carried out a systematic language revision and sentence-by-sentence proofreading throughout the manuscript. The revision focused on correcting spelling and grammatical errors, improving sentence structure, unifying technical terms and abbreviations, and enhancing the overall coherence and readability of the manuscript.

Comments 2.3: Comparison with reported literature concerning miscellaneous perovskites and/ or titanates must be cited.

Response 2.3: We thank the reviewer for raising this point. In the original manuscript, the comparison with reported photomagnetic perovskites and titanates could be further strengthened. We have added a new comparison table (the added Table S1 and the revised manuscript, line 40) in the Supporting Information and cited the relevant literature.

(i) The added Table S1 now summarizes representative photoinduced magnetic responses in titanate perovskites, transition-metal oxides, and halide/oxide heterostructures. The comparison includes material type, response direction, and operating temperature. The added comparison shows that many reported titanates and perovskite oxides exhibit photoinduced magnetization enhancement or light-stabilized ferromagnetic behavior. In contrast, alterlattice ($La_{0.4}Ca_{0.4}TiO_{3-\delta}$) shows a rapid and reversible negative photomagnetic response in a bulk A-site-deficient titanate perovskite.

(ii) We have revised the related discussion to highlight this distinction (the revised manuscript, lines 39–46). Under 473 nm illumination at 2 K and 1 T, $La_{0.4}Ca_{0.4}TiO_{3-\delta}$ shows $\Delta\chi_{mol}/\chi_{mol(0)} \approx -51.7\%$ within ~1 s. This response is different from the more common photoinduced magnetization enhancement and supports the involvement of photo-induced local AFM correlations in the alterlattice. The effect remains observable below 19 K and gradually weakens with increasing temperature due to thermal fluctuations.

These revisions provide a clearer literature comparison and place the present photomagnetic response in the broader context of perovskites, titanates, and related photomagnetic materials.

Table S1 | Photomagnetic response of the alterlattice compared with representative materials. Comparison of photoinduced magnetic responses in representative perovskites, titanates, and related photomagnetic materials,

including material type, photomagnetic-response characteristics, and corresponding temperature ranges. Compared with previously reported systems, the alterlattice exhibits a rare and pronounced negative photomagnetic response in a bulk transition-metal oxide, characterized by a rapid light-induced decrease in macroscopic magnetic susceptibility. (detailed references are provided in the revised Supporting Information).

**Material**	**Material Type**	**Photomagnetic Response**	**Temperature Range**
$La_{0.4}Ca_{0.4}TiO_{3-\delta}$	Alterlattice (cation- and anion-deficient titanate)	473 nm induced AFM interactions	< 19 K
$SrTiO_{3-\delta}$	O-deficient titanate	400–500 nm induced persistent magnetization	< 18 K
$YTiO_3$	Mott titanate	9 THz enhanced FM	< 80 K
$Ni/BaTiO_3$	Heterostructures	532 nm enhanced magnetization	< 100 K
$La_{1.2}Sr_{1.8}Mn_2O_7$	Bilayer manganite	532 nm enhanced magnetization	30–200 K
$Gd_{0.55}Sr_{0.45}MnO_3$	Charge-ordered manganite	800 nm induced FM switch	<44 K
$La_{1/2}Sr_{1/2}MnO_{3-\delta}$	Manganite thin film	500 nm enhanced magnetization	<50 K
$Zn_{0.5}Co_{0.5}Fe_2O_4$	Spinel ferrite	532 nm induced magnetoresistance effects	<350 K

Comments 2.4: Is there any estimation of the crystallite size, morphology/microstructure, please add if available

Response 2.4: We appreciate this helpful suggestion. In the original manuscript, the crystallite/grain-size information and pellet microstructure needed to be supplemented. We have therefore added SEM characterization and grain-size analysis in the revised manuscript and Supporting Information.

(i) We added low-magnification SEM images and statistical grain-size analysis (the added Fig. S34, the revised manuscript, lines 453–455, and the revised Supporting Information, line 476–486). The sintered ceramic pellet consists of micrometer-sized polycrystalline grains, mainly distributed in the range of 5–20 μm. The grain-size distribution is well described by a log-normal function, giving D_{50} = 11.61 μm, σ_g = 1.71, and an arithmetic mean grain size of 11.74 ± 5.18 μm.

(ii) We added high-magnification SEM images to show the surface morphology and microstructure (the added Fig. S35, the revised manuscript, line 453–455, and the revised Supporting Information, line 493–501). The grains exhibit clear step-like or terrace-like surface features, consistent with a typical sintered titanate ceramic microstructure.

Overall, these additions provide a clearer characterization of the ceramic microstructure, including direct estimates of grain size and morphology, and establish a more complete structural basis for interpreting the observed photomagnetic behavior.

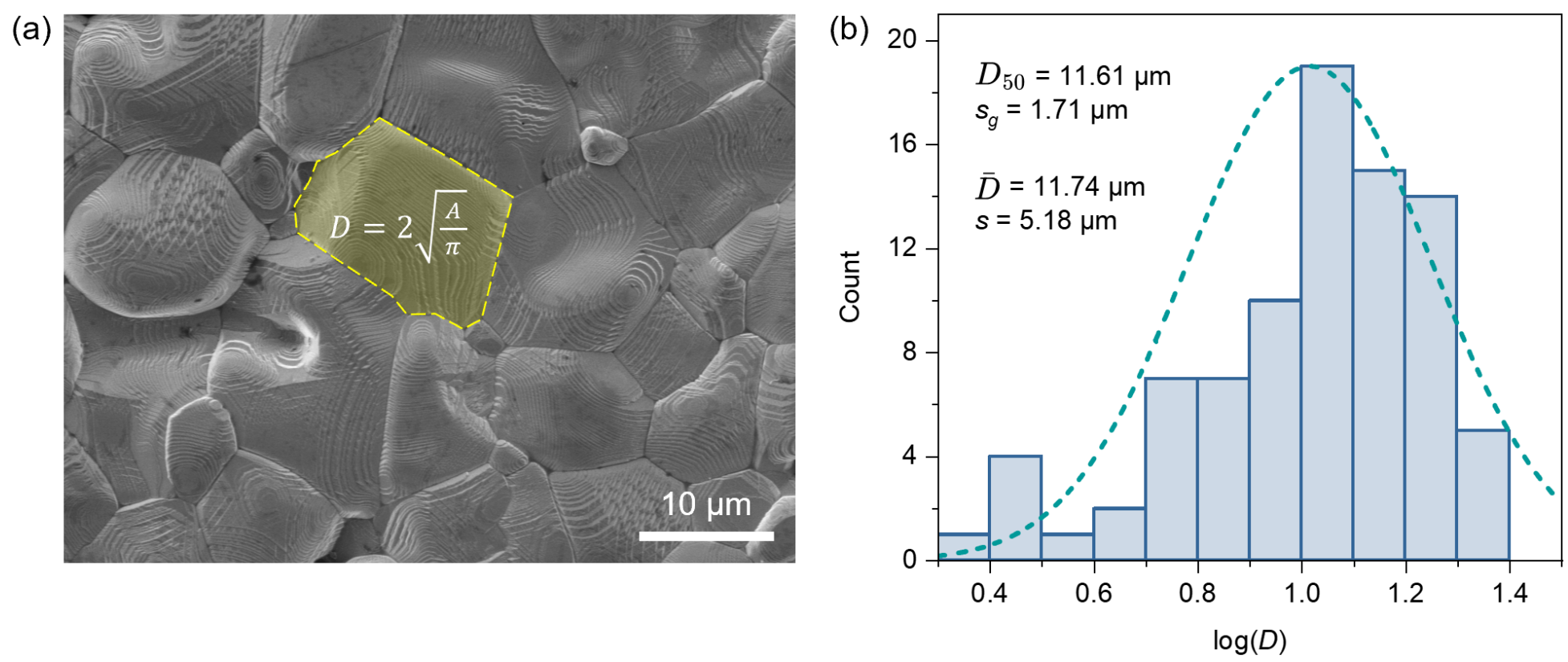

Figure S34 | SEM characterization and grain-size distribution analysis of the sintered alterlattice ceramic pellet used for photomagnetic measurements. (a) Low-magnification SEM image of the sintered ceramic pellet showing its dense polycrystalline microstructure. A representative grain is outlined to illustrate the calculation of the equivalent circular diameter using D = 2(A/π)^1/2, where A is the projected grain area. **(b)** Histogram of the logarithmic grain-size distribution fitted using a log-normal function. The extracted parameters include the median grain size D_{50}, geometric standard deviation σ_g, arithmetic mean grain size D, and standard deviation σ.

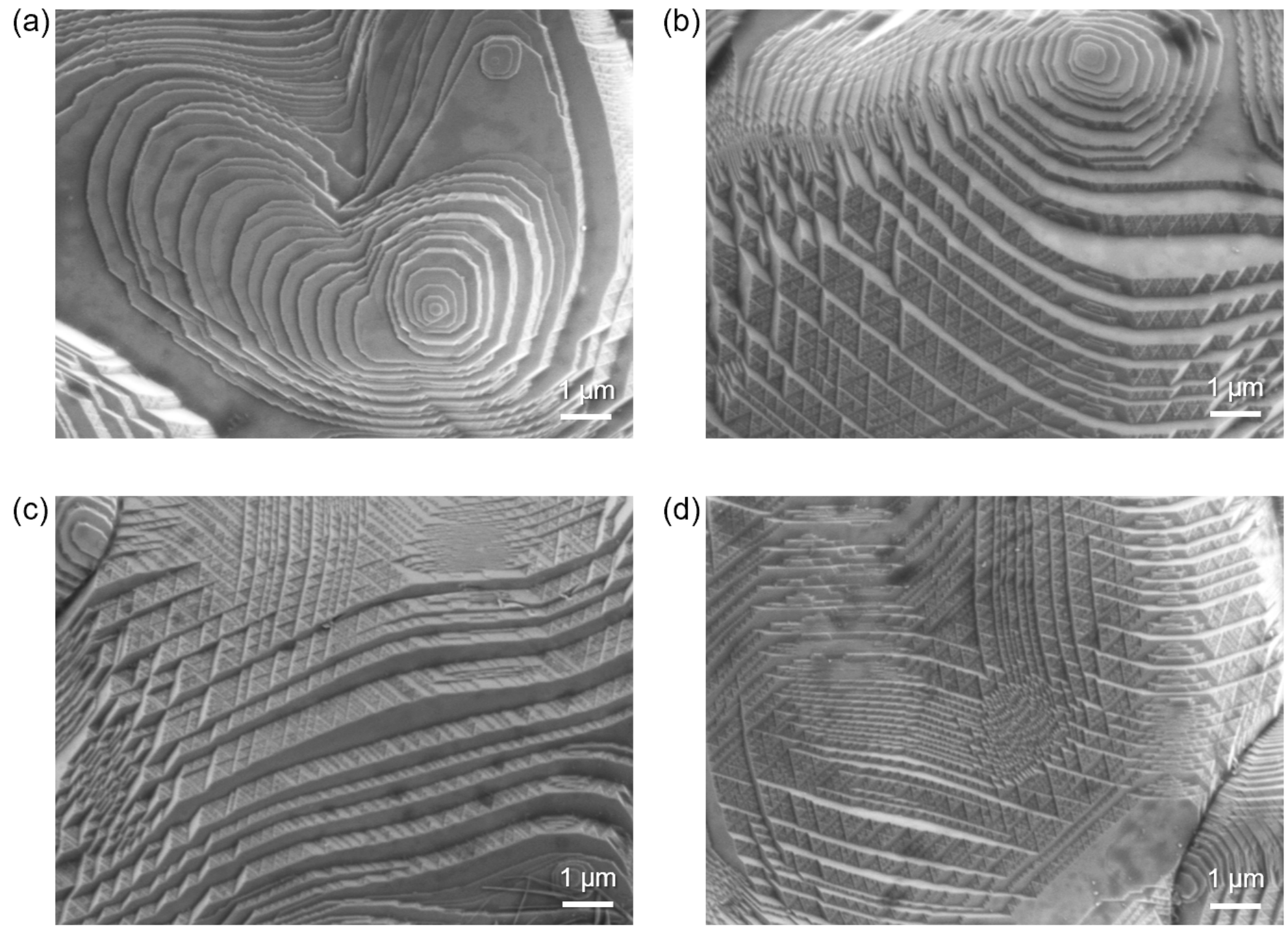

Figure S35 | SEM characterization of the step-like surface microstructure on grains of the sintered alterlattice ceramic pellet. (a-d) High-magnification SEM images collected from different grain regions show clear step-like, terrace-like, and ledge-terrace-type surface microstructures on the surfaces of the sintered ceramic grains.

Comments 2.5: Is there any evidence based data concerning the existence of Ti3+ ions, qualitative or quantitative analysis. We suggest to perform XPS for the obtained sample to find out binding energies of both Ti4+ and Ti3+

Response 2.5: We thank the reviewer for raising this point. In the original manuscript, the evidence for Ti^{3+} was not sufficiently summarized. In the revised manuscript and Supporting Information, we now provide multiple complementary qualitative and quantitative analyses to support the existence of Ti^{3+} in $La_{0.4}Ca_{0.4}TiO_{3-\delta}$.

(i) Following the reviewer's suggestion, we added Ti 2p XPS analysis (the added Fig. S21 and the revised

manuscript, lines 334–335). The Ti 2*p* spectrum, charge-corrected using C 1*s* = 284.8 eV, shows a lower-binding-energy shift relative to the Ti^{4+} reference together with a weak lower-valence component on the low-binding-energy side of Ti $2p_{3/2}$, qualitatively indicating the presence of Ti^{3+} species near the surface.

(ii) We also added Ti $L_{2,3}$ EELS analysis (the added Fig. S19d, Fig. S20d, and the revised manuscript, lines 329–332). In the A-site-vacancy-enriched region, the Ti $L_{2,3}$ spectrum exhibits a significant shift toward lower energy together with a suppressed e_g feature characteristic of Ti^{4+}, indicating partial Ti^{3+} character and providing local spectroscopic evidence for Ti^{3+} formation.

(iii) In addition to the qualitative spectroscopic evidence, we used Curie–Weiss fitting of the dark-state magnetic susceptibility to quantitatively estimate the Ti^{3+} concentration (the added Fig. S39 and the revised manuscript, lines 517–521). Since La^{3+}, Ca^{2+}, and O^{2-} do not contribute 3*d* local moments, the low-temperature paramagnetic response is mainly attributed to Ti^{3+} centers. The fitted Curie constant gives an estimated paramagnetically active Ti^{3+} content of ~0.27%.

Together, these qualitative and quantitative results provide mutually consistent evidence for the existence of a small amount of Ti^{3+} in $La_{0.4}Ca_{0.4}TiO_{3-\delta}$.

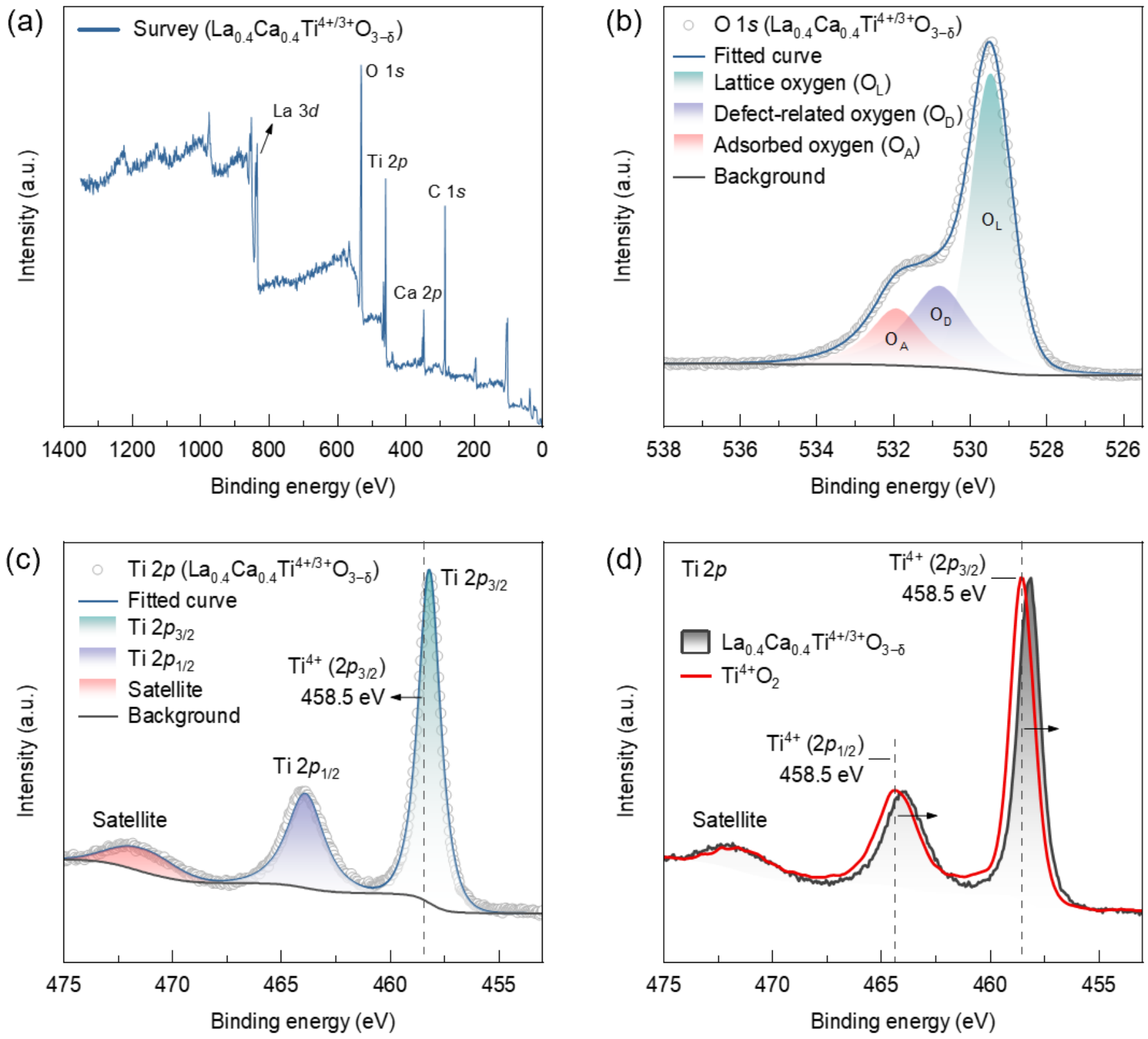

Figure S21 | XPS analysis of $La_{0.4}Ca_{0.4}TiO_{3-\delta}$. (a) Survey XPS spectrum showing the characteristic La 3*d*, Ca 2*p*, Ti 2*p*, O 1*s*, and C 1*s* signals. **(b)** High-resolution O 1*s* spectrum fitted with three components assigned to lattice oxygen (O_L), defect-related oxygen (O_D), and adsorbed oxygen (O_A). The defect-related oxygen component is consistent with oxygen nonstoichiometry and local defect structures in $La_{0.4}Ca_{0.4}TiO_{3-\delta}$. **(c)** High-resolution Ti 2*p* spectrum and peak-fitting result. The Ti 2*p* spectrum is dominated by Ti $2p_{3/2}$ and Ti $2p_{1/2}$ components, together with a weak satellite contribution. Compared with the typical Ti^{4+} $2p_{3/2}$ binding energy at approximately 458.5 eV, the Ti $2p_{3/2}$ peak in $La_{0.4}Ca_{0.4}TiO_{3-\delta}$ shifts toward lower binding energy, indicating partial reduction of Ti^{4+} and the presence of a small amount of Ti^{3+}. **(d)** Comparison of Ti 2*p* XPS spectra between $La_{0.4}Ca_{0.4}TiO_{3-\delta}$ and TiO_2, emphasizing the distinct lower-binding-energy shift of the Ti $2p_{3/2}$ and Ti $2p_{1/2}$ peaks in $La_{0.4}Ca_{0.4}TiO_{3-\delta}$.

Comments 2.6: It is also suggested to carry out a quantitative analysis to find out the exact oxygen stoichiometry as it plays a critical role in controlling the magnetic exchange interactions in AFM.

Response 2.6: We appreciate the reviewer's constructive suggestion. In the original manuscript, quantitative analysis of the oxygen stoichiometry was not sufficiently provided. We have therefore added Curie–Weiss analysis to estimate the paramagnetically active Ti^{3+} content and the corresponding oxygen stoichiometry, along with complementary spectroscopic evidence that qualitatively supports the presence of oxygen deficiency in the revised manuscript and Supporting Information.

(i) We estimated the Ti^{3+} content from Curie–Weiss fitting of the 2 to 100 K susceptibility measured for a $La_{0.4}Ca_{0.4}TiO_{3-\delta}$ ceramic pellet with a diameter of 3 mm and a thickness of 0.48 mm (the added Fig. S39, the revised manuscript, lines 517–521, and the revised Supporting Information, line 539–549). The fitting gives C = 0.00101 emu K mol^{-1} Oe^{-1} and $\theta = -0.87$ K, corresponding to $\mu_{eff} = 0.090\ \mu_B$ per formula unit. Assuming localized Ti^{3+} with $S = 1/2$ and $g \approx 2$, the estimated Ti^{3+} fraction is approximately 0.27% of Ti sites. Based on charge neutrality for $La_{0.4}Ca_{0.4}TiO_{3-\delta}$, this corresponds to $\delta \approx 1.4 \times 10^{-3}$.

(ii) We also added O *K* edge EELS analysis as complementary evidence (the added Fig. S19c, S20c, and the revised manuscript, lines 331–334). The results indicate that regions with enriched A-site vacancies exhibit slightly reduced O *K* edge intensity, suggesting a correlation between A-site vacancies and local oxygen deficiency.

(iii) The related discussion has been revised (the revised Fig. S32a and the revised manuscript, lines 352–354, 427–429). These additions provide quantitative oxygen-stoichiometry information and support the role of oxygen vacancies and Ti^{3+} centers in regulating local Ti–O–Ti exchange interactions and the photoinduced magnetic response.

In summary, the newly added Curie–Weiss analysis provides a quantitative estimate of the paramagnetically active Ti^{3+} content and the corresponding oxygen non-stoichiometry, while the EELS results provide complementary spectroscopic evidence for local oxygen deficiency in $La_{0.4}Ca_{0.4}TiO_{3-\delta}$.

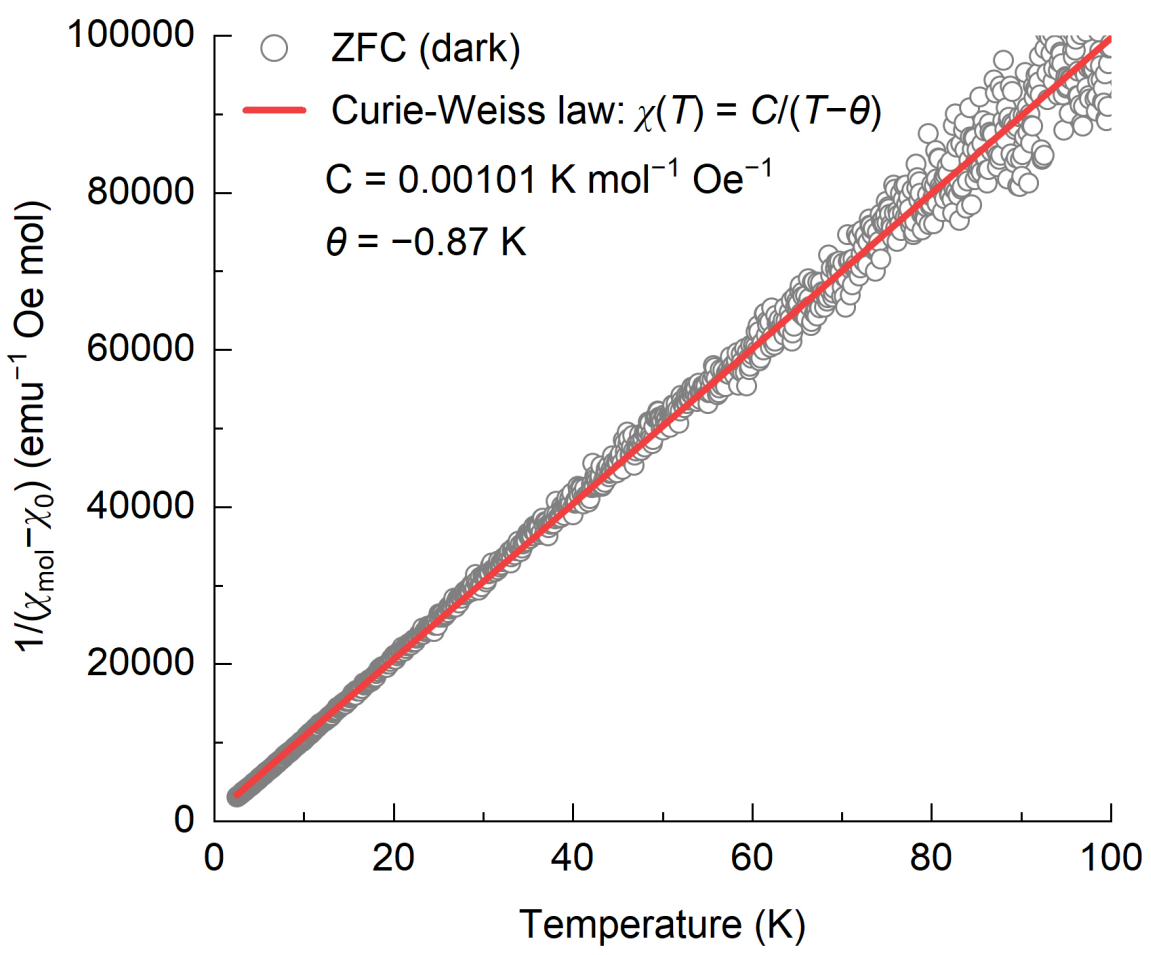

Figure S39 | Curie–Weiss fitting of the ZFC inverse susceptibility measured at 1 T in the dark for a $La_{0.4}Ca_{0.4}TiO_{3-\delta}$ ceramic pellet with a diameter of 3 mm and a thickness of 0.48 mm.

Comments 2.7: The field-dependent photo-response exhibits two characteristic fields at approximately 0.78 T and 4.24 T. This part needs extensive explanation.

Response 2.7: We appreciate this constructive suggestion. In the revised manuscript, we added further discussion on the possible physical meaning of the two characteristic fields observed in the field-dependent photo-response

(the revised manuscript, line 381–387). The field dependence of $\Delta\chi_{mol}/\chi_{mol(0)}$ may reflect a competition between photoinduced AFM interactions and magnetic-field-driven spin polarization.

(i) Below ~0.78 T, the negative photo-response increases with magnetic field. In this regime, the field is insufficient to fully polarize the localized spins, but can partially stabilize light-induced short-range AFM-correlated spin configurations by suppressing spin fluctuations. Weak magnetic fields may also facilitate carrier transport and suppress carrier recombination, further enhancing the photo-response.

(ii) Between ~0.78 and 4.24 T, the magnitude of $\Delta\chi_{mol}/\chi_{mol(0)}$ decreases with increasing field, suggesting that field-induced spin canting or spin polarization progressively counteracts the photoinduced AFM-correlated state.

(iii) Above ~4.24 T, the negative photo-response increases again, suggesting a re-entrant photomagnetism under high fields. We cautiously attribute this effect to field-enhanced correlation phenomena in a strongly spin-polarized background, such as magnetic-field-assisted carrier delocalization and negative magnetoresistance-related transport enhancement.

These revisions clarify the nonmonotonic field dependence and highlight the competition between photoinduced AFM correlations and magnetic-field-induced spin polarization.

Comments 2.8: Is there any correlation between Ti4+-O-Ti3+ chemical bond, photoinduced negative magnetism and magnetooptical properties of such perovskite??? if there is please discuss.

Response 2.8: We thank the reviewer for pointing this out. In the original manuscript, the relationship among Ti^{4+}–O–Ti^{3+} mixed-valence connections, photoinduced negative magnetism, and possible magneto-optical behavior was not sufficiently discussed. We have added a related discussion in the revised manuscript.

(i) Under dark conditions, the system is dominated by Ti^{4+}–O–Ti^{3+} mixed-valence linkages associated with isolated Ti^{3+} local moments. Here, Ti^{4+}–O–Ti^{3+} represents a defect-related mixed-valence pathway rather than a conventional super-exchange interaction, because Ti^{3+} is magnetic ($3d^1$) whereas Ti^{4+} is nonmagnetic ($3d^0$). As a result, these linkages mainly contribute paramagnetic-like local states instead of stable AFM exchange pairs (the revised manuscript, line 423–427).

(ii) Under illumination conditions, photogenerated carriers can be trapped near Ti^{4+} sites adjacent to Ti^{3+}/oxygen-vacancy centers, forming transient Ti^{3+}-like small-polaron states. This process locally converts part of the Ti^{4+}–O–Ti^{3+} network into Ti^{3+}–O–Ti^{3+} exchange units, thereby enhancing short-range AFM-like correlations and reducing the magnetic susceptibility. The Ti^{3+}/Ti^{4+} mixed valence and oxygen-vacancy-related defect states may provide sub-bandgap excitation channels. Since 473 nm light has a photon energy of ~2.62 eV, below the optical band gap of ~3.2 eV, the photo-response is more likely associated with defect-mediated excitation rather than only direct valence-band-to-conduction-band excitation (the revised Fig. S32a and the revised manuscript, line 427–434).

These revisions clarify that the Ti^{4+}–O–Ti^{3+} pathway acts as a defect-assisted photoexcitation and local exchange-reconstruction channel, linking sub-bandgap optical absorption, photoinduced short-range AFM-like correlations, and the negative photomagnetic response.

Comments 2.9: rewrite the conclusion

Response 2.9: We thank the reviewer for this suggestion. We have revised the Conclusion to clarify the definition of the alterlattice and to present a more evidence-based interpretation of the light-controlled magnetic response. We also refined the discussion of the magnetic mechanism and added a more cautious outlook on future structural and

magnetic investigations.

The lines 485–499 of the revised manuscript: “In conclusion, we establish the alterlattice as a structural route for generating optically tunable short-range magnetism in an A-site deficient perovskite oxide. The term alterlattice describes a structurally coherent lattice state in which the average framework is locally altered by superstructure domains, making this lattice motif distinct from a conventional long-range superlattice, a twin crystal, a simple phase mixture or phase separation. The alterlattice is defined by an intergrown nanoscale domain network arising from a pair of orthogonal superstructure modulation vectors, which produces a grid-like local strain field. Under sub-bandgap illumination, this constrained strain environment promotes low-dimensional antiferromagnetic excitations with an extremely small gap energy, indicating that the photo-induced state is governed primarily by short-range spin correlations confined within the structural domains. These findings point to local structure and strain-network engineering in A-site deficient oxides as an effective strategy for realizing light-controlled magnetic responses. Future studies combining techniques such as three-dimensional electron tomography, NV-center quantum magnetometry, magnetic pair distribution function analysis and first-principles spin-exchange simulations may help further resolve the 3D structural topology of the alterlattice, as well as the dynamical evolution and microscopic exchange pathways of the photo-induced magnetic phase.”

III. Reviewer #3 (Remarks to the Author)

Comments 3.1: The authors report a so-called "alterlattice" structure with giant negative photomagnetic effect. The authors present many high-quality HAADF-STEM images as well as detailed structural analyses, and the properties are also noteworthy. However, there are several issues need to be carefully addressed before the paper can be accepted for publication.

Response 3.1: We thank the reviewer for the overall evaluation and constructive comments on this work. We are pleased that the reviewer recognizes the quality and potential significance of both the structural characterization and the physical-property results presented in this study. In response to the reviewer's detailed comments, we have systematically revised the manuscript and provided point-by-point responses. We believe that these revisions have significantly improved the scientific rigor, logical coherence, and clarity of presentation of the manuscript. We sincerely thank the reviewer again for the careful evaluation and valuable suggestions, which have played a critical role in improving this work.

Comments 3.2: The description of synthesis control is insufficient. The authors should elaborate on the factors that give rise to this structure and on how the photomagnetic response varies among the different phases. Such details would greatly clarify the structure–property relationships.

Response 3.2: We thank the reviewer for this important suggestion. In the original manuscript, the synthesis-control factors and the structure–property relationship across different phases were not sufficiently described. We have therefore supplemented structural analyses and photomagnetic response measurements for different phases in the $La_{2(1-x)/3}Ca_xTiO_3$ system to demonstrate how the alterlattice structure forms through tuning the A-site vacancy concentration and the chemical competition between A-site cations, and to directly correlate the structural characteristics of the alterlattice with the negative photomagnetic response observed in this work.

(i) We added further synthesis-control details (the revised manuscript, lines 502–510), including precursor pretreatment, high-energy ultrasonication, vacuum drying, and controlled thermal processing. These procedures improve precursor homogeneity and help stabilize reproducible finite-correlation-length A-site vacancy/cation ordering.

(ii) We added a systematic $La_{2(1-x)/3}Ca_xTiO_3$ compositional series with x = 0, 0.2, 0.7, and 1.0 for comparison with the alterlattice composition x = 0.4 (the added Fig. R1, Fig. S3, and the revised manuscript, lines 130–141). The results show that Ca substitution tunes the A-site vacancy concentration, local strain, and structural phase competition. We further added XRD comparisons of the superstructure reflections and ordering correlation lengths across the series. The sharp superstructure reflection in $La_{2/3}TiO_3$ becomes broadened and diffuse near $La_{0.4}Ca_{0.4}TiO_{3-\delta}$, while the apparent ordering correlation length decreases from ~41.5 nm to ~3.4 nm, indicating the evolution from long-range A-site-vacancy ordering in the $La_{2/3}TiO_3$ parent phase to nanoscale short-range ordered domains in the alterlattice.

(iii) We added phase-dependent photomagnetic measurements under identical conditions (the added Fig. S4 and the revised manuscript, lines 146–151). The negative photomagnetic response is weak in $La_{2/3}TiO_3$, increases with Ca substitution, reaches its maximum near the alterlattice composition, and becomes much weaker in the Pbnm phase region. This trend indicates that the response is enhanced as the superstructure correlation length decreases to the nanoscale.

Based on these results, we further revised the discussion of the alterlattice formation mechanism and its relationship

to the observed photomagnetic response (the revised manuscript, line 173–178). The revised manuscript clarifies that the alterlattice originates from competition among A-site vacancy ordering, Ca/La substitution, local strain, and structural phase evolution, and that the pronounced negative photomagnetic response is closely associated with the nanoscale short-range ordered alterlattice (the revised manuscript, line 163–173).

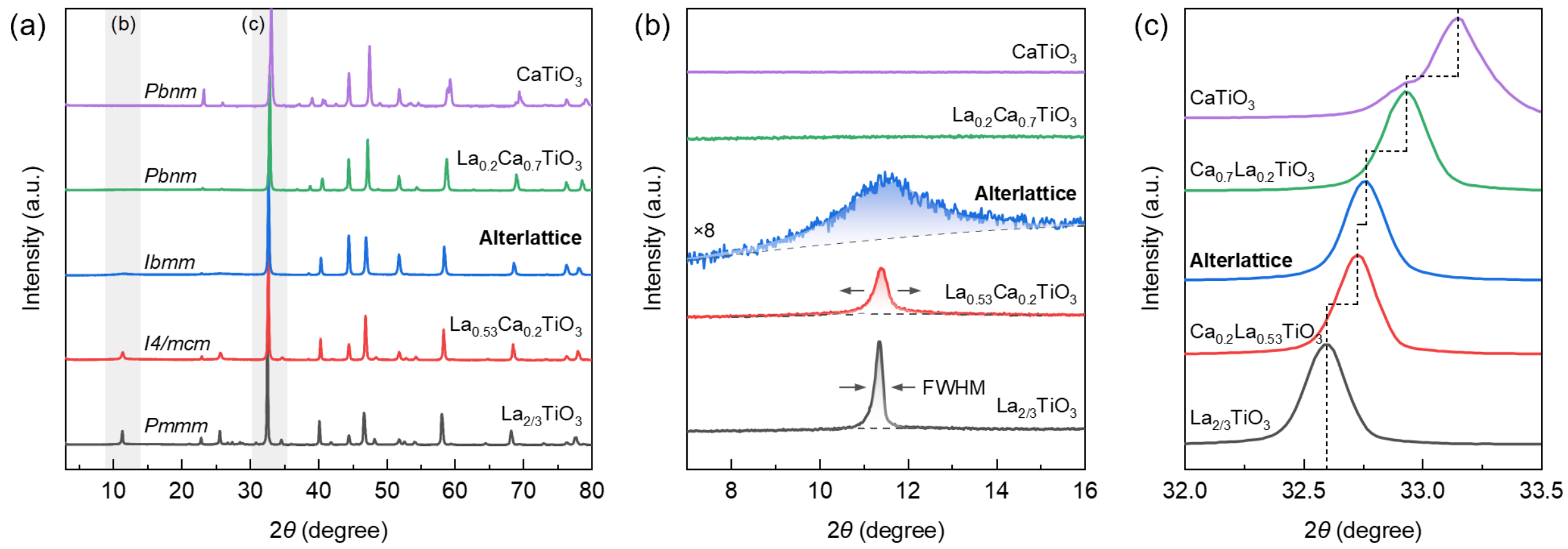

Figure R1. Structural evolution towards the alterlattice (x = 0.4) in the $La_{2(1-x)/3}Ca_xTiO_3$ series. (a) Full-range XRD patterns (2θ = 3–80°) reveal the emergence of the alterlattice upon Ca incorporation into the parent phase, driven by A-site chemical disorder and crystallographic phase competition. **(b)** Low-angle XRD patterns (2θ = 7–16°), featuring Scherrer analysis of the superstructure reflection near $2\theta \approx 12°$ to determine the full width at half maximum. **(c)** High-resolution XRD patterns (2θ = 32–33.5°), tracking the progressive shift of the strongest main-lattice reflection to higher angles with increasing Ca content.

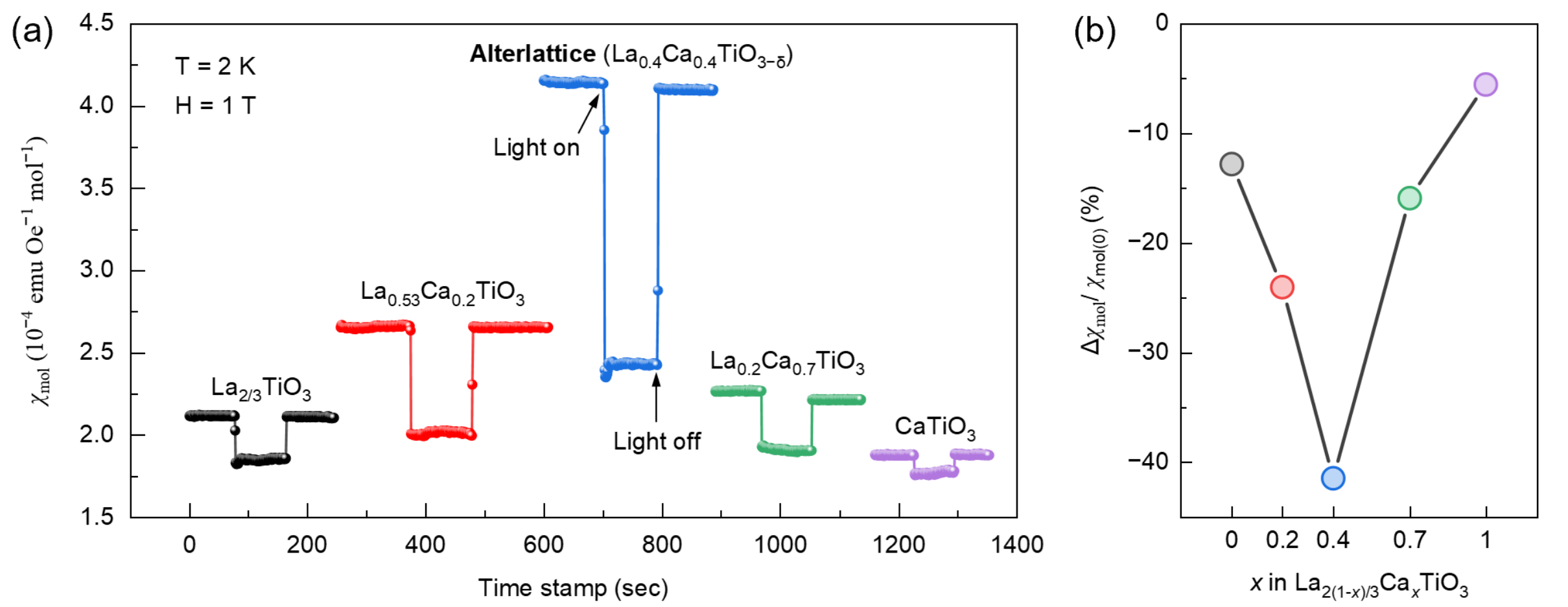

Figure S4 | Light on/off photomagnetic measurements of the $La_{2(1-x)/3}Ca_xTiO_3$ series. Time evolution of the molar magnetic susceptibility (χ_{mol}) at 2 K under 1 T using 473 nm illumination with a light intensity of 10 mW mm^{-2}. The measurements show a negative photomagnetic response, characterized by a rapid decrease in χ_{mol} under illumination and recovery after the light is turned off. **(a)** Negative photomagnetic responses of $La_{2(1-x)/3}Ca_xTiO_3$ with x = 0, 0.2, 0.4, 0.7, and 1.0. **(b)** Comparison of $\Delta\chi_{mol}/\chi_{mol(0)}$ for different compositions under identical conditions, where $\Delta\chi_{mol}$ is the difference between χ_{mol} under illumination and χ_{mol} in the dark state, and $\chi_{mol(0)}$ is the dark-state value before illumination.

Comments 3.3: In Fig. 1a, the authors show an idealized schematic of a 3D network composed of orthorhombic short-range domains. This schematic drawing is overly idealized, the actual intergrowth domains are more disordered. I suggest revising the schematic to better reflect the actual arrangement.

Response 3.3: We thank the reviewer for raising this point. In the original Figure 1a, the three-dimensional network schematic was overly idealized and could imply a highly regular arrangement of identical domains. We have redrawn the schematic drawing (the revised model of alterlattice in Fig 1a).

The revised schematic now shows a more nonuniform interpenetrating network of orthogonal short-range domains, with local disorder, defect-rich regions, and partially ordered A-site-vacancy arrangements. We also reduced the regularity of the domain size and interface distribution to better reflect the experimentally observed short-range and disordered intergrowth features. These revisions make Figure 1a more consistent with the HAADF-STEM, FFT/IFFT, and local structural analyses.

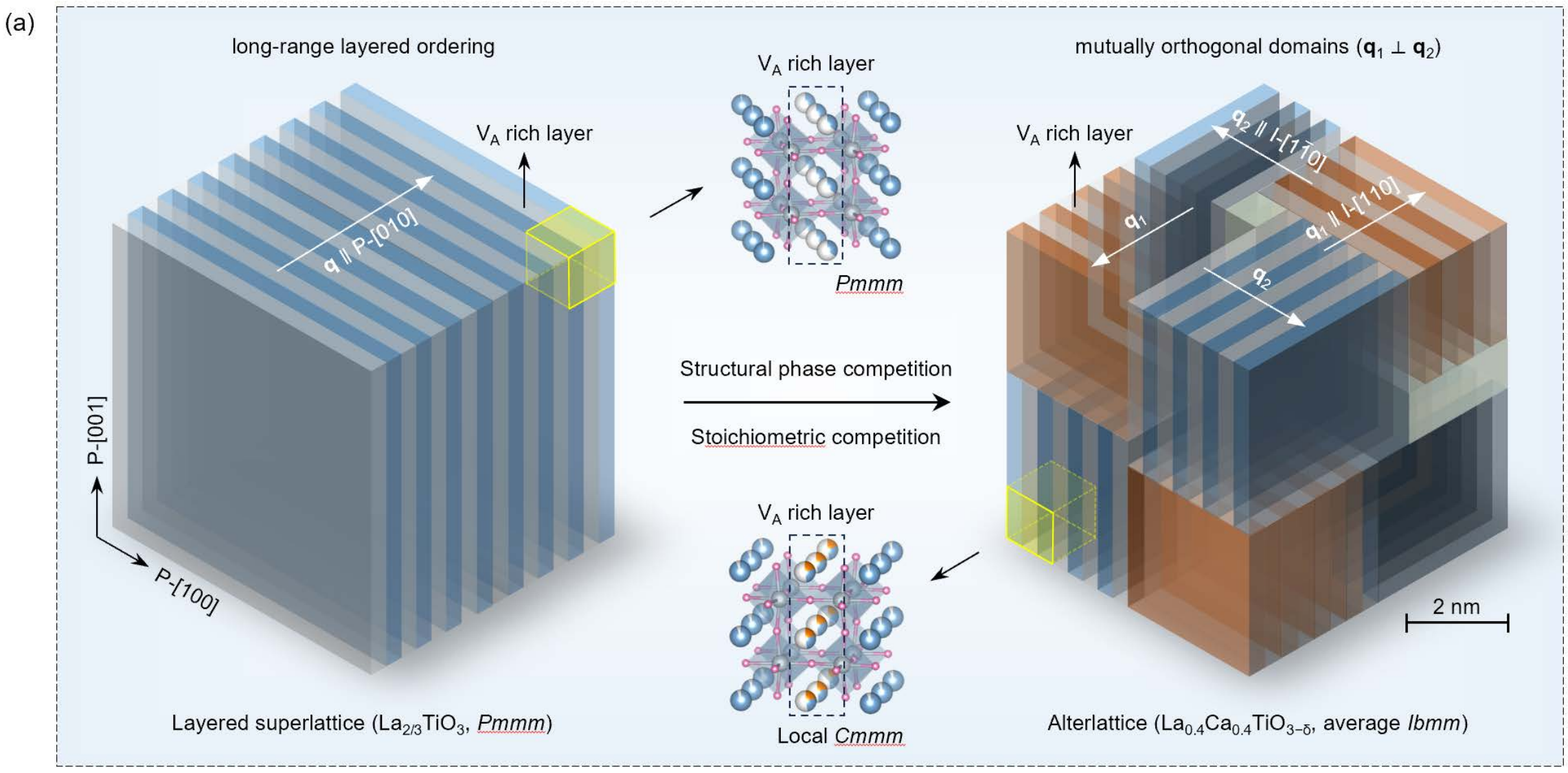

Fig. 1a. Schematic illustrating the competition between *Ibmm* and *Pmmm* phases, which fragments long-range A-site vacancy (V_A) ordering into an interwoven 3D network of *Cmmm*-like domains, termed the alterlattice. The alterlattice model represents an approximately 6 nm cubic structural region, with grayish green areas indicating the A-site disorder.

Comments 3.4: The authors indexed the structure as the Ibmm phase, and regard the additional diffraction spots from superstructures. The existence of the Ibmm structure was mainly confirmed by XRD analysis (Fig. 1d). However, the diffraction peak positions corresponding to the Cmmm structure are annotated in Fig. 1c but not in Fig. 1d. Would the 90° rotational intergrowth of Cmmm also give rise to diffraction peaks similar to those in Fig. 1d? Further superstructure construction and simulation are needed to verify this point.

Response 3.4: We thank the reviewer for raising this key issue. In the original manuscript, the distinction between the *Ibmm* average structure and the *Cmmm* local superstructure was not sufficiently clear. We have revised the related figures and structural analysis, and added superstructure schematics and XRD simulations to clarify the *Ibmm*/*Cmmm* relationship and the effect of 90° rotational intergrowth.

(i) We refined the 2θ = 3–80° XRD pattern using the *Ibmm* average structure (the revised Fig. 1e and revised manuscript, lines 152–155). The *Ibmm* model reproduces the sharp main Bragg reflections, confirming the long-range average symmetry. We also marked the additional reflections that cannot be indexed by *Ibmm*, since they require a local superstructure contribution.

(ii) Therefore, we performed separate low-angle XRD analysis and *Cmmm* superstructure simulations (the added Fig. 1f and revised manuscript, lines 154–155). The broadened reflections near $2\theta = 11.5°$ and 25.6° are consistent with A-site vacancy ordering, but are assigned to short-range local domains. We further simulated the combined diffraction pattern by superimposing powder XRD contributions from micron-sized *Ibmm* grains and nanoscale *Cmmm* domains (the added Fig. S5 and revised manuscript, lines 155–159), which successfully reproduced the experimentally observed alterlattice diffraction features. Importantly, unlike the simulated long-range *Cmmm* pattern, the experimental diffraction pattern shows no *Cmmm* reflections above 30°. These results indicate that the alterlattice retains *Ibmm* symmetry on average, while local A-site-vacancy ordering only produces the C-(001) and (011) superstructure reflections. The limited distortion correlation length prevents the formation of a true long-range *Cmmm* phase with additional high-angle reflections. Thus, the symmetry-breaking distortion remains confined to finite local domains and does not develop into a macroscopic order parameter that changes the average structure. Moreover, the orthogonal intergrowth of *Cmmm* domain variants further averages out their anisotropic distortion components, allowing the Bragg-averaged lattice to retain *Ibmm* symmetry rather than forming a true long-range *Cmmm* phase.

(iii) We further simulated 90°-rotated *Cmmm* domains and their combined diffraction contribution (the added Fig. S6 and revised manuscript, lines 159–162). Orthogonally oriented short-range domains with distributed ordering lengths generate partially overlapping broadened low-angle reflections in powder XRD, while the *Ibmm* model accounts for the sharp main reflections.

These revisions clarify that the alterlattice is an *Ibmm* average lattice containing *Cmmm* A-site-vacancy ordered domains with finite correlation length. The rotational-intergrowth model is consistent with the broadened superstructure reflections and is further supported by HAADF-STEM, FFT/IFFT, SAED, and local compositional analyses.

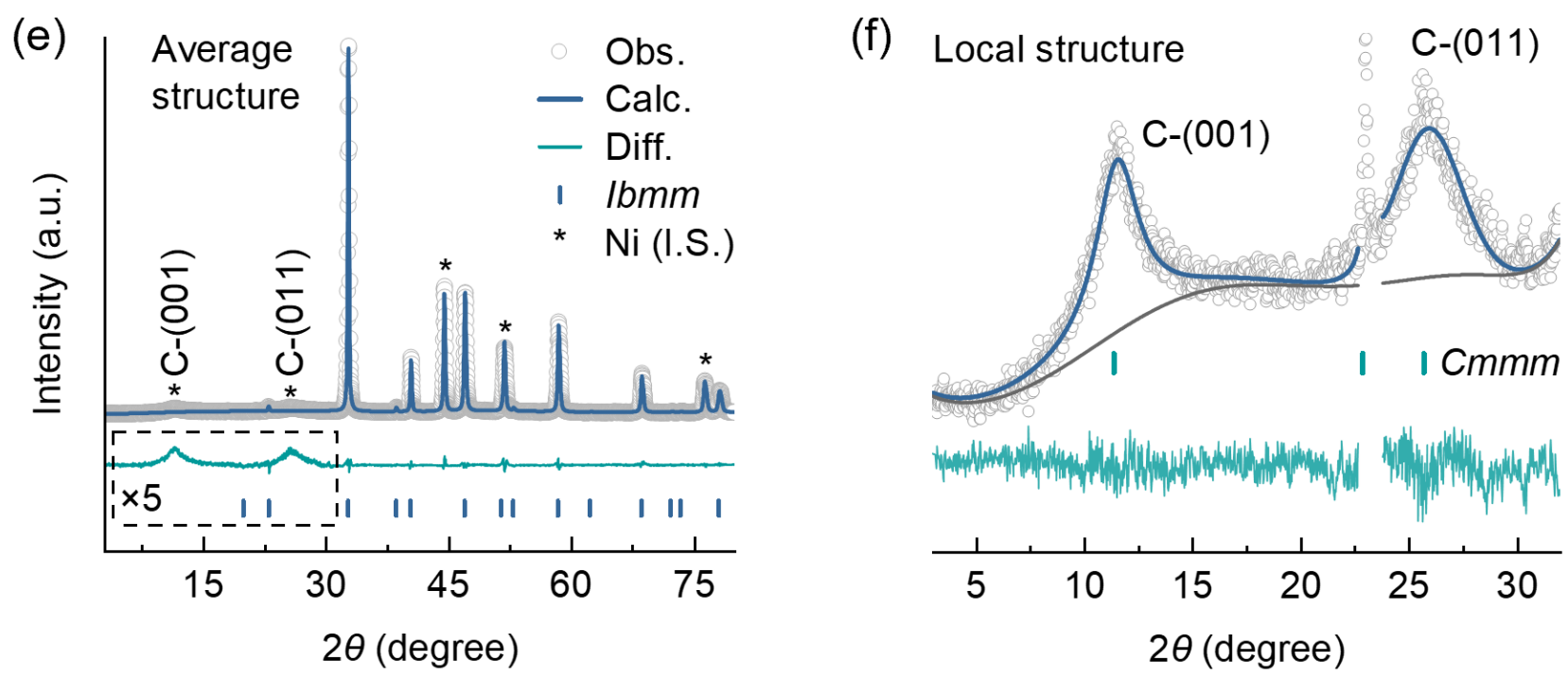

Fig. 1e-1f. **(e)** Rietveld refinement of $La_{0.4}Ca_{0.4}TiO_{3-\delta}$ based on the average *Ibmm* structure (ICSD-245543). Discrepancies between the observed and calculated diffraction patterns near $2\theta = 11.5°$ and 25.6° arise from microstructural features consistent with *Cmmm* symmetry that coexist with *Ibmm* phase. Obs. in the figure denotes the observed pattern, Calc. the calculated pattern, Diff. the difference pattern, and Ni (I.S.) the reflection positions of the Ni internal standard. **(f)** Rietveld refinement of $La_{0.4}Ca_{0.4}TiO_{3-\delta}$ based on the *Cmmm* structure (ICSD-245545). Broadened superstructure reflections assigned to C-(001) and C-(011) evidence short-range order of the alterlattice.

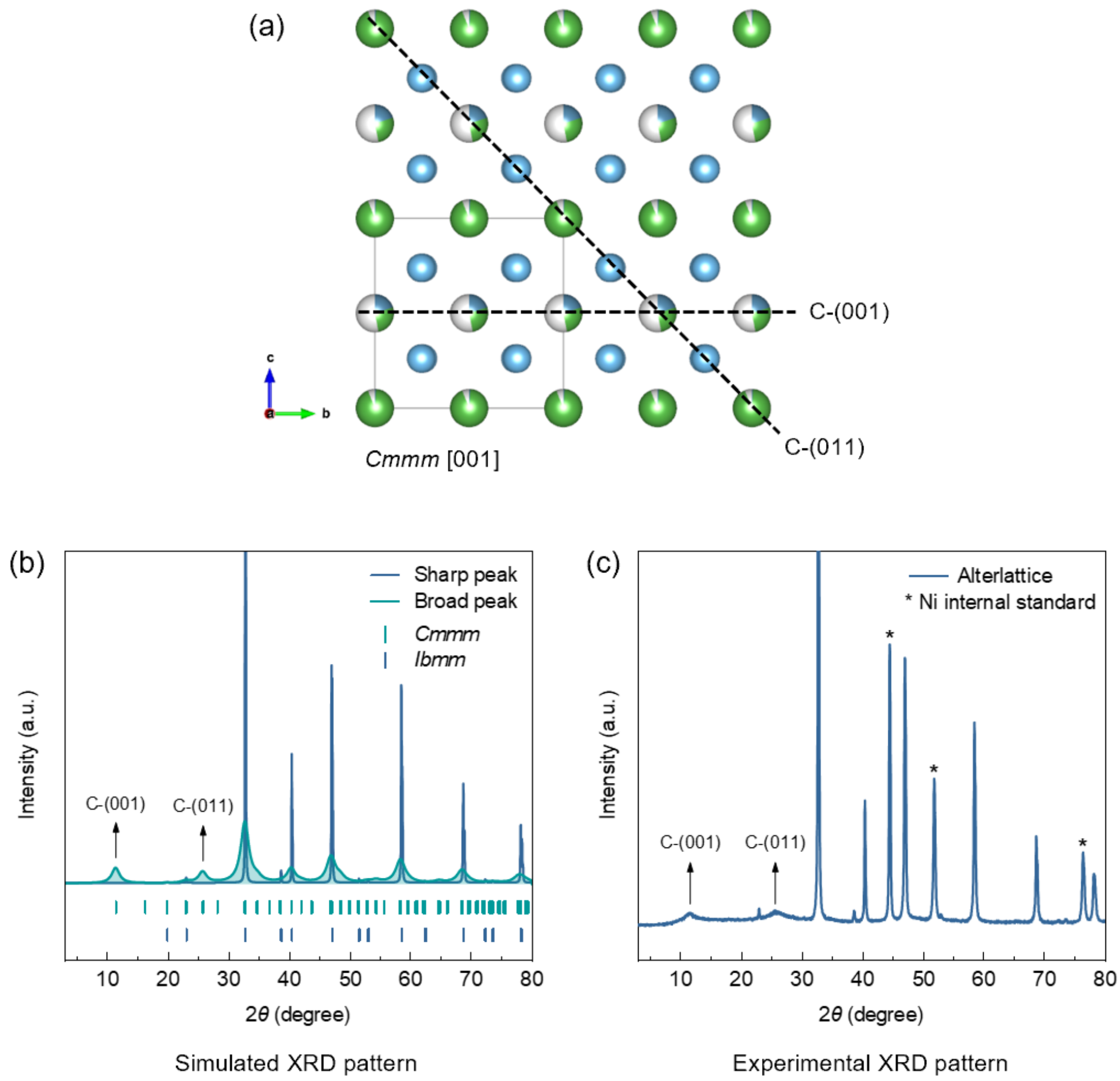

Figure S5 | Simulated and experimental XRD patterns comparing the *Ibmm* average structure with the *Cmmm* **short-range superstructure contributions in the alterlattice. (a)** Projected *Cmmm* structural model viewed along the [001] direction, showing the real-space origin of the *Cmmm*-related C-(001) and C-(011) superstructure reflections in the alterlattice. **(b)** The simulated patterns, obtained using the GSAS program, clarify the structural origins of the diffraction features. Sharp Bragg peaks are dominated by the orthorhombic *Ibmm* average structure, defining the long-range lattice. In contrast, the *Cmmm* A-site vacancy ordered model accounts for the broadened reflections, particularly those below $2\theta = 20°$. **(c)** The experimental pattern exhibits strong, sharp *Ibmm* main reflections, whereas only two broadened *Cmmm*-related superstructure reflections, C-(001) and C-(011), are observed. **Note**: Asterisks denote peaks from the Ni internal standard. This difference between the simulated and experimental patterns indicates that the *Cmmm* component mainly reflects microscopic A-site vacancy chemical ordering. The associated local distortions are therefore either short-ranged or averaged into the long-range *Ibmm* structure. The broadening of the low-angle superstructure reflections arises from orthogonally oriented short-range domains and their 90° rotational intergrowth, which produce overlapping diffraction signals in the powder pattern. The absence of sharp, high-angle reflections characteristic of a long-range *Cmmm* phase confirms that the alterlattice does not constitute a conventional long-range chemically ordered phase.

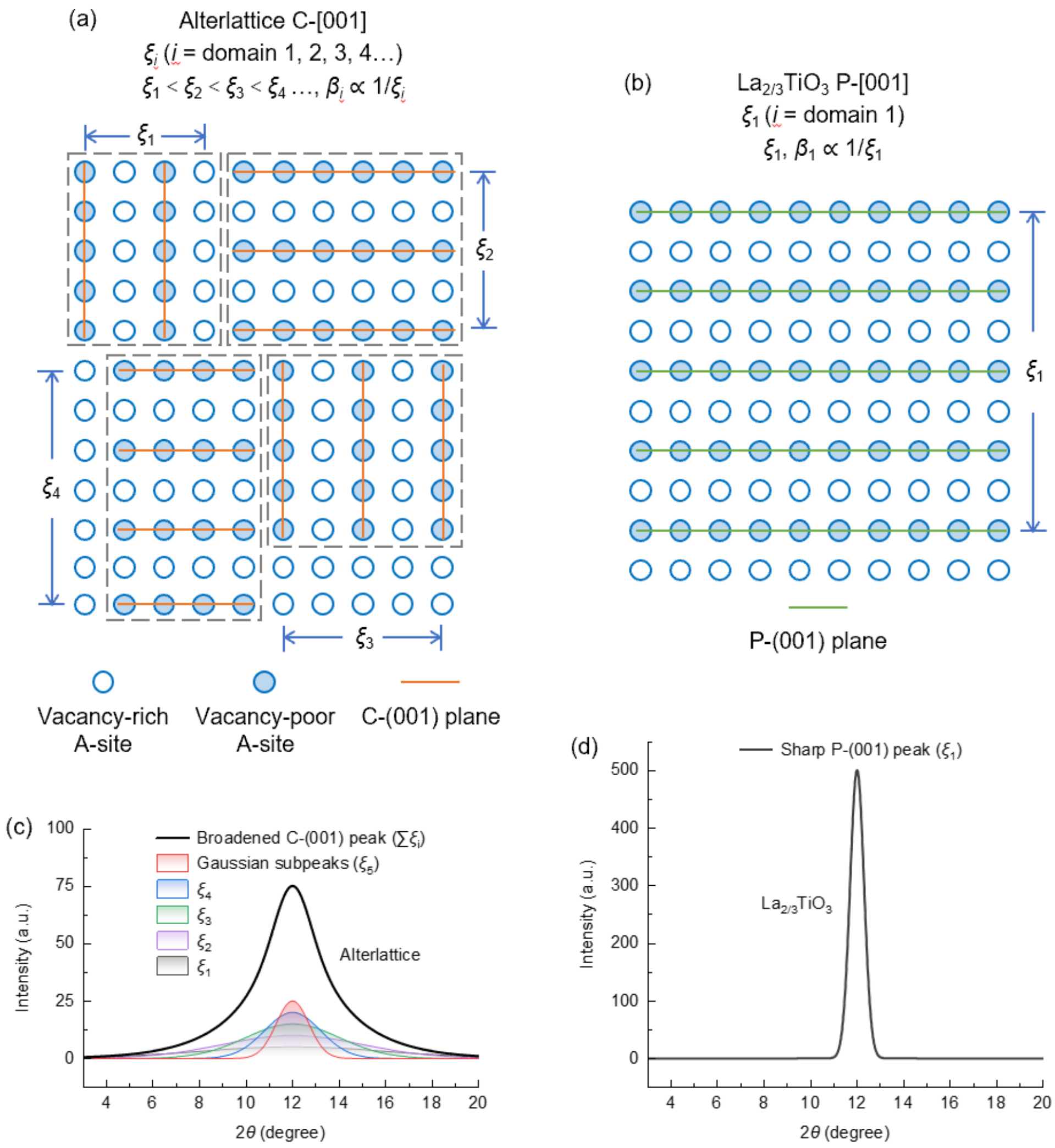

Figure S6 | Schematic origin of broadened superstructure reflections in the alterlattice. (a) Schematic illustration of short-range *Cmmm* A-site vacancy ordered domains in the alterlattice viewed along C-[001]. The local domains have finite and distributed superstructure correlation lengths, denoted as ξ_i (i = 1,2,3,4…). Their approximately orthogonal orientations and rotational intergrowth generate multiple partially overlapping superstructure diffraction contributions. Because each local domain has a limited correlation length, these contributions do not form a single sharp long-range Bragg reflection, but instead give weak and broadened superstructure intensity. The peak width follows approximately $\beta_i \propto 1/\xi_i$. **(b)** Schematic illustration of long-range A-site vacancy ordering in $La_{2/3}TiO_3$ viewed along P-[001]. In contrast to the alterlattice, the vacancy ordering can be approximated by one dominant long correlation length, ξ_1, which produces a much sharper superstructure reflection. **(c)** Schematic representation of the broadened low-angle superstructure peak in the alterlattice. The broad peak can be regarded as the superposition of multiple nearby Gaussian-like diffraction contributions with different intensities and widths. These components originate from *Cmmm* short-range ordered domains with distributed local correlation lengths and different orientations. The broadening mainly reflects finite correlation lengths and orientational intergrowth of A-site vacancy chemical ordering, rather than overall lattice strain. **(d)** Schematic sharp superstructure peak of long-range ordered $La_{2/3}TiO_3$. The longer-range A-site vacancy ordering gives a narrower Bragg-like reflection. The comparison indicates that the low-angle broadened superstructure reflections in the alterlattice most likely originate from short-range A-site vacancy chemical ordering and orthogonal orientational intergrowth, rather than from a long-range single-phase Cmmm structure or overall lattice distortion.

Comments 3.5: In Figs. 2a and 2g the entire structure appears to display contrast features corresponding to Cmmm, especially in the thin regions at the right edge of Fig. 2a and the bottom edge of Fig. 2g. By extracting intensity information from these thin regions, one could confirm the atomic count within the linear atom number-contrast regime and provide a more quantitative description of the intergrowth structure. Combining different projection directions would also help. Perhaps ptychography could be employed to reveal the 3D structure, even though the z-resolution is much lower.

Response 3.5: We thank the reviewer for this constructive suggestion. In the original manuscript, the *Cmmm* contrast features in Figures 2a and 2g were mainly discussed qualitatively. We have now added integrated HAADF-STEM column-intensity analysis from thin regions viewed along both I-[001] and I-[100], and revised the related structural discussion to provide a more quantitative description of the local intergrowth structure.

(i) We extracted intensity information from the thin regions at the right edge of Fig. 2a and the bottom edge of Fig. 2g, where the projected thickness is lower and the HAADF-STEM intensity is closer to the linear atom-number contrast regime (the added Fig. S16, Fig. S17, and the revised manuscript, lines 285–291). The analysis includes atomic-column localization, ROI-based integrated intensity extraction, and comparison of column-intensity variations associated with A-site vacancy ordering.

(ii) The added analysis shows that the *Cmmm* contrast is not limited to isolated local defects, but reflects short-range A-site-vacancy ordered domains embedded within the *Ibmm* average lattice (the revised Supporting Information, lines 258–298). By combining the I-[001] and I-[100] projections, we obtained complementary information on the lateral and depth-related distribution of the local domains, providing a more constrained description of the three-dimensional intergrowth geometry of the alterlattice. The I-[001] (Fig. S16d) and I-[100] (Fig. S17d) analyses indicate that the Cmmm superstructure domains are confined to nanoscale volumes of approximately 3×3×3 nm^3 and 3.5×3.5×3.5 nm^3, respectively. These domain sizes are close to the average apparent ordering correlation length of about 3.4 nm estimated from Scherrer analysis of the C-(001) superstructure reflection in the XRD pattern. The agreement between the local HAADF-STEM intensity analysis and the reciprocal-space XRD estimate supports the model that the alterlattice consists of three-dimensionally intergrown Cmmm domains with nearly cubic geometric sizes.

We also appreciate the reviewer's insightful suggestion regarding ptychography-based three-dimensional structural analysis. This approach may be useful for future efforts to further clarify the 3D intergrowth geometry of the alterlattice, especially when combined with projection-dependent STEM and diffraction analyses, although its z-resolution remains more limited than the in-plane resolution. We have added this method as a possible future direction in the final part of the Conclusion (the revised manuscript, lines 496–499). Beyond the present alterlattice system, this suggestion also provides an important methodological direction for readers studying other materials with nanoscale local ordering, short-range structural correlations, or intergrown domain networks.

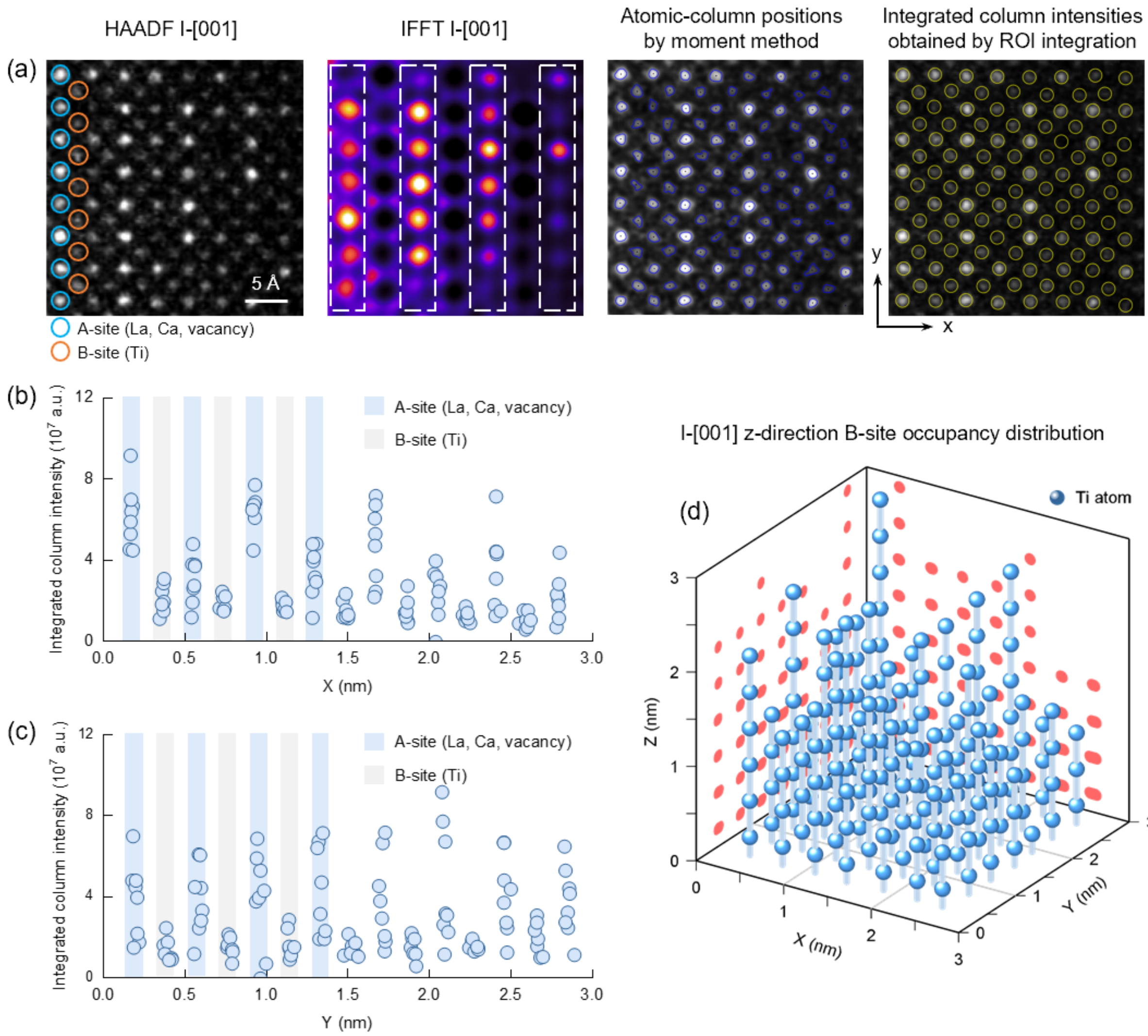

Figure S16 | Quantitative HAADF-STEM intensity analysis of the I-[001] *Cmmm* superstructure domain. (a) From left to right, atomic-resolution HAADF-STEM image of a thin region in $La_{0.4}Ca_{0.4}TiO_{3-\delta}$ viewed along I-[001], IFFT image reconstructed using only the superstructure spots, atomic-column positions determined by the moment method, and ROI-based integrated column intensity map. The selected thin region falls within the approximate linear atom number-contrast regime. The IFFT image highlights a single-**q** *Cmmm* superstructure domain embedded in the average perovskite framework. The moment-fitted atomic-column coordinates were used as the centers for ROI integration of the HAADF intensity. **(b)** Integrated column intensity distribution plotted as a function of x. The alternating A-site and B-site columns show distinct intensity levels, mainly arising from the different average atomic numbers of the A-site columns, La/Ca/vacancy, and the B-site Ti columns. **(c)** Integrated column intensity distribution plotted as a function of y. The projected intensity modulation further confirms that the contrast variation is spatially correlated with the *Cmmm* superstructure domain rather than random imaging noise. **(d)** I-[001] intensity-derived z-direction B-site occupancy distribution. The z-direction Ti occupancy was estimated semi-quantitatively from the integrated B-site column intensities using the thin-specimen approximation, ($I_{col} \propto N_{Ti}Z_{Ti}^{\alpha}$). The obtained B-site occupancy distribution indicates a nanoscale *Cmmm* domain with an approximate spatial extent of about 3×3×3 nm^3.

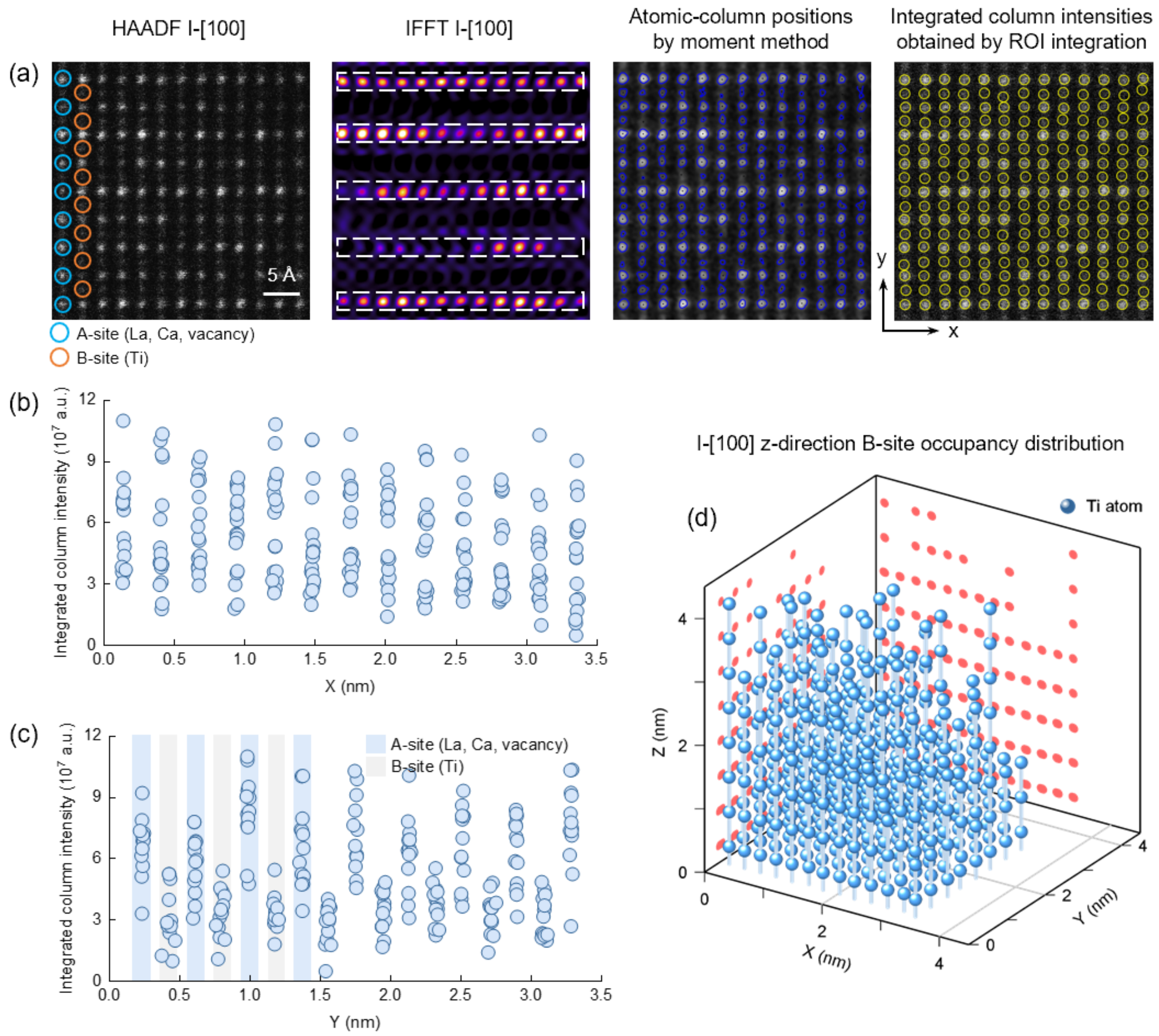

Figure S17. Quantitative HAADF-STEM intensity analysis of the I-[100] *Cmmm* superstructure domain… (the complete figure caption is provided in the revised Supporting Information, lines 296–312)

Comments 3.6: The insets in Fig. 2a and 2g do not correspond with the selected areas in the original images.

Response 3.6: We thank the reviewer for pointing this out. In the original manuscript, the inset images in Figs. 2a and 2g were not fully aligned with the marked regions in the corresponding original images because of incomplete alignment during figure preparation.

We have rechecked the original STEM-HAADF data and replaced the inset images with correctly matched enlarged regions (the revised Figs. 2a and 2g).

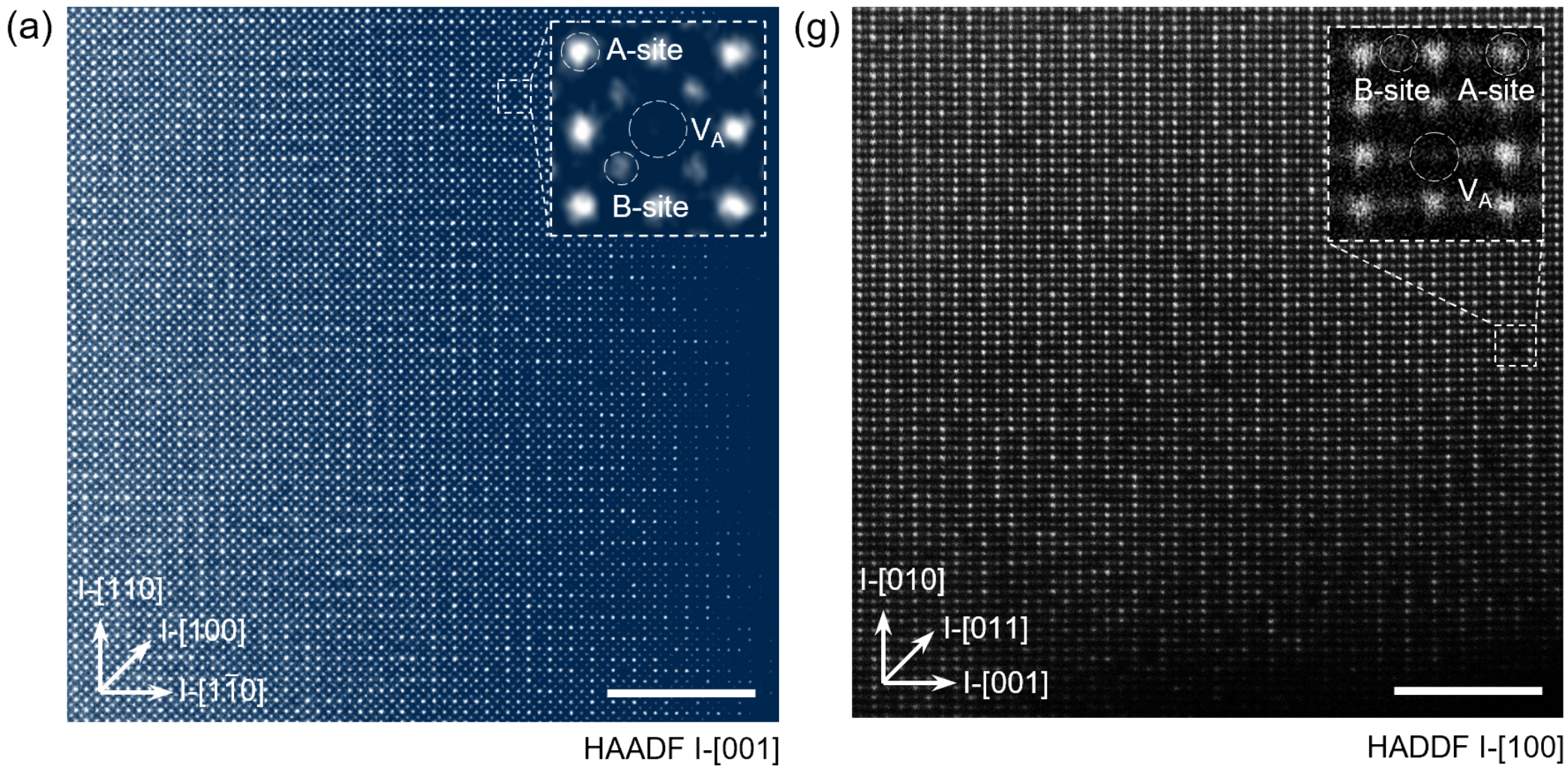

Figs. 2a and 2g. HAADF-STEM images along the I-[001] and I-[100] zone axes (scale bar, 5 nm). The insets schematically show typical positions of A-site cations and B-site cations, and A-site vacancies within a unit cell.

Comments 3.7: In Fig. 3d, did the authors simply overlay three simulated SAED patterns? Do the circles indicate the same d-values? If so, the camera length for the Ibmm [001] pattern appears inconsistent with the two left pattern.

Response 3.7: We thank the reviewer for pointing out the unclear description. In the original manuscript, the construction of the simulated SAED pattern in Figure 3d was not described clearly. We have modified Fig. 3d, added a note to the figure caption to emphasize that the circles marked in the simulated and experimental SAED patterns are compared using the same d-value (the revised manuscript, lines 250–251), and added the simulation details to the Methods section (the revised manuscript, lines 586–590).

(i) The revised description clarifies that Figure 3d was generated by overlaying the simulated SAED patterns of *Cmmm* [010], *Cmmm* [100], and *Ibmm* [001] on the same reciprocal-space scale. This overlay is used to represent the diffraction contribution from multiple orientation domains within the selected-area aperture, rather than an arbitrary superposition. The black circles indicate the same reciprocal-space radius, corresponding to the same 1/d scale. The yellow circles mark equivalent half-integer superstructure reflections from the *Cmmm* short-range domains.

(ii) We also clarify that the inconsistency noted by the reviewer arose from an unintended scaling mismatch during figure assembly in the previous version, rather than from independently rescaled simulations. In the revised Fig. 3d, the reciprocal-space calibration of the *Ibmm* [001] simulated pattern has been corrected so that all simulated patterns are displayed on the same 1/d scale.

These revisions clarify the overlay procedure and support the assignment of the additional SAED spots to orthogonally oriented *Cmmm* short-range superstructure domains.

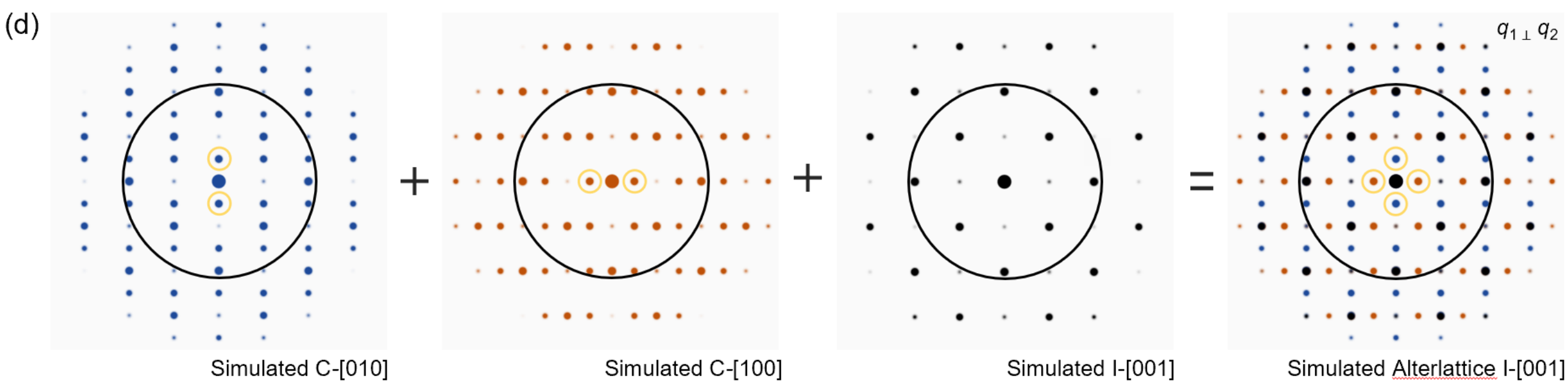

Fig. 3d Simulated I-[001] electron diffraction pattern of the alterlattice, obtained by overlaying the C-[010] and C-[100] diffraction patterns of $La_{0.6}Ca_{0.1}TiO_3$ (space group *Cmmm*, V_A ordered structure with a modulation wavevector) and $La_{0.4}Ca_{0.4}TiO_3$ (space group *Ibmm*). This mixed simulation reproduces the key features observed in the experimental I-[001] diffraction pattern of the alterlattice. **Note**: the white circle in (c) and the black circles in (d) mark the same radius in reciprocal space, corresponding to an identical 1/d scale.

Comments 3.8: Lattice/basis fluctuations can be observed at the bottom of Fig. 3b, the top-left corner and top-middle of Fig. 3f, and the middle of Fig. S3. Do these distortions affect the photomagnetic properties of the material?

Response 3.8: We thank the reviewer for raising this point. Local lattice or basis fluctuations are observed at the bottom of Figure 3b, the upper-left and top-middle regions of Figure 3f, and the central region of Figure S3. In the revised manuscript, we further discuss their possible connection with the photomagnetic behavior of the alterlattice system. These distortions reflect nanoscale structural heterogeneity associated with A-site-vacancy short-range ordering, interleaved orthogonal domains, local La and O concentration variations, and strain release near domain boundaries. We have added the related discussion to the revised manuscript (lines 417–419, 441–445, 489–494).

(i) We consider these local distortions as structural factors that modulate and amplify light-induced local magnetic exchange interactions, rather than as the sole origin of the photomagnetic effect. By modifying Ti-O bond lengths, Ti-O-Ti bond angles, and Ti 3*d*-O 2p-Ti 3*d* orbital overlap, they generate distributed local exchange pathways with different strengths. Such strong coupling among octahedral rotations, lattice distortions, orbital states, and magnetic exchange is well established in perovskite oxides, including $LaMnO_3/SrTiO_3$ superlattices and $RTiO_3$ titanates.

(ii) These distorted and defect-associated regions may also favor photocarrier localization and stabilize Ti^{3+}-like localized states or small polarons. This interpretation is consistent with previous studies showing that oxygen vacancies in $SrTiO_3$ can trap Ti^{3+} small polarons through electron-lattice coupling. In our system, local lattice or basis fluctuations may therefore help stabilize photoinduced localized Ti^{3+} states and enhance short-range antiferromagnetic exchange through Ti^{3+}-O-Ti^{4+} or Ti^{3+}-O-Ti^{3+} pathways, contributing to the pronounced decrease in macroscopic magnetization under illumination.

Accordingly, we describe these local distortions as important structural factors that promote carrier localization and short-range antiferromagnetic correlations within the alterlattice.

Comments 3.9: Some figures in the main text and captions only present their conclusions. The authors are suggested to providing detailed explanations for each figure to improve readability.

Response 3.9: We appreciate the reviewer's constructive suggestion. In the original manuscript, several figure captions mainly summarized conclusions without sufficient description of the data features and their connection to the text.

(i) We have revised several figure captions (the revised manuscript, line 94–109, 307–311, 314–318).

(ii) We also moved necessary explanatory text from the previous Discussion section into the Results section, so that the figures, data description, and physical interpretation are presented in a clearer sequence (the revised manuscript, line 147–151, 163–178, 275–277, 291–296…).

These revisions improve the readability of the figures and strengthen the correspondence between the captions, main text, and conclusions.

Comments 3.10: The TEM sample preparation method and imaging conditions (e.g., convergence angle, camera length, etc.) are not provided. These conditions are essential for interpreting image contrast.

Response 3.10: We thank the reviewer for raising this point. The revised text now includes the TEM/STEM specimen preparation procedure (the revised manuscript, line 532–541), including gentle grinding of the sintered ceramic sample, dispersion in anhydrous ethanol, ultrasonic treatment, and drop-casting onto carbon-supported copper grids. We also added the key imaging parameters used for atomic-resolution STEM characterization, including the camera length of 60 mm, probe convergence semi-angle of 20.3 mrad, probe current of 40 pA, probe size of 0.08 nm, ADF collection angle of 40–160 mrad, and HAADF collection angle of 90–370 mrad. For diffraction analysis, SAED patterns were acquired in TEM diffraction mode at 200 kV using a nominal camera length of 600 mm, with the camera constant calibrated to approximately 150.48 Å mm. These additions provide the necessary experimental basis for interpreting image contrast, atomic-column intensity, and diffraction-spot positions.

Point-by-point responses to the reviewers

I. Reviewer #1 (Remarks to the Author)

Comments: "The authors have seriously replied to all of my questions. I also found the manuscript itself greatly improved. I think that the present manuscript has elucidated the alterlattice strucuture, the photo-induced demagnetization, as well as the direct but phoenomenalogical connection between them. These results are worth publishing. However, I still think that the connection between the alterlattice structure and the photo-induced demagnetization somewhat lacks theoretical base and deep physics. The mechanism that the authors proposed could be one explanation, but not necessarily be it. So I just express my opinion here and leave the final decision to the editor. It is ok to make any decision on this manuscript without contacting me again. A few small comments: Figure S5 is not in the table of contents of the supplementary materials. Some AFM models, such as 1D,2d models, were mentioned without refering to any literatures. The readers may not be familiar with these models."

Response: We sincerely thank the reviewer for the careful reassessment of our revised manuscript and for the thoughtful and balanced evaluation. In the final paragraph of the Discussion, we have outlined directions for future work combining advanced characterization techniques with microscopic theoretical studies to distinguish the proposed mechanism from other possible explanations. We have also included Fig. S5 in the table of contents of the Supplementary Information and cited relevant literature when introducing the one- and two-dimensional antiferromagnetic correlation models.

II. Reviewer #2 (Remarks to the Author)

Comments: "The article is now ready to be accepted for publication in Nat.Communications"

Response: We sincerely thank the reviewer for the favorable assessment and appreciate the time and care devoted to evaluating our manuscript.

III. Reviewer #3 (Remarks to the Author)

Comments: "All my concerns have been addressed by the authors. I have no further comments and recommend acceptance of this paper in the current form."

Response: We sincerely thank the reviewer for carefully reassessing our revised manuscript and appreciate the constructive comments, which helped us improve the clarity and quality of the work.
